# Spectral Transmittance of Daily Disposable Contact Lenses: Variability in Ultraviolet Blocking

**DOI:** 10.3390/ma18204784

**Published:** 2025-10-20

**Authors:** Arief Abdurrazaq Dharma, Sachiko Kaidzu, Yoshihisa Ishiba, Tsutomu Okuno, Masaki Tanito

**Affiliations:** 1 Department of Ophthalmology, Shimane University Faculty of Medicine, Enya 89-1, Izumo 693-8501, Shimane, Japan; abdurrazaqacha@gmail.com (A.A.D.); kecha@med.shimane-u.ac.jp (S.K.); 2 Technology Development Department, Yamamoto Kogaku Co. Ltd., 3-25-8 Chodo, Higashi-Osaka 577-0056, Osaka, Japan; ishiba.yoshihisa.ile@osaka-u.ac.jp; 3 Institute of Laser Engineering, Osaka University, 2-6 Yamadaoka, Suita 565-0871, Osaka, Japan; 4 National Institute of Occupational Safety and Health Japan, 6-21-1 Nagao, Kawasaki 214-8585, Kanagawa, Japan

**Keywords:** ultraviolet radiation, contact lenses, spectral transmittance, lens power, oxygen transmissibility, daily disposable lenses

## Abstract

Ultraviolet radiation (UVR) is a well-established risk factor for ocular diseases; however, the ultraviolet-blocking properties of daily disposable contact lenses remain insufficiently characterized. This study evaluated thirteen commercially available lenses to determine their spectral transmittance across UV-B, UV-A, and visible light ranges using a UV–visible spectrophotometer. The oxygen permeability, central thickness, water content, and FDA material classification of each lens were documented, and oxygen transmissibility was subsequently calculated. A generalized linear mixed model (GLMM) was applied to identify predictors of spectral transmittance. All lenses demonstrated high visible light transmittance (>88%), but exhibited substantial variation in UV attenuation. While several lenses effectively blocked most UV radiation, others transmitted more than 70%. The analysis revealed that lens power was the most consistent predictor of spectral transmittance, with higher minus powers associated with reduced UV-blocking efficacy. Moisture content and material classification also influenced UV protection but had minimal effect on visible light transmission. In conclusion, daily disposable contact lenses vary considerably in their UV-blocking capabilities, and although lens power cannot be altered, consideration of material composition and UV transmittance properties may assist in selecting lenses that provide optimal ocular protection.

## 1. Introduction

Ultraviolet radiation (UVR) is a form of electromagnetic radiation (EMR) with wavelengths between 100 and 400 nanometers (nm), lying just beyond the visible light spectrum (400–700 nm) and shorter than infrared (700–1200 nm). UVR possesses higher photon energy compared to visible or infrared light, implying a heightened potential for biological damage. The ultraviolet (UV) spectrum is subdivided into three regions: UV-A (315–400 nanometers [nm]), UV-B (280–315 nm), and UV-C (100–280 nm). As sunlight traverses the atmosphere, a significant portion of UV-C and approximately 90% of UV-B radiation are absorbed by the ozone layer, water vapor, oxygen, and carbon dioxide. Consequently, the UVR that reaches the Earth’s surface consists predominantly of UV-A, with a smaller fraction of UV-B—approximately ten times less in intensity than UV-A [1]. UV exposure, primarily in the UV-B and UV-A wavelengths, is established as a major risk factor for a wide range of anterior and posterior eye disease [2].

Acute, high-intensity UV exposure has been shown to induce photokeratitis, a condition characterized by inflammatory damage to the corneal epithelium [3]. Pterygium, a fibrovascular conjunctival growth that extends onto the cornea, has been strongly associated with chronic exposure to UV radiation, particularly in regions with high UV index [4]. Beyond the ocular surface, UV-B can penetrate the crystalline lens, where protein denaturation and oxidative stress accelerate cataract formation [5]. In the posterior segment, cumulative UV exposure may act additively with high-energy visible (blue) light in the development of age-related macular degeneration (AMD), primarily through photochemical injury and reactive oxygen species-mediated apoptosis in retinal pigment epithelium cells [6]. Furthermore, chronic UV exposure is an established risk factor for conjunctival squamous cell carcinoma (SCC), with incidence significantly higher in equatorial regions and among individuals with prolonged outdoor occupations [7].

In addition to sunglasses, UV-blocking contact lenses have been shown to provide effective ocular surface protection against UVR by directly shielding the cornea [8]. In contrast to sunglasses, which may expose the periocular tissues to scattered or reflected UV rays, particularly from lateral angles or ground surfaces, contact lenses provide continuous coverage of the exposed ocular surface. These spectacles are particularly beneficial for individuals participating in sports or outdoor activities where sunglasses might be impractical. However, it is imperative that such lenses adhere to recognized certification standards (e.g., ISO 18369-3 [9], ANSI Z80.20 [10]) to ensure that their UV-blocking performance is adequate to prevent potential phototoxic damage.

While the ability to protect against UV rays is a salient attribute, contact lenses must also permit adequate oxygen transmissibility (Dk/t) to maintain corneal health. Dk/t is a pivotal performance metric, as it quantifies the amount of oxygen that can permeate the lens to the cornea during wear. Ensuring an adequate oxygen supply is imperative to avert hypoxic complications, including corneal edema and neovascularization [11,12]. However, Dk/t alone does not determine overall lens performance. Broader material attributes such as wettability, comfort, and resistance to protein deposition have also been highlighted as important determinants of long-term clinical success, since they influence wearing time, susceptibility to surface fouling, and overall patient tolerance [13]. Lenses with high Dk/t values support extended and safer wearing times, enhancing visual performance, comfort, and overall ocular health. Advancements in materials science, particularly the development of silicone hydrogel technology, have led to significant improvements in Dk/t. This advancement has effectively mitigated the hypoxic limitations that were prevalent in earlier hydrogel designs [14].

UVR has been demonstrated to contribute to a spectrum of ocular pathologies, thereby reinforcing the clinical relevance of protective strategies such as sunglasses and UV-blocking contact lenses. Additionally, material innovations, particularly silicone hydrogels, have enhanced Dk/t without compromising safety [2]. Despite the pervasive marketing claims, there is a paucity of independent data on the actual spectral transmittance of commercially available contact lenses across the UV-B, UV-A, and visible light ranges. Furthermore, the extent to which lens material, design, and optical power influence UV protection remains unclear.

The objective of this study was to evaluate and compare the spectral transmittance characteristics of a range of commercially available contact lenses. Rather than establishing definitive clinical guidance, the present work provides basic data that could serve as a foundation for future recommendations regarding UV-related ocular protection.

## 2. Materials and Methods

### 2.1. Contact Lenses Material

This study examined the spectral transmittance characteristics of daily disposable soft contact lenses commonly available in Japan. The study encompassed a total of 13 contact lens models from six manufacturers: Johnson & Johnson Vision (Jacksonville, FL, USA), Menicon Co., Ltd. (Nagoya, Japan), CooperVision, Inc (Victor, NY, USA), Alcon Laboratories (Fort Worth, TX, USA), Bausch + Lomb (Rochester, NY, USA), and SEED Co., Ltd. (Tokyo, Japan). The lenses exhibited variability in their material composition, encompassing both hydrogel and silicone hydrogel categories, with differences in water content and ionic properties, as designated by FDA Material Groups I–V. The classification of groups I–IV adheres to the original FDA scheme, with the basis of this classification being water content and ionic charge. Group V is used to denote silicone hydrogel materials. These materials were not included in the original FDA hydrogel classification, yet they are widely recognized as a separate category in contemporary lens research [15]. Detailed chemical compositions of the lens materials, including primary monomers, hydrophilic components, and classification type, are provided in Supplementary Table S1.

The contact lenses were tested at spherical powers of +3.00 D, −3.00 D, and −6.00 D, depending on the availability of the specific model. All lenses were new and unused, and no conditioning or pre-treatment was applied prior to measurement. A comprehensive array of technical specifications was obtained from manufacturer sources or published literature. These specifications encompass critical parameters such as material name, oxygen permeability (Dk), central thickness (t), and water content.

### 2.2. Spectral Transmittance Measurement

Spectral transmittance was measured using a Hitachi U-4100 UV–Visible spectrophotometer (Hitachi High-Tech Corporation, Tokyo, Japan) without an integrating sphere. This configuration was selected because it enables measurement of short-wavelength ultraviolet radiation down to 200 nm, which is essential for full-range UV analysis (200–400 nm). The measurement range was set from 200 to 800 nm, thereby covering the UV-B (280–315 nm), UV-A (315–400 nm), and visible light (400–700 nm) regions.

Each lens was immersed in physiological saline at room temperature and placed within a quartz cuvette during measurement. The central optical zone was isolated using a 3 mm aperture, approximating the physiological pupil diameter under daylight (photopic) conditions. To ensure accurate beam alignment, each lens was carefully centered within the beam path after punching an 8 mm circular section using a dermatological punch and securing it in a 6 mm aperture holder. Measurements were repeated several times to confirm stability and reproducibility, and only consistent readings were recorded as final data. Because the punched lens section remained nearly flat when hydrated in saline, the influence of residual curvature on light transmittance was considered negligible.

The measurement of each sample was conducted in duplicate, employing two lenses for each contact lens model. It is imperative to note that no data smoothing or normalization was applied. The raw transmittance data were recorded in their original state and subsequently analyzed. The complete raw spectral transmittance data (200–800 nm) for all lens models and powers are presented in Supplementary Table S2.

### 2.3. Calculation of Oxygen Transmissibility (Dk/t)

Dk/t was calculated as the ratio of the oxygen permeability of the lens material (Dk, expressed in Barrer units) to its central thickness (t, in millimeters). By convention, Dk/t values are reported in units of 10^−9^ (ml O_2_)/(cm^2^ s mmHg), derived from the definition of one Barrer. In this study, the central thickness was derived from the −3.00 D lens, which is widely regarded as the standard reference. This parameter provides a clinically relevant indication of the lens’s capacity to deliver oxygen to the corneal surface during wear, which is critical for maintaining corneal health, especially under extended wear conditions.

This Dk value came either from the manufacturer data or literature on each of the lens materials (e.g., etafilcon A, senofilcon A, etc.) and consequently, the center thickness values were pulled from the lens technical specification data at −3.00 D power that was available. When complete data were not available, models were then calculated using other resources that had complete data and stated accordingly.

### 2.4. UV Labeling and Regulatory Reference

Manufacturer-provided UV-transmittance data, when available, were used for comparison. UV-A and UV-B transmittance were calculated according to ISO 18369-3 [9] wavelength definitions (UV-B: 280–315 nm; UV-A: 316–400 nm). According to ISO 18369-3:2017, soft contact lenses are classified as UV-blocking Class I when UV-B and UV-A transmittance are below 1% and 10%, respectively, and as Class II when below 5% and 30%. Lenses exceeding these limits cannot be labeled as UV-blocking. These thresholds provided the reference framework for interpreting UV-protective performance in this study.

### 2.5. Statistical Analysis

Statistical analysis was conducted using JMP^®^ Student Edition 18 (SAS Institute Inc., Cary, NC, USA). To examine the factors potentially affecting spectral transmittance, a generalized linear mixed model (GLMM) was applied. In the GLMM framework, the lens model was specified as a random effect to account for repeated measurements within each lens type, while lens power, oxygen permeability (Dk), water content, and FDA material group were treated as fixed effects. Separate models were constructed for UV-B, UV-A, and visible light transmittance as dependent variables. This mixed-model framework was selected instead of a traditional ANOVA because it allows simultaneous evaluation of fixed and random effects, accommodates non-independent observations, and provides a more realistic representation of variability among different lens materials and designs.

## 3. Results

A total of 13 daily disposable contact lens models from six manufacturers were evaluated for key physical and material characteristics (Table 1). The oxygen permeability values exhibited significant variation, ranging from 12.0 in 1DFUP to 140.0 in Delefilcon A (DT1). Silicone hydrogel lenses, including Stenfilcon A (MD), Delefilcon A (DT1), and Kalifilcon A (AL1D), have been shown to exhibit higher Dk values in comparison to conventional hydrogel lenses.

The central thickness (CT) measurements exhibited a range from 0.07 mm (Kalifilcon A, AL1D; SEED Ionic Bond, 1DPMP) to 0.09 mm (Omafilcon A, P1D; Delefilcon A, DT1; Hilafilcon B, M1DP). Lenses with thinner profiles, particularly AL1D (0.07 mm), demonstrated high Dk/t (162.85), despite exhibiting slightly lower Dk compared to DT1. This observation highlights the combined influence of material and thickness on Dk/t.

The moisture content (MC) of the samples ranged from 33% (Delefilcon A, DT1) to 78% (Nesofilcon A, BOD). In comparison, hydrogel lenses demonstrated a higher MC, which is consistent with the water-binding characteristics of hydrogel polymers. However, certain high-MC hydrogels (e.g., Nesofilcon A) exhibited lower Dk/t values compared to low-MC silicone hydrogels. This finding suggests that water content alone is not the sole determining factor of oxygen performance.

**Figure 1:**
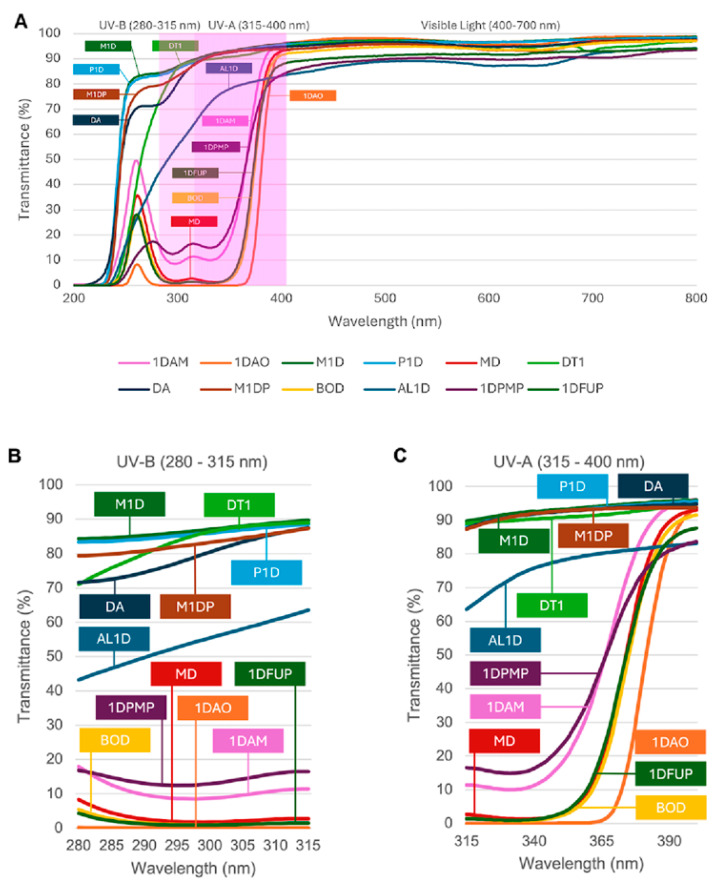
Spectral transmittance profiles of the measured soft contact lenses across three wavelength regions: (**A**) full spectrum (200–800 nm), (**B**) UV-B (280–315 nm), (**C**) UV-A (315–400 nm). Data were collected using −3.00 D lenses. The shaded area in panel A indicates the ultraviolet region (280–400 nm). Each curve in the figure represents the mean transmittance across the tested spectrum for a specific lens model.

The mean spectral transmittance curves shown in Figure 1 provide a clear overview of how the tested contact lenses filter light. All measurements used lenses with a standardized spherical power of −3.00 D to ensure that data were comparable. In the ultraviolet region, lenses like 1DAO, BOD, 1DFUP, 1DAM, and 1DPMP showed similar and strong attenuation in both the UV-B (280–315 nm) and UV-A (315–400 nm) ranges. This indicates effective UV blocking capability. AL1D had a different spectral profile with unique transmission traits in the UV region. On the other hand, lenses labeled M1D, P1D, M1DP, DA, and DT1 showed little attenuation in both UV-B and UV-A wavelengths, meaning they provided poor intrinsic UV blocking. Throughout the visible spectrum (400–700 nm), all lenses displayed consistently high transmittance, with minimal differences in optical transmission across models.

As shown in Table 2, the average transmittance values in the UV-B range were measured and reviewed for all lenses. These values were then compared to the manufacturer-provided data, when available. In addition, each measured value was evaluated according to ISO 18369-3 [9]:2017 UV-blocking thresholds (Class I: UV-B < 1% and UV-A < 10%; Class II: UV-B < 5% and UV-A < 30%), allowing direct classification of lenses based on their measured spectral performance. For 1DAM, the manufacturer reported a UV-B transmittance of less than 3%. The measured values were 3.2% at −6.00 D, 10.7% at −3.00 D, and 5.5% at +3.00 D. In the case of 1DAO, both the manufacturer-reported and measured values were below 1%. Experimental measurements showed values of less than 0.1% for both −3. The manufacturer’s data for MD indicated UV-B transmittance below 5%, which matched measured values of 3.0% at −3.00 D and 0.7% at +3.00 D. A similar pattern appeared in BOD (1.9% and 1.3%) and 1DFUP (1.8%) at −3.00 D, all reported as less than 5% by their manufacturers. The 1DPMP had a value of less than 50%, with measured values of 21.2% and 9.0% for −3.00 D and +3.00 D, respectively. For lenses such as M1D, P1D, DT1, DA, M1DP, and AL1D, no UV-B data was available from the manufacturer. The measured values for these lenses ranged from 47.6% to 86.7%, depending on the lens power.

Table 2 also shows substantial variation in mean UV-A transmittance values among the tested lenses. For 1DAM, the manufacturer reported a transmittance value of below 19%, while the measured values were 35.9% at −6.00 D, 44.1% at −3.00 D, and 38.8% at +3.00 D. 1DAO, reported as less than 4%, yielded measured values of 21.3% at −3.00 D and 18.2% at +3.00 D. The MD and BOD were each reported to transmit less than 50% in the UV-A region. The measured values for MD were 31.0% (−3.00 D) and 26.8% (+3.00 D), while BOD yielded 29.8% and 27.9%, respectively. For 1DPMP, reported as less than 65%, the corresponding measured values were 48.7% (−3.00 D) and 38.2% (+3.00 D). 1DFUP was reported to transmit less than 25%, with a measured value of 30.2% at −3.00 D. For lenses without manufacturer-provided UV-A data, specifically M1D, P1D, DT1, DA, M1DP, and AL1D, measured values ranged from 74.5% to 93.5%.

The values of measured visible light transmittance are also presented in Table 2. The availability of manufacturer-provided visible light transmittance data was limited for the majority of lenses; nevertheless, all measured values exhibited a consistent high level of performance. The 1DAM exhibited the highest values, ranging from 97.3% to 97.6%, across all powers. Levels of M1D and P1D were also found to be similarly elevated, with percentages of 97.4% and 97.1%, respectively. Lenses such as 1DAO (96.3–97.5%), DA (96.4%), and MD (95.5–95.8%) also exhibited elevated transmittance. DT1 and M1DP demonstrated a range of 91.7% to 95.5%, while BOD and 1DFUP exhibited a range of 91.3% to 95.0%. Notably, AL1D exhibited the lowest transmittance within the group, with values of 92.1% at −3.00 D and 88.1% at +3.00 D.

The correspondence between measured transmittance values and manufacturer-reported UV-blocking classes is summarized in Table 3. The categorization of each model was conducted in accordance with ISO 18369-3 criteria, classifying them as compliant (“Matched”), non-compliant (“Unmatched”), or not available (“N/A”) with respect to the UV-B and UV-A regions. As demonstrated in Figure 1, lenses such as MD, BOD, and 1DPMP satisfied their designated ISO classification for both UV-B and UV-A, while 1DAO and 1DFUP adhered exclusively to the UV-B standard. The 1DAM lens exhibited power-dependent deviations, while several models (M1D, P1D, DT1, DA, M1DP, AL1D) lacked UV-blocking data and demonstrated high UV transmittance across both regions.

After adjusting for various covariates using the GLMM, lens power emerged as an important predictor of spectral transmittance across all wavelength regions. In the UV-B range (Table 4), lens power significantly influenced UV-B transmittance (Estimate = −0.76, *p* = 0.002), with higher minus powers associated with increased transmission and reduced blocking capacity. Moisture content showed a negative association (Estimate = −4.12, *p* = 0.01), indicating improved UV-B protection with higher water content. Material group also had a significant effect: Group I lenses transmitted less UV-B than Group V (*p* = 0.04), whereas Group II transmitted more (*p* = 0.04).

In the UV-A range (Table 5), the lens power was significantly associated with UV-A transmittance (Estimate = −0.61, *p* = 0.001), with higher minus powers corresponding to increased transmission and reduced blocking capacity, whereas hyperopic lenses demonstrated lower transmission. Moisture content showed a significant negative effect (Estimate = −3.13, *p* = 0.01), indicating that higher water content was associated with improved UV-A protection. Material group analysis revealed that Group I lenses transmitted less UV-A than Group V (*p* = 0.02), while no significant differences were observed for Groups II or IV compared with Group V.

In the visible light spectrum (Table 6), lens power had a modest but significant association with visible light transmittance (Estimate = −0.21, *p* = 0.005), with higher minus powers linked to slightly increased transmission. No significant effects were observed for oxygen permeability, moisture content, or material group.

## 4. Discussion

This study comprehensively evaluated the spectral transmittance and oxygen-related characteristics of daily disposable contact lenses from multiple manufacturers. The findings indicated significant variability in UV attenuation, with certain lenses exhibiting effective blocking in both UV-B and UV-A ranges, while others demonstrated minimal protection despite high visible light transmittance. Oxygen permeability varied according to material type and central thickness, consistent with the known differences between silicone hydrogel and conventional hydrogel polymers. Importantly, statistical modeling revealed lens power as the most consistent predictor across all wavelength ranges, while moisture content and material group also influenced UV transmittance selectively. These integrated findings form the basis for a more in-depth discussion on how UV blocking, Dk/t, and optical design interact to determine the clinical performance of contact lenses. While the current measurements were performed under strictly axial illumination, real-world ocular exposure involves light entering from multiple directions due to lateral scatter and reflections from surrounding surfaces such as the ground or water. This angular dependence can significantly influence the effective UV shielding provided by contact lenses, particularly for designs with limited peripheral chromophore distribution or minimal edge thickness.

The protective role of UV filtering in preventing degenerative and potentially neoplastic ocular diseases is well recognized. Although many of these lenses are marketed with claims of UV-filtering properties, the extent to which their spectral transmission characteristics translate into meaningful biological protection remains insufficiently characterized. Spectral transmittance assessment provides an objective, wavelength-specific measure of the blocking of UV and visible light that penetrates a lens, directly indicating its potential efficacy in attenuating harmful radiation. In the present study, significant variability was observed among commercially available daily disposable lenses. Some models demonstrated strong attenuation in both UV-B and UV-A regions, while others transmitted more than 70–80% of incident radiation despite being marketed as premium products. These findings are consistent with previous reports indicating that only a subset of modern lenses incorporate effective UV-absorbing chromophores into their polymer matrix [8,16]. Furthermore, the observed discrepancies between manufacturer-provided data and measured values, particularly in the UV-A range, highlight the necessity of independent verification. Some lenses exhibited measured UV-B or UV-A transmittance values that exceeded the manufacturer’s reported specifications, especially at different optical powers. These discrepancies likely reflect power-dependent variations in lens geometry rather than measurement error. According to ISO 18369-3:2017, soft contact lenses are classified as UV-blocking Class 1 when mean UV-B and UV-A transmittance are below 1% and 10%, respectively, and as Class 2 when below 5% and 30%. However, the standard does not define numerical tolerances for measurement deviations, and minor differences between measured and manufacturer-reported data may therefore arise from methodological or instrumental variability rather than formal non-compliance. Nevertheless, labeling that omits power-specific information could lead to an overestimation of UV-blocking performance in higher minus powers, underscoring the need for standardized, power-dependent certification and more transparent manufacturer reporting [9]. Given the established role of UV-A exposure in oxidative stress–mediated cataractogenesis and retinal damage [5,6], ensuring accurate and consistent UV-blocking claims remains clinically relevant. A statistical analysis further demonstrated that moisture content and material group had a significant impact on UV transmission, suggesting that material chemistry interacts with optical design to determine the overall protective effect.

In addition to spectral filtering, Dk/t remains a critical determinant of contact lens safety and long-term tolerance. In this study, Dk/t exhibited significant variability among the materials examined, with silicone hydrogel lenses demonstrating consistently higher values compared to conventional hydrogels. This observation is consistent with prior research showing that silicone-based polymers enable oxygen diffusion through hydrophobic domains of the matrix, providing superior permeability regardless of water content [12,17]. In contrast, hydrogel lenses primarily rely on water-mediated diffusion, rendering their oxygen performance contingent on water content and thickness. The study demonstrated that lenses with high moisture levels, such as nesofilcon A, exhibited lower Dk/t values compared to thinner silicone hydrogel designs. Central thickness was identified as a pivotal factor in this study. Lenses with reduced thickness exhibited superior Dk/t, despite moderate intrinsic Dk values. This finding aligns with the well-established inverse relationship between thickness and oxygen flux [18,19]. The statistical model further indicated that moisture content and FDA material group exerted a significant influence on UV-B and UV-A transmittance, but not on visible light. This suggests that material chemistry contributes selectively to spectral protection without altering normal visual transmission. Recent polymer engineering strategies, including PEGDA cross-linking in silicone hydrogels, have demonstrated efficacy in enhancing flexibility and optical transparency [20]. Although all lenses maintained visible light transmittance above 88%, this level is slightly lower than the >95% typically reported for most commercial soft lenses [21]. Reduced transmission in the visible spectrum, even within this range, may potentially affect contrast sensitivity or night vision, underscoring the importance of balancing UV attenuation with preservation of visual quality. These findings reinforce the critical importance of achieving an optimal balance between material permeability, geometric design, and chromophore integration for sustaining corneal physiology while simultaneously enhancing UV defense. Notably, Group I hydrogels transmitted less UV-B and UV-A than Group V silicone hydrogels. This likely reflects the incorporation of UV-absorbing chromophores in some Group I materials, whereas silicone hydrogels, despite their superior oxygen performance, do not uniformly include such additives [12]. Thus, oxygen permeability and UV protection should be regarded as distinct material attributes.

**Figure 2:**
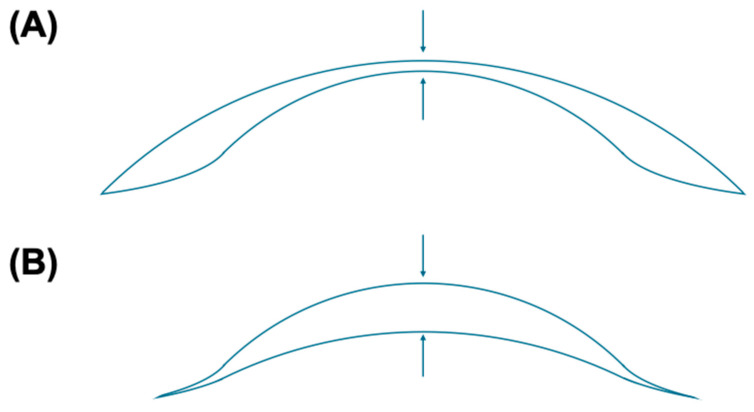
Schematic representation of power-dependent lens geometry and its effect on UV transmission. (**A**) Minus-powered lens has a thinner central zone than a peripheral zone. (**B**) Plus-powered lens has a thicker central zone than a peripheral zone.

In addition to material and thickness, this study identified lens power as the most consistent predictor of spectral transmittance across all wavelength regions. The mixed-model analysis demonstrated that higher minus powers were significantly associated with greater UV-B and UV-A transmittance, indicating reduced blocking capacity at stronger myopic prescriptions. This pattern reflects the geometric characteristics inherent in minus-powered designs, in which the peripheral zones are thicker while the central optical zone—responsible for the majority of light entry under physiological pupil conditions—is markedly thinner. As illustrated in Figure 2, this thinner central region shortens the optical path length and consequently reduces the absorption of UV-attenuating chromophores. In contrast, plus-powered lenses have thicker central zones that enhance UV attenuation. The measurement aperture in this study was designed to approximate the daylight pupil size, emphasizing the central region that dominates overall transmission. These findings refute the earlier assumption that peripheral thickness provides greater UV shielding and instead highlight the importance of central thickness in modulating spectral performance. Concurrent findings have shown that lens geometry in myopic corrections can significantly alter spectral transmission in ways relevant to ocular photoprotection [22]. Of particular significance was the observation that while moisture content and material group exhibited significant effects, these effects were limited to UV blocking. Conversely, lens power demonstrated a consistent ability to predict transmission across the UV-B, UV-A, and visible light ranges. This finding indicates the notion that optical design, rather than bulk material properties, functions as a pivotal and autonomous determinant of photoprotective capacity in soft contact lenses.

From a clinical perspective, the variability in UV-blocking efficacy among daily disposable lenses has direct implications for patient management. The statistical model confirmed that lens power significantly affects spectral transmission, with higher minus powers associated with reduced UV protection; however, lens power is inherently determined by the patient’s refractive status and cannot be modified in clinical practice. In contrast, material composition and moisture content are modifiable parameters that directly influence UV attenuation and oxygen performance. Silicone hydrogel lenses demonstrated superior Dk/t and thus remain preferable in situations requiring higher corneal oxygen supply. Nevertheless, their UV-blocking capacity is not uniform and should be considered alongside hydrogel options that incorporate UV-absorbing chromophores. Accordingly, clinicians may integrate both optical and material factors when selecting lenses, aiming for an appropriate balance between oxygen delivery and spectral protection tailored to the individual patient profile.

It is imperative to acknowledge several limitations when interpreting these findings. Firstly, the analysis was restricted to a limited range of spherical powers (+3.00 D, −3.00 D, and −6.00 D), which may not fully represent the spectral or oxygen performance across the complete prescription spectrum. Secondly, comparisons with manufacturer-provided specifications may be affected by methodological differences, hydration states, or production tolerances. Thirdly, the present measurements were executed under strictly axial illumination utilizing a spectrophotometer, which records light transmission perpendicular to the lens surface. However, in real-world conditions, the eye is exposed to light entering from multiple angles, including lateral scatter and reflections from ground or water surfaces. Because UV transmission can vary with the angle of incidence, the absence of angular or off-axis assessment may underestimate the actual exposure experienced by lens wearers. Fourthly, it should be noted that all measurements were conducted in vitro, and the lack of in vivo validation limits direct extrapolation to clinical outcomes.

Future research should therefore expand the range of prescriptions and manufacturers, incorporate dynamic and angular testing conditions, and include in vivo assessments of ocular surface UV dose and corneal physiology. In addition, angular-resolved spectrophotometry, ray-tracing simulations, and in vivo dosimetry would help elucidate how lens geometry and peripheral optical characteristics influence off-axis UV transmission under realistic lighting conditions. Finally, molecular-level analyses of chromophore distribution within the lens matrix may offer mechanistic insight into variability in UV attenuation, thereby guiding the design of next-generation contact lenses with an optimized balance of optical safety and physiological performance.

## 5. Conclusions

This study revealed substantial variability in the UV-blocking performance of daily disposable contact lenses, while all models maintained high visible light transmittance. Lens power emerged as the most consistent predictor of spectral transmission, with higher minus powers associated with reduced UV protection. Moisture content and material group also influenced UV attenuation, whereas oxygen permeability and water content showed no consistent effects. From a clinical perspective, these findings suggest that although lens power intrinsically impacts UV transmission, material composition and UV-blocking properties remain the principal modifiable factors in optimizing both physiological and photoprotective performance.

## Figures and Tables

**Table 1:** The Characteristics of The Contact Lenses in This Study.

Model	Manufacturer	Material	OP (Dk)	CT (mm)	OT (Dk/t)	MC (%)	Group
1DAM	Johnson & Johnson	Etafilcon A	28.0	0.08	33.33	58	IV
1DAO	Johnson & Johnson	Senofilcon A	103.0	0.08	121.17	38	I
M1D	Menicon	Ocufilcon D	19.7	0.07	26.26	55	IV
P1D	CooperVision	Omafilcon A	20.5	0.09	22.77	60	II
MD	CooperVision	Stenfilcon A	80.0	0.08	100	54	V
DT1	Alcon	Delefilcon A	140.0	0.09	155.55	33	V
DA	Alcon	Nelfilcon A	26.0	N/A	N/A	69	II
M1DP	Bausch + Lomb	Hilafilcon B	22.0	0.09	24.44	59	II
BOD	Bausch + Lomb	Nesofilcon A	42.0	N/A	N/A	78	II
AL1D	Bausch + Lomb	Kalifilcon A	114.0	0.07	162.85	46	V
1DPMP	SEED	SEED Ionic Bond	30.0	0.07	42.85	58	IV
1DFUP	SEED	N/A	12.0	0.08	14.28	38	I

1DAM = 1-DAY ACUVUE MOIST; 1DAO = 1-DAY ACUVUE OASYS; M1D = Menicon 1DAY; P1D = Proclear 1 Day; MD = MyDay; DT1 = DAILIES TOTAL1; DA = DAILIES Aqua; M1DP = Medalist 1Day Plus; BOD = Biotrue ONEday; AL1D = AquaLox 1Day; 1DPMP = 1day Pure Moisture Plus; 1DFUP = 1day Fine UV Plus; OP = Oxygen Permeability; CT = Central Thickness; OT = Oxygen Transmissibility; MC = Moisture Content; N/A = Not Available; Group I: Low water (<50%), non-ionic; Group II: High water (≥50%), non-ionic; Group III: Low water (<50%), ionic; Group IV: High water (≥50%), ionic; Group V: Silicone hydrogel materials (informal category, not part of original FDA I–IV classification).

**Table 2:** Transmittance Comparison of The Contact Lenses in This Study.

Model		Power (D)	Mean UV-B Transmittance (%)	Mean UV-A Transmittance (%)	Mean Visible Light Transmittance (%)	UV-Blocking Class (ISO)
1DAM	Company Provided		<3	<19	N/A	Class II
	Measured	−6.00	3.2	35.9	97.6	Non-UV Block
		−3.00	10.7	44.1	97.3	Non-UV Block
		+3.00	5.5	38.8	97.6	Non-UV Block
1DAO	Company Provided		<1	<4	N/A	Class I
	Measured	−3.00	<0.1	21.3	97.5	Class II
		+3.00	<0.1	18.2	96.3	Class II
M1D	Company Provided		N/A	N/A	N/A	N/A
	Measured	−3.00	86.7	93.5	97.4	Non-UV Block
P1D	Company Provided		N/A	N/A	N/A	N/A
	Measured	−3.00	85.7	92.9	97.1	Non-UV Block
MD	Company Provided		<5	<50	N/A	Class II
	Measured	−3.00	3.0	31.0	95.8	Non-UV Block
		+3.00	0.7	26.8	95.5	Class II
DT1	Company Provided		N/A	N/A	N/A	N/A
	Measured	−3.00	83.1	91.5	95.4	Non-UV Block
		+3.00	72.9	84.8	91.7	Non-UV Block
DA	Company Provided		N/A	N/A	N/A	N/A
	Measured	−3.00	73.2	91.7	96.4	Non-UV Block
M1DP	Company Provided		N/A	N/A	N/A	N/A
	Measured	−3.00	83.6	92.7	95.5	Non-UV Block
		+3.00	81.8	91.9	95.0	Non-UV Block
BOD	Company Provided		<5	<50	N/A	UV Block
	Measured	−3.00	1.9	29.8	95.0	Class II
		+3.00	1.3	27.9	93.0	Class II
AL1D	Company Provided		N/A	N/A	N/A	N/A
	Measured	−3.00	63.9	83.4	92.1	Non-UV Block
		+3.00	47.6	74.5	88.1	Non-UV Block
1DPMP	Company Provided		<50	<65	N/A	Non-UV Block
	Measured	−3.00	21.2	48.7	90.4	Non-UV Block
		+3.00	9.0	38.2	90.0	Non-UV Block
1DFUP	Company Provided		<5	<25	N/A	Non-UV Block
	Measured	−3.00	1.8	30.2	91.3	Non-UV Block

1DAM = 1-DAY ACUVUE MOIST; 1DAO = 1-DAY ACUVUE OASYS; M1D = Menicon 1DAY; P1D = Proclear 1 Day; MD = MyDay; DT1 = DAILIES TOTAL1; DA = DAILIES Aqua; M1DP = Medalist 1Day Plus; BOD = Biotrue ONEday; AL1D = AquaLox 1Day; 1DPMP = 1day Pure Moisture Plus; 1DFUP = 1day Fine UV Plus; UV = Ultraviolet; N/A = Not Available. UV-blocking Class (ISO), class classified by international standard organization; Class I (<1% UV-B and <10% UV-A); Class II (<5% UV-B and <30% UV-A).

**Table 3:** Summary of compliance between manufacturer-reported UV-blocking claims and measured spectral transmittance.

Spectral Region	Matched	Unmatched	Not Available
UV-B (280–315 nm)	1DAO, MD, BOD, 1DPMP, 1DFUP	1DAM	M1D, P1D, DT1, DA, M1DP, AL1D
UV-A (315–400 nm)	MD, BOD, 1DPMP	1DAM, 1DAO, 1DFUP	M1D, P1D, DT1, DA, M1DP, AL1D

1DAM = 1-DAY ACUVUE MOIST; 1DAO = 1-DAY ACUVUE OASYS; M1D = Menicon 1DAY; P1D = Proclear 1 Day; MD = MyDay; DT1 = DAILIES TOTAL1; DA = DAILIES Aqua; M1DP = Medalist 1Day Plus; BOD = Biotrue ONEday; AL1D = AquaLox 1Day; 1DPMP = 1day Pure Moisture Plus; 1DFUP = 1day Fine UV Plus; UV = Ultraviolet.

**Table 4:** Possible Parameters Associated with UV-B Transmittance.

Effect Term	Estimate	Std Error	95% CI	*p*-Value
Model	607.8	354.8	−87.5, 1303	0.08
Lens Power (D)	−0.76	0.22	−1.22, −0.29	0.002
Oxygen Permeability (Dk)	−0.11	0.31	−0.89, 0.66	0.73
Moisture Content (%)	−4.12	1.17	−6.99, −1.25	0.01
Group (I–V)	−77.6	30.19	−151.6, −3.62	0.04
Group (II–V)	96.3	38.16	2.83, 189.8	0.04
Group (IV–V)	32	34.68	−52.9, 117	0.39

Estimate, represents the effect size of each mixed factor relative to the reference group. Std Error, is the standard error of the estimate. 95% CI, denotes the 95% confidence interval. *p*-values are from generalized linear mixed model (GLMM), significance at *p* < 0.05.

**Table 5:** Possible Parameters Associated with UV-A Transmittance.

Effect Term	Estimate	Std Error	95% CI	*p*-Value
Model	378.2	220.5	−54.02, 810.5	0.08
Lens Power (D)	−0.61	0.17	−0.97, −0.26	0.001
Oxygen Permeability (Dk)	−0.14	0.25	−0.75, 0.47	0.59
Moisture Content (%)	−3.13	0.92	−5.39, −0.87	0.01
Group (I–V)	−68.6	23.8	−127, −10.3	0.02
Group (II–V)	67.9	30.09	−5.77, 141.6	0.06
Group (IV–V)	20.1	27.3	−46.8, 87.18	0.48

Estimate, represents the effect size of each mixed factor relative to the reference group. Std Error, is the standard error of the estimate. 95% CI, denotes the 95% confidence interval. *p*-values are from generalized linear mixed model (GLMM), significance at *p* < 0.05.

**Table 6:** Possible Parameters Associated with Visible Light Transmittance.

Effect Term	Estimate	Std Error	95% CI	*p*-Value
Model	10.23	6.2	−1.93, 22.4	0.09
Lens Power (D)	−0.21	0.07	−0.36, −0.06	0.005
Oxygen Permeability (Dk)	0.02	0.04	−0.07, 0.12	0.59
Moisture Content (%)	−0.007	0.15	−0.38, 0.37	0.96
Group (I–V)	1.93	3.99	−7.85, 11.7	0.64
Group (II–V)	4.38	5.05	−7.97, 16.7	0.41
Group (IV–V)	3.66	4.59	−7.57, 14.9	0.45

Estimate, represents the effect size of each mixed factor relative to the reference group. Std Error, is the standard error of the estimate. 95% CI, denotes the 95% confidence interval. *p*-values are from generalized linear mixed model (GLMM), significance at *p* < 0.05.

## Data Availability

The original contributions presented in this study are included in the article/Supplementary Material. Further inquiries can be directed to the corresponding author.

## Supplementary Materials

The following supporting information can be downloaded at: https://www.mdpi.com/article/10.3390/ma18204784/s1, Table S1: Chemical composition of contact lens materials; Table S2: Raw spectral transmittance data.

## Abbreviations

The following abbreviations are used in this manuscript:

UV	Ultraviolet
UV-B	Ultraviolet B
UV-A	Ultraviolet A
UVR	Ultraviolet Radiation
Dk	Oxygen Permeability
Dk/t	Oxygen Transmissibility
CT	Central Thickness
MC	Moisture Content
GLMM	Generalized Linear Mixed Model
FDA	Food and Drug Administration
ISO	International Organization for Standardization
N/A	Not Available
