# Supplementary material for: Spectral Transmittance of Daily Disposable Contact Lenses: Variability in Ultraviolet Blocking
Source: Materials (Basel). 2025 Oct 20;18(20):4784. doi: 10.3390/ma18204784 (PMC12566079; doi:10.3390/ma18204784)
Supplement: Supplementary file 1 [file materials-18-04784-s001.zip › materials-3896218-supplementary.docx]

Spectral Transmittance of Daily Disposable Contact Lenses: Variability in Ultraviolet Blocking

Arief Abdurrazaq Dharma ^1^, Sachiko Kaidzu ^1^, Yoshihisa Ishiba ^2, 3^, Tsutomu Okuno ^4^ and Masaki Tanito ^1 *^

^1^ Department of Ophthalmology, Shimane University Faculty of Medicine, Enya 89-1, Izumo, Shimane 693-8501, Japan; abdurrazaqacha@gmail.com (A.A.D.); kecha@med.shimane-u.ac.jp (S.K.)

^2^ Technology Development Department, Yamamoto Kogaku Co. Ltd., 3-25-8 Chodo, Higashi-Osaka, Osaka, 577-0056, Japan; ishiba.yoshihisa.ile@osaka-u.ac.jp

^3^ Institute of Laser Engineering, Osaka University, 2-6 Yamadaoka, Suita, Osaka, 565-0871, Japan

^4^ National Institute of Occupational Safety and Health, Japan, 6-21-1 Nagao, Kawasaki, Kanagawa, 214-8585, Japan; okuno.t@jcom.zaq.ne.jp

* Correspondence: mtanito@med.shimane-u.ac.jp; Tel.: +81-853-20-2284

**Table S1.** Chemical composition of contact lens materials.

| **Material** | **Type** | **Main Monomer(s)** | **Hydrophilic Co-Monomer(s)** |
| --- | --- | --- | --- |
| Etafilcon A | Hydrogel (ionic) | 2-HEMA | Methacrylic acid |
| Senofilcon A | Silicone hydrogel | TRIS, siloxane macromer | N-vinylpyrrolidone (NVP) |
| Ocufilcon D | Hydrogel (ionic) | 2-HEMA | Methacrylic acid |
| Omafilcon A | Hydrogel (non-ionic) | 2-HEMA | Phosphorylcholine (MPC) |
| Stenfilcon A | Silicone hydrogel | Silicone macromer | PEG monomer |
| Delefilcon A | Silicone hydrogel (water-gradient) | Silicone macromer | Polyvinylpyrrolidone (PVP) |
| Nelfilcon A | Hydrogel (non-ionic) | 2-HEMA | N-vinylpyrrolidone (NVP) |
| Hilafilcon B | Hydrogel (non-ionic) | 2-HEMA | N-vinylpyrrolidone (NVP) |
| Nesofilcon A | Hydrogel (non-ionic) | 2-HEMA | N-vinylpyrrolidone (NVP) + PVP |
| Kalifilcon A | Silicone hydrogel | Silicone methacrylate | NVP, DMA, 2-HEMA |
| SEED Ionic Bond | Hydrogel (ionic hybrid) | Proprietary polymer | Ionic + hydrophilic monomers |

**Table S2.** Raw spectral transmittance data.

| Wave length | 1DAM (+3) | | 1DAM (+6) | | 1DAM (-3) | | 1DAO (-3) | | 1DAO (+3) | | M1D (-3) | | P1D (-3) | | MD (-3) | | MD (+3) | | DT1 (-3) | | DT1 (+3) | | DA (-3) | | M1DP (-3) | | M1DP (+3) | | BOD (-3) | | BOD (+3) | | AL1D (-3) | | AL1D (+3) | | 1DPMP (-3) | | 1DPMP (+3) | | 1DFUP (-3) | |
| --- | --- | --- | --- | --- | --- | --- | --- | --- | --- | --- | --- | --- | --- | --- | --- | --- | --- | --- | --- | --- | --- | --- | --- | --- | --- | --- | --- | --- | --- | --- | --- | --- | --- | --- | --- | --- | --- | --- | --- | --- | --- | --- |
|  | ① | ② | ① | ② | ① | ② | ① | ② | ① | ② | ① | ② | ① | ② | ① | ② | ① | ② | ① | ② | ① | ② | ① | ② | ① | ② | ① | ② | ① | ② | ① | ② | ① | ② | ① | ② | ① | ② | ① | ② | ① | ② |
| 200 | 0.473 | 0.016 | 0.027 | 0.483 | 0.020 | 0.015 | 0.042 | 0.031 | 0.067 | 0.019 | 0.014 | 0.143 | 0.030 | 0.027 | 0.075 | 0.019 | -3.578 | 0.016 | 0.064 | 0.018 | 0.019 | 0.019 | 0.034 | 0.019 | 0.023 | 0.019 | 0.017 | 0.017 | 0.025 | 0.018 | 0.011 | 0.015 | 0.014 | 0.017 | 0.016 | 0.031 | 0.015 | 0.016 | 0.010 | 0.016 | 0.012 | 0.020 |
| 201 | 0.471 | 0.015 | 0.026 | 0.498 | 0.018 | 0.015 | 0.041 | 0.030 | 0.067 | 0.018 | 0.013 | 0.147 | 0.026 | 0.025 | 0.078 | 0.018 | -1.611 | 0.014 | 0.063 | 0.016 | 0.019 | 0.018 | 0.034 | 0.018 | 0.023 | 0.018 | 0.017 | 0.016 | 0.025 | 0.017 | 0.009 | 0.014 | 0.013 | 0.016 | 0.016 | 0.032 | 0.015 | 0.015 | 0.010 | 0.016 | 0.012 | 0.019 |
| 202 | 0.471 | 0.013 | 0.024 | 0.517 | 0.016 | 0.013 | 0.041 | 0.030 | 0.067 | 0.017 | 0.011 | 0.152 | 0.021 | 0.023 | 0.081 | 0.017 | 0.805 | 0.013 | 0.062 | 0.015 | 0.018 | 0.018 | 0.035 | 0.017 | 0.023 | 0.017 | 0.017 | 0.014 | 0.026 | 0.016 | 0.009 | 0.013 | 0.012 | 0.014 | 0.015 | 0.033 | 0.015 | 0.014 | 0.009 | 0.015 | 0.011 | 0.018 |
| 203 | 0.471 | 0.012 | 0.023 | 0.530 | 0.014 | 0.012 | 0.040 | 0.031 | 0.067 | 0.015 | 0.010 | 0.157 | 0.018 | 0.021 | 0.084 | 0.016 | 1.335 | 0.012 | 0.061 | 0.014 | 0.019 | 0.017 | 0.037 | 0.017 | 0.024 | 0.016 | 0.017 | 0.013 | 0.028 | 0.015 | 0.008 | 0.012 | 0.012 | 0.013 | 0.016 | 0.034 | 0.015 | 0.013 | 0.009 | 0.014 | 0.011 | 0.018 |
| 204 | 0.470 | 0.011 | 0.022 | 0.541 | 0.014 | 0.011 | 0.040 | 0.031 | 0.068 | 0.015 | 0.010 | 0.161 | 0.016 | 0.020 | 0.088 | 0.016 | -0.026 | 0.012 | 0.061 | 0.013 | 0.019 | 0.017 | 0.041 | 0.017 | 0.025 | 0.016 | 0.018 | 0.012 | 0.029 | 0.015 | 0.008 | 0.011 | 0.013 | 0.013 | 0.017 | 0.036 | 0.016 | 0.012 | 0.009 | 0.014 | 0.012 | 0.018 |
| 205 | 0.469 | 0.010 | 0.021 | 0.550 | 0.012 | 0.011 | 0.041 | 0.031 | 0.070 | 0.014 | 0.009 | 0.167 | 0.014 | 0.018 | 0.094 | 0.015 | -0.007 | 0.010 | 0.061 | 0.013 | 0.020 | 0.017 | 0.049 | 0.019 | 0.025 | 0.016 | 0.018 | 0.012 | 0.030 | 0.015 | 0.008 | 0.010 | 0.013 | 0.012 | 0.017 | 0.039 | 0.016 | 0.011 | 0.008 | 0.013 | 0.012 | 0.019 |
| 206 | 0.469 | 0.009 | 0.020 | 0.557 | 0.011 | 0.010 | 0.041 | 0.031 | 0.073 | 0.014 | 0.008 | 0.173 | 0.012 | 0.017 | 0.102 | 0.015 | -0.513 | 0.010 | 0.060 | 0.012 | 0.019 | 0.016 | 0.068 | 0.025 | 0.026 | 0.015 | 0.019 | 0.011 | 0.030 | 0.014 | 0.007 | 0.010 | 0.012 | 0.011 | 0.017 | 0.042 | 0.016 | 0.011 | 0.007 | 0.013 | 0.012 | 0.018 |
| 207 | 0.468 | 0.009 | 0.020 | 0.564 | 0.011 | 0.009 | 0.040 | 0.030 | 0.076 | 0.013 | 0.007 | 0.180 | 0.012 | 0.016 | 0.110 | 0.014 | -0.106 | 0.009 | 0.060 | 0.011 | 0.019 | 0.015 | 0.105 | 0.038 | 0.026 | 0.014 | 0.020 | 0.010 | 0.032 | 0.014 | 0.007 | 0.009 | 0.012 | 0.011 | 0.017 | 0.047 | 0.016 | 0.010 | 0.007 | 0.012 | 0.012 | 0.018 |
| 208 | 0.464 | 0.009 | 0.020 | 0.572 | 0.010 | 0.008 | 0.039 | 0.029 | 0.078 | 0.012 | 0.007 | 0.186 | 0.011 | 0.016 | 0.116 | 0.014 | -0.140 | 0.009 | 0.059 | 0.010 | 0.018 | 0.015 | 0.168 | 0.064 | 0.026 | 0.014 | 0.020 | 0.010 | 0.034 | 0.014 | 0.007 | 0.009 | 0.012 | 0.011 | 0.017 | 0.051 | 0.016 | 0.010 | 0.007 | 0.012 | 0.012 | 0.018 |
| 209 | 0.461 | 0.008 | 0.019 | 0.582 | 0.010 | 0.008 | 0.039 | 0.029 | 0.079 | 0.012 | 0.006 | 0.191 | 0.010 | 0.015 | 0.120 | 0.013 | 0.132 | 0.009 | 0.059 | 0.009 | 0.019 | 0.015 | 0.263 | 0.104 | 0.027 | 0.014 | 0.021 | 0.010 | 0.035 | 0.014 | 0.006 | 0.008 | 0.012 | 0.010 | 0.018 | 0.054 | 0.017 | 0.009 | 0.006 | 0.012 | 0.012 | 0.017 |
| 210 | 0.460 | 0.007 | 0.019 | 0.593 | 0.009 | 0.008 | 0.039 | 0.030 | 0.079 | 0.012 | 0.006 | 0.194 | 0.010 | 0.015 | 0.121 | 0.013 | 0.102 | 0.008 | 0.058 | 0.009 | 0.019 | 0.015 | 0.399 | 0.166 | 0.027 | 0.014 | 0.021 | 0.009 | 0.035 | 0.014 | 0.006 | 0.008 | 0.012 | 0.010 | 0.018 | 0.056 | 0.017 | 0.009 | 0.006 | 0.011 | 0.012 | 0.018 |
| 211 | 0.461 | 0.006 | 0.020 | 0.602 | 0.008 | 0.008 | 0.039 | 0.030 | 0.078 | 0.012 | 0.006 | 0.197 | 0.010 | 0.015 | 0.123 | 0.013 | 0.150 | 0.007 | 0.058 | 0.009 | 0.019 | 0.014 | 0.585 | 0.252 | 0.027 | 0.014 | 0.022 | 0.009 | 0.036 | 0.014 | 0.006 | 0.008 | 0.012 | 0.009 | 0.018 | 0.059 | 0.018 | 0.008 | 0.006 | 0.011 | 0.012 | 0.019 |
| 212 | 0.461 | 0.006 | 0.019 | 0.609 | 0.008 | 0.007 | 0.039 | 0.030 | 0.079 | 0.012 | 0.006 | 0.202 | 0.009 | 0.015 | 0.127 | 0.013 | 0.102 | 0.007 | 0.058 | 0.009 | 0.019 | 0.015 | 0.837 | 0.374 | 0.028 | 0.014 | 0.022 | 0.009 | 0.037 | 0.014 | 0.006 | 0.008 | 0.013 | 0.009 | 0.019 | 0.063 | 0.018 | 0.009 | 0.006 | 0.012 | 0.013 | 0.019 |
| 213 | 0.463 | 0.007 | 0.020 | 0.616 | 0.008 | 0.007 | 0.040 | 0.030 | 0.082 | 0.011 | 0.005 | 0.210 | 0.009 | 0.015 | 0.133 | 0.013 | -0.118 | 0.008 | 0.057 | 0.009 | 0.019 | 0.015 | 1.156 | 0.533 | 0.028 | 0.014 | 0.023 | 0.008 | 0.038 | 0.014 | 0.006 | 0.008 | 0.013 | 0.009 | 0.019 | 0.068 | 0.019 | 0.008 | 0.006 | 0.012 | 0.013 | 0.019 |
| 214 | 0.465 | 0.007 | 0.020 | 0.622 | 0.008 | 0.007 | 0.039 | 0.030 | 0.086 | 0.012 | 0.005 | 0.217 | 0.009 | 0.015 | 0.141 | 0.013 | 0.302 | 0.007 | 0.056 | 0.009 | 0.019 | 0.015 | 1.537 | 0.732 | 0.029 | 0.014 | 0.024 | 0.008 | 0.039 | 0.014 | 0.006 | 0.008 | 0.013 | 0.009 | 0.020 | 0.074 | 0.019 | 0.008 | 0.005 | 0.012 | 0.013 | 0.019 |
| 215 | 0.467 | 0.006 | 0.021 | 0.628 | 0.007 | 0.006 | 0.039 | 0.030 | 0.089 | 0.012 | 0.005 | 0.224 | 0.009 | 0.014 | 0.149 | 0.013 | 0.133 | 0.007 | 0.056 | 0.008 | 0.019 | 0.015 | 1.958 | 0.958 | 0.029 | 0.015 | 0.025 | 0.008 | 0.039 | 0.014 | 0.006 | 0.008 | 0.013 | 0.009 | 0.021 | 0.080 | 0.019 | 0.008 | 0.005 | 0.012 | 0.013 | 0.020 |
| 216 | 0.470 | 0.005 | 0.022 | 0.635 | 0.006 | 0.006 | 0.039 | 0.031 | 0.091 | 0.011 | 0.005 | 0.230 | 0.009 | 0.014 | 0.154 | 0.013 | 0.448 | 0.007 | 0.056 | 0.008 | 0.019 | 0.015 | 2.408 | 1.210 | 0.029 | 0.015 | 0.026 | 0.008 | 0.040 | 0.014 | 0.005 | 0.007 | 0.013 | 0.009 | 0.021 | 0.086 | 0.019 | 0.007 | 0.005 | 0.012 | 0.013 | 0.020 |
| 217 | 0.471 | 0.005 | 0.023 | 0.642 | 0.007 | 0.006 | 0.039 | 0.031 | 0.091 | 0.011 | 0.005 | 0.234 | 0.009 | 0.015 | 0.157 | 0.013 | 0.050 | 0.006 | 0.055 | 0.008 | 0.019 | 0.015 | 2.875 | 1.481 | 0.030 | 0.015 | 0.026 | 0.008 | 0.041 | 0.015 | 0.005 | 0.007 | 0.013 | 0.009 | 0.021 | 0.089 | 0.020 | 0.008 | 0.005 | 0.011 | 0.014 | 0.020 |
| 218 | 0.472 | 0.006 | 0.024 | 0.651 | 0.007 | 0.006 | 0.039 | 0.031 | 0.091 | 0.011 | 0.006 | 0.238 | 0.010 | 0.016 | 0.158 | 0.013 | 0.266 | 0.006 | 0.055 | 0.008 | 0.019 | 0.016 | 3.365 | 1.778 | 0.030 | 0.015 | 0.027 | 0.008 | 0.042 | 0.015 | 0.005 | 0.006 | 0.014 | 0.009 | 0.021 | 0.091 | 0.020 | 0.008 | 0.005 | 0.012 | 0.014 | 0.020 |
| 219 | 0.472 | 0.007 | 0.026 | 0.660 | 0.006 | 0.006 | 0.039 | 0.031 | 0.091 | 0.011 | 0.006 | 0.241 | 0.011 | 0.017 | 0.159 | 0.013 | 0.037 | 0.006 | 0.055 | 0.008 | 0.018 | 0.016 | 3.882 | 2.101 | 0.029 | 0.015 | 0.027 | 0.008 | 0.042 | 0.015 | 0.005 | 0.006 | 0.014 | 0.009 | 0.022 | 0.094 | 0.021 | 0.008 | 0.005 | 0.012 | 0.014 | 0.021 |
| 220 | 0.472 | 0.007 | 0.030 | 0.667 | 0.005 | 0.006 | 0.039 | 0.031 | 0.092 | 0.011 | 0.006 | 0.246 | 0.013 | 0.019 | 0.161 | 0.014 | 0.150 | 0.006 | 0.054 | 0.008 | 0.018 | 0.016 | 4.435 | 2.467 | 0.030 | 0.015 | 0.028 | 0.008 | 0.042 | 0.016 | 0.005 | 0.006 | 0.014 | 0.009 | 0.022 | 0.097 | 0.021 | 0.007 | 0.005 | 0.012 | 0.015 | 0.021 |
| 221 | 0.474 | 0.006 | 0.036 | 0.676 | 0.005 | 0.007 | 0.039 | 0.031 | 0.092 | 0.011 | 0.008 | 0.254 | 0.018 | 0.023 | 0.163 | 0.014 | 0.025 | 0.006 | 0.054 | 0.008 | 0.019 | 0.016 | 5.026 | 2.863 | 0.030 | 0.015 | 0.028 | 0.008 | 0.042 | 0.016 | 0.005 | 0.006 | 0.014 | 0.009 | 0.023 | 0.101 | 0.021 | 0.007 | 0.005 | 0.012 | 0.015 | 0.022 |
| 222 | 0.475 | 0.005 | 0.046 | 0.684 | 0.006 | 0.008 | 0.039 | 0.031 | 0.092 | 0.011 | 0.011 | 0.269 | 0.026 | 0.030 | 0.167 | 0.014 | 0.138 | 0.005 | 0.054 | 0.008 | 0.019 | 0.016 | 5.657 | 3.311 | 0.030 | 0.016 | 0.028 | 0.008 | 0.043 | 0.017 | 0.005 | 0.006 | 0.014 | 0.009 | 0.023 | 0.105 | 0.022 | 0.007 | 0.005 | 0.012 | 0.015 | 0.023 |
| 223 | 0.476 | 0.005 | 0.063 | 0.694 | 0.006 | 0.011 | 0.039 | 0.031 | 0.092 | 0.011 | 0.018 | 0.291 | 0.040 | 0.042 | 0.170 | 0.013 | 0.041 | 0.005 | 0.054 | 0.008 | 0.018 | 0.017 | 6.334 | 3.805 | 0.030 | 0.016 | 0.028 | 0.007 | 0.044 | 0.017 | 0.005 | 0.006 | 0.014 | 0.009 | 0.023 | 0.110 | 0.022 | 0.007 | 0.005 | 0.012 | 0.015 | 0.023 |
| 224 | 0.479 | 0.006 | 0.092 | 0.706 | 0.006 | 0.017 | 0.039 | 0.031 | 0.092 | 0.010 | 0.031 | 0.327 | 0.068 | 0.066 | 0.172 | 0.013 | 0.142 | 0.006 | 0.053 | 0.009 | 0.018 | 0.017 | 7.078 | 4.377 | 0.030 | 0.017 | 0.029 | 0.007 | 0.045 | 0.018 | 0.006 | 0.007 | 0.015 | 0.009 | 0.023 | 0.115 | 0.022 | 0.007 | 0.005 | 0.012 | 0.015 | 0.023 |
| 225 | 0.483 | 0.006 | 0.139 | 0.723 | 0.006 | 0.029 | 0.039 | 0.032 | 0.092 | 0.011 | 0.055 | 0.384 | 0.114 | 0.109 | 0.172 | 0.014 | 0.062 | 0.006 | 0.053 | 0.008 | 0.019 | 0.016 | 7.888 | 5.021 | 0.032 | 0.018 | 0.029 | 0.008 | 0.048 | 0.020 | 0.008 | 0.007 | 0.016 | 0.010 | 0.024 | 0.118 | 0.023 | 0.007 | 0.004 | 0.012 | 0.016 | 0.023 |
| 226 | 0.490 | 0.007 | 0.214 | 0.752 | 0.007 | 0.053 | 0.039 | 0.032 | 0.091 | 0.010 | 0.105 | 0.486 | 0.205 | 0.190 | 0.172 | 0.015 | 0.144 | 0.005 | 0.053 | 0.008 | 0.019 | 0.017 | 8.798 | 5.777 | 0.037 | 0.023 | 0.033 | 0.009 | 0.054 | 0.026 | 0.012 | 0.009 | 0.020 | 0.012 | 0.024 | 0.121 | 0.023 | 0.007 | 0.004 | 0.012 | 0.017 | 0.025 |
| 227 | 0.501 | 0.010 | 0.328 | 0.793 | 0.010 | 0.093 | 0.038 | 0.032 | 0.090 | 0.011 | 0.190 | 0.655 | 0.348 | 0.329 | 0.172 | 0.015 | 0.349 | 0.005 | 0.053 | 0.007 | 0.019 | 0.017 | 9.821 | 6.646 | 0.052 | 0.036 | 0.043 | 0.012 | 0.067 | 0.038 | 0.021 | 0.013 | 0.029 | 0.018 | 0.024 | 0.125 | 0.023 | 0.007 | 0.004 | 0.012 | 0.018 | 0.026 |
| 228 | 0.521 | 0.016 | 0.502 | 0.860 | 0.015 | 0.160 | 0.038 | 0.032 | 0.090 | 0.011 | 0.354 | 0.943 | 0.607 | 0.572 | 0.175 | 0.016 | -0.005 | 0.005 | 0.052 | 0.008 | 0.018 | 0.017 | 10.989 | 7.691 | 0.095 | 0.074 | 0.073 | 0.026 | 0.091 | 0.060 | 0.039 | 0.022 | 0.055 | 0.036 | 0.024 | 0.132 | 0.024 | 0.007 | 0.004 | 0.013 | 0.022 | 0.029 |
| 229 | 0.552 | 0.026 | 0.747 | 0.954 | 0.026 | 0.264 | 0.038 | 0.033 | 0.090 | 0.011 | 0.612 | 1.393 | 0.996 | 0.954 | 0.180 | 0.018 | 0.118 | 0.005 | 0.052 | 0.008 | 0.018 | 0.018 | 12.278 | 8.877 | 0.185 | 0.155 | 0.130 | 0.057 | 0.130 | 0.097 | 0.068 | 0.037 | 0.100 | 0.069 | 0.025 | 0.140 | 0.027 | 0.009 | 0.004 | 0.013 | 0.029 | 0.033 |
| 230 | 0.603 | 0.044 | 1.081 | 1.096 | 0.045 | 0.421 | 0.038 | 0.033 | 0.091 | 0.011 | 1.057 | 2.077 | 1.621 | 1.545 | 0.190 | 0.025 | -0.105 | 0.006 | 0.053 | 0.009 | 0.018 | 0.018 | 13.713 | 10.240 | 0.372 | 0.322 | 0.265 | 0.137 | 0.188 | 0.153 | 0.113 | 0.061 | 0.193 | 0.138 | 0.029 | 0.152 | 0.032 | 0.012 | 0.004 | 0.014 | 0.040 | 0.040 |
| 231 | 0.677 | 0.071 | 1.510 | 1.284 | 0.074 | 0.624 | 0.038 | 0.033 | 0.091 | 0.011 | 1.701 | 3.047 | 2.483 | 2.397 | 0.204 | 0.036 | 0.216 | 0.007 | 0.055 | 0.011 | 0.018 | 0.018 | 15.266 | 11.737 | 0.700 | 0.617 | 0.497 | 0.280 | 0.269 | 0.231 | 0.174 | 0.097 | 0.340 | 0.248 | 0.035 | 0.172 | 0.041 | 0.018 | 0.004 | 0.014 | 0.058 | 0.051 |
| 232 | 0.791 | 0.115 | 2.062 | 1.550 | 0.122 | 0.913 | 0.038 | 0.034 | 0.091 | 0.011 | 2.705 | 4.415 | 3.767 | 3.630 | 0.228 | 0.057 | 0.109 | 0.008 | 0.063 | 0.018 | 0.018 | 0.018 | 16.969 | 13.412 | 1.281 | 1.137 | 0.948 | 0.578 | 0.377 | 0.336 | 0.258 | 0.145 | 0.592 | 0.441 | 0.051 | 0.206 | 0.059 | 0.031 | 0.005 | 0.015 | 0.089 | 0.069 |
| 233 | 0.943 | 0.180 | 2.745 | 1.893 | 0.191 | 1.282 | 0.038 | 0.034 | 0.090 | 0.011 | 4.077 | 6.251 | 5.463 | 5.305 | 0.262 | 0.089 | 0.238 | 0.012 | 0.080 | 0.034 | 0.018 | 0.018 | 18.796 | 15.229 | 2.204 | 1.948 | 1.641 | 1.059 | 0.511 | 0.464 | 0.363 | 0.208 | 0.954 | 0.724 | 0.078 | 0.261 | 0.088 | 0.051 | 0.006 | 0.016 | 0.132 | 0.097 |
| 234 | 1.158 | 0.276 | 3.572 | 2.347 | 0.293 | 1.780 | 0.038 | 0.034 | 0.090 | 0.011 | 6.044 | 8.682 | 7.783 | 7.556 | 0.316 | 0.140 | 0.248 | 0.018 | 0.118 | 0.071 | 0.020 | 0.019 | 20.789 | 17.228 | 3.616 | 3.187 | 2.782 | 1.889 | 0.673 | 0.622 | 0.490 | 0.287 | 1.500 | 1.157 | 0.130 | 0.351 | 0.137 | 0.087 | 0.007 | 0.019 | 0.200 | 0.139 |
| 235 | 1.433 | 0.407 | 4.533 | 2.896 | 0.433 | 2.380 | 0.037 | 0.034 | 0.090 | 0.011 | 8.578 | 11.744 | 10.670 | 10.408 | 0.390 | 0.212 | 0.018 | 0.027 | 0.189 | 0.141 | 0.024 | 0.022 | 22.839 | 19.334 | 5.610 | 4.931 | 4.382 | 3.083 | 0.859 | 0.799 | 0.635 | 0.379 | 2.220 | 1.728 | 0.213 | 0.486 | 0.212 | 0.143 | 0.011 | 0.025 | 0.290 | 0.199 |
| 236 | 1.792 | 0.583 | 5.636 | 3.569 | 0.620 | 3.113 | 0.037 | 0.034 | 0.089 | 0.011 | 11.856 | 15.454 | 14.253 | 13.894 | 0.493 | 0.313 | 0.420 | 0.042 | 0.315 | 0.266 | 0.034 | 0.028 | 25.004 | 21.536 | 8.304 | 7.281 | 6.658 | 4.836 | 1.064 | 1.001 | 0.796 | 0.485 | 3.177 | 2.503 | 0.342 | 0.686 | 0.321 | 0.226 | 0.017 | 0.034 | 0.416 | 0.281 |
| 237 | 2.218 | 0.805 | 6.861 | 4.328 | 0.856 | 3.944 | 0.038 | 0.034 | 0.089 | 0.011 | 15.724 | 19.731 | 18.547 | 17.954 | 0.622 | 0.440 | 0.254 | 0.063 | 0.513 | 0.464 | 0.053 | 0.041 | 27.196 | 23.775 | 11.702 | 10.228 | 9.518 | 7.092 | 1.279 | 1.219 | 0.967 | 0.601 | 4.326 | 3.442 | 0.527 | 0.960 | 0.469 | 0.340 | 0.027 | 0.048 | 0.583 | 0.389 |
| 238 | 2.733 | 1.080 | 8.198 | 5.195 | 1.151 | 4.890 | 0.038 | 0.034 | 0.089 | 0.011 | 20.273 | 24.515 | 23.362 | 22.529 | 0.789 | 0.604 | 0.527 | 0.092 | 0.810 | 0.766 | 0.088 | 0.066 | 29.504 | 26.050 | 15.806 | 13.750 | 13.104 | 9.998 | 1.502 | 1.447 | 1.149 | 0.724 | 5.703 | 4.575 | 0.781 | 1.323 | 0.665 | 0.492 | 0.043 | 0.070 | 0.796 | 0.530 |
| 239 | 3.325 | 1.413 | 9.619 | 6.144 | 1.508 | 5.919 | 0.038 | 0.035 | 0.090 | 0.011 | 25.228 | 29.636 | 28.566 | 27.445 | 0.988 | 0.802 | 0.211 | 0.132 | 1.217 | 1.184 | 0.145 | 0.108 | 31.765 | 28.298 | 20.451 | 17.760 | 17.195 | 13.404 | 1.734 | 1.684 | 1.338 | 0.856 | 7.230 | 5.839 | 1.106 | 1.768 | 0.910 | 0.685 | 0.067 | 0.100 | 1.058 | 0.708 |
| 240 | 4.033 | 1.820 | 11.144 | 7.217 | 1.948 | 7.077 | 0.039 | 0.035 | 0.089 | 0.010 | 30.636 | 35.044 | 33.959 | 32.675 | 1.239 | 1.054 | 0.426 | 0.189 | 1.770 | 1.756 | 0.235 | 0.176 | 34.059 | 30.510 | 25.637 | 22.242 | 21.906 | 17.409 | 1.987 | 1.932 | 1.546 | 1.006 | 8.945 | 7.269 | 1.515 | 2.309 | 1.218 | 0.931 | 0.102 | 0.144 | 1.380 | 0.938 |
| 241 | 4.833 | 2.310 | 12.758 | 8.399 | 2.469 | 8.345 | 0.040 | 0.036 | 0.089 | 0.010 | 36.334 | 40.616 | 39.597 | 38.090 | 1.534 | 1.357 | 0.345 | 0.266 | 2.475 | 2.495 | 0.368 | 0.279 | 36.358 | 32.733 | 31.205 | 27.050 | 27.056 | 21.845 | 2.266 | 2.214 | 1.779 | 1.169 | 10.783 | 8.825 | 2.007 | 2.944 | 1.590 | 1.217 | 0.150 | 0.203 | 1.778 | 1.225 |
| 242 | 5.766 | 2.899 | 14.497 | 9.725 | 3.097 | 9.754 | 0.043 | 0.037 | 0.089 | 0.010 | 42.196 | 46.230 | 45.273 | 43.610 | 1.902 | 1.739 | 0.492 | 0.372 | 3.358 | 3.423 | 0.557 | 0.429 | 38.664 | 34.954 | 37.026 | 32.083 | 32.539 | 26.662 | 2.587 | 2.536 | 2.051 | 1.368 | 12.743 | 10.497 | 2.592 | 3.677 | 2.037 | 1.575 | 0.218 | 0.284 | 2.268 | 1.587 |
| 243 | 6.822 | 3.588 | 16.341 | 11.166 | 3.807 | 11.269 | 0.047 | 0.039 | 0.089 | 0.010 | 47.978 | 51.656 | 50.767 | 48.964 | 2.341 | 2.193 | 0.600 | 0.514 | 4.404 | 4.530 | 0.810 | 0.635 | 40.943 | 37.154 | 42.849 | 37.139 | 38.115 | 31.643 | 2.963 | 2.913 | 2.373 | 1.600 | 14.756 | 12.229 | 3.257 | 4.490 | 2.559 | 1.993 | 0.307 | 0.388 | 2.860 | 2.035 |
| 244 | 8.042 | 4.406 | 18.314 | 12.744 | 4.668 | 12.931 | 0.055 | 0.043 | 0.090 | 0.010 | 53.440 | 56.737 | 55.945 | 54.010 | 2.892 | 2.770 | 0.835 | 0.709 | 5.615 | 5.810 | 1.138 | 0.910 | 43.145 | 39.277 | 48.496 | 42.069 | 43.577 | 36.624 | 3.424 | 3.366 | 2.773 | 1.898 | 16.769 | 13.983 | 3.994 | 5.371 | 3.165 | 2.500 | 0.428 | 0.526 | 3.583 | 2.596 |
| 245 | 9.407 | 5.359 | 20.397 | 14.435 | 5.648 | 14.715 | 0.067 | 0.049 | 0.091 | 0.010 | 58.520 | 61.327 | 60.527 | 58.576 | 3.550 | 3.457 | 1.063 | 0.967 | 6.961 | 7.227 | 1.546 | 1.255 | 45.306 | 41.328 | 53.761 | 46.656 | 48.807 | 41.418 | 3.981 | 3.911 | 3.260 | 2.273 | 18.743 | 15.720 | 4.780 | 6.296 | 3.846 | 3.063 | 0.576 | 0.694 | 4.404 | 3.273 |
| 246 | 10.921 | 6.454 | 22.573 | 16.256 | 6.805 | 16.630 | 0.090 | 0.062 | 0.094 | 0.011 | 62.997 | 65.410 | 64.580 | 62.640 | 4.348 | 4.302 | 1.452 | 1.308 | 8.424 | 8.762 | 2.037 | 1.678 | 47.340 | 43.278 | 58.514 | 50.851 | 53.551 | 45.850 | 4.655 | 4.570 | 3.854 | 2.736 | 20.628 | 17.401 | 5.602 | 7.253 | 4.596 | 3.698 | 0.762 | 0.903 | 5.394 | 4.086 |
| 247 | 12.547 | 7.676 | 24.820 | 18.162 | 8.067 | 18.622 | 0.122 | 0.080 | 0.098 | 0.012 | 66.888 | 68.858 | 68.064 | 66.101 | 5.274 | 5.278 | 1.853 | 1.742 | 9.924 | 10.371 | 2.609 | 2.174 | 49.312 | 45.127 | 62.626 | 54.511 | 57.812 | 49.850 | 5.454 | 5.337 | 4.564 | 3.294 | 22.423 | 18.997 | 6.409 | 8.210 | 5.403 | 4.379 | 0.981 | 1.143 | 6.507 | 5.032 |
| 248 | 14.386 | 9.086 | 27.202 | 20.248 | 9.551 | 20.781 | 0.191 | 0.121 | 0.110 | 0.014 | 70.132 | 71.805 | 71.107 | 69.081 | 6.430 | 6.499 | 2.471 | 2.333 | 11.550 | 12.076 | 3.280 | 2.771 | 51.151 | 46.876 | 66.189 | 57.705 | 61.469 | 53.318 | 6.433 | 6.279 | 5.450 | 3.980 | 24.125 | 20.504 | 7.251 | 9.173 | 6.293 | 5.133 | 1.251 | 1.434 | 7.871 | 6.186 |
| 249 | 16.436 | 10.708 | 29.754 | 22.525 | 11.229 | 23.125 | 0.315 | 0.202 | 0.136 | 0.022 | 72.880 | 74.241 | 73.588 | 71.560 | 7.830 | 7.981 | 3.207 | 3.135 | 13.262 | 13.866 | 4.053 | 3.474 | 52.885 | 48.574 | 69.175 | 60.446 | 64.674 | 56.412 | 7.632 | 7.433 | 6.544 | 4.848 | 25.757 | 21.980 | 8.098 | 10.138 | 7.255 | 5.958 | 1.565 | 1.771 | 9.440 | 7.572 |
| 250 | 18.773 | 12.615 | 32.510 | 25.046 | 13.202 | 25.701 | 0.571 | 0.372 | 0.202 | 0.046 | 75.106 | 76.264 | 75.640 | 73.612 | 9.625 | 9.884 | 4.351 | 4.258 | 15.187 | 15.775 | 4.965 | 4.315 | 54.526 | 50.141 | 71.702 | 62.804 | 67.346 | 59.027 | 9.142 | 8.871 | 7.931 | 5.955 | 27.323 | 23.399 | 8.995 | 11.113 | 8.306 | 6.873 | 1.943 | 2.169 | 11.333 | 9.261 |
| 251 | 21.313 | 14.764 | 35.300 | 27.773 | 15.398 | 28.453 | 0.981 | 0.665 | 0.317 | 0.099 | 77.007 | 77.867 | 77.311 | 75.238 | 11.777 | 12.178 | 5.743 | 5.737 | 17.206 | 17.780 | 6.009 | 5.287 | 56.100 | 51.605 | 73.801 | 64.770 | 69.661 | 61.288 | 10.972 | 10.600 | 9.630 | 7.319 | 28.840 | 24.759 | 9.879 | 12.076 | 9.424 | 7.845 | 2.372 | 2.617 | 13.502 | 11.238 |
| 252 | 24.042 | 17.125 | 38.191 | 30.600 | 17.817 | 31.307 | 1.616 | 1.116 | 0.513 | 0.194 | 78.427 | 79.050 | 78.585 | 76.501 | 14.345 | 14.901 | 7.585 | 7.596 | 19.375 | 19.866 | 7.216 | 6.408 | 57.553 | 52.899 | 75.540 | 66.360 | 71.550 | 63.059 | 13.118 | 12.602 | 11.646 | 8.943 | 30.270 | 26.016 | 10.770 | 13.018 | 10.588 | 8.850 | 2.853 | 3.113 | 15.934 | 13.476 |
| 253 | 26.865 | 19.613 | 41.084 | 33.447 | 20.359 | 34.177 | 2.438 | 1.728 | 0.782 | 0.333 | 79.499 | 79.952 | 79.533 | 77.507 | 17.178 | 17.922 | 9.654 | 9.776 | 21.622 | 22.008 | 8.553 | 7.672 | 58.811 | 54.079 | 76.862 | 67.626 | 73.061 | 64.536 | 15.481 | 14.821 | 13.885 | 10.778 | 31.616 | 27.224 | 11.642 | 13.942 | 11.739 | 9.862 | 3.362 | 3.639 | 18.507 | 15.897 |
| 254 | 29.621 | 22.120 | 43.934 | 36.203 | 22.880 | 36.957 | 3.458 | 2.489 | 1.131 | 0.518 | 80.148 | 80.748 | 80.155 | 78.349 | 20.189 | 21.160 | 12.009 | 12.204 | 23.969 | 24.190 | 10.021 | 9.093 | 59.833 | 55.126 | 77.882 | 68.745 | 74.215 | 65.770 | 17.990 | 17.191 | 16.270 | 12.765 | 32.846 | 28.376 | 12.491 | 14.863 | 12.868 | 10.870 | 3.894 | 4.186 | 21.132 | 18.406 |
| 255 | 32.248 | 24.503 | 46.555 | 38.810 | 25.310 | 39.573 | 4.599 | 3.327 | 1.527 | 0.740 | 80.709 | 81.369 | 80.707 | 78.970 | 23.249 | 24.447 | 14.443 | 14.747 | 26.381 | 26.411 | 11.624 | 10.640 | 60.749 | 56.015 | 78.691 | 69.630 | 75.201 | 66.800 | 20.542 | 19.595 | 18.701 | 14.801 | 34.022 | 29.453 | 13.326 | 15.749 | 13.953 | 11.842 | 4.434 | 4.732 | 23.694 | 20.789 |
| 256 | 34.601 | 26.683 | 48.876 | 41.140 | 27.511 | 41.897 | 5.827 | 4.245 | 1.957 | 0.992 | 81.228 | 81.825 | 81.175 | 79.416 | 26.258 | 27.645 | 16.923 | 17.300 | 28.930 | 28.712 | 13.411 | 12.355 | 61.569 | 56.763 | 79.488 | 70.386 | 76.123 | 67.617 | 23.063 | 21.957 | 21.111 | 16.808 | 35.202 | 30.478 | 14.176 | 16.626 | 15.021 | 12.769 | 4.983 | 5.280 | 26.093 | 23.052 |
| 257 | 36.592 | 28.525 | 50.834 | 43.129 | 29.397 | 43.914 | 7.050 | 5.170 | 2.387 | 1.249 | 81.678 | 82.189 | 81.591 | 79.804 | 29.054 | 30.644 | 19.266 | 19.716 | 31.599 | 31.091 | 15.346 | 14.245 | 62.308 | 57.417 | 80.209 | 71.055 | 76.959 | 68.379 | 25.405 | 24.098 | 23.360 | 18.701 | 36.391 | 31.509 | 15.045 | 17.509 | 16.048 | 13.676 | 5.533 | 5.823 | 28.226 | 25.039 |
| 258 | 38.036 | 29.933 | 52.243 | 44.606 | 30.772 | 45.414 | 8.124 | 6.030 | 2.760 | 1.488 | 82.010 | 82.545 | 81.881 | 80.145 | 31.383 | 33.164 | 21.241 | 21.812 | 34.360 | 33.550 | 17.440 | 16.328 | 62.892 | 57.948 | 80.787 | 71.681 | 77.629 | 69.078 | 27.400 | 25.949 | 25.281 | 20.339 | 37.534 | 32.553 | 15.918 | 18.406 | 17.015 | 14.534 | 6.069 | 6.353 | 29.862 | 26.643 |
| 259 | 38.961 | 30.778 | 53.134 | 45.573 | 31.652 | 46.389 | 8.967 | 6.702 | 3.046 | 1.681 | 82.255 | 82.799 | 82.137 | 80.451 | 33.195 | 35.114 | 22.825 | 23.393 | 37.169 | 36.014 | 19.661 | 18.541 | 63.392 | 58.388 | 81.317 | 72.229 | 78.232 | 69.700 | 28.896 | 27.365 | 26.727 | 21.578 | 38.653 | 33.566 | 16.805 | 19.289 | 17.912 | 15.335 | 6.589 | 6.865 | 30.991 | 27.727 |
| 260 | 39.212 | 31.014 | 53.402 | 45.850 | 31.885 | 46.682 | 9.441 | 7.097 | 3.188 | 1.800 | 82.390 | 82.976 | 82.354 | 80.669 | 34.246 | 36.303 | 23.656 | 24.366 | 39.891 | 38.446 | 21.934 | 20.821 | 63.693 | 58.697 | 81.721 | 72.689 | 78.736 | 70.202 | 29.797 | 28.280 | 27.601 | 22.344 | 39.691 | 34.519 | 17.669 | 20.159 | 18.724 | 16.058 | 7.077 | 7.347 | 31.447 | 28.166 |
| 261 | 38.948 | 30.740 | 53.188 | 45.644 | 31.631 | 46.498 | 9.598 | 7.241 | 3.209 | 1.837 | 82.585 | 83.167 | 82.556 | 80.905 | 34.719 | 36.876 | 24.020 | 24.741 | 42.576 | 40.740 | 24.232 | 23.069 | 63.937 | 58.923 | 82.127 | 73.167 | 79.253 | 70.716 | 30.127 | 28.693 | 27.926 | 22.655 | 40.711 | 35.459 | 18.531 | 21.027 | 19.452 | 16.729 | 7.550 | 7.808 | 31.420 | 28.114 |
| 262 | 38.125 | 29.959 | 52.442 | 44.893 | 30.844 | 45.788 | 9.382 | 7.091 | 3.091 | 1.785 | 82.773 | 83.324 | 82.696 | 81.143 | 34.497 | 36.704 | 23.688 | 24.537 | 45.123 | 42.924 | 26.490 | 25.305 | 64.067 | 59.060 | 82.450 | 73.567 | 79.681 | 71.178 | 29.935 | 28.554 | 27.750 | 22.538 | 41.710 | 36.361 | 19.365 | 21.886 | 20.136 | 17.344 | 7.994 | 8.237 | 30.829 | 27.495 |
| 263 | 36.973 | 28.851 | 51.372 | 43.815 | 29.745 | 44.759 | 8.911 | 6.737 | 2.882 | 1.670 | 82.954 | 83.481 | 82.816 | 81.375 | 33.840 | 36.012 | 23.070 | 23.843 | 47.534 | 44.974 | 28.696 | 27.479 | 64.164 | 59.135 | 82.772 | 73.932 | 80.048 | 71.584 | 29.271 | 28.036 | 27.122 | 22.025 | 42.660 | 37.245 | 20.198 | 22.701 | 20.761 | 17.909 | 8.419 | 8.648 | 29.883 | 26.526 |
| 264 | 35.463 | 27.406 | 49.971 | 42.389 | 28.322 | 43.374 | 8.189 | 6.176 | 2.582 | 1.492 | 83.062 | 83.588 | 82.928 | 81.507 | 32.724 | 34.810 | 21.963 | 22.745 | 49.800 | 47.005 | 30.865 | 29.692 | 64.182 | 59.180 | 83.065 | 74.226 | 80.380 | 71.897 | 28.238 | 27.087 | 26.126 | 21.190 | 43.612 | 38.086 | 21.045 | 23.528 | 21.381 | 18.454 | 8.836 | 9.050 | 28.590 | 25.227 |
| 265 | 33.759 | 25.770 | 48.385 | 40.772 | 26.720 | 41.802 | 7.327 | 5.502 | 2.234 | 1.279 | 83.136 | 83.689 | 83.069 | 81.633 | 31.341 | 33.356 | 20.659 | 21.371 | 51.979 | 48.925 | 32.974 | 31.844 | 64.122 | 59.158 | 83.284 | 74.514 | 80.701 | 72.216 | 26.907 | 25.896 | 24.817 | 20.158 | 44.530 | 38.954 | 21.907 | 24.354 | 21.969 | 18.986 | 9.251 | 9.445 | 27.102 | 23.737 |
| 266 | 31.904 | 23.970 | 46.619 | 39.025 | 24.964 | 40.048 | 6.383 | 4.755 | 1.867 | 1.050 | 83.237 | 83.823 | 83.198 | 81.773 | 29.724 | 31.642 | 19.084 | 19.807 | 54.054 | 50.795 | 35.047 | 33.978 | 64.044 | 59.092 | 83.473 | 74.786 | 80.947 | 72.540 | 25.352 | 24.491 | 23.292 | 18.930 | 45.437 | 39.838 | 22.778 | 25.233 | 22.535 | 19.510 | 9.653 | 9.825 | 25.424 | 22.082 |
| 267 | 29.967 | 22.115 | 44.740 | 37.144 | 23.163 | 38.162 | 5.440 | 4.012 | 1.516 | 0.829 | 83.283 | 83.888 | 83.239 | 81.845 | 27.974 | 29.755 | 17.469 | 18.183 | 55.960 | 52.503 | 36.995 | 35.975 | 63.898 | 58.976 | 83.610 | 74.987 | 81.127 | 72.755 | 23.724 | 22.958 | 21.698 | 17.585 | 46.353 | 40.673 | 23.643 | 26.085 | 23.085 | 20.004 | 10.040 | 10.186 | 23.668 | 20.361 |
| 268 | 28.002 | 20.282 | 42.772 | 35.216 | 21.356 | 36.224 | 4.556 | 3.321 | 1.208 | 0.638 | 83.346 | 83.977 | 83.265 | 81.933 | 26.149 | 27.794 | 15.834 | 16.530 | 57.730 | 54.102 | 38.878 | 37.883 | 63.776 | 58.872 | 83.755 | 75.171 | 81.307 | 73.001 | 22.011 | 21.356 | 20.051 | 16.168 | 47.255 | 41.498 | 24.515 | 26.939 | 23.598 | 20.452 | 10.403 | 10.530 | 21.889 | 18.633 |
| 269 | 26.069 | 18.509 | 40.805 | 33.282 | 19.596 | 34.286 | 3.751 | 2.700 | 0.945 | 0.477 | 83.384 | 84.061 | 83.310 | 82.011 | 24.331 | 25.842 | 14.250 | 14.924 | 59.385 | 55.642 | 40.645 | 39.701 | 63.642 | 58.758 | 83.876 | 75.333 | 81.501 | 73.219 | 20.306 | 19.732 | 18.430 | 14.788 | 48.132 | 42.326 | 25.383 | 27.787 | 24.065 | 20.869 | 10.747 | 10.857 | 20.140 | 16.956 |
| 270 | 24.185 | 16.830 | 38.817 | 31.365 | 17.913 | 32.359 | 3.059 | 2.175 | 0.738 | 0.353 | 83.409 | 84.130 | 83.356 | 82.105 | 22.546 | 23.934 | 12.738 | 13.336 | 60.896 | 57.022 | 42.305 | 41.384 | 63.525 | 58.653 | 83.986 | 75.471 | 81.679 | 73.397 | 18.577 | 18.151 | 16.790 | 13.453 | 48.956 | 43.143 | 26.247 | 28.637 | 24.496 | 21.243 | 11.058 | 11.152 | 18.466 | 15.373 |
| 271 | 22.359 | 15.239 | 36.848 | 29.490 | 16.295 | 30.443 | 2.461 | 1.723 | 0.570 | 0.254 | 83.434 | 84.178 | 83.391 | 82.173 | 20.807 | 22.045 | 11.328 | 11.863 | 62.322 | 58.330 | 43.862 | 42.985 | 63.419 | 58.582 | 84.062 | 75.599 | 81.797 | 73.525 | 16.947 | 16.615 | 15.246 | 12.158 | 49.771 | 43.921 | 27.099 | 29.484 | 24.881 | 21.595 | 11.336 | 11.419 | 16.848 | 13.863 |
| 272 | 20.576 | 13.728 | 34.893 | 27.644 | 14.753 | 28.547 | 1.960 | 1.351 | 0.442 | 0.182 | 83.548 | 84.202 | 83.537 | 82.223 | 19.118 | 20.200 | 9.994 | 10.484 | 63.709 | 59.561 | 45.419 | 44.552 | 63.344 | 58.503 | 84.175 | 75.677 | 81.939 | 73.640 | 15.382 | 15.132 | 13.757 | 10.914 | 50.670 | 44.721 | 27.997 | 30.355 | 25.231 | 21.894 | 11.592 | 11.649 | 15.295 | 12.435 |
| 273 | 18.825 | 12.262 | 32.925 | 25.818 | 13.248 | 26.674 | 1.525 | 1.035 | 0.339 | 0.126 | 83.667 | 84.296 | 83.634 | 82.335 | 17.446 | 18.401 | 8.736 | 9.169 | 65.063 | 60.860 | 46.926 | 46.129 | 63.249 | 58.451 | 84.279 | 75.802 | 82.056 | 73.782 | 13.852 | 13.683 | 12.313 | 9.718 | 51.585 | 45.573 | 28.928 | 31.259 | 25.539 | 22.168 | 11.808 | 11.856 | 13.781 | 11.064 |
| 274 | 17.129 | 10.865 | 30.941 | 23.991 | 11.825 | 24.798 | 1.169 | 0.783 | 0.263 | 0.086 | 83.686 | 84.341 | 83.684 | 82.360 | 15.806 | 16.630 | 7.519 | 7.932 | 66.332 | 62.096 | 48.417 | 47.642 | 63.161 | 58.379 | 84.325 | 75.892 | 82.116 | 73.846 | 12.376 | 12.269 | 10.933 | 8.574 | 52.494 | 46.420 | 29.893 | 32.183 | 25.786 | 22.373 | 11.973 | 12.004 | 12.338 | 9.765 |
| 275 | 15.520 | 9.562 | 29.005 | 22.233 | 10.491 | 22.977 | 0.876 | 0.579 | 0.207 | 0.058 | 83.695 | 84.384 | 83.716 | 82.422 | 14.231 | 14.938 | 6.450 | 6.796 | 67.616 | 63.338 | 49.880 | 49.154 | 63.116 | 58.393 | 84.293 | 75.970 | 82.126 | 73.951 | 10.975 | 10.925 | 9.629 | 7.509 | 53.386 | 47.276 | 30.865 | 33.124 | 25.967 | 22.534 | 12.082 | 12.108 | 10.966 | 8.559 |
| 276 | 14.043 | 8.410 | 27.149 | 20.592 | 9.284 | 21.265 | 0.657 | 0.429 | 0.170 | 0.040 | 83.706 | 84.449 | 83.731 | 82.493 | 12.778 | 13.377 | 5.476 | 5.807 | 68.819 | 64.540 | 51.322 | 50.635 | 63.121 | 58.466 | 84.264 | 76.076 | 82.128 | 74.079 | 9.701 | 9.713 | 8.455 | 6.558 | 54.258 | 48.125 | 31.815 | 34.066 | 26.032 | 22.627 | 12.115 | 12.143 | 9.715 | 7.487 |
| 277 | 12.672 | 7.372 | 25.380 | 19.037 | 8.189 | 19.641 | 0.485 | 0.313 | 0.144 | 0.028 | 83.725 | 84.495 | 83.745 | 82.564 | 11.435 | 11.932 | 4.664 | 4.924 | 69.980 | 65.702 | 52.711 | 52.088 | 63.151 | 58.526 | 84.239 | 76.146 | 82.174 | 74.166 | 8.540 | 8.597 | 7.392 | 5.695 | 55.115 | 48.958 | 32.753 | 34.980 | 26.019 | 22.640 | 12.088 | 12.117 | 8.573 | 6.518 |
| 278 | 11.452 | 6.469 | 23.729 | 17.606 | 7.236 | 18.151 | 0.362 | 0.233 | 0.128 | 0.021 | 83.785 | 84.532 | 83.758 | 82.653 | 10.238 | 10.636 | 3.927 | 4.175 | 71.099 | 66.827 | 54.040 | 53.483 | 63.214 | 58.618 | 84.242 | 76.219 | 82.230 | 74.229 | 7.524 | 7.610 | 6.466 | 4.946 | 55.918 | 49.736 | 33.635 | 35.841 | 25.912 | 22.552 | 11.969 | 12.012 | 7.573 | 5.682 |
| 279 | 10.341 | 5.666 | 22.187 | 16.278 | 6.384 | 16.759 | 0.268 | 0.172 | 0.117 | 0.016 | 83.849 | 84.568 | 83.831 | 82.689 | 9.144 | 9.448 | 3.366 | 3.517 | 72.187 | 67.851 | 55.334 | 54.797 | 63.311 | 58.708 | 84.265 | 76.273 | 82.266 | 74.237 | 6.611 | 6.709 | 5.637 | 4.276 | 56.670 | 50.445 | 34.483 | 36.649 | 25.739 | 22.382 | 11.800 | 11.840 | 6.679 | 4.941 |
| 280 | 9.344 | 4.966 | 20.755 | 15.069 | 5.631 | 15.484 | 0.201 | 0.129 | 0.110 | 0.014 | 83.925 | 84.665 | 83.928 | 82.761 | 8.160 | 8.385 | 2.815 | 2.958 | 73.263 | 68.889 | 56.599 | 56.110 | 63.441 | 58.903 | 84.299 | 76.428 | 82.273 | 74.365 | 5.800 | 5.913 | 4.907 | 3.694 | 57.367 | 51.161 | 35.293 | 37.441 | 25.462 | 22.136 | 11.547 | 11.600 | 5.885 | 4.304 |
| 281 | 8.420 | 4.331 | 19.389 | 13.927 | 4.943 | 14.270 | 0.149 | 0.097 | 0.105 | 0.012 | 83.981 | 84.709 | 84.009 | 82.788 | 7.245 | 7.396 | 2.423 | 2.461 | 74.277 | 69.902 | 57.873 | 57.425 | 63.618 | 59.111 | 84.327 | 76.553 | 82.282 | 74.486 | 5.057 | 5.175 | 4.241 | 3.165 | 58.049 | 51.833 | 36.065 | 38.178 | 25.097 | 21.810 | 11.234 | 11.299 | 5.159 | 3.722 |
| 282 | 7.590 | 3.779 | 18.112 | 12.881 | 4.337 | 13.159 | 0.113 | 0.075 | 0.103 | 0.011 | 84.037 | 84.770 | 84.059 | 82.853 | 6.422 | 6.506 | 1.993 | 2.044 | 75.260 | 70.915 | 59.125 | 58.760 | 63.866 | 59.405 | 84.349 | 76.654 | 82.344 | 74.619 | 4.401 | 4.518 | 3.658 | 2.706 | 58.722 | 52.489 | 36.800 | 38.886 | 24.665 | 21.417 | 10.857 | 10.947 | 4.519 | 3.216 |
| 283 | 6.841 | 3.290 | 16.906 | 11.910 | 3.800 | 12.127 | 0.086 | 0.059 | 0.101 | 0.010 | 84.128 | 84.780 | 84.116 | 82.876 | 5.675 | 5.700 | 1.699 | 1.686 | 76.239 | 71.825 | 60.358 | 60.031 | 64.185 | 59.704 | 84.389 | 76.731 | 82.432 | 74.695 | 3.820 | 3.932 | 3.143 | 2.303 | 59.362 | 53.074 | 37.515 | 39.546 | 24.180 | 20.963 | 10.443 | 10.555 | 3.953 | 2.772 |
| 284 | 6.206 | 2.886 | 15.838 | 11.058 | 3.353 | 11.214 | 0.068 | 0.049 | 0.100 | 0.010 | 84.158 | 84.849 | 84.147 | 82.937 | 5.034 | 5.011 | 1.399 | 1.398 | 77.117 | 72.690 | 61.516 | 61.264 | 64.521 | 60.081 | 84.427 | 76.858 | 82.471 | 74.780 | 3.325 | 3.439 | 2.713 | 1.970 | 59.927 | 53.682 | 38.208 | 40.218 | 23.644 | 20.485 | 10.011 | 10.147 | 3.475 | 2.407 |
| 285 | 5.650 | 2.543 | 14.870 | 10.297 | 2.967 | 10.404 | 0.056 | 0.042 | 0.100 | 0.010 | 84.216 | 84.948 | 84.170 | 83.066 | 4.471 | 4.412 | 1.190 | 1.157 | 77.956 | 73.571 | 62.614 | 62.509 | 64.908 | 60.564 | 84.463 | 77.087 | 82.480 | 74.996 | 2.899 | 3.016 | 2.343 | 1.687 | 60.452 | 54.343 | 38.863 | 40.921 | 23.089 | 20.026 | 9.577 | 9.752 | 3.058 | 2.097 |
| 286 | 5.188 | 2.269 | 14.050 | 9.654 | 2.655 | 9.720 | 0.048 | 0.038 | 0.101 | 0.010 | 84.292 | 85.067 | 84.263 | 83.204 | 4.008 | 3.913 | 1.020 | 0.970 | 78.754 | 74.461 | 63.701 | 63.706 | 65.357 | 61.069 | 84.571 | 77.314 | 82.590 | 75.191 | 2.554 | 2.667 | 2.046 | 1.460 | 61.018 | 54.946 | 39.505 | 41.594 | 22.579 | 19.593 | 9.181 | 9.382 | 2.722 | 1.848 |
| 287 | 4.795 | 2.035 | 13.326 | 9.089 | 2.391 | 9.120 | 0.042 | 0.035 | 0.101 | 0.009 | 84.417 | 85.198 | 84.383 | 83.354 | 3.604 | 3.484 | 0.893 | 0.816 | 79.570 | 75.309 | 64.724 | 64.887 | 65.899 | 61.683 | 84.706 | 77.571 | 82.746 | 75.437 | 2.260 | 2.367 | 1.796 | 1.268 | 61.601 | 55.551 | 40.132 | 42.257 | 22.108 | 19.194 | 8.814 | 9.040 | 2.436 | 1.641 |
| 288 | 4.464 | 1.842 | 12.698 | 8.603 | 2.174 | 8.600 | 0.038 | 0.033 | 0.101 | 0.009 | 84.552 | 85.339 | 84.543 | 83.460 | 3.271 | 3.122 | 0.817 | 0.693 | 80.422 | 76.093 | 65.780 | 66.047 | 66.526 | 62.316 | 84.848 | 77.771 | 82.920 | 75.634 | 2.014 | 2.113 | 1.588 | 1.111 | 62.212 | 56.115 | 40.785 | 42.872 | 21.681 | 18.819 | 8.486 | 8.726 | 2.199 | 1.470 |
| 289 | 4.175 | 1.677 | 12.136 | 8.174 | 1.986 | 8.139 | 0.035 | 0.032 | 0.102 | 0.009 | 84.653 | 85.488 | 84.663 | 83.580 | 2.975 | 2.803 | 0.725 | 0.590 | 81.239 | 76.895 | 66.818 | 67.244 | 67.173 | 63.016 | 84.966 | 77.985 | 83.076 | 75.858 | 1.799 | 1.892 | 1.406 | 0.975 | 62.796 | 56.711 | 41.446 | 43.500 | 21.290 | 18.481 | 8.180 | 8.441 | 1.993 | 1.325 |
| 290 | 3.935 | 1.545 | 11.659 | 7.815 | 1.833 | 7.754 | 0.034 | 0.031 | 0.101 | 0.009 | 84.799 | 85.637 | 84.778 | 83.765 | 2.726 | 2.533 | 0.676 | 0.507 | 82.059 | 77.747 | 67.895 | 68.458 | 67.903 | 63.814 | 85.087 | 78.298 | 83.211 | 76.137 | 1.620 | 1.708 | 1.254 | 0.864 | 63.344 | 57.337 | 42.085 | 44.171 | 20.946 | 18.192 | 7.915 | 8.204 | 1.824 | 1.205 |
| 291 | 3.734 | 1.437 | 11.250 | 7.511 | 1.707 | 7.437 | 0.033 | 0.030 | 0.101 | 0.009 | 84.951 | 85.802 | 84.918 | 83.958 | 2.509 | 2.303 | 0.574 | 0.440 | 82.830 | 78.604 | 68.934 | 69.684 | 68.673 | 64.673 | 85.260 | 78.623 | 83.365 | 76.432 | 1.468 | 1.552 | 1.127 | 0.772 | 63.884 | 57.967 | 42.718 | 44.856 | 20.663 | 17.959 | 7.693 | 8.006 | 1.685 | 1.107 |
| 292 | 3.580 | 1.355 | 10.932 | 7.274 | 1.610 | 7.188 | 0.032 | 0.030 | 0.101 | 0.010 | 85.115 | 85.958 | 85.043 | 84.105 | 2.334 | 2.121 | 0.576 | 0.388 | 83.587 | 79.385 | 69.960 | 70.877 | 69.533 | 65.625 | 85.438 | 78.944 | 83.511 | 76.737 | 1.348 | 1.429 | 1.029 | 0.700 | 64.405 | 58.578 | 43.335 | 45.522 | 20.447 | 17.796 | 7.522 | 7.855 | 1.578 | 1.032 |
| 293 | 3.461 | 1.291 | 10.678 | 7.084 | 1.536 | 6.991 | 0.031 | 0.029 | 0.101 | 0.010 | 85.281 | 86.144 | 85.231 | 84.286 | 2.194 | 1.972 | 0.476 | 0.348 | 84.354 | 80.159 | 70.971 | 72.059 | 70.446 | 66.575 | 85.615 | 79.212 | 83.702 | 77.027 | 1.252 | 1.328 | 0.951 | 0.642 | 64.946 | 59.128 | 43.975 | 46.146 | 20.295 | 17.688 | 7.396 | 7.739 | 1.494 | 0.974 |
| 294 | 3.379 | 1.246 | 10.500 | 6.943 | 1.484 | 6.851 | 0.031 | 0.029 | 0.102 | 0.009 | 85.485 | 86.294 | 85.459 | 84.451 | 2.088 | 1.862 | 0.523 | 0.320 | 85.109 | 80.878 | 71.994 | 73.175 | 71.424 | 67.547 | 85.799 | 79.508 | 83.939 | 77.364 | 1.179 | 1.253 | 0.893 | 0.599 | 65.474 | 59.659 | 44.606 | 46.740 | 20.216 | 17.635 | 7.317 | 7.672 | 1.434 | 0.933 |
| 295 | 3.316 | 1.215 | 10.368 | 6.832 | 1.447 | 6.744 | 0.030 | 0.029 | 0.103 | 0.009 | 85.678 | 86.430 | 85.612 | 84.575 | 2.007 | 1.773 | 0.454 | 0.297 | 85.815 | 81.512 | 72.904 | 74.207 | 72.394 | 68.510 | 85.990 | 79.764 | 84.148 | 77.668 | 1.122 | 1.193 | 0.845 | 0.565 | 65.940 | 60.183 | 45.194 | 47.328 | 20.176 | 17.617 | 7.266 | 7.637 | 1.388 | 0.902 |
| 296 | 3.277 | 1.198 | 10.291 | 6.769 | 1.427 | 6.687 | 0.030 | 0.029 | 0.103 | 0.009 | 85.824 | 86.593 | 85.747 | 84.739 | 1.946 | 1.712 | 0.534 | 0.281 | 86.422 | 82.136 | 73.750 | 75.210 | 73.376 | 69.548 | 86.157 | 80.037 | 84.353 | 77.972 | 1.080 | 1.150 | 0.810 | 0.542 | 66.398 | 60.705 | 45.775 | 47.923 | 20.199 | 17.666 | 7.251 | 7.644 | 1.359 | 0.882 |
| 297 | 3.260 | 1.190 | 10.252 | 6.735 | 1.416 | 6.663 | 0.030 | 0.029 | 0.103 | 0.009 | 86.008 | 86.736 | 85.863 | 84.942 | 1.905 | 1.668 | 0.486 | 0.270 | 86.999 | 82.756 | 74.549 | 76.155 | 74.389 | 70.656 | 86.338 | 80.329 | 84.540 | 78.251 | 1.049 | 1.118 | 0.786 | 0.524 | 66.864 | 61.212 | 46.355 | 48.543 | 20.254 | 17.759 | 7.270 | 7.680 | 1.342 | 0.871 |
| 298 | 3.271 | 1.194 | 10.271 | 6.741 | 1.422 | 6.686 | 0.030 | 0.028 | 0.102 | 0.009 | 86.242 | 86.937 | 86.062 | 85.173 | 1.884 | 1.646 | 0.534 | 0.265 | 87.510 | 83.383 | 75.298 | 77.059 | 75.470 | 71.759 | 86.565 | 80.605 | 84.788 | 78.562 | 1.032 | 1.100 | 0.773 | 0.515 | 67.382 | 61.714 | 46.970 | 49.142 | 20.392 | 17.906 | 7.332 | 7.755 | 1.339 | 0.870 |
| 299 | 3.299 | 1.208 | 10.341 | 6.772 | 1.439 | 6.738 | 0.030 | 0.028 | 0.102 | 0.009 | 86.466 | 87.168 | 86.251 | 85.315 | 1.881 | 1.639 | 0.493 | 0.264 | 87.979 | 83.908 | 75.991 | 77.875 | 76.524 | 72.856 | 86.765 | 80.902 | 85.022 | 78.867 | 1.024 | 1.092 | 0.768 | 0.511 | 67.867 | 62.217 | 47.569 | 49.707 | 20.582 | 18.091 | 7.424 | 7.861 | 1.348 | 0.876 |
| 300 | 3.350 | 1.237 | 10.459 | 6.840 | 1.471 | 6.824 | 0.029 | 0.028 | 0.103 | 0.009 | 86.646 | 87.357 | 86.431 | 85.417 | 1.895 | 1.651 | 0.532 | 0.268 | 88.393 | 84.312 | 76.552 | 78.564 | 77.526 | 73.943 | 86.944 | 81.207 | 85.214 | 79.155 | 1.029 | 1.097 | 0.772 | 0.515 | 68.281 | 62.714 | 48.147 | 50.277 | 20.830 | 18.329 | 7.547 | 8.003 | 1.372 | 0.892 |
| 301 | 3.421 | 1.275 | 10.615 | 6.941 | 1.516 | 6.947 | 0.029 | 0.028 | 0.104 | 0.009 | 86.844 | 87.540 | 86.634 | 85.615 | 1.926 | 1.682 | 0.509 | 0.275 | 88.790 | 84.725 | 77.063 | 79.195 | 78.523 | 75.005 | 87.138 | 81.537 | 85.395 | 79.490 | 1.042 | 1.113 | 0.785 | 0.524 | 68.728 | 63.236 | 48.742 | 50.866 | 21.125 | 18.623 | 7.708 | 8.178 | 1.408 | 0.916 |
| 302 | 3.510 | 1.322 | 10.807 | 7.071 | 1.573 | 7.102 | 0.029 | 0.028 | 0.104 | 0.009 | 87.046 | 87.709 | 86.793 | 85.867 | 1.975 | 1.731 | 0.533 | 0.287 | 89.084 | 85.065 | 77.466 | 79.703 | 79.461 | 76.009 | 87.339 | 81.861 | 85.544 | 79.813 | 1.065 | 1.139 | 0.806 | 0.538 | 69.122 | 63.729 | 49.294 | 51.457 | 21.434 | 18.942 | 7.893 | 8.379 | 1.454 | 0.948 |
| 303 | 3.613 | 1.377 | 11.032 | 7.231 | 1.638 | 7.284 | 0.030 | 0.028 | 0.104 | 0.009 | 87.231 | 87.969 | 86.972 | 86.120 | 2.036 | 1.792 | 0.515 | 0.302 | 89.299 | 85.420 | 77.806 | 80.158 | 80.352 | 77.004 | 87.512 | 82.167 | 85.770 | 80.135 | 1.095 | 1.173 | 0.833 | 0.558 | 69.529 | 64.181 | 49.834 | 52.022 | 21.773 | 19.300 | 8.103 | 8.605 | 1.507 | 0.986 |
| 304 | 3.731 | 1.441 | 11.291 | 7.411 | 1.713 | 7.494 | 0.030 | 0.028 | 0.103 | 0.009 | 87.423 | 88.168 | 87.155 | 86.281 | 2.111 | 1.869 | 0.566 | 0.321 | 89.520 | 85.678 | 78.103 | 80.509 | 81.256 | 77.931 | 87.720 | 82.421 | 86.047 | 80.394 | 1.135 | 1.215 | 0.866 | 0.582 | 69.974 | 64.608 | 50.400 | 52.601 | 22.164 | 19.679 | 8.337 | 8.858 | 1.572 | 1.032 |
| 305 | 3.865 | 1.513 | 11.573 | 7.615 | 1.798 | 7.727 | 0.029 | 0.028 | 0.104 | 0.009 | 87.620 | 88.321 | 87.371 | 86.418 | 2.198 | 1.957 | 0.598 | 0.344 | 89.733 | 85.877 | 78.366 | 80.827 | 82.112 | 78.803 | 87.938 | 82.668 | 86.341 | 80.685 | 1.183 | 1.265 | 0.906 | 0.610 | 70.429 | 65.053 | 50.994 | 53.193 | 22.585 | 20.087 | 8.590 | 9.129 | 1.646 | 1.083 |
| 306 | 4.011 | 1.594 | 11.885 | 7.844 | 1.890 | 7.984 | 0.029 | 0.028 | 0.104 | 0.009 | 87.807 | 88.473 | 87.548 | 86.627 | 2.296 | 2.058 | 0.640 | 0.369 | 89.877 | 86.087 | 78.540 | 81.164 | 82.860 | 79.621 | 88.123 | 82.960 | 86.556 | 81.025 | 1.236 | 1.324 | 0.951 | 0.644 | 70.851 | 65.560 | 51.629 | 53.810 | 23.017 | 20.520 | 8.857 | 9.413 | 1.725 | 1.139 |
| 307 | 4.162 | 1.678 | 12.205 | 8.077 | 1.985 | 8.244 | 0.029 | 0.028 | 0.104 | 0.009 | 88.031 | 88.669 | 87.706 | 86.833 | 2.399 | 2.163 | 0.647 | 0.396 | 90.014 | 86.303 | 78.708 | 81.447 | 83.572 | 80.404 | 88.303 | 83.285 | 86.764 | 81.388 | 1.293 | 1.388 | 0.998 | 0.680 | 71.268 | 66.048 | 52.234 | 54.427 | 23.441 | 20.941 | 9.126 | 9.698 | 1.804 | 1.196 |
| 308 | 4.311 | 1.759 | 12.518 | 8.302 | 2.080 | 8.503 | 0.030 | 0.028 | 0.104 | 0.009 | 88.276 | 88.906 | 87.875 | 87.042 | 2.501 | 2.266 | 0.641 | 0.423 | 90.143 | 86.544 | 78.853 | 81.697 | 84.233 | 81.135 | 88.525 | 83.602 | 87.007 | 81.754 | 1.350 | 1.451 | 1.046 | 0.716 | 71.705 | 66.518 | 52.856 | 55.015 | 23.848 | 21.337 | 9.382 | 9.965 | 1.883 | 1.251 |
| 309 | 4.447 | 1.836 | 12.803 | 8.518 | 2.169 | 8.747 | 0.030 | 0.028 | 0.104 | 0.009 | 88.464 | 89.061 | 88.054 | 87.228 | 2.595 | 2.362 | 0.600 | 0.448 | 90.229 | 86.709 | 79.008 | 81.890 | 84.839 | 81.819 | 88.769 | 83.898 | 87.286 | 82.087 | 1.405 | 1.510 | 1.092 | 0.750 | 72.150 | 66.955 | 53.479 | 55.650 | 24.214 | 21.705 | 9.613 | 10.211 | 1.954 | 1.302 |
| 310 | 4.566 | 1.904 | 13.052 | 8.710 | 2.246 | 8.969 | 0.030 | 0.028 | 0.104 | 0.009 | 88.594 | 89.210 | 88.221 | 87.369 | 2.677 | 2.442 | 0.430 | 0.470 | 90.237 | 86.848 | 79.096 | 82.077 | 85.316 | 82.456 | 88.977 | 84.194 | 87.578 | 82.375 | 1.454 | 1.560 | 1.131 | 0.780 | 72.593 | 67.445 | 54.093 | 56.279 | 24.516 | 22.029 | 9.808 | 10.424 | 2.014 | 1.346 |
| 311 | 4.665 | 1.963 | 13.261 | 8.877 | 2.311 | 9.162 | 0.030 | 0.028 | 0.105 | 0.009 | 88.685 | 89.344 | 88.349 | 87.511 | 2.739 | 2.507 | 0.634 | 0.487 | 90.231 | 86.966 | 79.138 | 82.265 | 85.770 | 83.021 | 89.156 | 84.476 | 87.785 | 82.699 | 1.493 | 1.605 | 1.164 | 0.805 | 72.995 | 67.973 | 54.672 | 56.902 | 24.762 | 22.304 | 9.967 | 10.606 | 2.065 | 1.382 |
| 312 | 4.744 | 2.009 | 13.427 | 9.001 | 2.361 | 9.310 | 0.031 | 0.028 | 0.105 | 0.008 | 88.901 | 89.492 | 88.495 | 87.662 | 2.786 | 2.551 | 0.054 | 0.500 | 90.353 | 87.067 | 79.278 | 82.396 | 86.276 | 83.480 | 89.412 | 84.753 | 88.032 | 83.022 | 1.522 | 1.638 | 1.187 | 0.824 | 73.423 | 68.427 | 55.295 | 57.502 | 24.967 | 22.512 | 10.090 | 10.734 | 2.100 | 1.408 |
| 313 | 4.804 | 2.046 | 13.554 | 9.105 | 2.400 | 9.425 | 0.031 | 0.028 | 0.105 | 0.008 | 89.077 | 89.660 | 88.674 | 87.849 | 2.810 | 2.574 | 0.296 | 0.506 | 90.422 | 87.203 | 79.390 | 82.523 | 86.737 | 83.963 | 89.686 | 85.050 | 88.306 | 83.352 | 1.543 | 1.662 | 1.203 | 0.838 | 73.862 | 68.867 | 55.947 | 58.125 | 25.130 | 22.682 | 10.179 | 10.831 | 2.122 | 1.425 |
| 314 | 4.840 | 2.067 | 13.626 | 9.174 | 2.423 | 9.498 | 0.030 | 0.028 | 0.105 | 0.009 | 89.255 | 89.810 | 88.839 | 88.020 | 2.810 | 2.571 | -0.256 | 0.504 | 90.512 | 87.287 | 79.489 | 82.606 | 87.155 | 84.374 | 89.996 | 85.334 | 88.654 | 83.657 | 1.551 | 1.670 | 1.210 | 0.843 | 74.353 | 69.294 | 56.672 | 58.777 | 25.228 | 22.781 | 10.230 | 10.883 | 2.129 | 1.429 |
| 315 | 4.854 | 2.075 | 13.657 | 9.205 | 2.430 | 9.537 | 0.030 | 0.028 | 0.105 | 0.009 | 89.380 | 89.939 | 88.982 | 88.153 | 2.784 | 2.545 | 0.303 | 0.496 | 90.522 | 87.410 | 79.512 | 82.730 | 87.460 | 84.781 | 90.214 | 85.659 | 88.919 | 83.991 | 1.545 | 1.667 | 1.206 | 0.841 | 74.839 | 69.802 | 57.345 | 59.434 | 25.241 | 22.810 | 10.233 | 10.894 | 2.121 | 1.423 |
| 316 | 4.844 | 2.068 | 13.632 | 9.204 | 2.421 | 9.541 | 0.030 | 0.027 | 0.106 | 0.009 | 89.540 | 90.056 | 89.122 | 88.275 | 2.736 | 2.498 | -0.157 | 0.480 | 90.547 | 87.554 | 79.574 | 82.864 | 87.751 | 85.159 | 90.426 | 85.972 | 89.154 | 84.350 | 1.525 | 1.650 | 1.190 | 0.830 | 75.307 | 70.327 | 58.033 | 60.115 | 25.194 | 22.794 | 10.192 | 10.869 | 2.099 | 1.406 |
| 317 | 4.813 | 2.050 | 13.577 | 9.177 | 2.400 | 9.510 | 0.029 | 0.028 | 0.105 | 0.009 | 89.702 | 90.182 | 89.255 | 88.400 | 2.671 | 2.431 | -0.060 | 0.462 | 90.535 | 87.672 | 79.627 | 82.975 | 88.004 | 85.536 | 90.613 | 86.261 | 89.347 | 84.676 | 1.495 | 1.622 | 1.165 | 0.813 | 75.746 | 70.829 | 58.687 | 60.789 | 25.114 | 22.743 | 10.118 | 10.808 | 2.061 | 1.382 |
| 318 | 4.762 | 2.025 | 13.484 | 9.126 | 2.367 | 9.450 | 0.029 | 0.028 | 0.105 | 0.008 | 89.817 | 90.333 | 89.416 | 88.548 | 2.590 | 2.348 | -0.160 | 0.440 | 90.534 | 87.772 | 79.693 | 83.063 | 88.252 | 85.888 | 90.860 | 86.557 | 89.588 | 85.017 | 1.459 | 1.585 | 1.133 | 0.791 | 76.194 | 71.314 | 59.342 | 61.464 | 25.006 | 22.661 | 10.024 | 10.730 | 2.017 | 1.351 |
| 319 | 4.702 | 1.991 | 13.367 | 9.054 | 2.326 | 9.363 | 0.029 | 0.027 | 0.105 | 0.008 | 89.926 | 90.459 | 89.535 | 88.713 | 2.501 | 2.255 | -0.242 | 0.417 | 90.576 | 87.862 | 79.723 | 83.147 | 88.470 | 86.184 | 91.104 | 86.845 | 89.841 | 85.333 | 1.418 | 1.541 | 1.098 | 0.765 | 76.640 | 71.813 | 60.014 | 62.118 | 24.866 | 22.551 | 9.913 | 10.621 | 1.967 | 1.316 |
| 320 | 4.631 | 1.951 | 13.234 | 8.964 | 2.277 | 9.258 | 0.029 | 0.027 | 0.106 | 0.009 | 90.046 | 90.561 | 89.633 | 88.873 | 2.404 | 2.154 | -0.085 | 0.389 | 90.580 | 87.938 | 79.760 | 83.258 | 88.661 | 86.451 | 91.312 | 87.141 | 90.078 | 85.654 | 1.371 | 1.491 | 1.058 | 0.736 | 77.098 | 72.316 | 60.700 | 62.786 | 24.696 | 22.412 | 9.785 | 10.501 | 1.911 | 1.276 |
| 321 | 4.555 | 1.908 | 13.083 | 8.852 | 2.224 | 9.138 | 0.028 | 0.026 | 0.106 | 0.009 | 90.218 | 90.663 | 89.760 | 89.012 | 2.302 | 2.048 | -0.301 | 0.361 | 90.605 | 87.970 | 79.829 | 83.332 | 88.907 | 86.680 | 91.546 | 87.362 | 90.337 | 85.930 | 1.319 | 1.435 | 1.014 | 0.704 | 77.570 | 72.803 | 61.433 | 63.448 | 24.529 | 22.245 | 9.644 | 10.367 | 1.853 | 1.234 |
| 322 | 4.471 | 1.861 | 12.918 | 8.733 | 2.169 | 9.010 | 0.027 | 0.026 | 0.106 | 0.008 | 90.403 | 90.791 | 89.924 | 89.145 | 2.194 | 1.938 | 0.044 | 0.334 | 90.621 | 88.016 | 79.889 | 83.427 | 89.088 | 86.895 | 91.723 | 87.615 | 90.550 | 86.198 | 1.264 | 1.377 | 0.969 | 0.671 | 78.024 | 73.298 | 62.099 | 64.118 | 24.325 | 22.074 | 9.496 | 10.224 | 1.792 | 1.190 |
| 323 | 4.386 | 1.814 | 12.749 | 8.614 | 2.114 | 8.880 | 0.027 | 0.026 | 0.105 | 0.008 | 90.553 | 90.938 | 90.076 | 89.294 | 2.088 | 1.829 | -0.326 | 0.307 | 90.662 | 88.103 | 79.959 | 83.494 | 89.241 | 87.099 | 91.951 | 87.882 | 90.751 | 86.481 | 1.211 | 1.319 | 0.925 | 0.639 | 78.468 | 73.765 | 62.792 | 64.763 | 24.114 | 21.910 | 9.349 | 10.082 | 1.732 | 1.148 |
| 324 | 4.311 | 1.770 | 12.596 | 8.508 | 2.063 | 8.756 | 0.027 | 0.026 | 0.105 | 0.008 | 90.650 | 91.096 | 90.202 | 89.463 | 1.988 | 1.727 | 0.156 | 0.283 | 90.648 | 88.251 | 79.970 | 83.594 | 89.330 | 87.323 | 92.121 | 88.135 | 90.901 | 86.791 | 1.160 | 1.265 | 0.883 | 0.608 | 78.861 | 74.230 | 63.460 | 65.408 | 23.900 | 21.748 | 9.208 | 9.946 | 1.675 | 1.109 |
| 325 | 4.242 | 1.730 | 12.454 | 8.406 | 2.017 | 8.648 | 0.027 | 0.026 | 0.105 | 0.008 | 90.770 | 91.230 | 90.339 | 89.628 | 1.897 | 1.632 | -0.211 | 0.261 | 90.652 | 88.345 | 80.033 | 83.703 | 89.473 | 87.521 | 92.330 | 88.366 | 91.176 | 87.075 | 1.111 | 1.214 | 0.843 | 0.579 | 79.317 | 74.685 | 64.162 | 66.065 | 23.725 | 21.577 | 9.083 | 9.821 | 1.623 | 1.074 |
| 326 | 4.180 | 1.695 | 12.327 | 8.312 | 1.976 | 8.556 | 0.027 | 0.026 | 0.105 | 0.008 | 90.899 | 91.382 | 90.503 | 89.789 | 1.816 | 1.548 | 0.338 | 0.242 | 90.693 | 88.397 | 80.086 | 83.824 | 89.622 | 87.711 | 92.488 | 88.586 | 91.402 | 87.329 | 1.069 | 1.167 | 0.808 | 0.554 | 79.749 | 75.153 | 64.791 | 66.725 | 23.575 | 21.440 | 8.968 | 9.717 | 1.577 | 1.043 |
| 327 | 4.121 | 1.664 | 12.211 | 8.228 | 1.940 | 8.475 | 0.027 | 0.025 | 0.106 | 0.008 | 91.091 | 91.530 | 90.654 | 89.943 | 1.740 | 1.472 | -0.043 | 0.225 | 90.778 | 88.454 | 80.147 | 83.926 | 89.735 | 87.886 | 92.635 | 88.816 | 91.563 | 87.598 | 1.032 | 1.126 | 0.776 | 0.530 | 80.144 | 75.574 | 65.367 | 67.332 | 23.445 | 21.332 | 8.868 | 9.626 | 1.536 | 1.015 |
| 328 | 4.072 | 1.641 | 12.111 | 8.155 | 1.908 | 8.395 | 0.026 | 0.025 | 0.106 | 0.008 | 91.216 | 91.644 | 90.828 | 90.068 | 1.672 | 1.402 | 0.466 | 0.209 | 90.817 | 88.499 | 80.214 | 83.971 | 89.876 | 88.057 | 92.793 | 89.026 | 91.664 | 87.830 | 0.997 | 1.089 | 0.748 | 0.510 | 80.494 | 75.950 | 65.934 | 67.900 | 23.336 | 21.241 | 8.782 | 9.544 | 1.501 | 0.991 |
| 329 | 4.037 | 1.621 | 12.034 | 8.096 | 1.884 | 8.330 | 0.026 | 0.025 | 0.106 | 0.008 | 91.353 | 91.725 | 91.009 | 90.171 | 1.612 | 1.338 | 0.115 | 0.195 | 90.838 | 88.560 | 80.322 | 84.046 | 90.016 | 88.199 | 92.964 | 89.188 | 91.833 | 87.988 | 0.965 | 1.055 | 0.724 | 0.491 | 80.869 | 76.312 | 66.528 | 68.446 | 23.254 | 21.159 | 8.714 | 9.472 | 1.474 | 0.970 |
| 330 | 4.015 | 1.608 | 11.991 | 8.059 | 1.868 | 8.288 | 0.026 | 0.025 | 0.105 | 0.008 | 91.484 | 91.792 | 91.117 | 90.246 | 1.559 | 1.281 | 0.580 | 0.184 | 90.850 | 88.614 | 80.463 | 84.122 | 90.177 | 88.304 | 93.135 | 89.340 | 92.060 | 88.103 | 0.939 | 1.027 | 0.703 | 0.476 | 81.258 | 76.665 | 67.127 | 68.979 | 23.204 | 21.103 | 8.672 | 9.431 | 1.452 | 0.954 |
| 331 | 4.007 | 1.603 | 11.979 | 8.046 | 1.860 | 8.280 | 0.026 | 0.025 | 0.105 | 0.008 | 91.599 | 91.946 | 91.203 | 90.443 | 1.512 | 1.234 | 0.269 | 0.175 | 90.857 | 88.763 | 80.521 | 84.292 | 90.285 | 88.489 | 93.252 | 89.539 | 92.239 | 88.318 | 0.919 | 1.005 | 0.687 | 0.465 | 81.630 | 77.081 | 67.653 | 69.539 | 23.181 | 21.111 | 8.650 | 9.424 | 1.436 | 0.944 |
| 332 | 4.012 | 1.605 | 11.993 | 8.050 | 1.862 | 8.290 | 0.025 | 0.025 | 0.106 | 0.008 | 91.724 | 92.077 | 91.278 | 90.632 | 1.475 | 1.197 | 0.659 | 0.167 | 90.865 | 88.869 | 80.540 | 84.410 | 90.368 | 88.629 | 93.354 | 89.703 | 92.369 | 88.531 | 0.905 | 0.988 | 0.676 | 0.456 | 81.976 | 77.460 | 68.167 | 70.049 | 23.204 | 21.139 | 8.652 | 9.432 | 1.429 | 0.941 |
| 333 | 4.034 | 1.614 | 12.025 | 8.073 | 1.874 | 8.321 | 0.025 | 0.025 | 0.106 | 0.008 | 91.849 | 92.166 | 91.399 | 90.765 | 1.446 | 1.167 | 0.392 | 0.161 | 90.894 | 88.932 | 80.587 | 84.505 | 90.426 | 88.783 | 93.437 | 89.849 | 92.453 | 88.745 | 0.895 | 0.977 | 0.668 | 0.451 | 82.297 | 77.855 | 68.657 | 70.534 | 23.242 | 21.185 | 8.676 | 9.464 | 1.429 | 0.943 |
| 334 | 4.068 | 1.633 | 12.090 | 8.121 | 1.895 | 8.380 | 0.025 | 0.025 | 0.106 | 0.008 | 91.984 | 92.239 | 91.504 | 90.867 | 1.426 | 1.144 | 0.727 | 0.157 | 90.930 | 88.984 | 80.698 | 84.639 | 90.508 | 88.949 | 93.530 | 90.004 | 92.546 | 88.903 | 0.891 | 0.974 | 0.665 | 0.450 | 82.503 | 78.190 | 69.173 | 71.006 | 23.318 | 21.267 | 8.726 | 9.518 | 1.438 | 0.949 |
| 335 | 4.119 | 1.661 | 12.193 | 8.199 | 1.927 | 8.472 | 0.025 | 0.025 | 0.106 | 0.008 | 92.092 | 92.364 | 91.627 | 90.975 | 1.412 | 1.130 | 0.570 | 0.154 | 90.974 | 89.113 | 80.833 | 84.809 | 90.657 | 89.138 | 93.623 | 90.211 | 92.645 | 89.069 | 0.893 | 0.977 | 0.666 | 0.452 | 82.706 | 78.514 | 69.629 | 71.503 | 23.427 | 21.400 | 8.804 | 9.607 | 1.457 | 0.962 |
| 336 | 4.188 | 1.701 | 12.348 | 8.298 | 1.971 | 8.595 | 0.025 | 0.024 | 0.107 | 0.008 | 92.157 | 92.448 | 91.705 | 91.071 | 1.404 | 1.123 | 0.639 | 0.153 | 90.993 | 89.227 | 80.873 | 84.918 | 90.734 | 89.241 | 93.660 | 90.323 | 92.718 | 89.217 | 0.900 | 0.985 | 0.672 | 0.456 | 82.981 | 78.797 | 70.039 | 71.922 | 23.591 | 21.582 | 8.912 | 9.728 | 1.486 | 0.982 |
| 337 | 4.280 | 1.752 | 12.533 | 8.421 | 2.026 | 8.739 | 0.025 | 0.024 | 0.107 | 0.008 | 92.236 | 92.532 | 91.797 | 91.120 | 1.405 | 1.121 | 0.912 | 0.153 | 91.021 | 89.263 | 80.910 | 84.958 | 90.801 | 89.313 | 93.718 | 90.424 | 92.796 | 89.341 | 0.913 | 0.998 | 0.684 | 0.464 | 83.320 | 79.067 | 70.447 | 72.302 | 23.796 | 21.792 | 9.052 | 9.876 | 1.523 | 1.008 |
| 338 | 4.406 | 1.817 | 12.764 | 8.592 | 2.101 | 8.944 | 0.025 | 0.024 | 0.108 | 0.008 | 92.371 | 92.605 | 91.913 | 91.204 | 1.415 | 1.127 | 0.323 | 0.155 | 91.072 | 89.299 | 80.982 | 85.018 | 90.879 | 89.455 | 93.804 | 90.528 | 92.901 | 89.477 | 0.932 | 1.019 | 0.702 | 0.477 | 83.648 | 79.367 | 70.873 | 72.691 | 24.063 | 22.065 | 9.245 | 10.078 | 1.574 | 1.042 |
| 339 | 4.551 | 1.908 | 13.077 | 8.828 | 2.212 | 9.212 | 0.026 | 0.026 | 0.102 | 0.009 | 92.412 | 92.745 | 92.061 | 91.397 | 1.436 | 1.159 | 0.027 | 0.161 | 91.125 | 89.418 | 81.096 | 85.129 | 91.006 | 89.652 | 93.893 | 90.707 | 93.046 | 89.622 | 0.974 | 1.064 | 0.730 | 0.499 | 83.883 | 79.667 | 71.269 | 73.079 | 24.447 | 22.459 | 9.466 | 10.322 | 1.657 | 1.108 |
| 340 | 4.732 | 2.028 | 13.492 | 9.134 | 2.364 | 9.549 | 0.030 | 0.030 | 0.092 | 0.011 | 92.393 | 92.789 | 92.200 | 91.536 | 1.471 | 1.214 | -0.644 | 0.174 | 91.178 | 89.493 | 81.228 | 85.211 | 91.198 | 89.737 | 93.970 | 90.795 | 93.236 | 89.709 | 1.038 | 1.132 | 0.770 | 0.528 | 84.194 | 79.920 | 71.598 | 73.389 | 24.946 | 22.955 | 9.739 | 10.609 | 1.777 | 1.205 |
| 341 | 4.937 | 2.167 | 13.945 | 9.469 | 2.531 | 9.925 | 0.033 | 0.032 | 0.085 | 0.013 | 92.341 | 92.796 | 92.278 | 91.619 | 1.521 | 1.280 | 0.262 | 0.189 | 91.147 | 89.557 | 81.290 | 85.290 | 91.280 | 89.786 | 93.974 | 90.828 | 93.342 | 89.817 | 1.109 | 1.209 | 0.819 | 0.564 | 84.417 | 80.185 | 71.892 | 73.694 | 25.466 | 23.494 | 10.051 | 10.938 | 1.911 | 1.311 |
| 342 | 5.189 | 2.320 | 14.425 | 9.831 | 2.705 | 10.334 | 0.036 | 0.034 | 0.084 | 0.014 | 92.366 | 92.871 | 92.335 | 91.724 | 1.587 | 1.348 | 0.360 | 0.205 | 91.146 | 89.649 | 81.330 | 85.381 | 91.312 | 89.908 | 93.982 | 90.907 | 93.385 | 89.933 | 1.182 | 1.289 | 0.876 | 0.605 | 84.625 | 80.406 | 72.159 | 74.022 | 25.993 | 24.023 | 10.423 | 11.321 | 2.044 | 1.413 |
| 343 | 5.476 | 2.484 | 14.930 | 10.222 | 2.889 | 10.779 | 0.036 | 0.035 | 0.089 | 0.014 | 92.495 | 92.989 | 92.389 | 91.846 | 1.665 | 1.419 | 0.762 | 0.222 | 91.224 | 89.779 | 81.362 | 85.474 | 91.354 | 90.080 | 94.015 | 91.051 | 93.384 | 90.096 | 1.255 | 1.369 | 0.939 | 0.652 | 84.796 | 80.600 | 72.440 | 74.360 | 26.526 | 24.556 | 10.839 | 11.757 | 2.184 | 1.512 |
| 344 | 5.811 | 2.683 | 15.537 | 10.681 | 3.123 | 11.295 | 0.038 | 0.037 | 0.088 | 0.016 | 92.531 | 93.150 | 92.454 | 91.955 | 1.761 | 1.518 | 0.502 | 0.246 | 91.242 | 89.879 | 81.399 | 85.559 | 91.434 | 90.259 | 94.062 | 91.200 | 93.396 | 90.222 | 1.353 | 1.475 | 1.018 | 0.713 | 84.944 | 80.782 | 72.687 | 74.640 | 27.154 | 25.193 | 11.299 | 12.253 | 2.360 | 1.646 |
| 345 | 6.192 | 2.912 | 16.213 | 11.184 | 3.389 | 11.866 | 0.040 | 0.039 | 0.090 | 0.018 | 92.601 | 93.179 | 92.584 | 91.957 | 1.881 | 1.635 | 0.463 | 0.274 | 91.265 | 89.866 | 81.489 | 85.587 | 91.599 | 90.313 | 94.162 | 91.266 | 93.499 | 90.287 | 1.469 | 1.597 | 1.113 | 0.786 | 85.125 | 80.976 | 73.029 | 74.863 | 27.880 | 25.886 | 11.831 | 12.798 | 2.567 | 1.800 |
| 346 | 6.637 | 3.190 | 16.977 | 11.770 | 3.706 | 12.533 | 0.042 | 0.040 | 0.091 | 0.019 | 92.668 | 93.200 | 92.667 | 91.988 | 2.030 | 1.783 | 0.450 | 0.311 | 91.268 | 89.905 | 81.546 | 85.663 | 91.677 | 90.365 | 94.192 | 91.339 | 93.594 | 90.338 | 1.613 | 1.749 | 1.231 | 0.878 | 85.288 | 81.189 | 73.292 | 75.121 | 28.680 | 26.683 | 12.444 | 13.438 | 2.817 | 1.988 |
| 347 | 7.142 | 3.506 | 17.811 | 12.432 | 4.065 | 13.268 | 0.044 | 0.042 | 0.093 | 0.020 | 92.737 | 93.245 | 92.711 | 92.083 | 2.204 | 1.956 | 0.397 | 0.355 | 91.334 | 89.985 | 81.611 | 85.778 | 91.728 | 90.414 | 94.201 | 91.418 | 93.669 | 90.406 | 1.780 | 1.928 | 1.370 | 0.983 | 85.483 | 81.325 | 73.536 | 75.364 | 29.546 | 27.526 | 13.121 | 14.135 | 3.101 | 2.202 |
| 348 | 7.711 | 3.873 | 18.716 | 13.177 | 4.477 | 14.095 | 0.046 | 0.044 | 0.095 | 0.022 | 92.809 | 93.338 | 92.762 | 92.192 | 2.409 | 2.162 | 0.462 | 0.411 | 91.370 | 90.087 | 81.616 | 85.888 | 91.767 | 90.490 | 94.223 | 91.501 | 93.700 | 90.520 | 1.975 | 2.137 | 1.534 | 1.109 | 85.610 | 81.503 | 73.741 | 75.597 | 30.454 | 28.437 | 13.852 | 14.894 | 3.428 | 2.452 |
| 349 | 8.332 | 4.278 | 19.685 | 13.966 | 4.933 | 14.976 | 0.049 | 0.046 | 0.096 | 0.024 | 92.858 | 93.428 | 92.813 | 92.278 | 2.639 | 2.393 | 0.474 | 0.477 | 91.344 | 90.146 | 81.646 | 85.951 | 91.806 | 90.602 | 94.312 | 91.573 | 93.715 | 90.648 | 2.194 | 2.370 | 1.719 | 1.258 | 85.728 | 81.667 | 73.951 | 75.848 | 31.409 | 29.397 | 14.633 | 15.692 | 3.789 | 2.734 |
| 350 | 9.016 | 4.733 | 20.727 | 14.818 | 5.443 | 15.916 | 0.052 | 0.048 | 0.098 | 0.025 | 92.900 | 93.471 | 92.900 | 92.304 | 2.905 | 2.660 | 0.503 | 0.554 | 91.369 | 90.168 | 81.714 | 85.950 | 91.909 | 90.698 | 94.418 | 91.635 | 93.777 | 90.758 | 2.447 | 2.636 | 1.933 | 1.422 | 85.848 | 81.827 | 74.176 | 76.088 | 32.425 | 30.381 | 15.485 | 16.562 | 4.201 | 3.057 |
| 351 | 9.762 | 5.230 | 21.842 | 15.732 | 6.006 | 16.908 | 0.055 | 0.050 | 0.100 | 0.027 | 92.923 | 93.496 | 92.926 | 92.331 | 3.200 | 2.954 | 0.594 | 0.643 | 91.380 | 90.195 | 81.748 | 85.951 | 91.930 | 90.777 | 94.419 | 91.706 | 93.805 | 90.793 | 2.729 | 2.934 | 2.172 | 1.607 | 85.955 | 81.952 | 74.345 | 76.316 | 33.470 | 31.415 | 16.379 | 17.489 | 4.657 | 3.415 |
| 352 | 10.609 | 5.809 | 23.051 | 16.759 | 6.656 | 18.010 | 0.059 | 0.053 | 0.102 | 0.029 | 93.005 | 93.537 | 93.018 | 92.412 | 3.547 | 3.305 | 0.630 | 0.754 | 91.482 | 90.263 | 81.773 | 86.005 | 91.992 | 90.884 | 94.435 | 91.817 | 93.844 | 90.863 | 3.061 | 3.285 | 2.456 | 1.824 | 86.113 | 82.098 | 74.577 | 76.506 | 34.603 | 32.520 | 17.369 | 18.508 | 5.185 | 3.834 |
| 353 | 11.553 | 6.475 | 24.372 | 17.887 | 7.390 | 19.228 | 0.064 | 0.057 | 0.104 | 0.031 | 93.119 | 93.639 | 93.086 | 92.541 | 3.945 | 3.709 | 0.750 | 0.889 | 91.539 | 90.350 | 81.804 | 86.142 | 92.062 | 91.043 | 94.450 | 91.909 | 93.891 | 90.954 | 3.443 | 3.686 | 2.783 | 2.090 | 86.246 | 82.306 | 74.791 | 76.716 | 35.815 | 33.732 | 18.452 | 19.616 | 5.791 | 4.321 |
| 354 | 12.639 | 7.260 | 25.832 | 19.167 | 8.252 | 20.602 | 0.070 | 0.062 | 0.107 | 0.034 | 93.169 | 93.737 | 93.162 | 92.635 | 4.418 | 4.194 | 0.853 | 1.062 | 91.566 | 90.395 | 81.891 | 86.282 | 92.143 | 91.131 | 94.502 | 91.958 | 93.929 | 91.052 | 3.899 | 4.163 | 3.179 | 2.406 | 86.350 | 82.436 | 74.986 | 76.925 | 37.113 | 35.034 | 19.673 | 20.843 | 6.510 | 4.908 |
| 355 | 13.845 | 8.144 | 27.411 | 20.561 | 9.228 | 22.081 | 0.077 | 0.066 | 0.110 | 0.036 | 93.183 | 93.774 | 93.239 | 92.693 | 4.956 | 4.744 | 1.054 | 1.267 | 91.556 | 90.410 | 81.926 | 86.329 | 92.235 | 91.145 | 94.534 | 91.966 | 93.995 | 91.115 | 4.418 | 4.706 | 3.635 | 2.781 | 86.438 | 82.556 | 75.150 | 77.077 | 38.454 | 36.400 | 20.970 | 22.163 | 7.317 | 5.578 |
| 356 | 15.186 | 9.159 | 29.084 | 22.066 | 10.328 | 23.684 | 0.088 | 0.073 | 0.113 | 0.039 | 93.256 | 93.793 | 93.319 | 92.753 | 5.585 | 5.383 | 1.307 | 1.522 | 91.568 | 90.448 | 81.959 | 86.334 | 92.284 | 91.182 | 94.534 | 91.992 | 94.044 | 91.129 | 5.019 | 5.334 | 4.172 | 3.212 | 86.570 | 82.689 | 75.309 | 77.224 | 39.903 | 37.845 | 22.384 | 23.586 | 8.251 | 6.361 |
| 357 | 16.655 | 10.280 | 30.859 | 23.671 | 11.549 | 25.382 | 0.102 | 0.081 | 0.116 | 0.041 | 93.376 | 93.852 | 93.397 | 92.781 | 6.292 | 6.104 | 1.586 | 1.825 | 91.632 | 90.527 | 81.989 | 86.350 | 92.340 | 91.284 | 94.535 | 92.033 | 94.095 | 91.147 | 5.716 | 6.038 | 4.776 | 3.719 | 86.658 | 82.815 | 75.465 | 77.365 | 41.376 | 39.303 | 23.866 | 25.095 | 9.280 | 7.234 |
| 358 | 18.251 | 11.550 | 32.732 | 25.408 | 12.908 | 27.208 | 0.122 | 0.094 | 0.121 | 0.044 | 93.494 | 93.945 | 93.441 | 92.830 | 7.109 | 6.940 | 2.018 | 2.203 | 91.674 | 90.604 | 82.097 | 86.431 | 92.426 | 91.450 | 94.586 | 92.084 | 94.147 | 91.198 | 6.477 | 6.845 | 5.473 | 4.299 | 86.789 | 82.915 | 75.644 | 77.526 | 42.918 | 40.848 | 25.454 | 26.708 | 10.456 | 8.240 |
| 359 | 19.973 | 12.944 | 34.702 | 27.283 | 14.398 | 29.161 | 0.148 | 0.109 | 0.126 | 0.047 | 93.527 | 94.067 | 93.495 | 92.943 | 8.024 | 7.887 | 2.457 | 2.651 | 91.725 | 90.744 | 82.171 | 86.567 | 92.511 | 91.610 | 94.614 | 92.236 | 94.169 | 91.343 | 7.351 | 7.750 | 6.284 | 4.981 | 86.843 | 83.042 | 75.761 | 77.697 | 44.439 | 42.429 | 27.097 | 28.379 | 11.734 | 9.360 |
| 360 | 21.883 | 14.550 | 36.832 | 29.331 | 16.102 | 31.257 | 0.190 | 0.134 | 0.133 | 0.051 | 93.606 | 94.125 | 93.564 | 93.038 | 9.123 | 9.013 | 3.085 | 3.218 | 91.726 | 90.827 | 82.232 | 86.630 | 92.564 | 91.705 | 94.637 | 92.308 | 94.181 | 91.425 | 8.356 | 8.814 | 7.207 | 5.781 | 86.952 | 83.133 | 75.913 | 77.869 | 46.097 | 44.128 | 28.892 | 30.183 | 13.213 | 10.673 |
| 361 | 24.005 | 16.360 | 39.130 | 31.542 | 18.015 | 33.590 | 0.249 | 0.171 | 0.144 | 0.055 | 93.688 | 94.211 | 93.636 | 93.119 | 10.405 | 10.335 | 3.793 | 3.947 | 91.775 | 90.869 | 82.288 | 86.695 | 92.656 | 91.765 | 94.670 | 92.354 | 94.186 | 91.454 | 9.578 | 10.052 | 8.315 | 6.751 | 87.059 | 83.243 | 76.065 | 78.039 | 47.837 | 45.905 | 30.842 | 32.121 | 14.889 | 12.175 |
| 362 | 26.419 | 18.457 | 41.642 | 33.989 | 20.227 | 36.035 | 0.347 | 0.233 | 0.162 | 0.062 | 93.752 | 94.260 | 93.719 | 93.183 | 11.955 | 11.932 | 4.711 | 4.831 | 91.800 | 90.921 | 82.330 | 86.795 | 92.719 | 91.803 | 94.719 | 92.409 | 94.212 | 91.488 | 11.015 | 11.545 | 9.618 | 7.911 | 87.116 | 83.354 | 76.207 | 78.182 | 49.644 | 47.755 | 32.957 | 34.221 | 16.824 | 13.947 |
| 363 | 29.022 | 20.783 | 44.264 | 36.594 | 22.633 | 38.632 | 0.488 | 0.323 | 0.190 | 0.071 | 93.789 | 94.274 | 93.799 | 93.216 | 13.723 | 13.758 | 5.866 | 5.935 | 91.848 | 90.945 | 82.426 | 86.916 | 92.785 | 91.870 | 94.731 | 92.413 | 94.239 | 91.505 | 12.699 | 13.231 | 11.196 | 9.294 | 87.211 | 83.451 | 76.348 | 78.329 | 51.494 | 49.668 | 35.185 | 36.482 | 18.966 | 15.930 |
| 364 | 31.821 | 23.360 | 46.989 | 39.369 | 25.277 | 41.341 | 0.710 | 0.470 | 0.239 | 0.090 | 93.848 | 94.345 | 93.858 | 93.324 | 15.772 | 15.877 | 7.242 | 7.265 | 91.940 | 91.053 | 82.469 | 87.026 | 92.839 | 91.962 | 94.670 | 92.465 | 94.246 | 91.569 | 14.609 | 15.169 | 12.998 | 10.901 | 87.253 | 83.541 | 76.441 | 78.451 | 53.338 | 51.576 | 37.474 | 38.730 | 21.317 | 18.154 |
| 365 | 34.746 | 26.136 | 49.756 | 42.215 | 28.077 | 44.238 | 1.016 | 0.680 | 0.315 | 0.119 | 93.929 | 94.379 | 93.910 | 93.365 | 18.054 | 18.220 | 8.944 | 8.890 | 91.996 | 91.087 | 82.537 | 87.058 | 92.941 | 92.058 | 94.680 | 92.468 | 94.283 | 91.561 | 16.771 | 17.314 | 15.057 | 12.692 | 87.410 | 83.657 | 76.601 | 78.562 | 55.255 | 53.524 | 39.832 | 41.058 | 23.843 | 20.560 |
| 366 | 37.777 | 29.075 | 52.554 | 45.128 | 31.057 | 47.148 | 1.467 | 1.000 | 0.440 | 0.175 | 93.977 | 94.476 | 93.963 | 93.432 | 20.580 | 20.842 | 10.831 | 10.771 | 92.015 | 91.112 | 82.597 | 87.148 | 93.010 | 92.196 | 94.717 | 92.503 | 94.288 | 91.627 | 19.139 | 19.712 | 17.314 | 14.798 | 87.464 | 83.733 | 76.711 | 78.685 | 57.090 | 55.414 | 42.185 | 43.319 | 26.525 | 23.153 |
| 367 | 40.868 | 32.165 | 55.335 | 48.071 | 34.160 | 50.086 | 2.061 | 1.434 | 0.636 | 0.260 | 94.066 | 94.517 | 94.049 | 93.487 | 23.392 | 23.682 | 13.108 | 12.966 | 92.079 | 91.144 | 82.698 | 87.250 | 93.115 | 92.262 | 94.769 | 92.501 | 94.306 | 91.652 | 21.759 | 22.285 | 19.839 | 17.106 | 87.545 | 83.799 | 76.851 | 78.838 | 58.917 | 57.280 | 44.564 | 45.696 | 29.339 | 25.898 |
| 368 | 44.100 | 35.459 | 58.186 | 51.137 | 37.473 | 53.084 | 2.921 | 2.080 | 0.920 | 0.418 | 94.154 | 94.561 | 94.133 | 93.528 | 26.366 | 26.859 | 15.613 | 15.506 | 92.167 | 91.235 | 82.796 | 87.361 | 93.191 | 92.341 | 94.771 | 92.519 | 94.313 | 91.663 | 24.650 | 25.176 | 22.645 | 19.773 | 87.625 | 83.882 | 76.948 | 78.982 | 60.700 | 59.129 | 46.977 | 48.075 | 32.329 | 28.891 |
| 369 | 47.541 | 39.004 | 61.075 | 54.307 | 40.957 | 56.251 | 4.051 | 2.958 | 1.364 | 0.665 | 94.256 | 94.642 | 94.225 | 93.620 | 29.701 | 30.381 | 18.616 | 18.494 | 92.254 | 91.377 | 82.882 | 87.459 | 93.265 | 92.472 | 94.752 | 92.596 | 94.328 | 91.726 | 27.880 | 28.364 | 25.815 | 22.677 | 87.684 | 84.022 | 77.044 | 79.127 | 62.508 | 61.019 | 49.457 | 50.572 | 35.520 | 32.128 |
| 370 | 50.973 | 42.769 | 64.006 | 57.551 | 44.640 | 59.432 | 5.614 | 4.211 | 2.019 | 1.089 | 94.254 | 94.710 | 94.276 | 93.650 | 33.296 | 34.247 | 21.934 | 21.888 | 92.277 | 91.498 | 82.971 | 87.587 | 93.328 | 92.633 | 94.752 | 92.659 | 94.326 | 91.774 | 31.415 | 31.894 | 29.293 | 25.932 | 87.779 | 84.167 | 77.124 | 79.238 | 64.293 | 62.900 | 51.955 | 53.074 | 38.922 | 35.573 |
| 371 | 54.457 | 46.634 | 66.901 | 60.775 | 48.385 | 62.600 | 7.580 | 5.824 | 2.984 | 1.707 | 94.357 | 94.776 | 94.311 | 93.729 | 37.251 | 38.303 | 25.702 | 25.691 | 92.368 | 91.623 | 83.112 | 87.726 | 93.416 | 92.742 | 94.786 | 92.668 | 94.362 | 91.807 | 35.219 | 35.594 | 33.055 | 29.508 | 87.847 | 84.245 | 77.225 | 79.349 | 66.022 | 64.698 | 54.442 | 55.514 | 42.400 | 39.203 |
| 372 | 57.819 | 50.532 | 69.691 | 63.918 | 52.133 | 65.653 | 10.058 | 7.926 | 4.278 | 2.647 | 94.432 | 94.829 | 94.345 | 93.821 | 41.290 | 42.508 | 29.662 | 29.762 | 92.523 | 91.726 | 83.231 | 87.870 | 93.541 | 92.828 | 94.826 | 92.687 | 94.379 | 91.833 | 39.143 | 39.421 | 36.950 | 33.121 | 87.901 | 84.353 | 77.324 | 79.432 | 67.635 | 66.361 | 56.819 | 57.863 | 45.827 | 42.734 |
| 373 | 61.204 | 54.363 | 72.332 | 66.931 | 55.798 | 68.557 | 12.958 | 10.425 | 5.982 | 3.891 | 94.482 | 94.880 | 94.438 | 93.906 | 45.369 | 46.692 | 33.852 | 34.041 | 92.601 | 91.785 | 83.367 | 87.978 | 93.632 | 92.914 | 94.842 | 92.725 | 94.380 | 91.905 | 43.150 | 43.303 | 40.969 | 36.843 | 87.951 | 84.374 | 77.468 | 79.507 | 69.193 | 67.966 | 59.079 | 60.096 | 49.191 | 46.274 |
| 374 | 64.346 | 58.072 | 74.732 | 69.675 | 59.292 | 71.276 | 16.319 | 13.414 | 8.085 | 5.570 | 94.526 | 94.923 | 94.521 | 93.971 | 49.336 | 50.804 | 38.073 | 38.398 | 92.677 | 91.857 | 83.506 | 88.117 | 93.712 | 92.981 | 94.787 | 92.720 | 94.362 | 91.925 | 47.091 | 47.132 | 44.948 | 40.527 | 88.002 | 84.442 | 77.584 | 79.611 | 70.556 | 69.442 | 61.162 | 62.207 | 52.444 | 49.666 |
| 375 | 67.365 | 61.611 | 77.048 | 72.346 | 62.692 | 73.844 | 20.053 | 16.786 | 10.661 | 7.637 | 94.564 | 94.933 | 94.563 | 93.977 | 53.246 | 54.915 | 42.423 | 42.804 | 92.727 | 91.912 | 83.643 | 88.281 | 93.760 | 93.057 | 94.746 | 92.709 | 94.349 | 91.912 | 51.008 | 50.998 | 48.920 | 44.340 | 88.084 | 84.510 | 77.661 | 79.703 | 71.812 | 70.797 | 63.102 | 64.133 | 55.631 | 53.066 |
| 376 | 70.254 | 65.046 | 79.189 | 74.843 | 65.973 | 76.281 | 24.310 | 20.721 | 13.748 | 10.305 | 94.663 | 95.034 | 94.599 | 94.057 | 57.112 | 58.821 | 46.733 | 47.289 | 92.849 | 92.066 | 83.777 | 88.479 | 93.815 | 93.193 | 94.771 | 92.743 | 94.311 | 91.923 | 54.884 | 54.688 | 52.870 | 48.158 | 88.122 | 84.571 | 77.698 | 79.827 | 72.979 | 72.038 | 64.959 | 65.965 | 58.636 | 56.356 |
| 377 | 72.980 | 68.419 | 81.208 | 77.271 | 69.158 | 78.612 | 28.987 | 25.124 | 17.435 | 13.520 | 94.713 | 95.152 | 94.676 | 94.156 | 60.917 | 62.713 | 51.074 | 51.852 | 92.968 | 92.199 | 83.906 | 88.671 | 93.864 | 93.333 | 94.836 | 92.822 | 94.307 | 92.003 | 58.758 | 58.402 | 56.814 | 51.991 | 88.182 | 84.649 | 77.798 | 79.912 | 74.147 | 73.288 | 66.752 | 67.769 | 61.534 | 59.608 |
| 378 | 75.631 | 71.668 | 83.073 | 79.484 | 72.120 | 80.783 | 34.098 | 30.027 | 21.693 | 17.413 | 94.770 | 95.221 | 94.753 | 94.222 | 64.584 | 66.378 | 55.338 | 56.350 | 93.052 | 92.319 | 84.100 | 88.811 | 93.939 | 93.391 | 94.858 | 92.830 | 94.345 | 92.007 | 62.577 | 61.967 | 60.725 | 55.774 | 88.265 | 84.747 | 77.931 | 80.029 | 75.312 | 74.479 | 68.469 | 69.502 | 64.308 | 62.780 |
| 379 | 78.097 | 74.753 | 84.837 | 81.558 | 74.983 | 82.774 | 39.386 | 35.190 | 26.428 | 21.805 | 94.806 | 95.243 | 94.810 | 94.257 | 68.033 | 69.998 | 59.528 | 60.722 | 93.144 | 92.451 | 84.273 | 88.991 | 94.019 | 93.472 | 94.781 | 92.848 | 94.338 | 91.989 | 66.193 | 65.469 | 64.436 | 59.420 | 88.292 | 84.858 | 77.998 | 80.117 | 76.314 | 75.514 | 70.000 | 71.049 | 66.921 | 65.742 |
| 380 | 80.299 | 77.527 | 86.398 | 83.393 | 77.569 | 84.506 | 44.725 | 40.447 | 31.448 | 26.624 | 94.885 | 95.261 | 94.863 | 94.308 | 71.204 | 73.256 | 63.477 | 64.758 | 93.280 | 92.573 | 84.417 | 89.164 | 94.140 | 93.540 | 94.793 | 92.847 | 94.333 | 91.960 | 69.514 | 68.649 | 67.874 | 62.726 | 88.352 | 84.894 | 78.082 | 80.196 | 77.206 | 76.412 | 71.378 | 72.467 | 69.362 | 68.430 |
| 381 | 82.235 | 80.035 | 87.771 | 84.999 | 79.927 | 86.081 | 49.960 | 45.682 | 36.642 | 31.780 | 94.955 | 95.284 | 94.929 | 94.356 | 74.067 | 76.171 | 67.130 | 68.509 | 93.408 | 92.667 | 84.569 | 89.351 | 94.260 | 93.586 | 94.826 | 92.854 | 94.323 | 91.981 | 72.534 | 71.575 | 71.021 | 65.781 | 88.390 | 84.948 | 78.196 | 80.295 | 77.999 | 77.250 | 72.667 | 73.715 | 71.560 | 70.864 |
| 382 | 83.905 | 82.257 | 88.979 | 86.442 | 81.948 | 87.480 | 54.927 | 50.751 | 41.810 | 36.836 | 95.028 | 95.346 | 95.048 | 94.405 | 76.564 | 78.768 | 70.329 | 71.827 | 93.498 | 92.745 | 84.727 | 89.507 | 94.312 | 93.692 | 94.829 | 92.887 | 94.350 | 92.040 | 75.270 | 74.188 | 73.848 | 68.523 | 88.438 | 85.025 | 78.339 | 80.441 | 78.704 | 78.067 | 73.803 | 74.873 | 73.494 | 73.071 |
| 383 | 85.449 | 84.245 | 90.053 | 87.766 | 83.754 | 88.705 | 59.612 | 55.630 | 46.910 | 41.939 | 95.137 | 95.430 | 95.151 | 94.482 | 78.776 | 81.082 | 73.298 | 74.842 | 93.559 | 92.868 | 84.914 | 89.661 | 94.396 | 93.817 | 94.812 | 92.914 | 94.405 | 92.068 | 77.761 | 76.544 | 76.433 | 71.059 | 88.478 | 85.121 | 78.466 | 80.573 | 79.378 | 78.796 | 74.817 | 75.908 | 75.263 | 75.065 |
| 384 | 86.799 | 86.026 | 91.031 | 88.858 | 85.390 | 89.776 | 64.033 | 60.236 | 51.927 | 46.933 | 95.276 | 95.490 | 95.210 | 94.578 | 80.766 | 83.103 | 75.942 | 77.542 | 93.685 | 92.989 | 85.072 | 89.777 | 94.519 | 93.908 | 94.840 | 92.945 | 94.462 | 92.092 | 79.948 | 78.613 | 78.726 | 73.310 | 88.560 | 85.174 | 78.607 | 80.664 | 79.958 | 79.396 | 75.765 | 76.795 | 76.800 | 76.794 |
| 385 | 88.025 | 87.680 | 91.829 | 89.891 | 86.855 | 90.759 | 68.213 | 64.655 | 56.835 | 52.094 | 95.278 | 95.551 | 95.178 | 94.637 | 82.498 | 84.917 | 78.281 | 80.015 | 93.820 | 93.133 | 85.190 | 89.954 | 94.602 | 93.956 | 94.850 | 92.973 | 94.486 | 92.119 | 81.895 | 80.507 | 80.805 | 75.361 | 88.627 | 85.271 | 78.731 | 80.790 | 80.529 | 79.990 | 76.645 | 77.645 | 78.237 | 78.390 |
| 386 | 89.107 | 89.127 | 92.533 | 90.801 | 88.202 | 91.618 | 72.078 | 68.769 | 61.514 | 57.076 | 95.295 | 95.587 | 95.207 | 94.691 | 84.064 | 86.515 | 80.422 | 82.228 | 93.917 | 93.243 | 85.320 | 90.125 | 94.667 | 93.995 | 94.845 | 92.995 | 94.461 | 92.141 | 83.631 | 82.227 | 82.656 | 77.205 | 88.714 | 85.353 | 78.846 | 80.889 | 81.033 | 80.506 | 77.406 | 78.404 | 79.449 | 79.799 |
| 387 | 90.058 | 90.353 | 93.168 | 91.585 | 89.392 | 92.366 | 75.588 | 72.539 | 65.860 | 61.747 | 95.334 | 95.602 | 95.246 | 94.709 | 85.401 | 87.874 | 82.254 | 84.105 | 93.964 | 93.310 | 85.435 | 90.298 | 94.651 | 94.060 | 94.805 | 92.989 | 94.389 | 92.131 | 85.167 | 83.686 | 84.270 | 78.805 | 88.792 | 85.380 | 78.918 | 80.970 | 81.435 | 80.976 | 78.025 | 79.111 | 80.539 | 81.060 |
| 388 | 90.909 | 91.404 | 93.697 | 92.246 | 90.355 | 93.066 | 78.565 | 75.815 | 69.716 | 65.970 | 95.377 | 95.657 | 95.284 | 94.753 | 86.446 | 89.054 | 83.795 | 85.697 | 94.009 | 93.417 | 85.520 | 90.474 | 94.621 | 94.118 | 94.748 | 92.993 | 94.329 | 92.156 | 86.424 | 84.936 | 85.603 | 80.190 | 88.783 | 85.456 | 78.943 | 81.056 | 81.739 | 81.420 | 78.522 | 79.778 | 81.406 | 82.110 |
| 389 | 91.610 | 92.359 | 94.188 | 92.791 | 91.163 | 93.604 | 81.217 | 78.683 | 73.172 | 69.743 | 95.429 | 95.723 | 95.345 | 94.753 | 87.400 | 90.034 | 85.154 | 87.056 | 94.113 | 93.530 | 85.650 | 90.641 | 94.646 | 94.174 | 94.747 | 92.987 | 94.308 | 92.149 | 87.501 | 85.958 | 86.734 | 81.334 | 88.852 | 85.509 | 79.022 | 81.130 | 82.125 | 81.835 | 79.112 | 80.374 | 82.232 | 83.047 |
| 390 | 92.179 | 93.180 | 94.582 | 93.335 | 91.872 | 94.087 | 83.398 | 81.144 | 76.171 | 73.026 | 95.407 | 95.780 | 95.415 | 94.804 | 88.175 | 90.848 | 86.235 | 88.236 | 94.204 | 93.632 | 85.808 | 90.798 | 94.710 | 94.291 | 94.761 | 93.033 | 94.302 | 92.225 | 88.438 | 86.869 | 87.692 | 82.324 | 88.888 | 85.625 | 79.126 | 81.236 | 82.500 | 82.214 | 79.599 | 80.887 | 82.916 | 83.857 |
| 391 | 92.651 | 93.904 | 94.943 | 93.782 | 92.516 | 94.469 | 85.340 | 83.280 | 78.796 | 75.951 | 95.435 | 95.785 | 95.449 | 94.881 | 88.860 | 91.499 | 87.082 | 89.213 | 94.257 | 93.694 | 85.916 | 90.926 | 94.774 | 94.363 | 94.769 | 93.058 | 94.292 | 92.270 | 89.222 | 87.649 | 88.490 | 83.176 | 88.886 | 85.698 | 79.222 | 81.330 | 82.784 | 82.493 | 79.996 | 81.279 | 83.445 | 84.493 |
| 392 | 93.125 | 94.478 | 95.246 | 94.177 | 93.091 | 94.805 | 87.076 | 85.114 | 81.122 | 78.540 | 95.536 | 95.797 | 95.544 | 94.917 | 89.446 | 92.063 | 87.900 | 89.993 | 94.359 | 93.757 | 86.080 | 91.086 | 94.872 | 94.439 | 94.812 | 93.081 | 94.350 | 92.298 | 89.876 | 88.281 | 89.197 | 83.881 | 88.980 | 85.721 | 79.324 | 81.384 | 83.103 | 82.785 | 80.397 | 81.655 | 83.997 | 85.088 |
| 393 | 93.539 | 94.991 | 95.486 | 94.441 | 93.557 | 95.052 | 88.553 | 86.693 | 83.113 | 80.816 | 95.550 | 95.819 | 95.540 | 94.917 | 89.908 | 92.535 | 88.650 | 90.608 | 94.401 | 93.830 | 86.204 | 91.214 | 94.899 | 94.439 | 94.792 | 93.079 | 94.323 | 92.226 | 90.456 | 88.799 | 89.826 | 84.453 | 89.057 | 85.734 | 79.396 | 81.461 | 83.369 | 83.078 | 80.789 | 82.093 | 84.484 | 85.648 |
| 394 | 93.880 | 95.386 | 95.730 | 94.697 | 93.992 | 95.293 | 89.817 | 88.065 | 84.884 | 82.861 | 95.621 | 95.894 | 95.588 | 94.959 | 90.351 | 92.936 | 89.232 | 91.188 | 94.493 | 93.914 | 86.374 | 91.361 | 94.969 | 94.499 | 94.802 | 93.067 | 94.315 | 92.216 | 90.968 | 89.272 | 90.357 | 84.961 | 89.127 | 85.770 | 79.504 | 81.519 | 83.619 | 83.285 | 81.186 | 82.450 | 84.903 | 86.119 |
| 395 | 94.116 | 95.781 | 95.911 | 94.994 | 94.350 | 95.566 | 90.844 | 89.317 | 86.469 | 84.647 | 95.697 | 96.007 | 95.681 | 95.073 | 90.737 | 93.326 | 89.677 | 91.740 | 94.598 | 94.062 | 86.507 | 91.523 | 95.067 | 94.613 | 94.798 | 93.101 | 94.330 | 92.285 | 91.385 | 89.729 | 90.775 | 85.471 | 89.136 | 85.866 | 79.593 | 81.614 | 83.777 | 83.514 | 81.460 | 82.750 | 85.189 | 86.447 |
| 396 | 94.343 | 96.131 | 96.034 | 95.168 | 94.595 | 95.735 | 91.723 | 90.378 | 87.727 | 86.107 | 95.763 | 96.031 | 95.690 | 95.064 | 91.033 | 93.643 | 90.018 | 92.146 | 94.634 | 94.125 | 86.576 | 91.636 | 95.100 | 94.632 | 94.734 | 93.083 | 94.350 | 92.257 | 91.706 | 90.017 | 91.081 | 85.815 | 89.160 | 85.916 | 79.683 | 81.687 | 83.965 | 83.764 | 81.735 | 83.012 | 85.461 | 86.758 |
| 397 | 94.620 | 96.372 | 96.182 | 95.318 | 94.871 | 95.854 | 92.503 | 91.209 | 88.782 | 87.246 | 95.727 | 95.973 | 95.657 | 95.021 | 91.166 | 93.821 | 90.410 | 92.429 | 94.676 | 94.185 | 86.713 | 91.712 | 95.108 | 94.580 | 94.679 | 93.046 | 94.345 | 92.237 | 91.970 | 90.266 | 91.377 | 86.086 | 89.244 | 85.939 | 79.786 | 81.770 | 84.178 | 84.015 | 81.997 | 83.316 | 85.726 | 87.054 |
| 398 | 94.776 | 96.603 | 96.312 | 95.403 | 95.112 | 95.961 | 93.125 | 91.824 | 89.674 | 88.285 | 95.732 | 95.932 | 95.651 | 95.057 | 91.347 | 94.007 | 90.578 | 92.693 | 94.715 | 94.227 | 86.824 | 91.844 | 95.079 | 94.579 | 94.666 | 93.043 | 94.296 | 92.215 | 92.167 | 90.512 | 91.634 | 86.308 | 89.258 | 85.972 | 79.892 | 81.893 | 84.339 | 84.168 | 82.245 | 83.569 | 85.911 | 87.248 |
| 399 | 94.905 | 96.814 | 96.422 | 95.537 | 95.313 | 96.121 | 93.714 | 92.408 | 90.523 | 89.276 | 95.823 | 96.024 | 95.777 | 95.133 | 91.570 | 94.176 | 90.955 | 92.957 | 94.861 | 94.376 | 86.997 | 92.012 | 95.180 | 94.701 | 94.770 | 93.127 | 94.317 | 92.319 | 92.334 | 90.731 | 91.856 | 86.555 | 89.294 | 86.045 | 80.011 | 82.008 | 84.513 | 84.301 | 82.483 | 83.817 | 86.063 | 87.426 |
| 400 | 94.983 | 96.973 | 96.466 | 95.608 | 95.393 | 96.215 | 94.164 | 92.890 | 91.246 | 90.048 | 95.899 | 96.079 | 95.902 | 95.183 | 91.789 | 94.290 | 91.113 | 93.113 | 95.012 | 94.461 | 87.152 | 92.097 | 95.329 | 94.766 | 94.869 | 93.140 | 94.363 | 92.291 | 92.566 | 90.888 | 92.062 | 86.721 | 89.377 | 86.132 | 80.137 | 82.068 | 84.726 | 84.476 | 82.726 | 84.002 | 86.277 | 87.589 |
| 401 | 95.107 | 97.124 | 96.516 | 95.689 | 95.516 | 96.333 | 94.628 | 93.343 | 91.819 | 90.728 | 95.924 | 96.142 | 95.932 | 95.229 | 91.873 | 94.406 | 91.281 | 93.275 | 95.093 | 94.501 | 87.228 | 92.214 | 95.379 | 94.809 | 94.869 | 93.147 | 94.426 | 92.295 | 92.781 | 91.024 | 92.207 | 86.880 | 89.454 | 86.256 | 80.258 | 82.179 | 84.902 | 84.675 | 82.963 | 84.235 | 86.484 | 87.820 |
| 402 | 95.237 | 97.235 | 96.579 | 95.767 | 95.671 | 96.353 | 95.045 | 93.706 | 92.343 | 91.297 | 95.977 | 96.168 | 95.984 | 95.307 | 91.970 | 94.528 | 91.363 | 93.401 | 95.166 | 94.543 | 87.338 | 92.306 | 95.395 | 94.786 | 94.861 | 93.131 | 94.449 | 92.293 | 92.912 | 91.116 | 92.307 | 87.009 | 89.531 | 86.319 | 80.360 | 82.280 | 85.048 | 84.832 | 83.160 | 84.415 | 86.616 | 87.936 |
| 403 | 95.352 | 97.394 | 96.712 | 95.851 | 95.835 | 96.422 | 95.402 | 94.053 | 92.816 | 91.856 | 96.085 | 96.192 | 96.037 | 95.362 | 92.062 | 94.631 | 91.535 | 93.529 | 95.223 | 94.581 | 87.435 | 92.469 | 95.436 | 94.838 | 94.878 | 93.196 | 94.450 | 92.313 | 92.990 | 91.236 | 92.455 | 87.177 | 89.643 | 86.398 | 80.517 | 82.417 | 85.237 | 85.010 | 83.374 | 84.663 | 86.743 | 88.077 |
| 404 | 95.460 | 97.560 | 96.781 | 96.036 | 95.893 | 96.524 | 95.548 | 94.438 | 93.191 | 92.329 | 96.049 | 96.242 | 95.976 | 95.379 | 92.101 | 94.747 | 91.634 | 93.634 | 95.195 | 94.733 | 87.534 | 92.637 | 95.454 | 94.906 | 94.794 | 93.249 | 94.404 | 92.418 | 93.044 | 91.354 | 92.555 | 87.311 | 89.659 | 86.527 | 80.595 | 82.529 | 85.321 | 85.219 | 83.527 | 84.921 | 86.835 | 88.278 |
| 405 | 95.548 | 97.597 | 96.836 | 96.072 | 96.007 | 96.511 | 95.715 | 94.661 | 93.555 | 92.633 | 96.064 | 96.206 | 96.016 | 95.401 | 92.259 | 94.853 | 91.696 | 93.707 | 95.262 | 94.764 | 87.667 | 92.701 | 95.495 | 94.905 | 94.820 | 93.196 | 94.436 | 92.386 | 93.160 | 91.402 | 92.690 | 87.330 | 89.736 | 86.526 | 80.692 | 82.587 | 85.468 | 85.327 | 83.749 | 85.064 | 86.977 | 88.356 |
| 406 | 95.626 | 97.705 | 96.880 | 96.159 | 96.053 | 96.624 | 95.930 | 94.898 | 93.821 | 92.962 | 96.060 | 96.312 | 96.021 | 95.496 | 92.286 | 94.965 | 91.841 | 93.819 | 95.303 | 94.906 | 87.759 | 92.809 | 95.541 | 95.008 | 94.808 | 93.260 | 94.420 | 92.454 | 93.219 | 91.538 | 92.761 | 87.460 | 89.762 | 86.597 | 80.788 | 82.709 | 85.572 | 85.468 | 83.896 | 85.289 | 87.040 | 88.481 |
| 407 | 95.661 | 97.674 | 96.959 | 96.115 | 96.105 | 96.696 | 96.163 | 94.992 | 94.066 | 93.230 | 96.096 | 96.309 | 96.073 | 95.489 | 92.316 | 94.974 | 91.875 | 93.837 | 95.370 | 94.932 | 87.835 | 92.831 | 95.565 | 95.022 | 94.837 | 93.235 | 94.403 | 92.423 | 93.222 | 91.650 | 92.781 | 87.526 | 89.799 | 86.595 | 80.887 | 82.786 | 85.702 | 85.570 | 84.022 | 85.371 | 87.084 | 88.480 |
| 408 | 95.690 | 97.748 | 96.964 | 96.156 | 96.176 | 96.718 | 96.347 | 95.095 | 94.313 | 93.528 | 96.123 | 96.274 | 96.079 | 95.487 | 92.323 | 94.953 | 91.988 | 93.871 | 95.384 | 94.942 | 87.947 | 92.939 | 95.635 | 95.043 | 94.808 | 93.269 | 94.390 | 92.439 | 93.291 | 91.674 | 92.829 | 87.587 | 89.855 | 86.677 | 80.981 | 82.854 | 85.798 | 85.710 | 84.116 | 85.521 | 87.178 | 88.581 |
| 409 | 95.758 | 97.790 | 96.977 | 96.237 | 96.267 | 96.706 | 96.541 | 95.266 | 94.530 | 93.753 | 96.193 | 96.286 | 96.155 | 95.472 | 92.449 | 94.997 | 92.029 | 93.885 | 95.421 | 94.985 | 88.074 | 92.997 | 95.661 | 95.001 | 94.847 | 93.236 | 94.445 | 92.403 | 93.399 | 91.655 | 92.885 | 87.590 | 89.945 | 86.721 | 81.091 | 82.928 | 85.940 | 85.825 | 84.302 | 85.641 | 87.312 | 88.646 |
| 410 | 95.819 | 97.883 | 96.946 | 96.272 | 96.307 | 96.745 | 96.661 | 95.474 | 94.670 | 93.990 | 96.224 | 96.377 | 96.162 | 95.564 | 92.512 | 95.062 | 92.109 | 93.999 | 95.489 | 95.081 | 88.140 | 93.108 | 95.682 | 95.115 | 94.865 | 93.271 | 94.439 | 92.460 | 93.442 | 91.713 | 92.949 | 87.682 | 90.015 | 86.795 | 81.150 | 83.056 | 86.026 | 85.958 | 84.466 | 85.803 | 87.354 | 88.732 |
| 411 | 95.872 | 98.001 | 96.986 | 96.308 | 96.329 | 96.812 | 96.729 | 95.634 | 94.794 | 94.215 | 96.167 | 96.433 | 96.108 | 95.597 | 92.478 | 95.087 | 92.070 | 94.079 | 95.478 | 95.174 | 88.179 | 93.211 | 95.671 | 95.180 | 94.841 | 93.338 | 94.406 | 92.539 | 93.439 | 91.814 | 92.945 | 87.779 | 89.992 | 86.887 | 81.204 | 83.143 | 86.051 | 86.110 | 84.568 | 85.973 | 87.346 | 88.824 |
| 412 | 95.941 | 98.007 | 97.068 | 96.305 | 96.393 | 96.850 | 96.831 | 95.680 | 94.958 | 94.373 | 96.185 | 96.402 | 96.173 | 95.600 | 92.524 | 95.150 | 92.171 | 94.135 | 95.507 | 95.189 | 88.248 | 93.327 | 95.676 | 95.201 | 94.858 | 93.387 | 94.419 | 92.559 | 93.423 | 91.868 | 92.949 | 87.823 | 89.976 | 86.902 | 81.258 | 83.202 | 86.110 | 86.221 | 84.621 | 86.061 | 87.354 | 88.851 |
| 413 | 95.988 | 98.083 | 97.092 | 96.358 | 96.410 | 96.845 | 96.905 | 95.764 | 95.091 | 94.515 | 96.213 | 96.389 | 96.142 | 95.606 | 92.519 | 95.230 | 92.175 | 94.155 | 95.510 | 95.217 | 88.358 | 93.414 | 95.681 | 95.237 | 94.847 | 93.386 | 94.438 | 92.504 | 93.496 | 91.856 | 93.018 | 87.848 | 90.004 | 86.944 | 81.339 | 83.249 | 86.239 | 86.384 | 84.721 | 86.237 | 87.420 | 88.947 |
| 414 | 96.008 | 98.110 | 97.141 | 96.398 | 96.463 | 96.949 | 96.992 | 95.903 | 95.211 | 94.652 | 96.287 | 96.447 | 96.178 | 95.624 | 92.554 | 95.261 | 92.218 | 94.203 | 95.537 | 95.264 | 88.434 | 93.495 | 95.701 | 95.281 | 94.871 | 93.412 | 94.461 | 92.561 | 93.544 | 91.919 | 93.057 | 87.956 | 90.061 | 87.017 | 81.393 | 83.358 | 86.344 | 86.507 | 84.855 | 86.386 | 87.466 | 88.980 |
| 415 | 96.029 | 98.176 | 97.062 | 96.422 | 96.449 | 96.981 | 97.004 | 96.071 | 95.306 | 94.827 | 96.303 | 96.535 | 96.197 | 95.698 | 92.587 | 95.267 | 92.249 | 94.237 | 95.587 | 95.355 | 88.486 | 93.637 | 95.748 | 95.351 | 94.896 | 93.441 | 94.467 | 92.671 | 93.634 | 91.950 | 93.135 | 88.011 | 90.141 | 87.082 | 81.476 | 83.496 | 86.451 | 86.658 | 84.977 | 86.551 | 87.550 | 89.069 |
| 416 | 96.028 | 98.171 | 97.118 | 96.471 | 96.515 | 96.983 | 97.136 | 96.146 | 95.475 | 94.958 | 96.379 | 96.533 | 96.346 | 95.714 | 92.679 | 95.235 | 92.363 | 94.263 | 95.709 | 95.405 | 88.552 | 93.659 | 95.804 | 95.366 | 94.937 | 93.429 | 94.528 | 92.680 | 93.706 | 92.000 | 93.165 | 88.041 | 90.171 | 87.087 | 81.596 | 83.527 | 86.563 | 86.676 | 85.119 | 86.607 | 87.620 | 89.078 |
| 417 | 96.052 | 98.168 | 97.146 | 96.473 | 96.540 | 96.973 | 97.313 | 96.078 | 95.658 | 95.030 | 96.425 | 96.489 | 96.432 | 95.690 | 92.779 | 95.260 | 92.442 | 94.232 | 95.800 | 95.418 | 88.657 | 93.671 | 95.872 | 95.413 | 95.000 | 93.416 | 94.598 | 92.672 | 93.765 | 92.010 | 93.257 | 88.033 | 90.192 | 87.097 | 81.680 | 83.524 | 86.648 | 86.706 | 85.208 | 86.647 | 87.680 | 89.044 |
| 418 | 96.049 | 98.195 | 97.142 | 96.429 | 96.545 | 96.954 | 97.423 | 96.098 | 95.766 | 95.139 | 96.417 | 96.496 | 96.425 | 95.707 | 92.771 | 95.280 | 92.512 | 94.242 | 95.775 | 95.432 | 88.723 | 93.687 | 95.859 | 95.369 | 95.004 | 93.436 | 94.583 | 92.611 | 93.772 | 91.978 | 93.286 | 88.020 | 90.235 | 87.110 | 81.793 | 83.563 | 86.757 | 86.760 | 85.340 | 86.726 | 87.711 | 89.104 |
| 419 | 96.062 | 98.228 | 97.146 | 96.429 | 96.589 | 96.969 | 97.436 | 96.202 | 95.839 | 95.230 | 96.405 | 96.511 | 96.418 | 95.724 | 92.795 | 95.262 | 92.521 | 94.257 | 95.713 | 95.429 | 88.773 | 93.732 | 95.884 | 95.287 | 95.010 | 93.431 | 94.573 | 92.641 | 93.745 | 92.027 | 93.275 | 88.032 | 90.249 | 87.150 | 81.857 | 83.622 | 86.816 | 86.887 | 85.427 | 86.795 | 87.681 | 89.178 |
| 420 | 96.130 | 98.299 | 97.150 | 96.387 | 96.609 | 96.965 | 97.433 | 96.335 | 95.852 | 95.340 | 96.382 | 96.531 | 96.367 | 95.781 | 92.793 | 95.309 | 92.491 | 94.314 | 95.739 | 95.449 | 88.798 | 93.794 | 95.849 | 95.307 | 95.016 | 93.415 | 94.589 | 92.631 | 93.731 | 92.048 | 93.309 | 88.055 | 90.354 | 87.149 | 81.929 | 83.688 | 86.912 | 87.036 | 85.543 | 86.931 | 87.719 | 89.274 |
| 421 | 96.160 | 98.336 | 97.217 | 96.418 | 96.641 | 97.049 | 97.492 | 96.402 | 95.935 | 95.408 | 96.414 | 96.561 | 96.377 | 95.783 | 92.863 | 95.355 | 92.498 | 94.400 | 95.821 | 95.491 | 88.876 | 93.865 | 95.881 | 95.428 | 94.965 | 93.435 | 94.626 | 92.687 | 93.750 | 92.123 | 93.323 | 88.172 | 90.359 | 87.242 | 81.917 | 83.746 | 86.919 | 87.142 | 85.604 | 87.026 | 87.735 | 89.278 |
| 422 | 96.216 | 98.322 | 97.266 | 96.441 | 96.673 | 97.083 | 97.550 | 96.411 | 96.022 | 95.479 | 96.465 | 96.564 | 96.435 | 95.771 | 92.887 | 95.364 | 92.479 | 94.382 | 95.871 | 95.529 | 88.964 | 93.930 | 95.914 | 95.469 | 95.023 | 93.498 | 94.697 | 92.720 | 93.786 | 92.177 | 93.420 | 88.173 | 90.413 | 87.305 | 82.010 | 83.833 | 86.974 | 87.281 | 85.715 | 87.191 | 87.831 | 89.291 |
| 423 | 96.232 | 98.326 | 97.297 | 96.504 | 96.753 | 97.145 | 97.602 | 96.486 | 96.120 | 95.619 | 96.528 | 96.630 | 96.494 | 95.822 | 92.896 | 95.409 | 92.590 | 94.435 | 95.881 | 95.607 | 89.028 | 93.982 | 95.935 | 95.475 | 95.064 | 93.551 | 94.724 | 92.746 | 93.805 | 92.195 | 93.410 | 88.165 | 90.486 | 87.363 | 82.127 | 83.876 | 87.089 | 87.388 | 85.861 | 87.296 | 87.914 | 89.371 |
| 424 | 96.280 | 98.288 | 97.338 | 96.548 | 96.812 | 97.156 | 97.635 | 96.509 | 96.196 | 95.672 | 96.540 | 96.616 | 96.514 | 95.828 | 92.917 | 95.369 | 92.598 | 94.429 | 95.866 | 95.612 | 89.073 | 94.011 | 95.964 | 95.462 | 95.086 | 93.587 | 94.729 | 92.768 | 93.874 | 92.185 | 93.422 | 88.169 | 90.509 | 87.375 | 82.163 | 83.899 | 87.112 | 87.483 | 85.944 | 87.348 | 87.924 | 89.374 |
| 425 | 96.330 | 98.275 | 97.350 | 96.532 | 96.802 | 97.159 | 97.660 | 96.500 | 96.227 | 95.697 | 96.542 | 96.609 | 96.530 | 95.854 | 92.936 | 95.358 | 92.669 | 94.449 | 95.875 | 95.609 | 89.080 | 94.090 | 95.958 | 95.509 | 95.060 | 93.615 | 94.708 | 92.804 | 93.866 | 92.185 | 93.391 | 88.227 | 90.530 | 87.398 | 82.185 | 83.964 | 87.145 | 87.528 | 85.970 | 87.401 | 87.915 | 89.364 |
| 426 | 96.350 | 98.330 | 97.384 | 96.562 | 96.826 | 97.161 | 97.699 | 96.548 | 96.328 | 95.765 | 96.535 | 96.615 | 96.513 | 95.868 | 92.983 | 95.366 | 92.650 | 94.466 | 95.899 | 95.638 | 89.140 | 94.159 | 95.983 | 95.532 | 95.054 | 93.637 | 94.723 | 92.832 | 93.913 | 92.208 | 93.454 | 88.304 | 90.513 | 87.485 | 82.238 | 84.051 | 87.165 | 87.613 | 86.024 | 87.526 | 87.956 | 89.437 |
| 427 | 96.320 | 98.424 | 97.355 | 96.607 | 96.840 | 97.174 | 97.795 | 96.645 | 96.436 | 95.908 | 96.588 | 96.697 | 96.534 | 95.925 | 93.030 | 95.433 | 92.764 | 94.541 | 96.006 | 95.728 | 89.258 | 94.217 | 96.073 | 95.550 | 95.137 | 93.694 | 94.825 | 92.912 | 93.994 | 92.251 | 93.556 | 88.384 | 90.623 | 87.580 | 82.367 | 84.128 | 87.316 | 87.701 | 86.158 | 87.674 | 88.081 | 89.559 |
| 428 | 96.340 | 98.436 | 97.383 | 96.620 | 96.871 | 97.178 | 97.873 | 96.715 | 96.527 | 95.984 | 96.619 | 96.696 | 96.571 | 95.958 | 93.108 | 95.481 | 92.805 | 94.584 | 96.048 | 95.748 | 89.306 | 94.251 | 96.111 | 95.568 | 95.198 | 93.709 | 94.829 | 92.946 | 94.025 | 92.269 | 93.545 | 88.428 | 90.641 | 87.636 | 82.391 | 84.209 | 87.389 | 87.720 | 86.247 | 87.715 | 88.107 | 89.606 |
| 429 | 96.440 | 98.431 | 97.442 | 96.626 | 96.914 | 97.162 | 97.865 | 96.742 | 96.570 | 96.027 | 96.649 | 96.643 | 96.601 | 95.954 | 93.115 | 95.492 | 92.846 | 94.581 | 96.008 | 95.720 | 89.299 | 94.295 | 96.080 | 95.581 | 95.182 | 93.741 | 94.795 | 92.979 | 94.014 | 92.314 | 93.542 | 88.430 | 90.648 | 87.673 | 82.440 | 84.268 | 87.430 | 87.808 | 86.318 | 87.776 | 88.088 | 89.644 |
| 430 | 96.510 | 98.445 | 97.496 | 96.643 | 96.937 | 97.185 | 97.901 | 96.794 | 96.614 | 96.089 | 96.679 | 96.654 | 96.658 | 95.977 | 93.159 | 95.520 | 92.838 | 94.605 | 96.025 | 95.763 | 89.381 | 94.353 | 96.081 | 95.590 | 95.178 | 93.752 | 94.809 | 92.999 | 93.996 | 92.339 | 93.570 | 88.421 | 90.701 | 87.688 | 82.551 | 84.338 | 87.511 | 87.888 | 86.408 | 87.833 | 88.105 | 89.642 |
| 431 | 96.465 | 98.480 | 97.491 | 96.668 | 96.936 | 97.200 | 97.998 | 96.879 | 96.749 | 96.174 | 96.731 | 96.684 | 96.727 | 95.964 | 93.232 | 95.560 | 92.915 | 94.670 | 96.103 | 95.832 | 89.539 | 94.414 | 96.160 | 95.612 | 95.237 | 93.794 | 94.885 | 93.023 | 94.089 | 92.360 | 93.706 | 88.479 | 90.795 | 87.668 | 82.696 | 84.414 | 87.567 | 87.972 | 86.524 | 87.889 | 88.158 | 89.663 |
| 432 | 96.415 | 98.487 | 97.488 | 96.699 | 96.936 | 97.205 | 98.073 | 96.894 | 96.827 | 96.206 | 96.739 | 96.758 | 96.751 | 95.983 | 93.278 | 95.578 | 92.946 | 94.735 | 96.157 | 95.896 | 89.609 | 94.490 | 96.213 | 95.662 | 95.300 | 93.846 | 94.942 | 93.083 | 94.120 | 92.431 | 93.740 | 88.546 | 90.837 | 87.747 | 82.794 | 84.504 | 87.653 | 88.029 | 86.601 | 87.942 | 88.150 | 89.669 |
| 433 | 96.510 | 98.499 | 97.506 | 96.706 | 96.969 | 97.215 | 98.054 | 96.907 | 96.833 | 96.242 | 96.718 | 96.765 | 96.723 | 96.004 | 93.248 | 95.618 | 92.946 | 94.770 | 96.101 | 95.893 | 89.604 | 94.531 | 96.187 | 95.702 | 95.329 | 93.887 | 94.937 | 93.106 | 94.144 | 92.506 | 93.714 | 88.610 | 90.832 | 87.866 | 82.870 | 84.563 | 87.648 | 88.115 | 86.578 | 88.041 | 88.154 | 89.718 |
| 434 | 96.558 | 98.495 | 97.442 | 96.689 | 97.009 | 97.209 | 98.038 | 96.869 | 96.860 | 96.299 | 96.702 | 96.747 | 96.729 | 95.971 | 93.242 | 95.612 | 92.959 | 94.780 | 96.095 | 95.873 | 89.605 | 94.541 | 96.166 | 95.683 | 95.327 | 93.867 | 94.942 | 93.135 | 94.177 | 92.539 | 93.718 | 88.641 | 90.903 | 87.916 | 82.942 | 84.617 | 87.698 | 88.193 | 86.616 | 88.092 | 88.208 | 89.743 |
| 435 | 96.541 | 98.493 | 97.426 | 96.693 | 97.023 | 97.232 | 98.119 | 96.935 | 96.991 | 96.363 | 96.765 | 96.747 | 96.807 | 95.980 | 93.320 | 95.638 | 93.055 | 94.796 | 96.175 | 95.913 | 89.694 | 94.563 | 96.207 | 95.667 | 95.382 | 93.865 | 95.044 | 93.152 | 94.223 | 92.538 | 93.797 | 88.690 | 90.987 | 87.951 | 83.048 | 84.728 | 87.794 | 88.241 | 86.722 | 88.151 | 88.316 | 89.773 |
| 436 | 96.538 | 98.593 | 97.444 | 96.720 | 97.067 | 97.292 | 98.165 | 97.047 | 97.037 | 96.450 | 96.797 | 96.732 | 96.811 | 96.041 | 93.338 | 95.678 | 93.160 | 94.872 | 96.179 | 95.993 | 89.720 | 94.636 | 96.198 | 95.750 | 95.372 | 93.949 | 95.070 | 93.254 | 94.264 | 92.579 | 93.896 | 88.810 | 91.028 | 88.008 | 83.164 | 84.856 | 87.871 | 88.337 | 86.824 | 88.282 | 88.388 | 89.855 |
| 437 | 96.561 | 98.647 | 97.489 | 96.776 | 97.085 | 97.306 | 98.212 | 97.027 | 97.057 | 96.472 | 96.803 | 96.808 | 96.780 | 96.091 | 93.358 | 95.697 | 93.184 | 94.927 | 96.175 | 96.058 | 89.740 | 94.721 | 96.194 | 95.809 | 95.394 | 94.013 | 95.042 | 93.334 | 94.308 | 92.616 | 93.927 | 88.880 | 91.045 | 88.071 | 83.253 | 84.933 | 87.916 | 88.380 | 86.878 | 88.394 | 88.372 | 89.867 |
| 438 | 96.551 | 98.668 | 97.478 | 96.783 | 97.088 | 97.297 | 98.285 | 97.051 | 97.127 | 96.518 | 96.822 | 96.870 | 96.811 | 96.111 | 93.398 | 95.755 | 93.254 | 94.994 | 96.226 | 96.079 | 89.804 | 94.734 | 96.238 | 95.846 | 95.465 | 94.052 | 95.084 | 93.404 | 94.352 | 92.666 | 93.975 | 88.939 | 91.150 | 88.133 | 83.391 | 85.029 | 87.942 | 88.452 | 86.954 | 88.479 | 88.429 | 89.950 |
| 439 | 96.547 | 98.671 | 97.486 | 96.801 | 97.090 | 97.310 | 98.372 | 97.112 | 97.225 | 96.541 | 96.867 | 96.928 | 96.896 | 96.154 | 93.502 | 95.802 | 93.320 | 95.035 | 96.327 | 96.088 | 89.924 | 94.763 | 96.329 | 95.858 | 95.591 | 94.125 | 95.218 | 93.462 | 94.430 | 92.740 | 94.048 | 88.957 | 91.310 | 88.189 | 83.531 | 85.117 | 88.042 | 88.528 | 87.093 | 88.573 | 88.518 | 90.026 |
| 440 | 96.584 | 98.654 | 97.501 | 96.818 | 97.134 | 97.360 | 98.371 | 97.170 | 97.220 | 96.602 | 96.850 | 96.928 | 96.857 | 96.191 | 93.543 | 95.856 | 93.378 | 95.085 | 96.336 | 96.111 | 89.972 | 94.835 | 96.304 | 95.867 | 95.607 | 94.180 | 95.280 | 93.504 | 94.428 | 92.766 | 94.039 | 88.960 | 91.325 | 88.265 | 83.584 | 85.212 | 88.029 | 88.573 | 87.122 | 88.650 | 88.557 | 90.049 |
| 441 | 96.631 | 98.633 | 97.544 | 96.807 | 97.167 | 97.350 | 98.340 | 97.157 | 97.148 | 96.623 | 96.804 | 96.908 | 96.834 | 96.163 | 93.556 | 95.843 | 93.390 | 95.105 | 96.302 | 96.171 | 89.951 | 94.928 | 96.234 | 95.890 | 95.532 | 94.210 | 95.233 | 93.517 | 94.427 | 92.801 | 94.017 | 88.976 | 91.320 | 88.296 | 83.636 | 85.270 | 88.026 | 88.591 | 87.128 | 88.697 | 88.529 | 90.029 |
| 442 | 96.587 | 98.651 | 97.533 | 96.829 | 97.160 | 97.357 | 98.401 | 97.169 | 97.243 | 96.661 | 96.832 | 96.953 | 96.827 | 96.160 | 93.621 | 95.866 | 93.420 | 95.144 | 96.364 | 96.216 | 90.021 | 94.985 | 96.281 | 95.852 | 95.625 | 94.204 | 95.312 | 93.531 | 94.506 | 92.788 | 94.085 | 88.978 | 91.386 | 88.366 | 83.763 | 85.385 | 88.123 | 88.651 | 87.206 | 88.732 | 88.575 | 90.064 |
| 443 | 96.588 | 98.724 | 97.527 | 96.838 | 97.159 | 97.381 | 98.453 | 97.210 | 97.319 | 96.713 | 96.849 | 96.940 | 96.859 | 96.222 | 93.630 | 95.923 | 93.436 | 95.203 | 96.396 | 96.261 | 90.101 | 95.026 | 96.364 | 95.872 | 95.678 | 94.254 | 95.355 | 93.656 | 94.523 | 92.887 | 94.116 | 89.084 | 91.393 | 88.487 | 83.878 | 85.572 | 88.162 | 88.742 | 87.262 | 88.794 | 88.584 | 90.128 |
| 444 | 96.652 | 98.772 | 97.535 | 96.866 | 97.184 | 97.393 | 98.495 | 97.299 | 97.403 | 96.770 | 96.892 | 96.933 | 96.876 | 96.266 | 93.679 | 95.982 | 93.513 | 95.263 | 96.431 | 96.300 | 90.173 | 95.092 | 96.378 | 95.900 | 95.756 | 94.318 | 95.424 | 93.694 | 94.618 | 92.916 | 94.205 | 89.131 | 91.470 | 88.538 | 84.013 | 85.630 | 88.237 | 88.794 | 87.353 | 88.886 | 88.639 | 90.136 |
| 445 | 96.724 | 98.725 | 97.581 | 96.840 | 97.242 | 97.403 | 98.517 | 97.287 | 97.449 | 96.816 | 96.978 | 96.936 | 96.926 | 96.272 | 93.729 | 95.975 | 93.611 | 95.293 | 96.476 | 96.345 | 90.197 | 95.125 | 96.361 | 95.914 | 95.777 | 94.383 | 95.450 | 93.733 | 94.634 | 92.956 | 94.258 | 89.175 | 91.530 | 88.582 | 84.089 | 85.688 | 88.300 | 88.795 | 87.461 | 88.929 | 88.699 | 90.157 |
| 446 | 96.719 | 98.766 | 97.596 | 96.856 | 97.236 | 97.438 | 98.572 | 97.357 | 97.514 | 96.848 | 97.041 | 97.002 | 97.016 | 96.299 | 93.789 | 96.004 | 93.763 | 95.316 | 96.553 | 96.413 | 90.315 | 95.191 | 96.463 | 95.997 | 95.873 | 94.438 | 95.594 | 93.783 | 94.748 | 92.997 | 94.368 | 89.243 | 91.641 | 88.666 | 84.226 | 85.797 | 88.446 | 88.878 | 87.566 | 89.028 | 88.783 | 90.281 |
| 447 | 96.658 | 98.718 | 97.575 | 96.796 | 97.222 | 97.403 | 98.585 | 97.346 | 97.569 | 96.865 | 97.048 | 96.978 | 97.005 | 96.278 | 93.808 | 96.035 | 93.780 | 95.314 | 96.565 | 96.346 | 90.365 | 95.180 | 96.484 | 95.964 | 95.905 | 94.393 | 95.645 | 93.811 | 94.737 | 92.959 | 94.392 | 89.246 | 91.712 | 88.698 | 84.348 | 85.873 | 88.506 | 88.894 | 87.596 | 89.077 | 88.804 | 90.294 |
| 448 | 96.680 | 98.731 | 97.604 | 96.813 | 97.245 | 97.447 | 98.586 | 97.337 | 97.564 | 96.854 | 97.028 | 96.923 | 96.990 | 96.309 | 93.812 | 96.084 | 93.778 | 95.391 | 96.516 | 96.366 | 90.364 | 95.261 | 96.481 | 96.020 | 95.888 | 94.459 | 95.623 | 93.877 | 94.714 | 93.020 | 94.347 | 89.311 | 91.721 | 88.793 | 84.397 | 85.947 | 88.446 | 88.915 | 87.571 | 89.096 | 88.799 | 90.259 |
| 449 | 96.708 | 98.796 | 97.608 | 96.873 | 97.244 | 97.504 | 98.599 | 97.422 | 97.600 | 96.915 | 97.032 | 97.010 | 96.970 | 96.333 | 93.867 | 96.126 | 93.760 | 95.483 | 96.566 | 96.437 | 90.411 | 95.357 | 96.488 | 96.092 | 95.949 | 94.549 | 95.633 | 93.960 | 94.767 | 93.063 | 94.401 | 89.382 | 91.787 | 88.916 | 84.489 | 86.010 | 88.513 | 88.988 | 87.647 | 89.164 | 88.839 | 90.287 |
| 450 | 96.706 | 98.837 | 97.607 | 96.904 | 97.316 | 97.557 | 98.589 | 97.450 | 97.573 | 96.959 | 97.006 | 97.034 | 96.969 | 96.355 | 93.906 | 96.133 | 93.794 | 95.562 | 96.573 | 96.480 | 90.448 | 95.445 | 96.516 | 96.132 | 95.953 | 94.622 | 95.631 | 94.045 | 94.778 | 93.158 | 94.417 | 89.486 | 91.747 | 89.001 | 84.529 | 86.141 | 88.446 | 89.044 | 87.638 | 89.201 | 88.794 | 90.320 |
| 451 | 96.684 | 98.827 | 97.620 | 96.890 | 97.303 | 97.502 | 98.581 | 97.490 | 97.577 | 96.995 | 96.964 | 97.028 | 96.966 | 96.348 | 93.927 | 96.158 | 93.812 | 95.530 | 96.602 | 96.445 | 90.520 | 95.456 | 96.537 | 96.111 | 95.972 | 94.604 | 95.714 | 94.020 | 94.841 | 93.131 | 94.482 | 89.478 | 91.770 | 89.014 | 84.641 | 86.216 | 88.478 | 89.059 | 87.711 | 89.235 | 88.769 | 90.333 |
| 452 | 96.705 | 98.749 | 97.627 | 96.843 | 97.331 | 97.419 | 98.582 | 97.453 | 97.634 | 97.011 | 96.995 | 96.982 | 96.971 | 96.333 | 93.942 | 96.157 | 93.849 | 95.517 | 96.606 | 96.431 | 90.561 | 95.451 | 96.511 | 96.052 | 95.993 | 94.589 | 95.738 | 93.992 | 94.856 | 93.127 | 94.501 | 89.440 | 91.841 | 88.981 | 84.734 | 86.243 | 88.540 | 89.073 | 87.725 | 89.267 | 88.814 | 90.316 |
| 453 | 96.697 | 98.753 | 97.605 | 96.888 | 97.305 | 97.454 | 98.621 | 97.456 | 97.721 | 97.041 | 97.028 | 96.995 | 97.019 | 96.369 | 93.999 | 96.160 | 93.872 | 95.554 | 96.644 | 96.466 | 90.635 | 95.498 | 96.543 | 96.107 | 96.079 | 94.671 | 95.810 | 94.082 | 94.904 | 93.181 | 94.547 | 89.464 | 91.951 | 89.011 | 84.794 | 86.310 | 88.632 | 89.150 | 87.775 | 89.327 | 88.918 | 90.345 |
| 454 | 96.726 | 98.762 | 97.620 | 96.860 | 97.320 | 97.454 | 98.640 | 97.427 | 97.766 | 97.054 | 97.046 | 97.027 | 97.051 | 96.327 | 94.036 | 96.157 | 93.923 | 95.568 | 96.674 | 96.442 | 90.671 | 95.509 | 96.519 | 96.057 | 96.084 | 94.656 | 95.801 | 94.112 | 94.852 | 93.187 | 94.558 | 89.478 | 91.979 | 89.052 | 84.856 | 86.384 | 88.669 | 89.184 | 87.811 | 89.386 | 88.916 | 90.363 |
| 455 | 96.771 | 98.767 | 97.642 | 96.882 | 97.373 | 97.508 | 98.685 | 97.468 | 97.781 | 97.076 | 97.054 | 97.029 | 97.064 | 96.396 | 94.049 | 96.195 | 93.999 | 95.633 | 96.688 | 96.478 | 90.710 | 95.556 | 96.545 | 96.044 | 96.114 | 94.647 | 95.876 | 94.116 | 94.905 | 93.201 | 94.645 | 89.508 | 92.024 | 89.093 | 84.996 | 86.491 | 88.657 | 89.258 | 87.912 | 89.457 | 88.940 | 90.409 |
| 456 | 96.837 | 98.744 | 97.695 | 96.834 | 97.369 | 97.468 | 98.716 | 97.453 | 97.781 | 97.052 | 97.127 | 97.008 | 97.073 | 96.419 | 94.075 | 96.185 | 94.076 | 95.627 | 96.736 | 96.514 | 90.752 | 95.564 | 96.593 | 96.055 | 96.179 | 94.665 | 95.917 | 94.151 | 94.935 | 93.205 | 94.659 | 89.560 | 92.063 | 89.156 | 85.071 | 86.584 | 88.716 | 89.248 | 87.973 | 89.463 | 88.965 | 90.419 |
| 457 | 96.827 | 98.778 | 97.646 | 96.793 | 97.386 | 97.496 | 98.754 | 97.442 | 97.764 | 97.056 | 97.121 | 97.024 | 97.074 | 96.421 | 94.097 | 96.221 | 94.112 | 95.699 | 96.732 | 96.579 | 90.814 | 95.581 | 96.592 | 96.141 | 96.212 | 94.740 | 95.912 | 94.218 | 94.970 | 93.291 | 94.636 | 89.613 | 92.102 | 89.233 | 85.081 | 86.667 | 88.725 | 89.281 | 87.906 | 89.483 | 89.000 | 90.423 |
| 458 | 96.806 | 98.810 | 97.665 | 96.814 | 97.380 | 97.514 | 98.704 | 97.479 | 97.729 | 97.133 | 97.076 | 97.031 | 97.046 | 96.408 | 94.109 | 96.262 | 94.056 | 95.727 | 96.683 | 96.561 | 90.818 | 95.618 | 96.562 | 96.175 | 96.171 | 94.802 | 95.907 | 94.291 | 94.977 | 93.362 | 94.602 | 89.672 | 92.090 | 89.297 | 85.120 | 86.717 | 88.726 | 89.313 | 87.869 | 89.541 | 88.967 | 90.458 |
| 459 | 96.828 | 98.831 | 97.693 | 96.835 | 97.438 | 97.503 | 98.686 | 97.523 | 97.748 | 97.175 | 97.061 | 97.023 | 97.057 | 96.406 | 94.130 | 96.267 | 94.064 | 95.753 | 96.699 | 96.575 | 90.839 | 95.671 | 96.587 | 96.149 | 96.173 | 94.802 | 95.888 | 94.277 | 94.989 | 93.383 | 94.602 | 89.698 | 92.104 | 89.334 | 85.201 | 86.775 | 88.740 | 89.318 | 87.939 | 89.618 | 88.974 | 90.549 |
| 460 | 96.801 | 98.773 | 97.732 | 96.863 | 97.483 | 97.513 | 98.747 | 97.578 | 97.790 | 97.186 | 97.118 | 97.056 | 97.106 | 96.424 | 94.177 | 96.276 | 94.146 | 95.801 | 96.766 | 96.651 | 90.908 | 95.726 | 96.683 | 96.218 | 96.285 | 94.863 | 95.970 | 94.312 | 95.067 | 93.411 | 94.729 | 89.726 | 92.194 | 89.339 | 85.338 | 86.833 | 88.821 | 89.348 | 88.074 | 89.684 | 89.043 | 90.624 |
| 461 | 96.798 | 98.773 | 97.748 | 96.902 | 97.432 | 97.544 | 98.771 | 97.581 | 97.792 | 97.171 | 97.152 | 97.062 | 97.149 | 96.438 | 94.227 | 96.270 | 94.226 | 95.843 | 96.771 | 96.679 | 90.945 | 95.785 | 96.696 | 96.313 | 96.328 | 94.925 | 95.994 | 94.412 | 95.067 | 93.485 | 94.740 | 89.814 | 92.221 | 89.447 | 85.375 | 86.965 | 88.822 | 89.371 | 88.062 | 89.716 | 89.118 | 90.658 |
| 462 | 96.788 | 98.806 | 97.713 | 96.938 | 97.409 | 97.545 | 98.767 | 97.603 | 97.770 | 97.191 | 97.098 | 97.112 | 97.132 | 96.429 | 94.186 | 96.289 | 94.186 | 95.848 | 96.737 | 96.674 | 90.946 | 95.794 | 96.655 | 96.307 | 96.309 | 94.919 | 96.030 | 94.409 | 95.083 | 93.516 | 94.749 | 89.799 | 92.213 | 89.501 | 85.403 | 87.023 | 88.796 | 89.429 | 88.066 | 89.745 | 89.094 | 90.687 |
| 463 | 96.814 | 98.832 | 97.687 | 96.948 | 97.409 | 97.557 | 98.731 | 97.625 | 97.783 | 97.258 | 97.093 | 97.136 | 97.131 | 96.470 | 94.213 | 96.350 | 94.159 | 95.880 | 96.761 | 96.676 | 90.940 | 95.853 | 96.671 | 96.291 | 96.286 | 94.942 | 96.031 | 94.448 | 95.078 | 93.513 | 94.769 | 89.855 | 92.259 | 89.554 | 85.472 | 87.064 | 88.857 | 89.455 | 88.118 | 89.772 | 89.134 | 90.679 |
| 464 | 96.814 | 98.865 | 97.694 | 96.927 | 97.450 | 97.579 | 98.783 | 97.623 | 97.825 | 97.270 | 97.126 | 97.123 | 97.161 | 96.498 | 94.231 | 96.382 | 94.221 | 95.893 | 96.794 | 96.674 | 90.999 | 95.816 | 96.708 | 96.307 | 96.326 | 94.980 | 96.068 | 94.453 | 95.159 | 93.545 | 94.830 | 89.883 | 92.363 | 89.562 | 85.565 | 87.107 | 88.926 | 89.457 | 88.168 | 89.790 | 89.177 | 90.663 |
| 465 | 96.802 | 98.919 | 97.694 | 96.944 | 97.406 | 97.604 | 98.780 | 97.649 | 97.829 | 97.313 | 97.162 | 97.136 | 97.131 | 96.514 | 94.240 | 96.446 | 94.310 | 95.981 | 96.774 | 96.715 | 91.034 | 95.884 | 96.720 | 96.361 | 96.339 | 95.087 | 96.078 | 94.570 | 95.194 | 93.642 | 94.838 | 90.010 | 92.413 | 89.644 | 85.616 | 87.218 | 88.906 | 89.507 | 88.152 | 89.890 | 89.133 | 90.705 |
| 466 | 96.783 | 98.881 | 97.666 | 96.958 | 97.370 | 97.592 | 98.789 | 97.650 | 97.849 | 97.310 | 97.170 | 97.127 | 97.115 | 96.507 | 94.282 | 96.444 | 94.289 | 95.998 | 96.772 | 96.713 | 91.034 | 95.915 | 96.673 | 96.322 | 96.388 | 95.068 | 96.112 | 94.572 | 95.239 | 93.620 | 94.865 | 90.042 | 92.466 | 89.683 | 85.680 | 87.292 | 88.917 | 89.526 | 88.167 | 89.913 | 89.151 | 90.714 |
| 467 | 96.787 | 98.775 | 97.680 | 96.904 | 97.404 | 97.575 | 98.771 | 97.633 | 97.828 | 97.278 | 97.172 | 97.131 | 97.080 | 96.508 | 94.296 | 96.435 | 94.270 | 96.013 | 96.779 | 96.738 | 91.062 | 95.895 | 96.669 | 96.268 | 96.432 | 95.005 | 96.186 | 94.553 | 95.279 | 93.568 | 94.917 | 89.972 | 92.481 | 89.633 | 85.776 | 87.261 | 89.003 | 89.528 | 88.257 | 89.900 | 89.194 | 90.715 |
| 468 | 96.811 | 98.765 | 97.705 | 96.901 | 97.421 | 97.538 | 98.741 | 97.612 | 97.825 | 97.257 | 97.227 | 97.171 | 97.142 | 96.530 | 94.338 | 96.446 | 94.232 | 96.044 | 96.780 | 96.795 | 91.077 | 95.943 | 96.658 | 96.307 | 96.386 | 95.033 | 96.172 | 94.609 | 95.244 | 93.620 | 94.903 | 90.026 | 92.466 | 89.689 | 85.812 | 87.327 | 89.041 | 89.541 | 88.300 | 89.938 | 89.223 | 90.736 |
| 469 | 96.821 | 98.815 | 97.724 | 96.895 | 97.420 | 97.504 | 98.752 | 97.609 | 97.828 | 97.223 | 97.196 | 97.137 | 97.134 | 96.546 | 94.317 | 96.443 | 94.273 | 96.018 | 96.787 | 96.758 | 91.120 | 95.924 | 96.673 | 96.291 | 96.347 | 95.027 | 96.161 | 94.576 | 95.174 | 93.638 | 94.854 | 90.031 | 92.419 | 89.745 | 85.821 | 87.398 | 89.031 | 89.544 | 88.273 | 89.992 | 89.180 | 90.758 |
| 470 | 96.825 | 98.887 | 97.682 | 96.913 | 97.365 | 97.522 | 98.720 | 97.637 | 97.795 | 97.231 | 97.185 | 97.173 | 97.093 | 96.549 | 94.361 | 96.458 | 94.297 | 96.036 | 96.811 | 96.808 | 91.132 | 96.056 | 96.688 | 96.368 | 96.318 | 95.125 | 96.129 | 94.675 | 95.095 | 93.728 | 94.804 | 90.121 | 92.433 | 89.817 | 85.835 | 87.489 | 89.037 | 89.580 | 88.279 | 90.074 | 89.135 | 90.815 |
| 471 | 96.836 | 98.866 | 97.704 | 96.953 | 97.409 | 97.575 | 98.738 | 97.659 | 97.819 | 97.245 | 97.174 | 97.175 | 97.078 | 96.548 | 94.376 | 96.470 | 94.354 | 96.044 | 96.792 | 96.846 | 91.185 | 96.079 | 96.666 | 96.409 | 96.401 | 95.141 | 96.199 | 94.683 | 95.157 | 93.759 | 94.876 | 90.122 | 92.535 | 89.800 | 85.936 | 87.497 | 89.116 | 89.612 | 88.395 | 90.042 | 89.214 | 90.842 |
| 472 | 96.853 | 98.841 | 97.689 | 96.904 | 97.433 | 97.617 | 98.766 | 97.668 | 97.838 | 97.226 | 97.180 | 97.166 | 97.038 | 96.551 | 94.403 | 96.475 | 94.393 | 96.056 | 96.763 | 96.828 | 91.211 | 96.043 | 96.714 | 96.366 | 96.452 | 95.135 | 96.239 | 94.678 | 95.237 | 93.723 | 94.914 | 90.140 | 92.627 | 89.793 | 86.034 | 87.504 | 89.175 | 89.623 | 88.476 | 90.038 | 89.258 | 90.876 |
| 473 | 96.827 | 98.829 | 97.695 | 96.928 | 97.417 | 97.590 | 98.776 | 97.656 | 97.874 | 97.235 | 97.201 | 97.183 | 97.069 | 96.520 | 94.451 | 96.506 | 94.406 | 96.093 | 96.784 | 96.822 | 91.214 | 96.057 | 96.725 | 96.344 | 96.485 | 95.169 | 96.212 | 94.703 | 95.253 | 93.713 | 94.909 | 90.195 | 92.626 | 89.882 | 86.044 | 87.586 | 89.192 | 89.623 | 88.468 | 90.078 | 89.257 | 90.888 |
| 474 | 96.808 | 98.825 | 97.689 | 96.896 | 97.393 | 97.621 | 98.799 | 97.668 | 97.867 | 97.237 | 97.179 | 97.192 | 97.097 | 96.557 | 94.512 | 96.551 | 94.462 | 96.118 | 96.870 | 96.833 | 91.273 | 96.080 | 96.767 | 96.339 | 96.502 | 95.190 | 96.280 | 94.716 | 95.276 | 93.723 | 94.978 | 90.175 | 92.672 | 89.938 | 86.153 | 87.651 | 89.240 | 89.630 | 88.515 | 90.102 | 89.344 | 90.879 |
| 475 | 96.837 | 98.825 | 97.696 | 96.841 | 97.429 | 97.587 | 98.834 | 97.618 | 97.870 | 97.208 | 97.236 | 97.201 | 97.147 | 96.511 | 94.557 | 96.516 | 94.467 | 96.086 | 96.913 | 96.838 | 91.340 | 96.096 | 96.790 | 96.359 | 96.525 | 95.201 | 96.318 | 94.713 | 95.286 | 93.783 | 95.030 | 90.160 | 92.713 | 89.949 | 86.229 | 87.736 | 89.265 | 89.622 | 88.551 | 90.108 | 89.396 | 90.877 |
| 476 | 96.848 | 98.813 | 97.713 | 96.834 | 97.408 | 97.524 | 98.891 | 97.574 | 97.929 | 97.193 | 97.272 | 97.147 | 97.230 | 96.479 | 94.611 | 96.506 | 94.530 | 96.065 | 96.965 | 96.782 | 91.437 | 96.040 | 96.826 | 96.352 | 96.576 | 95.164 | 96.374 | 94.673 | 95.330 | 93.800 | 95.086 | 90.127 | 92.761 | 89.964 | 86.301 | 87.784 | 89.305 | 89.643 | 88.620 | 90.095 | 89.463 | 90.884 |
| 477 | 96.830 | 98.758 | 97.724 | 96.861 | 97.401 | 97.488 | 98.902 | 97.522 | 97.905 | 97.197 | 97.251 | 97.123 | 97.204 | 96.492 | 94.609 | 96.500 | 94.532 | 96.071 | 96.980 | 96.785 | 91.453 | 96.023 | 96.841 | 96.321 | 96.594 | 95.178 | 96.360 | 94.687 | 95.314 | 93.778 | 95.083 | 90.157 | 92.781 | 89.994 | 86.352 | 87.772 | 89.334 | 89.686 | 88.666 | 90.149 | 89.505 | 90.910 |
| 478 | 96.865 | 98.796 | 97.733 | 96.948 | 97.407 | 97.550 | 98.900 | 97.588 | 97.933 | 97.265 | 97.212 | 97.170 | 97.238 | 96.555 | 94.634 | 96.593 | 94.599 | 96.175 | 96.988 | 96.881 | 91.448 | 96.112 | 96.825 | 96.430 | 96.587 | 95.237 | 96.340 | 94.765 | 95.333 | 93.845 | 95.082 | 90.218 | 92.804 | 90.067 | 86.407 | 87.840 | 89.367 | 89.756 | 88.719 | 90.254 | 89.535 | 90.991 |
| 479 | 96.914 | 98.824 | 97.722 | 96.930 | 97.437 | 97.590 | 98.874 | 97.640 | 97.923 | 97.273 | 97.246 | 97.213 | 97.244 | 96.603 | 94.655 | 96.631 | 94.626 | 96.174 | 96.976 | 96.917 | 91.474 | 96.145 | 96.810 | 96.457 | 96.569 | 95.247 | 96.373 | 94.782 | 95.372 | 93.827 | 95.112 | 90.232 | 92.883 | 90.086 | 86.509 | 87.879 | 89.418 | 89.776 | 88.797 | 90.270 | 89.566 | 90.959 |
| 480 | 96.885 | 98.860 | 97.678 | 96.922 | 97.418 | 97.588 | 98.835 | 97.702 | 97.913 | 97.287 | 97.253 | 97.233 | 97.263 | 96.599 | 94.676 | 96.625 | 94.586 | 96.195 | 96.940 | 96.933 | 91.510 | 96.197 | 96.823 | 96.493 | 96.553 | 95.282 | 96.341 | 94.796 | 95.379 | 93.841 | 95.095 | 90.288 | 92.837 | 90.128 | 86.491 | 87.963 | 89.405 | 89.797 | 88.775 | 90.339 | 89.504 | 90.964 |
| 481 | 96.867 | 98.862 | 97.690 | 96.955 | 97.419 | 97.590 | 98.755 | 97.692 | 97.856 | 97.262 | 97.234 | 97.249 | 97.225 | 96.632 | 94.658 | 96.651 | 94.530 | 96.233 | 96.925 | 96.974 | 91.481 | 96.240 | 96.803 | 96.496 | 96.551 | 95.340 | 96.330 | 94.823 | 95.364 | 93.892 | 95.073 | 90.342 | 92.807 | 90.197 | 86.505 | 88.023 | 89.403 | 89.859 | 88.774 | 90.380 | 89.480 | 90.977 |
| 482 | 96.880 | 98.818 | 97.747 | 96.943 | 97.475 | 97.578 | 98.772 | 97.664 | 97.808 | 97.243 | 97.199 | 97.252 | 97.195 | 96.649 | 94.653 | 96.633 | 94.553 | 96.247 | 96.931 | 96.986 | 91.487 | 96.244 | 96.819 | 96.448 | 96.554 | 95.322 | 96.358 | 94.803 | 95.379 | 93.894 | 95.074 | 90.326 | 92.807 | 90.244 | 86.547 | 88.049 | 89.399 | 89.890 | 88.768 | 90.404 | 89.496 | 91.015 |
| 483 | 96.896 | 98.854 | 97.765 | 96.913 | 97.451 | 97.538 | 98.873 | 97.666 | 97.868 | 97.229 | 97.298 | 97.229 | 97.278 | 96.608 | 94.771 | 96.573 | 94.686 | 96.190 | 97.006 | 96.895 | 91.593 | 96.201 | 96.894 | 96.427 | 96.597 | 95.261 | 96.421 | 94.777 | 95.431 | 93.880 | 95.124 | 90.335 | 92.867 | 90.257 | 86.649 | 88.080 | 89.422 | 89.863 | 88.801 | 90.377 | 89.546 | 91.020 |
| 484 | 96.893 | 98.880 | 97.778 | 96.917 | 97.465 | 97.575 | 98.906 | 97.649 | 97.896 | 97.211 | 97.371 | 97.209 | 97.323 | 96.638 | 94.825 | 96.605 | 94.731 | 96.202 | 97.069 | 96.910 | 91.666 | 96.249 | 96.946 | 96.472 | 96.673 | 95.333 | 96.475 | 94.883 | 95.468 | 93.930 | 95.198 | 90.396 | 92.991 | 90.252 | 86.763 | 88.093 | 89.535 | 89.869 | 88.941 | 90.430 | 89.658 | 91.057 |
| 485 | 96.866 | 98.877 | 97.720 | 96.965 | 97.440 | 97.618 | 98.906 | 97.638 | 97.899 | 97.221 | 97.327 | 97.217 | 97.306 | 96.672 | 94.858 | 96.625 | 94.695 | 96.232 | 97.042 | 96.946 | 91.666 | 96.283 | 96.959 | 96.510 | 96.662 | 95.372 | 96.452 | 94.926 | 95.458 | 93.974 | 95.210 | 90.457 | 92.996 | 90.294 | 86.779 | 88.163 | 89.553 | 89.921 | 88.964 | 90.475 | 89.662 | 91.108 |
| 486 | 96.884 | 98.851 | 97.722 | 96.950 | 97.450 | 97.631 | 98.853 | 97.578 | 97.847 | 97.206 | 97.286 | 97.224 | 97.223 | 96.709 | 94.790 | 96.666 | 94.659 | 96.295 | 96.982 | 96.970 | 91.627 | 96.327 | 96.895 | 96.503 | 96.652 | 95.426 | 96.433 | 94.965 | 95.474 | 94.045 | 95.199 | 90.453 | 93.031 | 90.325 | 86.791 | 88.195 | 89.570 | 89.984 | 89.004 | 90.530 | 89.684 | 91.108 |
| 487 | 96.856 | 98.778 | 97.730 | 96.913 | 97.435 | 97.612 | 98.811 | 97.609 | 97.814 | 97.217 | 97.261 | 97.202 | 97.201 | 96.671 | 94.720 | 96.652 | 94.659 | 96.271 | 96.965 | 96.939 | 91.616 | 96.302 | 96.854 | 96.466 | 96.592 | 95.368 | 96.386 | 94.942 | 95.444 | 93.984 | 95.129 | 90.406 | 92.969 | 90.324 | 86.751 | 88.210 | 89.516 | 90.001 | 88.974 | 90.519 | 89.642 | 91.093 |
| 488 | 96.836 | 98.751 | 97.706 | 96.922 | 97.411 | 97.583 | 98.838 | 97.611 | 97.835 | 97.208 | 97.330 | 97.203 | 97.272 | 96.602 | 94.748 | 96.661 | 94.709 | 96.262 | 97.038 | 96.949 | 91.666 | 96.300 | 96.900 | 96.437 | 96.634 | 95.330 | 96.424 | 94.922 | 95.488 | 93.934 | 95.162 | 90.424 | 93.010 | 90.322 | 86.806 | 88.244 | 89.566 | 90.011 | 89.054 | 90.493 | 89.690 | 91.112 |
| 489 | 96.853 | 98.787 | 97.723 | 96.921 | 97.425 | 97.609 | 98.923 | 97.610 | 97.844 | 97.194 | 97.382 | 97.213 | 97.337 | 96.611 | 94.800 | 96.701 | 94.724 | 96.296 | 97.063 | 97.026 | 91.733 | 96.325 | 96.947 | 96.469 | 96.703 | 95.381 | 96.478 | 94.946 | 95.566 | 93.964 | 95.222 | 90.455 | 93.093 | 90.356 | 86.895 | 88.285 | 89.684 | 89.998 | 89.138 | 90.532 | 89.778 | 91.172 |
| 490 | 96.837 | 98.787 | 97.729 | 96.899 | 97.430 | 97.601 | 98.921 | 97.633 | 97.803 | 97.195 | 97.346 | 97.237 | 97.329 | 96.633 | 94.800 | 96.723 | 94.735 | 96.280 | 96.989 | 97.012 | 91.772 | 96.327 | 96.955 | 96.441 | 96.684 | 95.377 | 96.496 | 94.898 | 95.571 | 93.964 | 95.237 | 90.450 | 93.060 | 90.367 | 86.889 | 88.324 | 89.670 | 90.042 | 89.133 | 90.565 | 89.790 | 91.164 |
| 491 | 96.850 | 98.835 | 97.760 | 96.858 | 97.445 | 97.598 | 98.849 | 97.671 | 97.810 | 97.174 | 97.330 | 97.218 | 97.322 | 96.663 | 94.776 | 96.727 | 94.770 | 96.314 | 97.014 | 97.020 | 91.762 | 96.388 | 96.936 | 96.472 | 96.673 | 95.405 | 96.485 | 94.896 | 95.551 | 94.020 | 95.261 | 90.450 | 93.104 | 90.375 | 86.911 | 88.366 | 89.663 | 90.055 | 89.149 | 90.618 | 89.839 | 91.135 |
| 492 | 96.875 | 98.746 | 97.761 | 96.836 | 97.447 | 97.565 | 98.793 | 97.642 | 97.792 | 97.116 | 97.325 | 97.191 | 97.319 | 96.644 | 94.794 | 96.658 | 94.797 | 96.279 | 97.031 | 96.978 | 91.746 | 96.346 | 96.963 | 96.442 | 96.685 | 95.330 | 96.502 | 94.895 | 95.555 | 93.984 | 95.290 | 90.433 | 93.080 | 90.352 | 86.964 | 88.331 | 89.670 | 90.075 | 89.174 | 90.632 | 89.820 | 91.143 |
| 493 | 96.856 | 98.712 | 97.715 | 96.850 | 97.427 | 97.591 | 98.805 | 97.586 | 97.816 | 97.089 | 97.339 | 97.224 | 97.308 | 96.669 | 94.812 | 96.654 | 94.814 | 96.324 | 97.053 | 96.983 | 91.772 | 96.331 | 96.999 | 96.455 | 96.704 | 95.335 | 96.538 | 94.917 | 95.586 | 94.047 | 95.312 | 90.470 | 93.128 | 90.365 | 87.032 | 88.346 | 89.704 | 90.115 | 89.219 | 90.676 | 89.827 | 91.184 |
| 494 | 96.848 | 98.764 | 97.730 | 96.892 | 97.404 | 97.664 | 98.799 | 97.579 | 97.792 | 97.094 | 97.344 | 97.249 | 97.254 | 96.739 | 94.820 | 96.663 | 94.775 | 96.320 | 97.020 | 97.007 | 91.788 | 96.352 | 96.941 | 96.497 | 96.683 | 95.340 | 96.511 | 94.968 | 95.576 | 94.095 | 95.261 | 90.494 | 93.108 | 90.388 | 87.049 | 88.406 | 89.697 | 90.143 | 89.177 | 90.716 | 89.801 | 91.210 |
| 495 | 96.828 | 98.784 | 97.755 | 96.902 | 97.378 | 97.694 | 98.830 | 97.593 | 97.835 | 97.075 | 97.370 | 97.265 | 97.308 | 96.770 | 94.856 | 96.721 | 94.808 | 96.324 | 97.062 | 97.019 | 91.843 | 96.394 | 96.969 | 96.529 | 96.717 | 95.386 | 96.530 | 94.979 | 95.649 | 94.130 | 95.346 | 90.509 | 93.170 | 90.435 | 87.119 | 88.491 | 89.756 | 90.180 | 89.258 | 90.734 | 89.861 | 91.215 |
| 496 | 96.832 | 98.765 | 97.757 | 96.855 | 97.384 | 97.650 | 98.809 | 97.540 | 97.833 | 97.032 | 97.359 | 97.215 | 97.328 | 96.709 | 94.850 | 96.727 | 94.804 | 96.305 | 97.059 | 96.998 | 91.847 | 96.354 | 96.968 | 96.518 | 96.732 | 95.397 | 96.497 | 94.936 | 95.661 | 94.108 | 95.353 | 90.479 | 93.182 | 90.457 | 87.143 | 88.450 | 89.776 | 90.165 | 89.241 | 90.707 | 89.855 | 91.211 |
| 497 | 96.822 | 98.717 | 97.699 | 96.856 | 97.378 | 97.625 | 98.768 | 97.531 | 97.780 | 97.048 | 97.367 | 97.218 | 97.319 | 96.691 | 94.843 | 96.707 | 94.820 | 96.290 | 97.016 | 96.979 | 91.803 | 96.325 | 96.952 | 96.493 | 96.669 | 95.372 | 96.464 | 94.965 | 95.604 | 94.090 | 95.297 | 90.521 | 93.117 | 90.539 | 87.098 | 88.457 | 89.766 | 90.181 | 89.266 | 90.700 | 89.807 | 91.239 |
| 498 | 96.798 | 98.725 | 97.648 | 96.903 | 97.343 | 97.643 | 98.783 | 97.516 | 97.724 | 97.061 | 97.334 | 97.250 | 97.285 | 96.703 | 94.842 | 96.695 | 94.838 | 96.331 | 97.007 | 97.005 | 91.808 | 96.400 | 96.934 | 96.545 | 96.676 | 95.387 | 96.457 | 94.959 | 95.574 | 94.114 | 95.283 | 90.576 | 93.156 | 90.576 | 87.073 | 88.562 | 89.768 | 90.215 | 89.283 | 90.736 | 89.800 | 91.264 |
| 499 | 96.823 | 98.774 | 97.693 | 96.958 | 97.370 | 97.725 | 98.769 | 97.584 | 97.654 | 97.101 | 97.333 | 97.278 | 97.260 | 96.737 | 94.843 | 96.735 | 94.834 | 96.357 | 96.976 | 97.049 | 91.796 | 96.485 | 96.901 | 96.613 | 96.687 | 95.423 | 96.444 | 95.020 | 95.561 | 94.184 | 95.278 | 90.643 | 93.130 | 90.634 | 87.101 | 88.662 | 89.762 | 90.299 | 89.313 | 90.820 | 89.838 | 91.308 |
| 500 | 96.815 | 98.768 | 97.725 | 96.925 | 97.377 | 97.754 | 98.804 | 97.582 | 97.683 | 97.110 | 97.320 | 97.326 | 97.300 | 96.787 | 94.865 | 96.817 | 94.850 | 96.399 | 96.986 | 97.091 | 91.812 | 96.516 | 96.932 | 96.670 | 96.713 | 95.489 | 96.458 | 95.055 | 95.619 | 94.265 | 95.314 | 90.657 | 93.130 | 90.649 | 87.171 | 88.695 | 89.771 | 90.374 | 89.357 | 90.926 | 89.862 | 91.390 |
| 501 | 96.815 | 98.722 | 97.763 | 96.862 | 97.424 | 97.668 | 98.778 | 97.504 | 97.641 | 97.073 | 97.318 | 97.269 | 97.320 | 96.757 | 94.901 | 96.772 | 94.820 | 96.339 | 96.967 | 97.012 | 91.804 | 96.439 | 96.902 | 96.622 | 96.700 | 95.452 | 96.455 | 95.007 | 95.638 | 94.193 | 95.288 | 90.602 | 93.129 | 90.620 | 87.186 | 88.632 | 89.757 | 90.311 | 89.371 | 90.889 | 89.905 | 91.350 |
| 502 | 96.810 | 98.764 | 97.762 | 96.901 | 97.381 | 97.672 | 98.804 | 97.499 | 97.672 | 97.075 | 97.344 | 97.292 | 97.346 | 96.810 | 94.922 | 96.804 | 94.799 | 96.418 | 96.992 | 97.012 | 91.813 | 96.441 | 96.924 | 96.647 | 96.682 | 95.481 | 96.463 | 95.030 | 95.648 | 94.183 | 95.271 | 90.627 | 93.154 | 90.615 | 87.203 | 88.678 | 89.787 | 90.312 | 89.437 | 90.896 | 89.918 | 91.360 |
| 503 | 96.789 | 98.794 | 97.748 | 96.944 | 97.356 | 97.697 | 98.777 | 97.505 | 97.683 | 97.038 | 97.341 | 97.276 | 97.325 | 96.818 | 94.912 | 96.848 | 94.825 | 96.470 | 96.977 | 97.035 | 91.868 | 96.449 | 96.965 | 96.676 | 96.723 | 95.489 | 96.471 | 95.029 | 95.665 | 94.175 | 95.311 | 90.629 | 93.176 | 90.615 | 87.224 | 88.715 | 89.830 | 90.414 | 89.456 | 90.959 | 89.903 | 91.426 |
| 504 | 96.791 | 98.764 | 97.748 | 96.944 | 97.373 | 97.716 | 98.750 | 97.526 | 97.687 | 97.008 | 97.404 | 97.241 | 97.353 | 96.764 | 94.907 | 96.822 | 94.826 | 96.352 | 96.982 | 97.004 | 91.868 | 96.380 | 97.000 | 96.592 | 96.660 | 95.443 | 96.462 | 95.014 | 95.656 | 94.202 | 95.323 | 90.665 | 93.207 | 90.637 | 87.273 | 88.695 | 89.932 | 90.447 | 89.501 | 90.953 | 89.962 | 91.410 |
| 505 | 96.821 | 98.698 | 97.760 | 96.885 | 97.379 | 97.696 | 98.747 | 97.466 | 97.628 | 96.920 | 97.381 | 97.203 | 97.350 | 96.705 | 94.938 | 96.762 | 94.848 | 96.250 | 96.991 | 96.942 | 91.848 | 96.383 | 97.000 | 96.523 | 96.650 | 95.390 | 96.457 | 94.943 | 95.658 | 94.186 | 95.336 | 90.636 | 93.228 | 90.624 | 87.290 | 88.661 | 89.972 | 90.441 | 89.494 | 90.943 | 89.950 | 91.414 |
| 506 | 96.837 | 98.660 | 97.780 | 96.914 | 97.409 | 97.737 | 98.746 | 97.455 | 97.614 | 96.900 | 97.388 | 97.241 | 97.358 | 96.688 | 94.945 | 96.746 | 94.830 | 96.256 | 97.010 | 96.901 | 91.830 | 96.418 | 96.997 | 96.518 | 96.677 | 95.370 | 96.433 | 94.924 | 95.643 | 94.212 | 95.349 | 90.647 | 93.224 | 90.649 | 87.331 | 88.664 | 89.915 | 90.437 | 89.511 | 90.947 | 89.975 | 91.398 |
| 507 | 96.792 | 98.650 | 97.745 | 96.901 | 97.368 | 97.754 | 98.745 | 97.455 | 97.612 | 96.931 | 97.348 | 97.258 | 97.348 | 96.720 | 94.923 | 96.854 | 94.820 | 96.400 | 96.984 | 96.977 | 91.854 | 96.450 | 97.021 | 96.600 | 96.710 | 95.436 | 96.402 | 94.965 | 95.651 | 94.273 | 95.356 | 90.675 | 93.203 | 90.674 | 87.357 | 88.697 | 89.857 | 90.489 | 89.536 | 91.020 | 89.958 | 91.437 |
| 508 | 96.763 | 98.663 | 97.750 | 96.855 | 97.392 | 97.680 | 98.716 | 97.425 | 97.596 | 96.913 | 97.323 | 97.185 | 97.368 | 96.716 | 94.945 | 96.834 | 94.881 | 96.382 | 96.943 | 96.972 | 91.895 | 96.441 | 97.028 | 96.632 | 96.752 | 95.467 | 96.471 | 94.964 | 95.721 | 94.265 | 95.448 | 90.679 | 93.263 | 90.664 | 87.392 | 88.687 | 89.887 | 90.496 | 89.580 | 91.012 | 90.040 | 91.445 |
| 509 | 96.761 | 98.662 | 97.760 | 96.846 | 97.393 | 97.666 | 98.718 | 97.439 | 97.564 | 96.916 | 97.350 | 97.205 | 97.411 | 96.741 | 94.962 | 96.777 | 94.902 | 96.317 | 96.940 | 96.949 | 91.874 | 96.394 | 97.003 | 96.579 | 96.681 | 95.393 | 96.485 | 94.960 | 95.708 | 94.238 | 95.426 | 90.702 | 93.320 | 90.653 | 87.412 | 88.685 | 89.964 | 90.461 | 89.627 | 90.981 | 90.108 | 91.412 |
| 510 | 96.754 | 98.640 | 97.747 | 96.841 | 97.373 | 97.673 | 98.715 | 97.481 | 97.533 | 96.900 | 97.367 | 97.259 | 97.393 | 96.769 | 94.967 | 96.733 | 94.870 | 96.330 | 96.941 | 96.927 | 91.845 | 96.377 | 96.982 | 96.539 | 96.628 | 95.374 | 96.433 | 94.932 | 95.645 | 94.218 | 95.393 | 90.697 | 93.311 | 90.674 | 87.395 | 88.679 | 90.002 | 90.491 | 89.608 | 91.005 | 90.099 | 91.425 |
| 511 | 96.724 | 98.690 | 97.705 | 96.891 | 97.320 | 97.763 | 98.740 | 97.483 | 97.572 | 96.931 | 97.403 | 97.368 | 97.396 | 96.818 | 94.980 | 96.806 | 94.867 | 96.445 | 97.007 | 96.992 | 91.864 | 96.421 | 97.001 | 96.599 | 96.646 | 95.456 | 96.417 | 94.969 | 95.647 | 94.291 | 95.404 | 90.725 | 93.308 | 90.751 | 87.424 | 88.717 | 90.019 | 90.600 | 89.632 | 91.102 | 90.163 | 91.533 |
| 512 | 96.706 | 98.688 | 97.690 | 96.878 | 97.328 | 97.759 | 98.708 | 97.413 | 97.535 | 96.913 | 97.373 | 97.337 | 97.352 | 96.802 | 94.992 | 96.845 | 94.937 | 96.475 | 96.994 | 97.055 | 91.880 | 96.398 | 97.003 | 96.614 | 96.695 | 95.485 | 96.429 | 94.964 | 95.670 | 94.273 | 95.386 | 90.676 | 93.273 | 90.754 | 87.432 | 88.739 | 89.986 | 90.646 | 89.626 | 91.169 | 90.134 | 91.552 |
| 513 | 96.753 | 98.680 | 97.698 | 96.865 | 97.351 | 97.726 | 98.681 | 97.374 | 97.470 | 96.894 | 97.343 | 97.297 | 97.340 | 96.776 | 94.984 | 96.829 | 94.952 | 96.432 | 96.945 | 97.069 | 91.873 | 96.400 | 96.950 | 96.651 | 96.684 | 95.471 | 96.441 | 94.976 | 95.694 | 94.256 | 95.391 | 90.663 | 93.245 | 90.728 | 87.425 | 88.757 | 89.972 | 90.635 | 89.610 | 91.167 | 90.087 | 91.553 |
| 514 | 96.771 | 98.634 | 97.723 | 96.828 | 97.354 | 97.685 | 98.684 | 97.344 | 97.421 | 96.855 | 97.364 | 97.255 | 97.335 | 96.736 | 94.959 | 96.803 | 94.930 | 96.396 | 96.877 | 97.048 | 91.855 | 96.342 | 96.932 | 96.653 | 96.659 | 95.399 | 96.410 | 94.959 | 95.683 | 94.242 | 95.373 | 90.660 | 93.270 | 90.699 | 87.435 | 88.739 | 89.988 | 90.583 | 89.640 | 91.138 | 90.099 | 91.524 |
| 515 | 96.753 | 98.589 | 97.722 | 96.821 | 97.343 | 97.643 | 98.708 | 97.361 | 97.447 | 96.813 | 97.356 | 97.252 | 97.315 | 96.740 | 94.961 | 96.744 | 94.856 | 96.352 | 96.833 | 96.969 | 91.809 | 96.315 | 96.934 | 96.639 | 96.643 | 95.404 | 96.353 | 94.975 | 95.648 | 94.274 | 95.366 | 90.669 | 93.276 | 90.724 | 87.413 | 88.738 | 89.981 | 90.572 | 89.645 | 91.106 | 90.132 | 91.546 |
| 516 | 96.697 | 98.582 | 97.692 | 96.855 | 97.289 | 97.639 | 98.702 | 97.374 | 97.399 | 96.807 | 97.337 | 97.277 | 97.312 | 96.785 | 94.946 | 96.721 | 94.864 | 96.367 | 96.819 | 96.904 | 91.772 | 96.335 | 96.949 | 96.651 | 96.646 | 95.389 | 96.331 | 95.009 | 95.699 | 94.341 | 95.364 | 90.672 | 93.283 | 90.782 | 87.406 | 88.770 | 89.972 | 90.614 | 89.701 | 91.139 | 90.177 | 91.591 |
| 517 | 96.678 | 98.551 | 97.685 | 96.848 | 97.302 | 97.634 | 98.671 | 97.415 | 97.359 | 96.795 | 97.304 | 97.233 | 97.290 | 96.782 | 94.922 | 96.730 | 94.852 | 96.380 | 96.808 | 96.841 | 91.728 | 96.297 | 96.963 | 96.617 | 96.633 | 95.381 | 96.319 | 94.997 | 95.728 | 94.330 | 95.355 | 90.655 | 93.269 | 90.783 | 87.425 | 88.793 | 89.971 | 90.647 | 89.685 | 91.162 | 90.183 | 91.576 |
| 518 | 96.671 | 98.508 | 97.683 | 96.779 | 97.271 | 97.643 | 98.612 | 97.404 | 97.336 | 96.740 | 97.298 | 97.226 | 97.273 | 96.739 | 94.903 | 96.741 | 94.836 | 96.382 | 96.803 | 96.811 | 91.679 | 96.214 | 96.961 | 96.596 | 96.596 | 95.371 | 96.299 | 94.922 | 95.718 | 94.291 | 95.359 | 90.661 | 93.247 | 90.771 | 87.461 | 88.762 | 89.978 | 90.632 | 89.675 | 91.157 | 90.199 | 91.574 |
| 519 | 96.643 | 98.497 | 97.682 | 96.777 | 97.257 | 97.676 | 98.526 | 97.360 | 97.298 | 96.691 | 97.307 | 97.255 | 97.270 | 96.721 | 94.915 | 96.769 | 94.820 | 96.370 | 96.759 | 96.833 | 91.653 | 96.159 | 96.943 | 96.588 | 96.558 | 95.355 | 96.281 | 94.868 | 95.678 | 94.270 | 95.375 | 90.635 | 93.243 | 90.751 | 87.483 | 88.757 | 89.989 | 90.637 | 89.695 | 91.172 | 90.209 | 91.600 |
| 520 | 96.667 | 98.503 | 97.654 | 96.785 | 97.232 | 97.681 | 98.494 | 97.306 | 97.271 | 96.660 | 97.306 | 97.266 | 97.314 | 96.761 | 94.925 | 96.817 | 94.849 | 96.347 | 96.703 | 96.829 | 91.642 | 96.190 | 96.944 | 96.560 | 96.515 | 95.341 | 96.291 | 94.872 | 95.661 | 94.270 | 95.381 | 90.626 | 93.256 | 90.754 | 87.490 | 88.784 | 89.996 | 90.667 | 89.719 | 91.192 | 90.198 | 91.628 |
| 521 | 96.665 | 98.497 | 97.614 | 96.796 | 97.215 | 97.655 | 98.525 | 97.241 | 97.256 | 96.671 | 97.295 | 97.240 | 97.333 | 96.761 | 94.934 | 96.842 | 94.887 | 96.349 | 96.661 | 96.825 | 91.639 | 96.188 | 96.944 | 96.552 | 96.524 | 95.330 | 96.294 | 94.853 | 95.678 | 94.283 | 95.355 | 90.639 | 93.285 | 90.784 | 87.495 | 88.803 | 90.031 | 90.733 | 89.749 | 91.221 | 90.213 | 91.645 |
| 522 | 96.658 | 98.503 | 97.581 | 96.774 | 97.187 | 97.676 | 98.591 | 97.233 | 97.281 | 96.674 | 97.308 | 97.237 | 97.339 | 96.768 | 94.970 | 96.850 | 94.912 | 96.367 | 96.645 | 96.828 | 91.614 | 96.151 | 96.940 | 96.571 | 96.558 | 95.369 | 96.283 | 94.821 | 95.704 | 94.287 | 95.309 | 90.657 | 93.300 | 90.812 | 87.499 | 88.816 | 90.072 | 90.781 | 89.749 | 91.249 | 90.253 | 91.671 |
| 523 | 96.602 | 98.447 | 97.578 | 96.744 | 97.175 | 97.696 | 98.561 | 97.249 | 97.229 | 96.632 | 97.320 | 97.247 | 97.310 | 96.744 | 94.967 | 96.791 | 94.887 | 96.334 | 96.606 | 96.787 | 91.541 | 96.068 | 96.922 | 96.580 | 96.520 | 95.391 | 96.248 | 94.820 | 95.699 | 94.294 | 95.299 | 90.653 | 93.288 | 90.810 | 87.480 | 88.791 | 90.086 | 90.763 | 89.757 | 91.268 | 90.266 | 91.674 |
| 524 | 96.584 | 98.435 | 97.568 | 96.769 | 97.206 | 97.711 | 98.504 | 97.257 | 97.162 | 96.614 | 97.323 | 97.257 | 97.289 | 96.741 | 94.971 | 96.790 | 94.843 | 96.301 | 96.535 | 96.776 | 91.508 | 96.060 | 96.895 | 96.579 | 96.471 | 95.397 | 96.230 | 94.878 | 95.682 | 94.328 | 95.322 | 90.660 | 93.258 | 90.820 | 87.485 | 88.822 | 90.068 | 90.767 | 89.770 | 91.304 | 90.262 | 91.652 |
| 525 | 96.566 | 98.426 | 97.572 | 96.778 | 97.184 | 97.677 | 98.488 | 97.183 | 97.131 | 96.591 | 97.324 | 97.252 | 97.280 | 96.735 | 94.976 | 96.788 | 94.848 | 96.276 | 96.498 | 96.738 | 91.497 | 96.037 | 96.910 | 96.561 | 96.452 | 95.330 | 96.208 | 94.846 | 95.672 | 94.315 | 95.363 | 90.668 | 93.226 | 90.821 | 87.483 | 88.845 | 90.055 | 90.783 | 89.796 | 91.287 | 90.296 | 91.611 |
| 526 | 96.529 | 98.379 | 97.587 | 96.713 | 97.121 | 97.600 | 98.502 | 97.121 | 97.113 | 96.512 | 97.305 | 97.209 | 97.281 | 96.686 | 95.009 | 96.733 | 94.840 | 96.240 | 96.500 | 96.687 | 91.497 | 95.933 | 96.900 | 96.498 | 96.473 | 95.234 | 96.212 | 94.742 | 95.697 | 94.264 | 95.382 | 90.637 | 93.251 | 90.799 | 87.487 | 88.798 | 90.121 | 90.791 | 89.802 | 91.237 | 90.352 | 91.606 |
| 527 | 96.561 | 98.376 | 97.611 | 96.692 | 97.072 | 97.625 | 98.482 | 97.130 | 97.065 | 96.459 | 97.302 | 97.216 | 97.279 | 96.715 | 95.013 | 96.724 | 94.858 | 96.276 | 96.504 | 96.678 | 91.444 | 95.876 | 96.883 | 96.463 | 96.482 | 95.228 | 96.199 | 94.725 | 95.691 | 94.267 | 95.370 | 90.659 | 93.291 | 90.804 | 87.495 | 88.793 | 90.189 | 90.817 | 89.812 | 91.236 | 90.371 | 91.675 |
| 528 | 96.577 | 98.338 | 97.623 | 96.702 | 97.117 | 97.642 | 98.441 | 97.156 | 97.037 | 96.435 | 97.329 | 97.210 | 97.258 | 96.729 | 94.985 | 96.709 | 94.850 | 96.272 | 96.517 | 96.659 | 91.366 | 95.818 | 96.868 | 96.442 | 96.458 | 95.236 | 96.219 | 94.764 | 95.713 | 94.332 | 95.415 | 90.685 | 93.305 | 90.819 | 87.526 | 88.814 | 90.223 | 90.814 | 89.829 | 91.265 | 90.361 | 91.736 |
| 529 | 96.570 | 98.379 | 97.586 | 96.724 | 97.142 | 97.664 | 98.395 | 97.124 | 97.015 | 96.434 | 97.357 | 97.229 | 97.248 | 96.764 | 95.010 | 96.733 | 94.851 | 96.279 | 96.502 | 96.669 | 91.319 | 95.768 | 96.881 | 96.500 | 96.462 | 95.273 | 96.235 | 94.807 | 95.752 | 94.361 | 95.448 | 90.680 | 93.286 | 90.854 | 87.551 | 88.832 | 90.214 | 90.816 | 89.852 | 91.296 | 90.361 | 91.748 |
| 530 | 96.541 | 98.388 | 97.600 | 96.731 | 97.131 | 97.664 | 98.400 | 97.105 | 97.016 | 96.387 | 97.348 | 97.236 | 97.233 | 96.766 | 95.016 | 96.759 | 94.864 | 96.282 | 96.497 | 96.652 | 91.283 | 95.754 | 96.902 | 96.596 | 96.453 | 95.306 | 96.215 | 94.774 | 95.797 | 94.365 | 95.446 | 90.658 | 93.260 | 90.873 | 87.514 | 88.807 | 90.222 | 90.816 | 89.870 | 91.311 | 90.403 | 91.736 |
| 531 | 96.528 | 98.336 | 97.586 | 96.681 | 97.088 | 97.625 | 98.408 | 97.053 | 96.960 | 96.284 | 97.305 | 97.208 | 97.224 | 96.728 | 94.985 | 96.743 | 94.865 | 96.241 | 96.446 | 96.582 | 91.236 | 95.689 | 96.893 | 96.583 | 96.436 | 95.304 | 96.165 | 94.715 | 95.783 | 94.323 | 95.389 | 90.610 | 93.244 | 90.868 | 87.451 | 88.751 | 90.243 | 90.810 | 89.858 | 91.306 | 90.404 | 91.743 |
| 532 | 96.562 | 98.275 | 97.588 | 96.655 | 97.100 | 97.606 | 98.440 | 96.912 | 96.969 | 96.236 | 97.284 | 97.202 | 97.238 | 96.716 | 94.969 | 96.729 | 94.859 | 96.248 | 96.382 | 96.547 | 91.220 | 95.636 | 96.849 | 96.530 | 96.423 | 95.243 | 96.121 | 94.680 | 95.727 | 94.268 | 95.374 | 90.616 | 93.229 | 90.841 | 87.450 | 88.740 | 90.284 | 90.835 | 89.894 | 91.283 | 90.417 | 91.773 |
| 533 | 96.530 | 98.227 | 97.556 | 96.623 | 97.076 | 97.554 | 98.414 | 96.816 | 96.930 | 96.169 | 97.255 | 97.149 | 97.192 | 96.729 | 94.947 | 96.727 | 94.823 | 96.205 | 96.317 | 96.475 | 91.133 | 95.530 | 96.806 | 96.425 | 96.373 | 95.173 | 96.059 | 94.632 | 95.672 | 94.248 | 95.367 | 90.597 | 93.164 | 90.814 | 87.440 | 88.685 | 90.296 | 90.836 | 89.858 | 91.260 | 90.411 | 91.768 |
| 534 | 96.501 | 98.232 | 97.548 | 96.599 | 97.026 | 97.557 | 98.333 | 96.776 | 96.857 | 96.162 | 97.239 | 97.173 | 97.157 | 96.745 | 94.927 | 96.738 | 94.789 | 96.163 | 96.247 | 96.425 | 91.031 | 95.460 | 96.786 | 96.371 | 96.338 | 95.130 | 95.995 | 94.582 | 95.612 | 94.235 | 95.320 | 90.583 | 93.147 | 90.802 | 87.392 | 88.686 | 90.284 | 90.836 | 89.806 | 91.253 | 90.392 | 91.753 |
| 535 | 96.502 | 98.278 | 97.562 | 96.642 | 97.051 | 97.607 | 98.266 | 96.893 | 96.781 | 96.172 | 97.263 | 97.242 | 97.128 | 96.775 | 94.934 | 96.750 | 94.828 | 96.178 | 96.233 | 96.435 | 91.003 | 95.472 | 96.795 | 96.413 | 96.305 | 95.166 | 95.987 | 94.563 | 95.656 | 94.295 | 95.298 | 90.648 | 93.174 | 90.832 | 87.370 | 88.760 | 90.307 | 90.889 | 89.829 | 91.324 | 90.406 | 91.775 |
| 536 | 96.506 | 98.212 | 97.556 | 96.657 | 97.085 | 97.582 | 98.231 | 96.933 | 96.707 | 96.102 | 97.311 | 97.227 | 97.128 | 96.715 | 94.928 | 96.676 | 94.845 | 96.155 | 96.184 | 96.369 | 90.992 | 95.378 | 96.800 | 96.371 | 96.271 | 95.098 | 95.993 | 94.544 | 95.693 | 94.285 | 95.278 | 90.679 | 93.204 | 90.836 | 87.390 | 88.750 | 90.349 | 90.885 | 89.895 | 91.332 | 90.451 | 91.796 |
| 537 | 96.490 | 98.156 | 97.546 | 96.638 | 97.071 | 97.544 | 98.252 | 96.873 | 96.683 | 95.988 | 97.289 | 97.187 | 97.098 | 96.648 | 94.922 | 96.629 | 94.804 | 96.193 | 96.135 | 96.296 | 90.943 | 95.249 | 96.768 | 96.353 | 96.258 | 95.048 | 95.995 | 94.532 | 95.727 | 94.280 | 95.292 | 90.657 | 93.202 | 90.825 | 87.401 | 88.678 | 90.372 | 90.860 | 89.922 | 91.308 | 90.472 | 91.780 |
| 538 | 96.440 | 98.147 | 97.503 | 96.582 | 96.970 | 97.483 | 98.243 | 96.793 | 96.595 | 95.870 | 97.201 | 97.104 | 97.041 | 96.616 | 94.888 | 96.620 | 94.727 | 96.155 | 96.036 | 96.206 | 90.774 | 95.128 | 96.705 | 96.345 | 96.222 | 95.031 | 95.942 | 94.472 | 95.711 | 94.259 | 95.277 | 90.587 | 93.131 | 90.790 | 87.360 | 88.568 | 90.305 | 90.852 | 89.862 | 91.295 | 90.457 | 91.793 |
| 539 | 96.432 | 98.185 | 97.514 | 96.547 | 96.903 | 97.494 | 98.284 | 96.744 | 96.590 | 95.852 | 97.198 | 97.121 | 97.082 | 96.616 | 94.898 | 96.632 | 94.731 | 96.184 | 96.008 | 96.180 | 90.736 | 95.074 | 96.716 | 96.391 | 96.250 | 95.052 | 95.954 | 94.448 | 95.715 | 94.268 | 95.321 | 90.570 | 93.164 | 90.788 | 87.341 | 88.582 | 90.333 | 90.918 | 89.898 | 91.301 | 90.494 | 91.870 |
| 540 | 96.422 | 98.208 | 97.516 | 96.564 | 96.873 | 97.482 | 98.263 | 96.780 | 96.605 | 95.870 | 97.202 | 97.150 | 97.119 | 96.678 | 94.930 | 96.651 | 94.755 | 96.168 | 96.013 | 96.173 | 90.701 | 95.010 | 96.719 | 96.404 | 96.240 | 95.083 | 95.928 | 94.466 | 95.684 | 94.269 | 95.315 | 90.567 | 93.183 | 90.805 | 87.330 | 88.575 | 90.364 | 90.989 | 89.937 | 91.301 | 90.513 | 91.921 |
| 541 | 96.430 | 98.155 | 97.512 | 96.582 | 96.881 | 97.531 | 98.198 | 96.765 | 96.569 | 95.865 | 97.199 | 97.168 | 97.124 | 96.722 | 94.904 | 96.670 | 94.756 | 96.119 | 95.964 | 96.155 | 90.589 | 94.924 | 96.649 | 96.332 | 96.181 | 95.064 | 95.847 | 94.452 | 95.602 | 94.259 | 95.239 | 90.597 | 93.119 | 90.804 | 87.279 | 88.595 | 90.365 | 91.018 | 89.905 | 91.287 | 90.509 | 91.924 |
| 542 | 96.397 | 98.078 | 97.483 | 96.594 | 96.922 | 97.543 | 98.117 | 96.748 | 96.500 | 95.798 | 97.162 | 97.146 | 97.046 | 96.708 | 94.877 | 96.641 | 94.708 | 96.079 | 95.886 | 96.101 | 90.425 | 94.786 | 96.520 | 96.227 | 96.124 | 94.997 | 95.762 | 94.399 | 95.562 | 94.264 | 95.239 | 90.588 | 93.068 | 90.788 | 87.234 | 88.572 | 90.359 | 90.983 | 89.862 | 91.267 | 90.494 | 91.851 |
| 543 | 96.376 | 98.019 | 97.460 | 96.565 | 96.940 | 97.514 | 98.080 | 96.691 | 96.447 | 95.687 | 97.153 | 97.102 | 97.006 | 96.627 | 94.877 | 96.564 | 94.709 | 96.018 | 95.756 | 95.982 | 90.338 | 94.713 | 96.485 | 96.168 | 96.112 | 94.919 | 95.774 | 94.333 | 95.621 | 94.222 | 95.272 | 90.547 | 93.060 | 90.759 | 87.223 | 88.485 | 90.363 | 90.948 | 89.868 | 91.238 | 90.527 | 91.836 |
| 544 | 96.370 | 98.047 | 97.469 | 96.524 | 96.958 | 97.510 | 98.122 | 96.626 | 96.418 | 95.599 | 97.204 | 97.064 | 97.003 | 96.558 | 94.901 | 96.536 | 94.707 | 96.016 | 95.731 | 95.903 | 90.371 | 94.650 | 96.575 | 96.169 | 96.129 | 94.923 | 95.829 | 94.293 | 95.700 | 94.209 | 95.329 | 90.526 | 93.112 | 90.770 | 87.240 | 88.420 | 90.426 | 90.970 | 89.933 | 91.245 | 90.563 | 91.842 |
| 545 | 96.368 | 98.100 | 97.442 | 96.488 | 96.883 | 97.513 | 98.101 | 96.597 | 96.364 | 95.590 | 97.204 | 97.101 | 97.028 | 96.612 | 94.903 | 96.574 | 94.649 | 96.028 | 95.697 | 95.915 | 90.308 | 94.592 | 96.564 | 96.215 | 96.065 | 94.953 | 95.781 | 94.310 | 95.660 | 94.206 | 95.278 | 90.544 | 93.029 | 90.764 | 87.176 | 88.360 | 90.377 | 91.020 | 89.883 | 91.266 | 90.538 | 91.895 |
| 546 | 96.364 | 98.097 | 97.435 | 96.480 | 96.839 | 97.584 | 98.045 | 96.571 | 96.269 | 95.540 | 97.201 | 97.137 | 97.017 | 96.679 | 94.844 | 96.623 | 94.582 | 96.037 | 95.649 | 95.907 | 90.181 | 94.485 | 96.515 | 96.238 | 96.004 | 94.929 | 95.674 | 94.280 | 95.573 | 94.208 | 95.208 | 90.561 | 92.962 | 90.768 | 87.103 | 88.381 | 90.365 | 91.039 | 89.844 | 91.281 | 90.548 | 91.926 |
| 547 | 96.383 | 98.026 | 97.476 | 96.501 | 96.864 | 97.569 | 98.012 | 96.545 | 96.256 | 95.467 | 97.201 | 97.132 | 97.002 | 96.686 | 94.825 | 96.570 | 94.611 | 95.971 | 95.624 | 95.789 | 90.041 | 94.308 | 96.456 | 96.183 | 96.003 | 94.846 | 95.663 | 94.248 | 95.609 | 94.171 | 95.253 | 90.552 | 92.993 | 90.761 | 87.130 | 88.338 | 90.382 | 90.944 | 89.891 | 91.241 | 90.595 | 91.896 |
| 548 | 96.408 | 98.037 | 97.519 | 96.518 | 96.878 | 97.569 | 98.043 | 96.503 | 96.245 | 95.414 | 97.227 | 97.117 | 96.998 | 96.656 | 94.886 | 96.519 | 94.713 | 95.922 | 95.576 | 95.662 | 89.965 | 94.223 | 96.481 | 96.136 | 96.070 | 94.838 | 95.757 | 94.200 | 95.737 | 94.150 | 95.292 | 90.544 | 93.040 | 90.750 | 87.161 | 88.300 | 90.448 | 90.892 | 89.935 | 91.205 | 90.677 | 91.915 |
| 549 | 96.367 | 98.034 | 97.483 | 96.504 | 96.834 | 97.504 | 97.967 | 96.430 | 96.174 | 95.340 | 97.128 | 97.044 | 96.949 | 96.616 | 94.833 | 96.509 | 94.671 | 95.879 | 95.507 | 95.603 | 89.841 | 94.103 | 96.433 | 96.091 | 95.984 | 94.823 | 95.706 | 94.138 | 95.690 | 94.120 | 95.218 | 90.499 | 92.986 | 90.695 | 87.097 | 88.242 | 90.418 | 90.879 | 89.882 | 91.153 | 90.629 | 91.907 |
| 550 | 96.304 | 98.035 | 97.415 | 96.495 | 96.763 | 97.507 | 97.861 | 96.428 | 96.079 | 95.312 | 96.996 | 97.086 | 96.915 | 96.678 | 94.775 | 96.609 | 94.581 | 95.951 | 95.386 | 95.613 | 89.660 | 94.023 | 96.300 | 96.104 | 95.841 | 94.840 | 95.551 | 94.138 | 95.558 | 94.151 | 95.078 | 90.510 | 92.847 | 90.702 | 86.942 | 88.251 | 90.282 | 90.925 | 89.721 | 91.175 | 90.521 | 91.958 |
| 551 | 96.273 | 97.982 | 97.409 | 96.502 | 96.769 | 97.543 | 97.800 | 96.422 | 95.988 | 95.270 | 96.989 | 97.072 | 96.878 | 96.680 | 94.720 | 96.607 | 94.485 | 95.940 | 95.315 | 95.557 | 89.516 | 93.850 | 96.223 | 96.030 | 95.759 | 94.785 | 95.430 | 94.084 | 95.451 | 94.151 | 95.009 | 90.527 | 92.823 | 90.692 | 86.910 | 88.290 | 90.260 | 90.937 | 89.677 | 91.173 | 90.505 | 91.933 |
| 552 | 96.285 | 97.900 | 97.397 | 96.473 | 96.775 | 97.539 | 97.782 | 96.386 | 95.939 | 95.196 | 97.078 | 97.009 | 96.891 | 96.608 | 94.739 | 96.521 | 94.534 | 95.861 | 95.314 | 95.390 | 89.456 | 93.653 | 96.189 | 95.931 | 95.792 | 94.707 | 95.447 | 94.035 | 95.517 | 94.142 | 95.076 | 90.546 | 92.871 | 90.648 | 86.983 | 88.246 | 90.290 | 90.866 | 89.720 | 91.141 | 90.561 | 91.869 |
| 553 | 96.256 | 97.897 | 97.418 | 96.461 | 96.778 | 97.499 | 97.844 | 96.328 | 95.961 | 95.102 | 97.131 | 96.980 | 96.916 | 96.543 | 94.795 | 96.473 | 94.624 | 95.827 | 95.301 | 95.274 | 89.417 | 93.539 | 96.250 | 95.922 | 95.863 | 94.717 | 95.528 | 94.045 | 95.627 | 94.177 | 95.175 | 90.520 | 92.923 | 90.592 | 87.030 | 88.220 | 90.373 | 90.865 | 89.760 | 91.127 | 90.647 | 91.863 |
| 554 | 96.219 | 97.927 | 97.430 | 96.420 | 96.757 | 97.433 | 97.807 | 96.307 | 95.925 | 95.026 | 97.086 | 97.017 | 96.893 | 96.580 | 94.762 | 96.496 | 94.554 | 95.855 | 95.228 | 95.255 | 89.268 | 93.455 | 96.188 | 95.969 | 95.799 | 94.726 | 95.469 | 94.071 | 95.545 | 94.198 | 95.110 | 90.485 | 92.834 | 90.598 | 86.915 | 88.213 | 90.291 | 90.891 | 89.660 | 91.152 | 90.586 | 91.934 |
| 555 | 96.242 | 97.934 | 97.445 | 96.400 | 96.764 | 97.454 | 97.813 | 96.293 | 95.858 | 94.998 | 97.065 | 97.040 | 96.900 | 96.609 | 94.766 | 96.512 | 94.519 | 95.896 | 95.126 | 95.232 | 89.117 | 93.369 | 96.120 | 95.932 | 95.721 | 94.704 | 95.395 | 94.042 | 95.439 | 94.165 | 95.053 | 90.483 | 92.784 | 90.655 | 86.843 | 88.215 | 90.260 | 90.877 | 89.588 | 91.143 | 90.582 | 91.974 |
| 556 | 96.273 | 97.833 | 97.468 | 96.369 | 96.787 | 97.459 | 97.762 | 96.271 | 95.786 | 94.960 | 97.066 | 96.965 | 96.896 | 96.568 | 94.757 | 96.451 | 94.523 | 95.807 | 95.031 | 95.128 | 89.014 | 93.199 | 96.104 | 95.804 | 95.718 | 94.632 | 95.409 | 93.976 | 95.427 | 94.107 | 95.084 | 90.455 | 92.822 | 90.649 | 86.902 | 88.169 | 90.302 | 90.813 | 89.635 | 91.075 | 90.630 | 91.942 |
| 557 | 96.275 | 97.790 | 97.411 | 96.341 | 96.755 | 97.440 | 97.739 | 96.243 | 95.749 | 94.912 | 97.046 | 96.905 | 96.886 | 96.519 | 94.743 | 96.400 | 94.544 | 95.782 | 94.973 | 95.063 | 88.935 | 93.089 | 96.098 | 95.741 | 95.736 | 94.627 | 95.427 | 93.963 | 95.517 | 94.100 | 95.110 | 90.451 | 92.838 | 90.626 | 86.948 | 88.107 | 90.320 | 90.778 | 89.662 | 91.038 | 90.629 | 91.935 |
| 558 | 96.218 | 97.757 | 97.390 | 96.308 | 96.683 | 97.384 | 97.720 | 96.161 | 95.735 | 94.809 | 96.984 | 96.888 | 96.858 | 96.468 | 94.719 | 96.390 | 94.510 | 95.767 | 94.921 | 95.008 | 88.811 | 92.944 | 96.056 | 95.723 | 95.696 | 94.606 | 95.380 | 93.897 | 95.534 | 94.072 | 95.061 | 90.406 | 92.769 | 90.538 | 86.926 | 88.017 | 90.295 | 90.776 | 89.584 | 90.980 | 90.590 | 91.907 |
| 559 | 96.180 | 97.740 | 97.356 | 96.312 | 96.631 | 97.339 | 97.676 | 96.123 | 95.667 | 94.763 | 96.958 | 96.928 | 96.849 | 96.483 | 94.654 | 96.404 | 94.471 | 95.767 | 94.875 | 94.932 | 88.653 | 92.795 | 95.910 | 95.680 | 95.621 | 94.554 | 95.273 | 93.800 | 95.431 | 94.050 | 94.949 | 90.348 | 92.708 | 90.537 | 86.773 | 87.984 | 90.196 | 90.763 | 89.494 | 90.910 | 90.556 | 91.861 |
| 560 | 96.185 | 97.703 | 97.383 | 96.346 | 96.648 | 97.378 | 97.687 | 96.110 | 95.648 | 94.763 | 97.016 | 96.925 | 96.923 | 96.507 | 94.633 | 96.391 | 94.544 | 95.737 | 94.856 | 94.849 | 88.566 | 92.636 | 95.904 | 95.629 | 95.630 | 94.535 | 95.307 | 93.773 | 95.430 | 94.063 | 95.000 | 90.357 | 92.808 | 90.569 | 86.748 | 88.001 | 90.260 | 90.717 | 89.519 | 90.855 | 90.660 | 91.807 |
| 561 | 96.130 | 97.709 | 97.356 | 96.370 | 96.652 | 97.386 | 97.616 | 96.128 | 95.589 | 94.759 | 97.016 | 96.874 | 96.878 | 96.466 | 94.646 | 96.358 | 94.563 | 95.718 | 94.766 | 94.781 | 88.499 | 92.527 | 95.906 | 95.620 | 95.645 | 94.538 | 95.335 | 93.809 | 95.439 | 94.097 | 95.039 | 90.378 | 92.809 | 90.592 | 86.733 | 87.981 | 90.238 | 90.722 | 89.503 | 90.872 | 90.682 | 91.793 |
| 562 | 96.074 | 97.747 | 97.320 | 96.363 | 96.622 | 97.429 | 97.611 | 96.087 | 95.564 | 94.707 | 97.004 | 96.860 | 96.870 | 96.435 | 94.653 | 96.378 | 94.502 | 95.750 | 94.695 | 94.787 | 88.399 | 92.453 | 95.881 | 95.631 | 95.617 | 94.563 | 95.266 | 93.798 | 95.422 | 94.098 | 94.974 | 90.368 | 92.768 | 90.561 | 86.673 | 87.955 | 90.212 | 90.751 | 89.456 | 90.892 | 90.664 | 91.824 |
| 563 | 96.042 | 97.684 | 97.326 | 96.320 | 96.588 | 97.421 | 97.578 | 96.021 | 95.512 | 94.611 | 96.981 | 96.886 | 96.864 | 96.409 | 94.675 | 96.408 | 94.466 | 95.695 | 94.642 | 94.722 | 88.260 | 92.326 | 95.784 | 95.570 | 95.559 | 94.487 | 95.210 | 93.738 | 95.416 | 94.057 | 94.916 | 90.357 | 92.708 | 90.508 | 86.652 | 87.912 | 90.188 | 90.742 | 89.424 | 90.818 | 90.655 | 91.763 |
| 564 | 96.056 | 97.617 | 97.304 | 96.258 | 96.554 | 97.343 | 97.585 | 95.968 | 95.475 | 94.525 | 96.990 | 96.866 | 96.833 | 96.357 | 94.644 | 96.320 | 94.470 | 95.629 | 94.581 | 94.616 | 88.147 | 92.188 | 95.765 | 95.482 | 95.569 | 94.438 | 95.237 | 93.716 | 95.417 | 94.013 | 94.925 | 90.327 | 92.682 | 90.515 | 86.657 | 87.853 | 90.196 | 90.692 | 89.433 | 90.743 | 90.663 | 91.732 |
| 565 | 96.077 | 97.585 | 97.307 | 96.246 | 96.524 | 97.355 | 97.576 | 95.917 | 95.431 | 94.466 | 97.006 | 96.865 | 96.805 | 96.401 | 94.639 | 96.264 | 94.446 | 95.602 | 94.536 | 94.548 | 88.060 | 92.099 | 95.754 | 95.471 | 95.571 | 94.430 | 95.261 | 93.697 | 95.403 | 94.014 | 94.931 | 90.330 | 92.614 | 90.530 | 86.641 | 87.839 | 90.173 | 90.682 | 89.393 | 90.716 | 90.676 | 91.732 |
| 566 | 96.045 | 97.586 | 97.312 | 96.292 | 96.486 | 97.400 | 97.490 | 95.919 | 95.337 | 94.444 | 96.955 | 96.859 | 96.774 | 96.468 | 94.605 | 96.265 | 94.373 | 95.641 | 94.433 | 94.543 | 87.963 | 92.035 | 95.703 | 95.469 | 95.511 | 94.415 | 95.162 | 93.705 | 95.331 | 94.068 | 94.858 | 90.324 | 92.540 | 90.563 | 86.550 | 87.836 | 90.096 | 90.722 | 89.352 | 90.737 | 90.672 | 91.745 |
| 567 | 96.006 | 97.587 | 97.312 | 96.324 | 96.461 | 97.440 | 97.457 | 95.913 | 95.293 | 94.430 | 96.975 | 96.851 | 96.815 | 96.504 | 94.585 | 96.300 | 94.369 | 95.651 | 94.374 | 94.537 | 87.907 | 91.990 | 95.707 | 95.468 | 95.481 | 94.403 | 95.123 | 93.691 | 95.308 | 94.110 | 94.865 | 90.310 | 92.611 | 90.558 | 86.538 | 87.837 | 90.098 | 90.744 | 89.375 | 90.749 | 90.704 | 91.783 |
| 568 | 96.015 | 97.599 | 97.324 | 96.338 | 96.454 | 97.439 | 97.420 | 95.898 | 95.223 | 94.388 | 96.897 | 96.855 | 96.796 | 96.474 | 94.533 | 96.351 | 94.353 | 95.646 | 94.316 | 94.500 | 87.846 | 91.966 | 95.669 | 95.464 | 95.441 | 94.399 | 95.057 | 93.685 | 95.276 | 94.110 | 94.844 | 90.358 | 92.566 | 90.542 | 86.494 | 87.859 | 90.066 | 90.771 | 89.335 | 90.768 | 90.679 | 91.841 |
| 569 | 95.996 | 97.526 | 97.320 | 96.253 | 96.463 | 97.373 | 97.422 | 95.793 | 95.156 | 94.287 | 96.824 | 96.803 | 96.789 | 96.372 | 94.493 | 96.312 | 94.301 | 95.557 | 94.274 | 94.407 | 87.766 | 91.828 | 95.630 | 95.390 | 95.373 | 94.333 | 95.023 | 93.595 | 95.257 | 94.065 | 94.807 | 90.303 | 92.546 | 90.447 | 86.412 | 87.789 | 90.025 | 90.694 | 89.274 | 90.695 | 90.637 | 91.799 |
| 570 | 95.996 | 97.460 | 97.318 | 96.241 | 96.464 | 97.368 | 97.378 | 95.702 | 95.094 | 94.225 | 96.775 | 96.771 | 96.734 | 96.350 | 94.504 | 96.284 | 94.250 | 95.540 | 94.249 | 94.358 | 87.684 | 91.741 | 95.594 | 95.354 | 95.297 | 94.288 | 94.981 | 93.532 | 95.232 | 94.030 | 94.749 | 90.304 | 92.486 | 90.423 | 86.331 | 87.733 | 90.003 | 90.674 | 89.209 | 90.636 | 90.609 | 91.755 |
| 571 | 95.940 | 97.439 | 97.260 | 96.218 | 96.411 | 97.339 | 97.317 | 95.678 | 95.058 | 94.150 | 96.813 | 96.746 | 96.754 | 96.369 | 94.524 | 96.221 | 94.298 | 95.515 | 94.207 | 94.316 | 87.644 | 91.677 | 95.647 | 95.324 | 95.320 | 94.254 | 94.942 | 93.485 | 95.220 | 93.988 | 94.716 | 90.321 | 92.514 | 90.417 | 86.291 | 87.664 | 90.035 | 90.663 | 89.208 | 90.595 | 90.675 | 91.743 |
| 572 | 95.932 | 97.454 | 97.212 | 96.211 | 96.399 | 97.335 | 97.286 | 95.663 | 95.062 | 94.097 | 96.863 | 96.780 | 96.782 | 96.400 | 94.561 | 96.204 | 94.356 | 95.494 | 94.215 | 94.276 | 87.607 | 91.635 | 95.659 | 95.336 | 95.398 | 94.243 | 94.954 | 93.464 | 95.267 | 93.966 | 94.781 | 90.312 | 92.540 | 90.424 | 86.305 | 87.643 | 90.069 | 90.694 | 89.250 | 90.614 | 90.762 | 91.823 |
| 573 | 95.958 | 97.473 | 97.243 | 96.206 | 96.381 | 97.346 | 97.244 | 95.655 | 95.017 | 94.072 | 96.799 | 96.780 | 96.743 | 96.400 | 94.527 | 96.243 | 94.301 | 95.515 | 94.204 | 94.299 | 87.586 | 91.643 | 95.605 | 95.388 | 95.353 | 94.258 | 94.879 | 93.463 | 95.227 | 93.941 | 94.747 | 90.314 | 92.467 | 90.413 | 86.213 | 87.641 | 90.003 | 90.740 | 89.201 | 90.613 | 90.724 | 91.872 |
| 574 | 95.908 | 97.397 | 97.243 | 96.196 | 96.379 | 97.293 | 97.229 | 95.595 | 94.940 | 94.001 | 96.794 | 96.750 | 96.718 | 96.343 | 94.500 | 96.226 | 94.220 | 95.465 | 94.162 | 94.268 | 87.559 | 91.581 | 95.582 | 95.361 | 95.258 | 94.168 | 94.826 | 93.377 | 95.151 | 93.893 | 94.707 | 90.228 | 92.452 | 90.373 | 86.152 | 87.576 | 90.007 | 90.689 | 89.186 | 90.521 | 90.725 | 91.860 |
| 575 | 95.832 | 97.353 | 97.203 | 96.191 | 96.345 | 97.270 | 97.230 | 95.529 | 94.806 | 93.920 | 96.787 | 96.700 | 96.690 | 96.291 | 94.505 | 96.203 | 94.149 | 95.405 | 94.143 | 94.216 | 87.502 | 91.553 | 95.585 | 95.352 | 95.218 | 94.123 | 94.829 | 93.359 | 95.138 | 93.860 | 94.648 | 90.200 | 92.410 | 90.333 | 86.111 | 87.515 | 90.057 | 90.666 | 89.151 | 90.490 | 90.733 | 91.908 |
| 576 | 95.796 | 97.386 | 97.141 | 96.156 | 96.294 | 97.258 | 97.256 | 95.491 | 94.757 | 93.812 | 96.776 | 96.736 | 96.747 | 96.343 | 94.497 | 96.200 | 94.116 | 95.391 | 94.162 | 94.222 | 87.475 | 91.542 | 95.573 | 95.342 | 95.181 | 94.099 | 94.802 | 93.320 | 95.117 | 93.829 | 94.604 | 90.154 | 92.368 | 90.304 | 86.049 | 87.478 | 90.045 | 90.685 | 89.131 | 90.477 | 90.746 | 91.928 |
| 577 | 95.811 | 97.332 | 97.124 | 96.167 | 96.287 | 97.255 | 97.202 | 95.454 | 94.699 | 93.715 | 96.760 | 96.720 | 96.744 | 96.328 | 94.459 | 96.145 | 94.111 | 95.306 | 94.158 | 94.154 | 87.432 | 91.468 | 95.551 | 95.297 | 95.099 | 94.017 | 94.761 | 93.260 | 95.129 | 93.786 | 94.628 | 90.089 | 92.371 | 90.299 | 86.025 | 87.444 | 90.022 | 90.696 | 89.173 | 90.450 | 90.741 | 91.874 |
| 578 | 95.833 | 97.309 | 97.149 | 96.128 | 96.287 | 97.214 | 97.130 | 95.364 | 94.649 | 93.579 | 96.708 | 96.636 | 96.700 | 96.274 | 94.383 | 96.079 | 94.083 | 95.225 | 94.124 | 94.131 | 87.432 | 91.437 | 95.577 | 95.279 | 95.054 | 93.960 | 94.747 | 93.168 | 95.135 | 93.760 | 94.588 | 90.037 | 92.332 | 90.223 | 85.970 | 87.335 | 89.980 | 90.711 | 89.121 | 90.411 | 90.703 | 91.820 |
| 579 | 95.840 | 97.296 | 97.169 | 96.163 | 96.337 | 97.272 | 97.091 | 95.326 | 94.602 | 93.553 | 96.724 | 96.713 | 96.692 | 96.314 | 94.371 | 96.116 | 94.046 | 95.245 | 94.095 | 94.199 | 87.397 | 91.447 | 95.544 | 95.310 | 95.013 | 93.948 | 94.697 | 93.153 | 95.077 | 93.790 | 94.544 | 90.052 | 92.305 | 90.240 | 85.919 | 87.305 | 90.006 | 90.743 | 89.065 | 90.427 | 90.717 | 91.873 |
| 580 | 95.846 | 97.256 | 97.172 | 96.147 | 96.343 | 97.324 | 97.087 | 95.286 | 94.564 | 93.528 | 96.732 | 96.706 | 96.672 | 96.301 | 94.398 | 96.055 | 94.039 | 95.171 | 94.078 | 94.154 | 87.400 | 91.372 | 95.524 | 95.299 | 95.038 | 93.926 | 94.723 | 93.144 | 95.112 | 93.785 | 94.596 | 90.069 | 92.317 | 90.222 | 85.926 | 87.262 | 90.066 | 90.703 | 89.076 | 90.415 | 90.752 | 91.895 |
| 581 | 95.838 | 97.265 | 97.150 | 96.117 | 96.290 | 97.296 | 97.075 | 95.240 | 94.505 | 93.450 | 96.681 | 96.673 | 96.643 | 96.276 | 94.379 | 95.996 | 93.982 | 95.160 | 94.078 | 94.159 | 87.406 | 91.346 | 95.590 | 95.336 | 95.005 | 93.899 | 94.642 | 93.086 | 95.030 | 93.751 | 94.539 | 90.031 | 92.204 | 90.173 | 85.817 | 87.181 | 90.027 | 90.723 | 89.063 | 90.425 | 90.719 | 91.943 |
| 582 | 95.783 | 97.224 | 97.160 | 96.105 | 96.280 | 97.291 | 96.998 | 95.233 | 94.394 | 93.392 | 96.652 | 96.654 | 96.630 | 96.265 | 94.356 | 95.999 | 93.899 | 95.115 | 94.049 | 94.136 | 87.361 | 91.320 | 95.635 | 95.292 | 94.910 | 93.796 | 94.526 | 92.989 | 94.979 | 93.705 | 94.469 | 90.021 | 92.172 | 90.149 | 85.714 | 87.109 | 89.998 | 90.746 | 89.099 | 90.390 | 90.749 | 91.916 |
| 583 | 95.793 | 97.256 | 97.136 | 96.102 | 96.258 | 97.285 | 96.937 | 95.219 | 94.366 | 93.314 | 96.658 | 96.642 | 96.668 | 96.257 | 94.338 | 95.979 | 93.909 | 95.087 | 94.040 | 94.157 | 87.340 | 91.358 | 95.655 | 95.309 | 94.918 | 93.788 | 94.504 | 92.966 | 95.007 | 93.721 | 94.448 | 90.032 | 92.148 | 90.106 | 85.691 | 87.062 | 90.012 | 90.763 | 89.097 | 90.403 | 90.771 | 91.964 |
| 584 | 95.790 | 97.285 | 97.134 | 96.165 | 96.257 | 97.315 | 96.868 | 95.203 | 94.320 | 93.255 | 96.648 | 96.706 | 96.666 | 96.295 | 94.359 | 96.003 | 93.860 | 95.099 | 94.038 | 94.223 | 87.295 | 91.403 | 95.602 | 95.375 | 94.826 | 93.777 | 94.394 | 92.925 | 94.931 | 93.742 | 94.377 | 90.009 | 92.124 | 90.105 | 85.626 | 87.075 | 90.000 | 90.791 | 89.059 | 90.414 | 90.767 | 92.004 |
| 585 | 95.826 | 97.182 | 97.164 | 96.147 | 96.260 | 97.256 | 96.852 | 95.137 | 94.254 | 93.135 | 96.656 | 96.643 | 96.671 | 96.204 | 94.364 | 95.890 | 93.857 | 94.966 | 94.046 | 94.141 | 87.350 | 91.288 | 95.583 | 95.330 | 94.836 | 93.707 | 94.418 | 92.838 | 94.979 | 93.698 | 94.437 | 89.938 | 92.152 | 90.013 | 85.638 | 86.994 | 90.048 | 90.718 | 89.105 | 90.361 | 90.793 | 91.947 |
| 586 | 95.838 | 97.189 | 97.147 | 96.136 | 96.240 | 97.209 | 96.816 | 95.074 | 94.156 | 93.049 | 96.615 | 96.635 | 96.634 | 96.208 | 94.319 | 95.879 | 93.794 | 94.958 | 94.047 | 94.124 | 87.393 | 91.292 | 95.591 | 95.345 | 94.759 | 93.688 | 94.340 | 92.779 | 94.914 | 93.678 | 94.364 | 89.915 | 92.048 | 89.925 | 85.485 | 86.931 | 89.999 | 90.779 | 89.076 | 90.390 | 90.769 | 91.973 |
| 587 | 95.836 | 97.167 | 97.172 | 96.129 | 96.282 | 97.217 | 96.787 | 95.018 | 94.055 | 92.981 | 96.615 | 96.639 | 96.631 | 96.248 | 94.269 | 95.918 | 93.759 | 94.924 | 94.035 | 94.068 | 87.376 | 91.212 | 95.543 | 95.269 | 94.668 | 93.581 | 94.273 | 92.698 | 94.829 | 93.617 | 94.343 | 89.880 | 92.018 | 89.872 | 85.421 | 86.865 | 90.039 | 90.749 | 89.090 | 90.316 | 90.825 | 91.921 |
| 588 | 95.765 | 97.164 | 97.116 | 96.132 | 96.246 | 97.231 | 96.737 | 94.972 | 93.995 | 92.884 | 96.588 | 96.571 | 96.611 | 96.203 | 94.186 | 95.870 | 93.749 | 94.889 | 94.026 | 94.049 | 87.350 | 91.198 | 95.578 | 95.269 | 94.623 | 93.517 | 94.258 | 92.666 | 94.813 | 93.586 | 94.318 | 89.865 | 91.961 | 89.816 | 85.356 | 86.777 | 90.057 | 90.727 | 89.051 | 90.287 | 90.813 | 91.907 |
| 589 | 95.744 | 97.163 | 97.129 | 96.130 | 96.256 | 97.229 | 96.670 | 94.935 | 93.901 | 92.849 | 96.548 | 96.589 | 96.577 | 96.211 | 94.156 | 95.881 | 93.651 | 94.864 | 93.996 | 94.117 | 87.273 | 91.222 | 95.576 | 95.302 | 94.511 | 93.453 | 94.139 | 92.585 | 94.748 | 93.558 | 94.232 | 89.832 | 91.861 | 89.841 | 85.256 | 86.718 | 90.021 | 90.745 | 88.995 | 90.286 | 90.777 | 91.847 |
| 590 | 95.738 | 97.126 | 97.119 | 96.141 | 96.250 | 97.221 | 96.611 | 94.917 | 93.832 | 92.835 | 96.578 | 96.631 | 96.587 | 96.222 | 94.131 | 95.808 | 93.630 | 94.799 | 93.934 | 94.096 | 87.247 | 91.221 | 95.566 | 95.323 | 94.491 | 93.433 | 94.129 | 92.557 | 94.791 | 93.549 | 94.248 | 89.825 | 91.841 | 89.849 | 85.229 | 86.676 | 90.028 | 90.740 | 89.023 | 90.294 | 90.795 | 91.823 |
| 591 | 95.742 | 97.182 | 97.139 | 96.181 | 96.267 | 97.213 | 96.594 | 94.866 | 93.849 | 92.784 | 96.553 | 96.657 | 96.593 | 96.254 | 94.162 | 95.785 | 93.618 | 94.844 | 93.913 | 94.108 | 87.264 | 91.270 | 95.553 | 95.351 | 94.442 | 93.421 | 94.051 | 92.530 | 94.734 | 93.539 | 94.199 | 89.790 | 91.790 | 89.798 | 85.153 | 86.648 | 89.977 | 90.797 | 89.012 | 90.364 | 90.818 | 91.905 |
| 592 | 95.781 | 97.153 | 97.175 | 96.195 | 96.310 | 97.236 | 96.664 | 94.812 | 93.843 | 92.735 | 96.591 | 96.669 | 96.647 | 96.263 | 94.181 | 95.752 | 93.647 | 94.800 | 93.942 | 94.056 | 87.265 | 91.212 | 95.585 | 95.299 | 94.468 | 93.375 | 94.089 | 92.453 | 94.762 | 93.489 | 94.253 | 89.744 | 91.835 | 89.748 | 85.181 | 86.602 | 90.059 | 90.743 | 89.111 | 90.333 | 90.910 | 91.937 |
| 593 | 95.815 | 97.182 | 97.175 | 96.191 | 96.290 | 97.270 | 96.686 | 94.810 | 93.856 | 92.687 | 96.566 | 96.630 | 96.661 | 96.253 | 94.174 | 95.764 | 93.622 | 94.805 | 93.993 | 94.060 | 87.285 | 91.224 | 95.661 | 95.327 | 94.493 | 93.398 | 94.078 | 92.421 | 94.784 | 93.483 | 94.209 | 89.745 | 91.823 | 89.703 | 85.122 | 86.524 | 90.044 | 90.749 | 89.116 | 90.359 | 90.895 | 92.005 |
| 594 | 95.808 | 97.142 | 97.169 | 96.164 | 96.265 | 97.287 | 96.649 | 94.756 | 93.790 | 92.606 | 96.596 | 96.633 | 96.689 | 96.258 | 94.167 | 95.796 | 93.569 | 94.745 | 94.009 | 94.026 | 87.238 | 91.122 | 95.624 | 95.273 | 94.401 | 93.317 | 93.991 | 92.361 | 94.733 | 93.460 | 94.128 | 89.703 | 91.760 | 89.631 | 85.022 | 86.437 | 90.027 | 90.694 | 89.085 | 90.257 | 90.887 | 91.973 |
| 595 | 95.783 | 97.061 | 97.137 | 96.128 | 96.269 | 97.256 | 96.603 | 94.684 | 93.711 | 92.515 | 96.612 | 96.599 | 96.691 | 96.207 | 94.179 | 95.719 | 93.537 | 94.688 | 93.987 | 93.982 | 87.231 | 91.055 | 95.587 | 95.258 | 94.357 | 93.254 | 93.962 | 92.326 | 94.697 | 93.423 | 94.111 | 89.666 | 91.700 | 89.539 | 84.952 | 86.349 | 90.004 | 90.648 | 89.031 | 90.177 | 90.867 | 91.933 |
| 596 | 95.758 | 97.062 | 97.127 | 96.154 | 96.307 | 97.258 | 96.567 | 94.677 | 93.650 | 92.494 | 96.623 | 96.641 | 96.684 | 96.243 | 94.194 | 95.753 | 93.483 | 94.696 | 93.958 | 94.036 | 87.186 | 91.081 | 95.550 | 95.284 | 94.275 | 93.223 | 93.901 | 92.285 | 94.622 | 93.391 | 94.028 | 89.667 | 91.645 | 89.552 | 84.896 | 86.363 | 89.954 | 90.662 | 88.983 | 90.195 | 90.862 | 91.941 |
| 597 | 95.757 | 97.071 | 97.163 | 96.160 | 96.276 | 97.237 | 96.488 | 94.662 | 93.542 | 92.492 | 96.625 | 96.635 | 96.642 | 96.234 | 94.131 | 95.718 | 93.483 | 94.654 | 93.889 | 93.988 | 87.137 | 91.061 | 95.541 | 95.273 | 94.235 | 93.212 | 93.895 | 92.260 | 94.598 | 93.375 | 93.986 | 89.674 | 91.630 | 89.571 | 84.859 | 86.365 | 89.957 | 90.633 | 88.969 | 90.203 | 90.827 | 91.923 |
| 598 | 95.786 | 97.146 | 97.196 | 96.198 | 96.282 | 97.230 | 96.494 | 94.655 | 93.548 | 92.459 | 96.661 | 96.627 | 96.694 | 96.248 | 94.157 | 95.742 | 93.531 | 94.663 | 93.912 | 93.981 | 87.171 | 91.059 | 95.559 | 95.268 | 94.240 | 93.199 | 93.835 | 92.248 | 94.577 | 93.384 | 94.003 | 89.651 | 91.645 | 89.578 | 84.858 | 86.366 | 89.956 | 90.647 | 88.973 | 90.204 | 90.863 | 91.919 |
| 599 | 95.778 | 97.186 | 97.167 | 96.212 | 96.255 | 97.288 | 96.535 | 94.677 | 93.575 | 92.443 | 96.673 | 96.660 | 96.732 | 96.283 | 94.131 | 95.758 | 93.543 | 94.625 | 93.896 | 93.968 | 87.158 | 91.036 | 95.586 | 95.264 | 94.243 | 93.204 | 93.809 | 92.262 | 94.573 | 93.430 | 94.017 | 89.682 | 91.629 | 89.555 | 84.844 | 86.350 | 89.969 | 90.672 | 88.948 | 90.224 | 90.887 | 91.945 |
| 600 | 95.798 | 97.191 | 97.147 | 96.225 | 96.283 | 97.267 | 96.533 | 94.682 | 93.560 | 92.406 | 96.635 | 96.678 | 96.702 | 96.273 | 94.095 | 95.739 | 93.489 | 94.645 | 93.816 | 93.943 | 87.084 | 91.015 | 95.576 | 95.273 | 94.215 | 93.168 | 93.789 | 92.268 | 94.528 | 93.404 | 93.995 | 89.642 | 91.589 | 89.552 | 84.803 | 86.333 | 89.946 | 90.644 | 88.939 | 90.242 | 90.851 | 91.908 |
| 601 | 95.782 | 97.164 | 97.123 | 96.248 | 96.256 | 97.245 | 96.543 | 94.692 | 93.570 | 92.379 | 96.654 | 96.693 | 96.752 | 96.270 | 94.097 | 95.699 | 93.456 | 94.604 | 93.788 | 93.920 | 87.032 | 90.968 | 95.588 | 95.274 | 94.207 | 93.098 | 93.767 | 92.256 | 94.496 | 93.352 | 93.957 | 89.602 | 91.593 | 89.544 | 84.744 | 86.304 | 89.939 | 90.629 | 88.926 | 90.220 | 90.846 | 91.825 |
| 602 | 95.796 | 97.142 | 97.128 | 96.264 | 96.264 | 97.243 | 96.565 | 94.648 | 93.579 | 92.399 | 96.682 | 96.701 | 96.811 | 96.269 | 94.096 | 95.668 | 93.460 | 94.582 | 93.814 | 93.890 | 87.010 | 90.919 | 95.575 | 95.293 | 94.231 | 93.065 | 93.765 | 92.257 | 94.507 | 93.358 | 94.001 | 89.573 | 91.621 | 89.537 | 84.741 | 86.286 | 89.974 | 90.592 | 88.915 | 90.193 | 90.862 | 91.708 |
| 603 | 95.802 | 97.158 | 97.119 | 96.274 | 96.272 | 97.248 | 96.544 | 94.632 | 93.580 | 92.428 | 96.674 | 96.713 | 96.854 | 96.342 | 94.099 | 95.687 | 93.424 | 94.600 | 93.837 | 93.934 | 87.054 | 90.929 | 95.537 | 95.364 | 94.212 | 93.119 | 93.730 | 92.199 | 94.494 | 93.365 | 94.007 | 89.558 | 91.605 | 89.534 | 84.730 | 86.269 | 89.944 | 90.630 | 88.881 | 90.183 | 90.900 | 91.660 |
| 604 | 95.809 | 97.093 | 97.117 | 96.227 | 96.275 | 97.249 | 96.500 | 94.609 | 93.535 | 92.402 | 96.683 | 96.692 | 96.823 | 96.316 | 94.084 | 95.698 | 93.452 | 94.566 | 93.800 | 93.851 | 87.030 | 90.869 | 95.515 | 95.337 | 94.186 | 93.111 | 93.741 | 92.162 | 94.508 | 93.353 | 93.999 | 89.575 | 91.628 | 89.500 | 84.742 | 86.248 | 89.931 | 90.552 | 88.842 | 90.153 | 90.909 | 91.645 |
| 605 | 95.792 | 97.129 | 97.122 | 96.241 | 96.262 | 97.269 | 96.445 | 94.614 | 93.482 | 92.398 | 96.654 | 96.698 | 96.767 | 96.355 | 94.090 | 95.759 | 93.384 | 94.619 | 93.721 | 93.853 | 86.951 | 90.873 | 95.461 | 95.390 | 94.075 | 93.130 | 93.667 | 92.174 | 94.429 | 93.359 | 93.886 | 89.584 | 91.568 | 89.509 | 84.694 | 86.289 | 89.864 | 90.528 | 88.821 | 90.156 | 90.869 | 91.636 |
| 606 | 95.754 | 97.166 | 97.106 | 96.268 | 96.207 | 97.301 | 96.479 | 94.641 | 93.495 | 92.448 | 96.691 | 96.694 | 96.791 | 96.339 | 94.106 | 95.729 | 93.430 | 94.582 | 93.671 | 93.857 | 86.937 | 90.864 | 95.479 | 95.363 | 94.091 | 93.129 | 93.698 | 92.197 | 94.478 | 93.372 | 93.881 | 89.630 | 91.584 | 89.511 | 84.736 | 86.281 | 89.885 | 90.553 | 88.813 | 90.175 | 90.876 | 91.668 |
| 607 | 95.773 | 97.183 | 97.149 | 96.291 | 96.216 | 97.315 | 96.509 | 94.638 | 93.524 | 92.455 | 96.714 | 96.732 | 96.796 | 96.364 | 94.096 | 95.714 | 93.421 | 94.598 | 93.633 | 93.853 | 86.855 | 90.821 | 95.436 | 95.298 | 94.039 | 93.086 | 93.645 | 92.184 | 94.434 | 93.345 | 93.892 | 89.616 | 91.581 | 89.533 | 84.785 | 86.270 | 89.889 | 90.511 | 88.796 | 90.164 | 90.888 | 91.631 |
| 608 | 95.813 | 97.157 | 97.187 | 96.295 | 96.228 | 97.333 | 96.507 | 94.650 | 93.557 | 92.480 | 96.697 | 96.723 | 96.791 | 96.386 | 94.130 | 95.718 | 93.434 | 94.627 | 93.650 | 93.852 | 86.875 | 90.765 | 95.425 | 95.271 | 94.073 | 93.114 | 93.634 | 92.148 | 94.423 | 93.361 | 93.903 | 89.595 | 91.575 | 89.549 | 84.785 | 86.277 | 89.834 | 90.546 | 88.751 | 90.157 | 90.874 | 91.655 |
| 609 | 95.837 | 97.138 | 97.182 | 96.272 | 96.268 | 97.347 | 96.523 | 94.639 | 93.586 | 92.441 | 96.754 | 96.752 | 96.820 | 96.398 | 94.180 | 95.726 | 93.490 | 94.574 | 93.672 | 93.790 | 86.830 | 90.648 | 95.451 | 95.231 | 94.126 | 93.127 | 93.659 | 92.166 | 94.462 | 93.362 | 93.948 | 89.621 | 91.633 | 89.520 | 84.845 | 86.312 | 89.875 | 90.491 | 88.762 | 90.124 | 90.901 | 91.650 |
| 610 | 95.798 | 97.096 | 97.142 | 96.261 | 96.247 | 97.327 | 96.489 | 94.612 | 93.594 | 92.424 | 96.788 | 96.724 | 96.858 | 96.397 | 94.197 | 95.654 | 93.460 | 94.539 | 93.679 | 93.757 | 86.833 | 90.596 | 95.492 | 95.253 | 94.171 | 93.138 | 93.692 | 92.134 | 94.511 | 93.312 | 93.940 | 89.593 | 91.641 | 89.489 | 84.890 | 86.309 | 89.891 | 90.459 | 88.753 | 90.098 | 90.916 | 91.604 |
| 611 | 95.768 | 97.075 | 97.124 | 96.258 | 96.243 | 97.313 | 96.590 | 94.618 | 93.660 | 92.418 | 96.901 | 96.694 | 96.958 | 96.406 | 94.255 | 95.655 | 93.513 | 94.562 | 93.713 | 93.722 | 86.860 | 90.560 | 95.539 | 95.259 | 94.226 | 93.112 | 93.798 | 92.127 | 94.600 | 93.284 | 94.007 | 89.593 | 91.716 | 89.497 | 84.952 | 86.311 | 89.962 | 90.433 | 88.823 | 90.091 | 90.976 | 91.616 |
| 612 | 95.796 | 97.126 | 97.114 | 96.308 | 96.212 | 97.305 | 96.637 | 94.652 | 93.664 | 92.479 | 96.893 | 96.708 | 96.946 | 96.419 | 94.267 | 95.695 | 93.514 | 94.653 | 93.732 | 93.742 | 86.871 | 90.596 | 95.592 | 95.342 | 94.254 | 93.156 | 93.846 | 92.166 | 94.570 | 93.340 | 93.997 | 89.606 | 91.715 | 89.547 | 84.937 | 86.323 | 89.938 | 90.494 | 88.828 | 90.166 | 90.990 | 91.676 |
| 613 | 95.812 | 97.164 | 97.125 | 96.371 | 96.247 | 97.334 | 96.636 | 94.715 | 93.663 | 92.572 | 96.859 | 96.795 | 96.913 | 96.466 | 94.295 | 95.771 | 93.519 | 94.687 | 93.713 | 93.771 | 86.850 | 90.567 | 95.595 | 95.353 | 94.259 | 93.189 | 93.838 | 92.209 | 94.599 | 93.430 | 94.005 | 89.679 | 91.694 | 89.637 | 84.966 | 86.401 | 89.874 | 90.534 | 88.817 | 90.197 | 90.992 | 91.698 |
| 614 | 95.815 | 97.146 | 97.127 | 96.313 | 96.244 | 97.344 | 96.636 | 94.684 | 93.694 | 92.522 | 96.861 | 96.825 | 96.947 | 96.486 | 94.331 | 95.764 | 93.558 | 94.622 | 93.660 | 93.727 | 86.786 | 90.508 | 95.601 | 95.334 | 94.220 | 93.130 | 93.750 | 92.173 | 94.565 | 93.378 | 94.003 | 89.674 | 91.707 | 89.627 | 84.996 | 86.407 | 89.885 | 90.488 | 88.795 | 90.131 | 91.019 | 91.642 |
| 615 | 95.764 | 97.105 | 97.131 | 96.278 | 96.223 | 97.338 | 96.598 | 94.710 | 93.660 | 92.533 | 96.804 | 96.795 | 96.922 | 96.466 | 94.296 | 95.794 | 93.488 | 94.662 | 93.609 | 93.730 | 86.723 | 90.538 | 95.569 | 95.345 | 94.171 | 93.103 | 93.694 | 92.181 | 94.521 | 93.358 | 93.936 | 89.621 | 91.641 | 89.620 | 84.954 | 86.404 | 89.829 | 90.492 | 88.696 | 90.145 | 90.954 | 91.646 |
| 616 | 95.788 | 97.125 | 97.121 | 96.310 | 96.205 | 97.333 | 96.581 | 94.728 | 93.645 | 92.553 | 96.836 | 96.782 | 96.903 | 96.421 | 94.256 | 95.775 | 93.526 | 94.661 | 93.570 | 93.734 | 86.737 | 90.583 | 95.609 | 95.384 | 94.178 | 93.146 | 93.728 | 92.252 | 94.519 | 93.364 | 93.966 | 89.629 | 91.666 | 89.594 | 84.971 | 86.408 | 89.852 | 90.539 | 88.688 | 90.191 | 90.946 | 91.710 |
| 617 | 95.782 | 97.075 | 97.119 | 96.286 | 96.196 | 97.267 | 96.542 | 94.680 | 93.618 | 92.533 | 96.787 | 96.752 | 96.865 | 96.394 | 94.201 | 95.763 | 93.517 | 94.661 | 93.551 | 93.698 | 86.726 | 90.572 | 95.637 | 95.373 | 94.130 | 93.124 | 93.734 | 92.209 | 94.478 | 93.348 | 93.922 | 89.612 | 91.632 | 89.553 | 84.948 | 86.393 | 89.834 | 90.502 | 88.672 | 90.135 | 90.898 | 91.668 |
| 618 | 95.786 | 97.087 | 97.143 | 96.261 | 96.213 | 97.266 | 96.565 | 94.702 | 93.651 | 92.536 | 96.811 | 96.787 | 96.919 | 96.438 | 94.227 | 95.778 | 93.497 | 94.698 | 93.560 | 93.704 | 86.706 | 90.586 | 95.623 | 95.415 | 94.101 | 93.134 | 93.727 | 92.171 | 94.428 | 93.343 | 93.887 | 89.663 | 91.630 | 89.577 | 84.973 | 86.427 | 89.821 | 90.485 | 88.674 | 90.123 | 90.894 | 91.660 |
| 619 | 95.724 | 97.089 | 97.110 | 96.220 | 96.144 | 97.268 | 96.564 | 94.709 | 93.619 | 92.507 | 96.808 | 96.768 | 96.936 | 96.428 | 94.269 | 95.730 | 93.478 | 94.653 | 93.574 | 93.694 | 86.732 | 90.570 | 95.669 | 95.453 | 94.132 | 93.119 | 93.727 | 92.120 | 94.421 | 93.371 | 93.872 | 89.683 | 91.672 | 89.601 | 84.990 | 86.402 | 89.826 | 90.486 | 88.656 | 90.144 | 90.933 | 91.683 |
| 620 | 95.685 | 97.084 | 97.080 | 96.259 | 96.154 | 97.306 | 96.549 | 94.752 | 93.572 | 92.517 | 96.823 | 96.779 | 96.899 | 96.466 | 94.274 | 95.741 | 93.471 | 94.615 | 93.573 | 93.712 | 86.743 | 90.557 | 95.685 | 95.483 | 94.148 | 93.109 | 93.729 | 92.155 | 94.429 | 93.396 | 93.846 | 89.711 | 91.661 | 89.632 | 84.996 | 86.426 | 89.804 | 90.471 | 88.634 | 90.174 | 90.935 | 91.700 |
| 621 | 95.774 | 97.088 | 97.110 | 96.257 | 96.222 | 97.308 | 96.513 | 94.781 | 93.600 | 92.501 | 96.834 | 96.793 | 96.899 | 96.479 | 94.287 | 95.747 | 93.505 | 94.609 | 93.615 | 93.715 | 86.792 | 90.593 | 95.726 | 95.521 | 94.142 | 93.101 | 93.696 | 92.166 | 94.489 | 93.399 | 93.880 | 89.723 | 91.702 | 89.666 | 85.025 | 86.438 | 89.844 | 90.510 | 88.654 | 90.129 | 90.992 | 91.704 |
| 622 | 95.797 | 97.041 | 97.120 | 96.231 | 96.243 | 97.283 | 96.577 | 94.805 | 93.697 | 92.437 | 96.852 | 96.787 | 96.949 | 96.512 | 94.342 | 95.740 | 93.528 | 94.621 | 93.721 | 93.735 | 86.835 | 90.635 | 95.798 | 95.566 | 94.173 | 93.083 | 93.725 | 92.157 | 94.532 | 93.390 | 93.934 | 89.709 | 91.739 | 89.627 | 85.078 | 86.458 | 89.914 | 90.492 | 88.724 | 90.098 | 91.074 | 91.664 |
| 623 | 95.802 | 97.039 | 97.131 | 96.270 | 96.234 | 97.252 | 96.626 | 94.809 | 93.740 | 92.415 | 96.932 | 96.813 | 96.990 | 96.493 | 94.424 | 95.730 | 93.558 | 94.679 | 93.776 | 93.761 | 86.872 | 90.688 | 95.860 | 95.596 | 94.169 | 93.101 | 93.758 | 92.171 | 94.517 | 93.368 | 93.990 | 89.684 | 91.779 | 89.618 | 85.136 | 86.478 | 89.941 | 90.514 | 88.785 | 90.186 | 91.172 | 91.675 |
| 624 | 95.741 | 97.092 | 97.119 | 96.286 | 96.210 | 97.283 | 96.607 | 94.808 | 93.694 | 92.407 | 96.914 | 96.807 | 96.972 | 96.485 | 94.434 | 95.751 | 93.541 | 94.674 | 93.733 | 93.750 | 86.924 | 90.696 | 95.905 | 95.644 | 94.175 | 93.135 | 93.770 | 92.187 | 94.504 | 93.368 | 93.977 | 89.681 | 91.755 | 89.616 | 85.121 | 86.508 | 89.947 | 90.543 | 88.801 | 90.230 | 91.151 | 91.709 |
| 625 | 95.774 | 97.104 | 97.127 | 96.272 | 96.243 | 97.282 | 96.610 | 94.801 | 93.668 | 92.401 | 96.932 | 96.846 | 96.987 | 96.432 | 94.420 | 95.767 | 93.528 | 94.653 | 93.726 | 93.760 | 86.958 | 90.728 | 95.922 | 95.643 | 94.157 | 93.100 | 93.763 | 92.161 | 94.475 | 93.347 | 93.945 | 89.674 | 91.733 | 89.599 | 85.093 | 86.559 | 89.930 | 90.555 | 88.791 | 90.252 | 91.101 | 91.672 |
| 626 | 95.837 | 97.046 | 97.141 | 96.229 | 96.254 | 97.268 | 96.560 | 94.787 | 93.640 | 92.337 | 96.927 | 96.818 | 96.994 | 96.374 | 94.392 | 95.732 | 93.487 | 94.583 | 93.713 | 93.756 | 86.979 | 90.757 | 95.937 | 95.587 | 94.133 | 93.051 | 93.714 | 92.078 | 94.474 | 93.370 | 93.928 | 89.645 | 91.721 | 89.612 | 85.078 | 86.540 | 89.910 | 90.546 | 88.768 | 90.189 | 91.067 | 91.596 |
| 627 | 95.857 | 97.040 | 97.147 | 96.235 | 96.233 | 97.299 | 96.548 | 94.771 | 93.620 | 92.281 | 96.901 | 96.839 | 96.959 | 96.413 | 94.318 | 95.719 | 93.472 | 94.585 | 93.723 | 93.825 | 87.014 | 90.827 | 95.966 | 95.624 | 94.104 | 93.024 | 93.707 | 92.053 | 94.433 | 93.368 | 93.884 | 89.622 | 91.687 | 89.622 | 85.079 | 86.508 | 89.883 | 90.580 | 88.766 | 90.192 | 91.048 | 91.607 |
| 628 | 95.888 | 97.098 | 97.171 | 96.254 | 96.277 | 97.310 | 96.500 | 94.734 | 93.556 | 92.266 | 96.878 | 96.845 | 96.945 | 96.442 | 94.292 | 95.723 | 93.470 | 94.602 | 93.774 | 93.859 | 87.049 | 90.881 | 95.971 | 95.706 | 94.034 | 93.047 | 93.681 | 92.067 | 94.411 | 93.360 | 93.865 | 89.614 | 91.649 | 89.642 | 85.079 | 86.446 | 89.884 | 90.603 | 88.756 | 90.215 | 91.035 | 91.671 |
| 629 | 95.885 | 97.157 | 97.145 | 96.289 | 96.276 | 97.360 | 96.511 | 94.762 | 93.530 | 92.283 | 96.897 | 96.839 | 96.948 | 96.523 | 94.292 | 95.735 | 93.487 | 94.612 | 93.833 | 93.912 | 87.144 | 90.965 | 96.026 | 95.829 | 94.069 | 93.077 | 93.658 | 92.054 | 94.415 | 93.346 | 93.906 | 89.672 | 91.657 | 89.630 | 85.103 | 86.408 | 89.918 | 90.610 | 88.780 | 90.278 | 91.039 | 91.725 |
| 630 | 95.905 | 97.189 | 97.168 | 96.353 | 96.309 | 97.387 | 96.501 | 94.776 | 93.509 | 92.292 | 96.927 | 96.899 | 96.962 | 96.553 | 94.315 | 95.769 | 93.486 | 94.648 | 93.875 | 93.984 | 87.225 | 91.047 | 96.089 | 95.884 | 94.076 | 93.060 | 93.622 | 92.066 | 94.400 | 93.386 | 93.903 | 89.712 | 91.644 | 89.629 | 85.112 | 86.424 | 89.927 | 90.618 | 88.769 | 90.340 | 91.067 | 91.809 |
| 631 | 95.922 | 97.200 | 97.193 | 96.348 | 96.335 | 97.410 | 96.490 | 94.776 | 93.519 | 92.252 | 96.922 | 96.928 | 97.011 | 96.575 | 94.321 | 95.745 | 93.488 | 94.677 | 93.892 | 94.036 | 87.297 | 91.083 | 96.137 | 95.860 | 94.073 | 93.037 | 93.589 | 92.033 | 94.406 | 93.414 | 93.862 | 89.682 | 91.631 | 89.622 | 85.120 | 86.431 | 89.943 | 90.634 | 88.794 | 90.354 | 91.076 | 91.843 |
| 632 | 95.933 | 97.222 | 97.229 | 96.329 | 96.343 | 97.383 | 96.449 | 94.754 | 93.512 | 92.168 | 96.913 | 96.948 | 97.003 | 96.543 | 94.279 | 95.719 | 93.452 | 94.654 | 93.966 | 94.065 | 87.332 | 91.140 | 96.160 | 95.854 | 93.999 | 93.006 | 93.548 | 92.002 | 94.405 | 93.380 | 93.819 | 89.650 | 91.612 | 89.586 | 85.117 | 86.428 | 89.930 | 90.640 | 88.803 | 90.334 | 91.061 | 91.845 |
| 633 | 95.940 | 97.223 | 97.228 | 96.336 | 96.409 | 97.391 | 96.416 | 94.717 | 93.496 | 92.128 | 96.957 | 96.942 | 97.046 | 96.529 | 94.290 | 95.696 | 93.472 | 94.560 | 94.025 | 94.070 | 87.410 | 91.190 | 96.199 | 95.877 | 93.959 | 92.994 | 93.554 | 91.972 | 94.429 | 93.331 | 93.822 | 89.633 | 91.623 | 89.573 | 85.085 | 86.389 | 89.948 | 90.678 | 88.813 | 90.317 | 91.098 | 91.831 |
| 634 | 95.970 | 97.303 | 97.214 | 96.412 | 96.386 | 97.474 | 96.462 | 94.774 | 93.453 | 92.156 | 96.977 | 97.005 | 96.967 | 96.627 | 94.251 | 95.742 | 93.430 | 94.597 | 94.014 | 94.184 | 87.492 | 91.346 | 96.240 | 95.992 | 93.918 | 93.017 | 93.555 | 92.041 | 94.408 | 93.385 | 93.820 | 89.686 | 91.625 | 89.624 | 85.021 | 86.414 | 89.979 | 90.797 | 88.824 | 90.390 | 91.111 | 91.910 |
| 635 | 95.997 | 97.324 | 97.224 | 96.440 | 96.435 | 97.504 | 96.531 | 94.766 | 93.459 | 92.154 | 97.018 | 97.034 | 97.001 | 96.659 | 94.269 | 95.722 | 93.471 | 94.584 | 94.062 | 94.262 | 87.581 | 91.444 | 96.287 | 96.033 | 93.896 | 92.996 | 93.554 | 92.039 | 94.454 | 93.385 | 93.850 | 89.697 | 91.632 | 89.637 | 85.016 | 86.415 | 90.051 | 90.817 | 88.862 | 90.421 | 91.160 | 91.975 |
| 636 | 96.013 | 97.291 | 97.240 | 96.420 | 96.496 | 97.473 | 96.585 | 94.724 | 93.488 | 92.107 | 97.029 | 96.991 | 97.058 | 96.621 | 94.269 | 95.653 | 93.472 | 94.539 | 94.134 | 94.294 | 87.654 | 91.476 | 96.307 | 95.997 | 93.858 | 92.942 | 93.533 | 91.940 | 94.438 | 93.328 | 93.828 | 89.639 | 91.631 | 89.609 | 85.008 | 86.377 | 90.087 | 90.746 | 88.920 | 90.382 | 91.183 | 91.969 |
| 637 | 95.994 | 97.311 | 97.220 | 96.448 | 96.476 | 97.468 | 96.579 | 94.704 | 93.497 | 92.069 | 97.042 | 96.993 | 97.083 | 96.592 | 94.228 | 95.655 | 93.493 | 94.489 | 94.192 | 94.316 | 87.725 | 91.516 | 96.364 | 96.033 | 93.846 | 92.920 | 93.508 | 91.906 | 94.443 | 93.313 | 93.833 | 89.638 | 91.629 | 89.585 | 85.024 | 86.351 | 90.107 | 90.719 | 88.939 | 90.361 | 91.188 | 91.986 |
| 638 | 96.017 | 97.368 | 97.243 | 96.412 | 96.486 | 97.474 | 96.519 | 94.670 | 93.483 | 92.054 | 97.007 | 96.983 | 97.132 | 96.582 | 94.201 | 95.665 | 93.480 | 94.480 | 94.220 | 94.327 | 87.806 | 91.574 | 96.388 | 96.072 | 93.877 | 92.922 | 93.492 | 91.918 | 94.443 | 93.300 | 93.838 | 89.642 | 91.614 | 89.559 | 84.990 | 86.310 | 90.077 | 90.759 | 88.923 | 90.394 | 91.168 | 92.035 |
| 639 | 96.060 | 97.400 | 97.259 | 96.435 | 96.451 | 97.513 | 96.409 | 94.679 | 93.400 | 92.038 | 96.975 | 97.003 | 97.055 | 96.611 | 94.187 | 95.687 | 93.440 | 94.536 | 94.261 | 94.402 | 87.849 | 91.686 | 96.388 | 96.084 | 93.856 | 92.901 | 93.390 | 91.906 | 94.408 | 93.310 | 93.799 | 89.640 | 91.548 | 89.551 | 84.922 | 86.288 | 89.989 | 90.808 | 88.895 | 90.414 | 91.126 | 92.105 |
| 640 | 96.143 | 97.419 | 97.333 | 96.472 | 96.572 | 97.523 | 96.378 | 94.702 | 93.381 | 92.066 | 97.076 | 97.032 | 97.098 | 96.627 | 94.273 | 95.673 | 93.470 | 94.542 | 94.380 | 94.465 | 87.945 | 91.763 | 96.409 | 96.071 | 93.836 | 92.843 | 93.402 | 91.848 | 94.433 | 93.304 | 93.851 | 89.669 | 91.582 | 89.587 | 84.958 | 86.286 | 90.038 | 90.808 | 88.947 | 90.441 | 91.187 | 92.133 |
| 641 | 96.193 | 97.490 | 97.322 | 96.495 | 96.619 | 97.553 | 96.397 | 94.703 | 93.390 | 92.063 | 97.104 | 97.080 | 97.083 | 96.660 | 94.284 | 95.713 | 93.449 | 94.530 | 94.439 | 94.509 | 88.030 | 91.913 | 96.472 | 96.157 | 93.822 | 92.876 | 93.453 | 91.887 | 94.449 | 93.359 | 93.829 | 89.694 | 91.595 | 89.590 | 84.935 | 86.329 | 90.073 | 90.857 | 88.977 | 90.474 | 91.195 | 92.242 |
| 642 | 96.182 | 97.531 | 97.303 | 96.492 | 96.631 | 97.566 | 96.468 | 94.696 | 93.423 | 92.063 | 97.136 | 97.065 | 97.140 | 96.685 | 94.272 | 95.710 | 93.431 | 94.468 | 94.456 | 94.513 | 88.115 | 91.993 | 96.538 | 96.194 | 93.854 | 92.862 | 93.465 | 91.908 | 94.488 | 93.341 | 93.847 | 89.716 | 91.641 | 89.575 | 84.891 | 86.348 | 90.138 | 90.851 | 89.026 | 90.480 | 91.227 | 92.283 |
| 643 | 96.232 | 97.523 | 97.352 | 96.438 | 96.676 | 97.534 | 96.439 | 94.637 | 93.496 | 92.012 | 97.110 | 97.007 | 97.134 | 96.635 | 94.274 | 95.661 | 93.409 | 94.439 | 94.467 | 94.534 | 88.190 | 92.081 | 96.536 | 96.201 | 93.855 | 92.828 | 93.421 | 91.853 | 94.446 | 93.259 | 93.822 | 89.626 | 91.582 | 89.516 | 84.802 | 86.290 | 90.097 | 90.835 | 89.056 | 90.483 | 91.178 | 92.298 |
| 644 | 96.260 | 97.579 | 97.397 | 96.482 | 96.726 | 97.478 | 96.431 | 94.728 | 93.526 | 92.044 | 97.118 | 97.047 | 97.146 | 96.633 | 94.308 | 95.689 | 93.449 | 94.484 | 94.537 | 94.608 | 88.282 | 92.129 | 96.568 | 96.232 | 93.861 | 92.797 | 93.433 | 91.840 | 94.467 | 93.320 | 93.845 | 89.634 | 91.596 | 89.538 | 84.855 | 86.288 | 90.125 | 90.876 | 89.148 | 90.517 | 91.213 | 92.305 |
| 645 | 96.313 | 97.612 | 97.463 | 96.516 | 96.810 | 97.462 | 96.479 | 94.739 | 93.569 | 92.104 | 97.184 | 97.093 | 97.207 | 96.660 | 94.341 | 95.705 | 93.475 | 94.500 | 94.621 | 94.633 | 88.398 | 92.185 | 96.609 | 96.272 | 93.877 | 92.807 | 93.462 | 91.824 | 94.479 | 93.358 | 93.854 | 89.629 | 91.610 | 89.517 | 84.926 | 86.258 | 90.157 | 90.889 | 89.183 | 90.556 | 91.252 | 92.295 |
| 646 | 96.355 | 97.615 | 97.477 | 96.550 | 96.814 | 97.553 | 96.485 | 94.771 | 93.565 | 92.143 | 97.185 | 97.124 | 97.210 | 96.721 | 94.284 | 95.746 | 93.443 | 94.502 | 94.647 | 94.675 | 88.464 | 92.286 | 96.611 | 96.283 | 93.862 | 92.822 | 93.404 | 91.855 | 94.454 | 93.353 | 93.770 | 89.676 | 91.563 | 89.541 | 84.924 | 86.279 | 90.128 | 90.900 | 89.191 | 90.563 | 91.280 | 92.337 |
| 647 | 96.382 | 97.626 | 97.506 | 96.582 | 96.849 | 97.614 | 96.528 | 94.797 | 93.602 | 92.216 | 97.260 | 97.128 | 97.254 | 96.729 | 94.310 | 95.705 | 93.470 | 94.508 | 94.732 | 94.723 | 88.578 | 92.334 | 96.609 | 96.281 | 93.828 | 92.810 | 93.373 | 91.823 | 94.447 | 93.306 | 93.778 | 89.668 | 91.583 | 89.572 | 84.964 | 86.290 | 90.184 | 90.869 | 89.246 | 90.566 | 91.314 | 92.317 |
| 648 | 96.387 | 97.701 | 97.488 | 96.585 | 96.869 | 97.625 | 96.547 | 94.791 | 93.654 | 92.231 | 97.251 | 97.150 | 97.217 | 96.721 | 94.348 | 95.667 | 93.534 | 94.523 | 94.811 | 94.790 | 88.683 | 92.429 | 96.653 | 96.343 | 93.883 | 92.839 | 93.427 | 91.850 | 94.514 | 93.333 | 93.838 | 89.655 | 91.612 | 89.571 | 85.003 | 86.281 | 90.210 | 90.907 | 89.274 | 90.643 | 91.310 | 92.416 |
| 649 | 96.426 | 97.856 | 97.531 | 96.666 | 96.938 | 97.649 | 96.636 | 94.904 | 93.713 | 92.349 | 97.237 | 97.208 | 97.268 | 96.794 | 94.373 | 95.714 | 93.578 | 94.581 | 94.869 | 94.856 | 88.719 | 92.518 | 96.671 | 96.369 | 93.901 | 92.868 | 93.444 | 91.904 | 94.534 | 93.413 | 93.930 | 89.739 | 91.706 | 89.645 | 85.026 | 86.373 | 90.287 | 90.932 | 89.304 | 90.701 | 91.344 | 92.504 |
| 650 | 96.471 | 97.843 | 97.531 | 96.636 | 96.980 | 97.664 | 96.617 | 94.893 | 93.773 | 92.374 | 97.231 | 97.181 | 97.224 | 96.754 | 94.349 | 95.722 | 93.587 | 94.580 | 94.847 | 94.891 | 88.752 | 92.590 | 96.679 | 96.342 | 93.868 | 92.854 | 93.384 | 91.885 | 94.450 | 93.350 | 93.881 | 89.711 | 91.632 | 89.610 | 84.971 | 86.342 | 90.219 | 90.933 | 89.234 | 90.722 | 91.308 | 92.470 |
| 651 | 96.510 | 97.895 | 97.535 | 96.620 | 96.981 | 97.677 | 96.587 | 94.977 | 93.804 | 92.426 | 97.228 | 97.191 | 97.178 | 96.773 | 94.344 | 95.784 | 93.517 | 94.595 | 94.863 | 94.940 | 88.803 | 92.683 | 96.661 | 96.376 | 93.857 | 92.876 | 93.409 | 91.934 | 94.453 | 93.415 | 93.856 | 89.798 | 91.638 | 89.677 | 84.983 | 86.424 | 90.228 | 90.944 | 89.275 | 90.775 | 91.318 | 92.443 |
| 652 | 96.522 | 97.959 | 97.539 | 96.639 | 96.997 | 97.701 | 96.659 | 95.043 | 93.901 | 92.542 | 97.296 | 97.255 | 97.234 | 96.834 | 94.430 | 95.823 | 93.559 | 94.640 | 94.959 | 94.989 | 88.915 | 92.765 | 96.705 | 96.399 | 93.910 | 92.922 | 93.476 | 91.965 | 94.492 | 93.447 | 93.902 | 89.867 | 91.681 | 89.740 | 85.018 | 86.479 | 90.243 | 90.962 | 89.368 | 90.823 | 91.348 | 92.444 |
| 653 | 96.547 | 97.999 | 97.557 | 96.718 | 97.042 | 97.753 | 96.733 | 95.132 | 93.985 | 92.658 | 97.311 | 97.301 | 97.284 | 96.886 | 94.491 | 95.848 | 93.592 | 94.681 | 94.981 | 95.050 | 88.972 | 92.813 | 96.753 | 96.419 | 93.961 | 92.985 | 93.531 | 92.005 | 94.588 | 93.482 | 93.944 | 89.918 | 91.764 | 89.823 | 85.083 | 86.596 | 90.276 | 90.960 | 89.451 | 90.874 | 91.420 | 92.501 |
| 654 | 96.592 | 98.059 | 97.600 | 96.768 | 97.118 | 97.780 | 96.800 | 95.176 | 94.053 | 92.764 | 97.343 | 97.287 | 97.298 | 96.873 | 94.482 | 95.861 | 93.654 | 94.704 | 95.000 | 95.093 | 89.016 | 92.896 | 96.756 | 96.459 | 93.942 | 93.064 | 93.529 | 92.050 | 94.630 | 93.498 | 93.970 | 89.898 | 91.810 | 89.830 | 85.115 | 86.666 | 90.288 | 90.950 | 89.466 | 90.883 | 91.430 | 92.488 |
| 655 | 96.643 | 98.046 | 97.642 | 96.760 | 97.160 | 97.802 | 96.814 | 95.212 | 94.070 | 92.827 | 97.297 | 97.270 | 97.234 | 96.829 | 94.467 | 95.867 | 93.667 | 94.690 | 95.023 | 95.122 | 89.098 | 92.924 | 96.765 | 96.427 | 93.969 | 93.057 | 93.540 | 92.074 | 94.645 | 93.463 | 93.925 | 89.852 | 91.773 | 89.748 | 85.108 | 86.633 | 90.220 | 90.963 | 89.439 | 90.886 | 91.369 | 92.461 |
| 656 | 96.688 | 98.134 | 97.661 | 96.804 | 97.165 | 97.821 | 96.840 | 95.276 | 94.165 | 92.986 | 97.257 | 97.315 | 97.225 | 96.884 | 94.436 | 95.918 | 93.697 | 94.751 | 95.103 | 95.161 | 89.155 | 92.996 | 96.780 | 96.467 | 94.020 | 93.077 | 93.581 | 92.118 | 94.614 | 93.515 | 93.983 | 89.906 | 91.836 | 89.806 | 85.129 | 86.726 | 90.232 | 90.984 | 89.486 | 90.948 | 91.367 | 92.506 |
| 657 | 96.700 | 98.210 | 97.673 | 96.823 | 97.214 | 97.820 | 96.945 | 95.323 | 94.373 | 93.149 | 97.356 | 97.337 | 97.356 | 96.933 | 94.509 | 95.943 | 93.822 | 94.852 | 95.205 | 95.224 | 89.226 | 93.100 | 96.836 | 96.498 | 94.128 | 93.151 | 93.712 | 92.215 | 94.701 | 93.563 | 94.061 | 89.953 | 91.954 | 89.920 | 85.273 | 86.847 | 90.302 | 91.009 | 89.590 | 90.995 | 91.459 | 92.536 |
| 658 | 96.710 | 98.234 | 97.670 | 96.837 | 97.259 | 97.812 | 97.060 | 95.390 | 94.527 | 93.254 | 97.457 | 97.322 | 97.440 | 96.957 | 94.607 | 95.906 | 93.938 | 94.903 | 95.252 | 95.284 | 89.349 | 93.197 | 96.916 | 96.509 | 94.260 | 93.181 | 93.824 | 92.272 | 94.886 | 93.636 | 94.135 | 90.013 | 92.084 | 90.015 | 85.457 | 86.945 | 90.384 | 91.038 | 89.693 | 91.053 | 91.539 | 92.528 |
| 659 | 96.774 | 98.315 | 97.739 | 96.887 | 97.360 | 97.845 | 97.114 | 95.506 | 94.645 | 93.396 | 97.573 | 97.369 | 97.495 | 97.001 | 94.671 | 95.978 | 93.998 | 94.934 | 95.293 | 95.335 | 89.433 | 93.267 | 96.916 | 96.524 | 94.302 | 93.262 | 93.831 | 92.381 | 94.898 | 93.734 | 94.221 | 90.122 | 92.123 | 90.092 | 85.544 | 87.068 | 90.418 | 91.068 | 89.758 | 91.112 | 91.592 | 92.547 |
| 660 | 96.842 | 98.301 | 97.776 | 96.925 | 97.403 | 97.906 | 97.144 | 95.633 | 94.755 | 93.554 | 97.515 | 97.425 | 97.428 | 97.014 | 94.673 | 96.061 | 93.978 | 94.978 | 95.279 | 95.365 | 89.503 | 93.310 | 96.908 | 96.501 | 94.309 | 93.309 | 93.852 | 92.427 | 94.842 | 93.736 | 94.194 | 90.170 | 92.093 | 90.103 | 85.574 | 87.140 | 90.398 | 91.117 | 89.698 | 91.140 | 91.539 | 92.606 |
| 661 | 96.837 | 98.355 | 97.766 | 96.959 | 97.400 | 97.956 | 97.172 | 95.684 | 94.846 | 93.692 | 97.497 | 97.413 | 97.409 | 97.018 | 94.655 | 96.123 | 94.007 | 95.020 | 95.263 | 95.320 | 89.485 | 93.371 | 96.880 | 96.512 | 94.309 | 93.356 | 93.894 | 92.520 | 94.797 | 93.750 | 94.177 | 90.188 | 92.110 | 90.159 | 85.590 | 87.260 | 90.372 | 91.106 | 89.677 | 91.128 | 91.532 | 92.680 |
| 662 | 96.832 | 98.373 | 97.735 | 96.935 | 97.395 | 97.935 | 97.270 | 95.713 | 94.974 | 93.768 | 97.538 | 97.357 | 97.461 | 96.997 | 94.726 | 96.125 | 94.102 | 95.085 | 95.331 | 95.327 | 89.515 | 93.393 | 96.863 | 96.476 | 94.397 | 93.409 | 93.998 | 92.560 | 94.875 | 93.759 | 94.221 | 90.213 | 92.234 | 90.229 | 85.676 | 87.342 | 90.442 | 91.093 | 89.741 | 91.145 | 91.555 | 92.680 |
| 663 | 96.880 | 98.418 | 97.791 | 96.951 | 97.453 | 97.957 | 97.319 | 95.800 | 95.058 | 93.882 | 97.543 | 97.404 | 97.456 | 97.046 | 94.730 | 96.104 | 94.184 | 95.188 | 95.324 | 95.366 | 89.579 | 93.423 | 96.895 | 96.512 | 94.455 | 93.460 | 94.080 | 92.589 | 94.916 | 93.786 | 94.238 | 90.294 | 92.269 | 90.304 | 85.773 | 87.391 | 90.488 | 91.148 | 89.747 | 91.192 | 91.530 | 92.715 |
| 664 | 96.948 | 98.569 | 97.849 | 97.034 | 97.533 | 98.035 | 97.404 | 95.962 | 95.188 | 94.108 | 97.625 | 97.514 | 97.466 | 97.116 | 94.783 | 96.198 | 94.218 | 95.304 | 95.306 | 95.494 | 89.619 | 93.522 | 96.916 | 96.580 | 94.532 | 93.616 | 94.138 | 92.739 | 94.997 | 93.919 | 94.307 | 90.433 | 92.362 | 90.425 | 85.883 | 87.578 | 90.505 | 91.252 | 89.757 | 91.313 | 91.561 | 92.786 |
| 665 | 96.997 | 98.630 | 97.873 | 97.080 | 97.576 | 98.037 | 97.469 | 96.087 | 95.312 | 94.236 | 97.676 | 97.545 | 97.486 | 97.116 | 94.876 | 96.272 | 94.281 | 95.362 | 95.309 | 95.519 | 89.683 | 93.518 | 96.951 | 96.597 | 94.635 | 93.657 | 94.217 | 92.809 | 95.107 | 93.944 | 94.382 | 90.443 | 92.423 | 90.476 | 86.005 | 87.708 | 90.518 | 91.305 | 89.777 | 91.300 | 91.586 | 92.802 |
| 666 | 96.997 | 98.629 | 97.876 | 97.063 | 97.600 | 98.000 | 97.492 | 96.120 | 95.437 | 94.301 | 97.693 | 97.497 | 97.527 | 97.057 | 94.904 | 96.309 | 94.325 | 95.384 | 95.346 | 95.437 | 89.659 | 93.462 | 96.916 | 96.549 | 94.717 | 93.669 | 94.283 | 92.902 | 95.149 | 93.983 | 94.494 | 90.470 | 92.502 | 90.552 | 86.122 | 87.838 | 90.528 | 91.280 | 89.806 | 91.281 | 91.631 | 92.770 |
| 667 | 97.008 | 98.540 | 97.852 | 97.079 | 97.602 | 98.006 | 97.567 | 96.165 | 95.650 | 94.401 | 97.660 | 97.507 | 97.519 | 97.077 | 94.944 | 96.381 | 94.402 | 95.434 | 95.400 | 95.406 | 89.716 | 93.458 | 96.928 | 96.512 | 94.794 | 93.718 | 94.378 | 92.949 | 95.224 | 93.993 | 94.553 | 90.517 | 92.595 | 90.578 | 86.285 | 87.904 | 90.531 | 91.304 | 89.827 | 91.297 | 91.638 | 92.736 |
| 668 | 97.006 | 98.574 | 97.889 | 97.132 | 97.657 | 98.045 | 97.638 | 96.283 | 95.787 | 94.575 | 97.676 | 97.554 | 97.511 | 97.167 | 94.997 | 96.406 | 94.483 | 95.510 | 95.397 | 95.412 | 89.739 | 93.526 | 96.940 | 96.481 | 94.846 | 93.819 | 94.469 | 93.059 | 95.241 | 94.058 | 94.562 | 90.558 | 92.646 | 90.648 | 86.453 | 88.010 | 90.555 | 91.352 | 89.779 | 91.327 | 91.619 | 92.763 |
| 669 | 97.047 | 98.699 | 97.865 | 97.187 | 97.678 | 98.108 | 97.716 | 96.408 | 95.856 | 94.768 | 97.693 | 97.577 | 97.493 | 97.207 | 95.056 | 96.474 | 94.531 | 95.606 | 95.354 | 95.486 | 89.738 | 93.574 | 96.913 | 96.519 | 94.920 | 93.949 | 94.567 | 93.206 | 95.290 | 94.143 | 94.633 | 90.655 | 92.728 | 90.791 | 86.624 | 88.231 | 90.605 | 91.398 | 89.773 | 91.422 | 91.624 | 92.816 |
| 670 | 97.075 | 98.785 | 97.893 | 97.244 | 97.696 | 98.136 | 97.787 | 96.490 | 95.957 | 94.914 | 97.708 | 97.568 | 97.547 | 97.239 | 95.110 | 96.543 | 94.613 | 95.678 | 95.365 | 95.467 | 89.748 | 93.508 | 96.903 | 96.573 | 95.007 | 93.996 | 94.667 | 93.278 | 95.328 | 94.162 | 94.709 | 90.702 | 92.825 | 90.927 | 86.768 | 88.414 | 90.702 | 91.385 | 89.818 | 91.402 | 91.641 | 92.829 |
| 671 | 97.116 | 98.775 | 97.929 | 97.208 | 97.700 | 98.146 | 97.828 | 96.481 | 96.076 | 95.011 | 97.698 | 97.529 | 97.553 | 97.163 | 95.130 | 96.573 | 94.660 | 95.738 | 95.310 | 95.435 | 89.730 | 93.473 | 96.874 | 96.588 | 95.088 | 94.025 | 94.721 | 93.353 | 95.370 | 94.182 | 94.734 | 90.747 | 92.870 | 90.955 | 86.828 | 88.474 | 90.641 | 91.396 | 89.767 | 91.385 | 91.616 | 92.838 |
| 672 | 97.100 | 98.743 | 97.960 | 97.174 | 97.700 | 98.124 | 97.888 | 96.514 | 96.205 | 95.116 | 97.742 | 97.513 | 97.582 | 97.156 | 95.216 | 96.590 | 94.768 | 95.760 | 95.255 | 95.406 | 89.723 | 93.426 | 96.847 | 96.528 | 95.176 | 94.059 | 94.774 | 93.426 | 95.434 | 94.175 | 94.784 | 90.779 | 92.959 | 90.961 | 86.946 | 88.493 | 90.611 | 91.382 | 89.791 | 91.369 | 91.659 | 92.795 |
| 673 | 97.108 | 98.797 | 97.947 | 97.136 | 97.736 | 98.157 | 97.926 | 96.605 | 96.265 | 95.261 | 97.753 | 97.558 | 97.583 | 97.161 | 95.285 | 96.557 | 94.845 | 95.848 | 95.277 | 95.384 | 89.708 | 93.399 | 96.856 | 96.480 | 95.277 | 94.160 | 94.871 | 93.537 | 95.500 | 94.262 | 94.875 | 90.851 | 93.047 | 91.022 | 87.135 | 88.586 | 90.591 | 91.420 | 89.795 | 91.390 | 91.676 | 92.810 |
| 674 | 97.105 | 98.869 | 97.949 | 97.186 | 97.756 | 98.211 | 98.000 | 96.711 | 96.387 | 95.398 | 97.718 | 97.612 | 97.626 | 97.211 | 95.312 | 96.636 | 94.881 | 95.935 | 95.295 | 95.384 | 89.695 | 93.389 | 96.879 | 96.508 | 95.312 | 94.268 | 94.936 | 93.647 | 95.526 | 94.351 | 94.935 | 90.974 | 93.115 | 91.136 | 87.322 | 88.788 | 90.596 | 91.476 | 89.845 | 91.419 | 91.692 | 92.843 |
| 675 | 97.132 | 98.875 | 97.929 | 97.224 | 97.774 | 98.194 | 98.082 | 96.793 | 96.548 | 95.504 | 97.696 | 97.620 | 97.618 | 97.214 | 95.297 | 96.662 | 95.025 | 95.977 | 95.268 | 95.378 | 89.662 | 93.360 | 96.839 | 96.464 | 95.421 | 94.346 | 95.050 | 93.709 | 95.548 | 94.424 | 95.010 | 91.061 | 93.171 | 91.171 | 87.490 | 88.943 | 90.615 | 91.458 | 89.872 | 91.394 | 91.687 | 92.869 |
| 676 | 97.194 | 98.926 | 97.976 | 97.240 | 97.868 | 98.176 | 98.129 | 96.854 | 96.697 | 95.603 | 97.706 | 97.612 | 97.608 | 97.213 | 95.322 | 96.689 | 95.078 | 96.042 | 95.152 | 95.382 | 89.613 | 93.338 | 96.815 | 96.429 | 95.485 | 94.405 | 95.104 | 93.762 | 95.597 | 94.468 | 95.057 | 91.096 | 93.202 | 91.260 | 87.629 | 89.054 | 90.598 | 91.474 | 89.826 | 91.414 | 91.669 | 92.849 |
| 677 | 97.232 | 98.995 | 98.034 | 97.286 | 97.929 | 98.214 | 98.213 | 96.974 | 96.812 | 95.763 | 97.707 | 97.656 | 97.625 | 97.289 | 95.411 | 96.712 | 95.193 | 96.106 | 95.081 | 95.385 | 89.599 | 93.321 | 96.854 | 96.430 | 95.584 | 94.488 | 95.187 | 93.836 | 95.684 | 94.524 | 95.116 | 91.148 | 93.334 | 91.435 | 87.789 | 89.189 | 90.605 | 91.474 | 89.824 | 91.427 | 91.707 | 92.859 |
| 678 | 97.283 | 99.039 | 98.067 | 97.317 | 97.942 | 98.294 | 98.252 | 97.050 | 96.913 | 95.934 | 97.738 | 97.676 | 97.635 | 97.308 | 95.492 | 96.759 | 95.256 | 96.234 | 95.029 | 95.384 | 89.574 | 93.289 | 96.893 | 96.471 | 95.669 | 94.573 | 95.233 | 93.947 | 95.759 | 94.586 | 95.191 | 91.187 | 93.407 | 91.545 | 87.936 | 89.349 | 90.548 | 91.493 | 89.803 | 91.444 | 91.726 | 92.894 |
| 679 | 97.229 | 99.028 | 98.089 | 97.308 | 97.927 | 98.312 | 98.297 | 97.071 | 97.017 | 96.006 | 97.787 | 97.616 | 97.640 | 97.256 | 95.502 | 96.816 | 95.278 | 96.325 | 94.911 | 95.338 | 89.520 | 93.243 | 96.841 | 96.463 | 95.714 | 94.637 | 95.259 | 94.017 | 95.770 | 94.576 | 95.217 | 91.222 | 93.498 | 91.605 | 88.091 | 89.457 | 90.491 | 91.450 | 89.804 | 91.384 | 91.750 | 92.906 |
| 680 | 97.236 | 99.045 | 98.050 | 97.330 | 97.953 | 98.352 | 98.336 | 97.131 | 97.109 | 96.135 | 97.815 | 97.649 | 97.622 | 97.272 | 95.530 | 96.901 | 95.328 | 96.393 | 94.822 | 95.324 | 89.466 | 93.178 | 96.758 | 96.410 | 95.790 | 94.743 | 95.390 | 94.142 | 95.801 | 94.641 | 95.300 | 91.346 | 93.576 | 91.664 | 88.263 | 89.614 | 90.407 | 91.450 | 89.798 | 91.385 | 91.780 | 92.918 |
| 681 | 97.223 | 99.048 | 98.028 | 97.330 | 97.929 | 98.318 | 98.404 | 97.186 | 97.208 | 96.209 | 97.812 | 97.639 | 97.621 | 97.291 | 95.606 | 96.960 | 95.311 | 96.439 | 94.767 | 95.293 | 89.431 | 93.099 | 96.754 | 96.401 | 95.843 | 94.833 | 95.492 | 94.256 | 95.812 | 94.727 | 95.329 | 91.397 | 93.651 | 91.721 | 88.425 | 89.726 | 90.380 | 91.517 | 89.820 | 91.419 | 91.785 | 92.888 |
| 682 | 97.292 | 99.072 | 98.072 | 97.358 | 97.984 | 98.276 | 98.494 | 97.254 | 97.371 | 96.300 | 97.821 | 97.626 | 97.675 | 97.313 | 95.722 | 97.018 | 95.470 | 96.494 | 94.708 | 95.250 | 89.388 | 93.065 | 96.773 | 96.372 | 95.952 | 94.910 | 95.631 | 94.304 | 95.911 | 94.771 | 95.426 | 91.442 | 93.805 | 91.789 | 88.620 | 89.835 | 90.401 | 91.503 | 89.856 | 91.441 | 91.810 | 92.860 |
| 683 | 97.323 | 99.143 | 98.099 | 97.374 | 98.027 | 98.302 | 98.536 | 97.322 | 97.465 | 96.388 | 97.820 | 97.607 | 97.701 | 97.339 | 95.750 | 97.110 | 95.541 | 96.545 | 94.619 | 95.232 | 89.305 | 93.029 | 96.755 | 96.340 | 96.032 | 94.956 | 95.701 | 94.390 | 95.995 | 94.812 | 95.533 | 91.523 | 93.891 | 91.866 | 88.740 | 89.972 | 90.365 | 91.490 | 89.833 | 91.459 | 91.755 | 92.868 |
| 684 | 97.338 | 99.158 | 98.162 | 97.382 | 98.097 | 98.336 | 98.604 | 97.399 | 97.523 | 96.516 | 97.827 | 97.647 | 97.699 | 97.379 | 95.763 | 97.153 | 95.674 | 96.592 | 94.508 | 95.163 | 89.224 | 92.965 | 96.715 | 96.351 | 96.112 | 94.978 | 95.776 | 94.454 | 96.082 | 94.890 | 95.568 | 91.613 | 93.946 | 91.955 | 88.890 | 90.114 | 90.327 | 91.486 | 89.785 | 91.471 | 91.709 | 92.921 |
| 685 | 97.375 | 99.189 | 98.175 | 97.393 | 98.138 | 98.393 | 98.637 | 97.473 | 97.610 | 96.658 | 97.839 | 97.693 | 97.691 | 97.417 | 95.813 | 97.191 | 95.673 | 96.693 | 94.409 | 95.126 | 89.179 | 92.893 | 96.675 | 96.356 | 96.195 | 95.051 | 95.881 | 94.549 | 96.135 | 94.980 | 95.623 | 91.691 | 94.006 | 92.044 | 89.032 | 90.284 | 90.331 | 91.494 | 89.797 | 91.479 | 91.778 | 92.956 |
| 686 | 97.335 | 99.154 | 98.136 | 97.399 | 98.122 | 98.360 | 98.662 | 97.503 | 97.729 | 96.704 | 97.841 | 97.668 | 97.691 | 97.365 | 95.863 | 97.160 | 95.740 | 96.751 | 94.218 | 95.047 | 89.117 | 92.785 | 96.700 | 96.327 | 96.264 | 95.137 | 95.969 | 94.631 | 96.200 | 95.013 | 95.668 | 91.686 | 94.119 | 92.073 | 89.184 | 90.354 | 90.356 | 91.463 | 89.829 | 91.440 | 91.857 | 92.951 |
| 687 | 97.329 | 99.197 | 98.118 | 97.441 | 98.144 | 98.385 | 98.691 | 97.562 | 97.812 | 96.775 | 97.818 | 97.667 | 97.689 | 97.351 | 95.911 | 97.203 | 95.775 | 96.804 | 93.942 | 95.017 | 89.022 | 92.744 | 96.703 | 96.326 | 96.309 | 95.258 | 96.073 | 94.737 | 96.235 | 95.013 | 95.715 | 91.718 | 94.186 | 92.210 | 89.302 | 90.464 | 90.319 | 91.481 | 89.779 | 91.441 | 91.846 | 92.924 |
| 688 | 97.335 | 99.309 | 98.143 | 97.504 | 98.121 | 98.447 | 98.774 | 97.654 | 97.872 | 96.891 | 97.846 | 97.695 | 97.675 | 97.405 | 95.931 | 97.312 | 95.841 | 96.879 | 93.576 | 95.069 | 88.986 | 92.788 | 96.664 | 96.362 | 96.404 | 95.392 | 96.136 | 94.884 | 96.253 | 95.110 | 95.735 | 91.826 | 94.233 | 92.348 | 89.432 | 90.618 | 90.306 | 91.571 | 89.751 | 91.496 | 91.787 | 92.967 |
| 689 | 97.408 | 99.351 | 98.189 | 97.529 | 98.142 | 98.459 | 98.842 | 97.712 | 97.959 | 97.016 | 97.874 | 97.697 | 97.689 | 97.469 | 95.936 | 97.360 | 95.891 | 96.955 | 93.044 | 95.038 | 88.910 | 92.796 | 96.639 | 96.364 | 96.451 | 95.454 | 96.179 | 94.986 | 96.324 | 95.184 | 95.764 | 91.914 | 94.292 | 92.460 | 89.580 | 90.753 | 90.313 | 91.591 | 89.757 | 91.508 | 91.815 | 92.985 |
| 690 | 97.454 | 99.382 | 98.184 | 97.529 | 98.132 | 98.465 | 98.849 | 97.802 | 98.019 | 97.129 | 97.886 | 97.702 | 97.710 | 97.457 | 95.942 | 97.426 | 95.929 | 97.032 | 92.442 | 95.008 | 88.875 | 92.750 | 96.675 | 96.355 | 96.489 | 95.499 | 96.246 | 95.080 | 96.373 | 95.260 | 95.842 | 92.013 | 94.367 | 92.512 | 89.712 | 90.891 | 90.334 | 91.549 | 89.763 | 91.484 | 91.809 | 93.019 |
| 691 | 97.490 | 99.359 | 98.204 | 97.487 | 98.168 | 98.445 | 98.848 | 97.817 | 98.076 | 97.177 | 97.838 | 97.671 | 97.687 | 97.430 | 95.951 | 97.418 | 96.008 | 97.026 | 91.891 | 94.940 | 88.788 | 92.642 | 96.722 | 96.348 | 96.559 | 95.534 | 96.270 | 95.119 | 96.400 | 95.268 | 95.932 | 91.999 | 94.441 | 92.510 | 89.825 | 90.956 | 90.341 | 91.500 | 89.708 | 91.464 | 91.799 | 93.007 |
| 692 | 97.501 | 99.313 | 98.225 | 97.454 | 98.186 | 98.433 | 98.861 | 97.789 | 98.109 | 97.171 | 97.824 | 97.629 | 97.646 | 97.378 | 95.986 | 97.403 | 96.005 | 97.052 | 91.508 | 94.908 | 88.731 | 92.529 | 96.671 | 96.300 | 96.614 | 95.576 | 96.323 | 95.072 | 96.443 | 95.258 | 95.986 | 91.967 | 94.512 | 92.555 | 89.944 | 91.016 | 90.362 | 91.499 | 89.653 | 91.400 | 91.782 | 92.937 |
| 693 | 97.495 | 99.312 | 98.199 | 97.530 | 98.157 | 98.442 | 98.901 | 97.821 | 98.169 | 97.255 | 97.856 | 97.680 | 97.638 | 97.374 | 96.015 | 97.429 | 96.068 | 97.128 | 91.289 | 94.907 | 88.707 | 92.524 | 96.669 | 96.309 | 96.715 | 95.680 | 96.369 | 95.147 | 96.476 | 95.343 | 96.016 | 92.057 | 94.587 | 92.650 | 90.057 | 91.127 | 90.333 | 91.506 | 89.648 | 91.460 | 91.772 | 92.959 |
| 694 | 97.500 | 99.389 | 98.229 | 97.576 | 98.143 | 98.460 | 98.935 | 97.883 | 98.198 | 97.349 | 97.884 | 97.700 | 97.668 | 97.420 | 96.065 | 97.493 | 96.047 | 97.240 | 91.123 | 94.918 | 88.685 | 92.543 | 96.659 | 96.334 | 96.707 | 95.768 | 96.412 | 95.253 | 96.470 | 95.403 | 96.038 | 92.202 | 94.670 | 92.739 | 90.160 | 91.256 | 90.299 | 91.529 | 89.633 | 91.479 | 91.783 | 92.983 |
| 695 | 97.512 | 99.408 | 98.264 | 97.577 | 98.180 | 98.483 | 99.004 | 97.936 | 98.276 | 97.430 | 97.898 | 97.695 | 97.687 | 97.437 | 96.133 | 97.575 | 96.189 | 97.330 | 91.065 | 94.891 | 88.700 | 92.514 | 96.678 | 96.343 | 96.752 | 95.783 | 96.535 | 95.346 | 96.498 | 95.446 | 96.101 | 92.298 | 94.757 | 92.815 | 90.304 | 91.372 | 90.295 | 91.494 | 89.625 | 91.479 | 91.766 | 93.026 |
| 696 | 97.567 | 99.362 | 98.270 | 97.517 | 98.223 | 98.463 | 99.056 | 97.929 | 98.377 | 97.457 | 97.898 | 97.677 | 97.703 | 97.424 | 96.225 | 97.545 | 96.255 | 97.313 | 91.047 | 94.829 | 88.686 | 92.445 | 96.699 | 96.289 | 96.874 | 95.795 | 96.624 | 95.357 | 96.600 | 95.438 | 96.202 | 92.253 | 94.824 | 92.844 | 90.470 | 91.451 | 90.340 | 91.434 | 89.647 | 91.366 | 91.825 | 92.991 |
| 697 | 97.606 | 99.408 | 98.253 | 97.539 | 98.223 | 98.497 | 99.059 | 97.967 | 98.438 | 97.518 | 97.874 | 97.710 | 97.678 | 97.437 | 96.252 | 97.599 | 96.353 | 97.342 | 91.108 | 94.825 | 88.690 | 92.439 | 96.697 | 96.310 | 96.913 | 95.855 | 96.707 | 95.447 | 96.688 | 95.512 | 96.227 | 92.314 | 94.826 | 92.944 | 90.588 | 91.581 | 90.316 | 91.445 | 89.634 | 91.359 | 91.799 | 92.946 |
| 698 | 97.582 | 99.465 | 98.240 | 97.596 | 98.197 | 98.515 | 99.060 | 98.031 | 98.448 | 97.619 | 97.855 | 97.751 | 97.671 | 97.414 | 96.256 | 97.610 | 96.381 | 97.416 | 91.213 | 94.834 | 88.642 | 92.441 | 96.762 | 96.394 | 96.927 | 95.939 | 96.720 | 95.544 | 96.699 | 95.554 | 96.235 | 92.400 | 94.856 | 93.027 | 90.641 | 91.699 | 90.299 | 91.422 | 89.603 | 91.377 | 91.768 | 92.920 |
| 699 | 97.617 | 99.473 | 98.315 | 97.613 | 98.272 | 98.561 | 99.061 | 98.064 | 98.461 | 97.669 | 97.855 | 97.747 | 97.701 | 97.442 | 96.280 | 97.643 | 96.419 | 97.518 | 91.375 | 94.847 | 88.609 | 92.437 | 96.753 | 96.435 | 96.950 | 96.002 | 96.788 | 95.618 | 96.775 | 95.626 | 96.309 | 92.491 | 94.980 | 93.095 | 90.744 | 91.820 | 90.336 | 91.446 | 89.600 | 91.371 | 91.790 | 92.963 |
| 700 | 97.626 | 99.398 | 98.315 | 97.618 | 98.285 | 98.556 | 99.080 | 98.063 | 98.506 | 97.662 | 97.886 | 97.727 | 97.746 | 97.449 | 96.313 | 97.622 | 96.497 | 97.480 | 91.634 | 94.831 | 88.620 | 92.443 | 96.796 | 96.411 | 97.057 | 96.057 | 96.906 | 95.694 | 96.832 | 95.671 | 96.366 | 92.520 | 95.039 | 93.119 | 90.890 | 91.864 | 90.350 | 91.428 | 89.638 | 91.344 | 91.838 | 93.013 |
| 701 | 97.657 | 99.416 | 98.358 | 97.595 | 98.310 | 98.521 | 99.141 | 98.085 | 98.561 | 97.670 | 97.912 | 97.723 | 97.758 | 97.521 | 96.351 | 97.642 | 96.504 | 97.494 | 91.902 | 94.870 | 88.664 | 92.490 | 96.802 | 96.395 | 97.126 | 96.122 | 96.954 | 95.732 | 96.872 | 95.705 | 96.423 | 92.509 | 95.144 | 93.135 | 91.038 | 91.905 | 90.299 | 91.431 | 89.644 | 91.287 | 91.892 | 93.010 |
| 702 | 97.675 | 99.440 | 98.364 | 97.625 | 98.294 | 98.531 | 99.138 | 98.108 | 98.585 | 97.746 | 97.934 | 97.736 | 97.720 | 97.506 | 96.369 | 97.704 | 96.526 | 97.527 | 92.145 | 94.870 | 88.625 | 92.455 | 96.782 | 96.427 | 97.150 | 96.126 | 96.936 | 95.805 | 96.895 | 95.730 | 96.396 | 92.530 | 95.134 | 93.180 | 91.066 | 91.999 | 90.209 | 91.392 | 89.586 | 91.310 | 91.809 | 92.979 |
| 703 | 97.626 | 99.401 | 98.329 | 97.613 | 98.268 | 98.481 | 99.120 | 98.120 | 98.606 | 97.767 | 97.994 | 97.751 | 97.745 | 97.456 | 96.406 | 97.699 | 96.510 | 97.557 | 92.459 | 94.852 | 88.580 | 92.380 | 96.791 | 96.413 | 97.167 | 96.116 | 96.926 | 95.780 | 96.933 | 95.701 | 96.419 | 92.504 | 95.190 | 93.189 | 91.110 | 92.092 | 90.205 | 91.364 | 89.593 | 91.232 | 91.765 | 92.949 |
| 704 | 97.616 | 99.379 | 98.306 | 97.583 | 98.248 | 98.467 | 99.145 | 98.104 | 98.638 | 97.757 | 98.029 | 97.739 | 97.787 | 97.452 | 96.450 | 97.680 | 96.593 | 97.511 | 92.841 | 94.808 | 88.663 | 92.366 | 96.814 | 96.352 | 97.219 | 96.148 | 97.034 | 95.805 | 96.997 | 95.719 | 96.511 | 92.555 | 95.265 | 93.211 | 91.235 | 92.171 | 90.234 | 91.379 | 89.617 | 91.200 | 91.792 | 92.950 |
| 705 | 97.622 | 99.399 | 98.333 | 97.550 | 98.268 | 98.461 | 99.137 | 98.113 | 98.609 | 97.781 | 97.955 | 97.737 | 97.714 | 97.474 | 96.432 | 97.652 | 96.549 | 97.501 | 93.088 | 94.787 | 88.707 | 92.368 | 96.821 | 96.340 | 97.228 | 96.178 | 97.031 | 95.851 | 96.964 | 95.767 | 96.494 | 92.621 | 95.251 | 93.222 | 91.276 | 92.205 | 90.198 | 91.461 | 89.559 | 91.152 | 91.752 | 92.959 |
| 706 | 97.647 | 99.428 | 98.342 | 97.571 | 98.291 | 98.505 | 99.182 | 98.160 | 98.654 | 97.822 | 97.917 | 97.742 | 97.680 | 97.516 | 96.465 | 97.669 | 96.606 | 97.557 | 93.286 | 94.803 | 88.727 | 92.396 | 96.854 | 96.395 | 97.280 | 96.192 | 97.101 | 95.909 | 97.000 | 95.818 | 96.569 | 92.661 | 95.340 | 93.288 | 91.355 | 92.273 | 90.212 | 91.482 | 89.553 | 91.136 | 91.795 | 92.978 |
| 707 | 97.652 | 99.382 | 98.348 | 97.561 | 98.311 | 98.508 | 99.179 | 98.171 | 98.690 | 97.815 | 97.945 | 97.724 | 97.729 | 97.479 | 96.464 | 97.719 | 96.573 | 97.610 | 93.445 | 94.825 | 88.691 | 92.408 | 96.872 | 96.402 | 97.324 | 96.215 | 97.162 | 95.941 | 97.031 | 95.815 | 96.605 | 92.667 | 95.393 | 93.351 | 91.446 | 92.338 | 90.250 | 91.435 | 89.548 | 91.120 | 91.823 | 92.940 |
| 708 | 97.663 | 99.356 | 98.350 | 97.519 | 98.347 | 98.543 | 99.188 | 98.170 | 98.701 | 97.858 | 97.984 | 97.722 | 97.776 | 97.458 | 96.458 | 97.730 | 96.616 | 97.661 | 93.611 | 94.832 | 88.678 | 92.410 | 96.864 | 96.421 | 97.383 | 96.262 | 97.246 | 95.973 | 97.035 | 95.818 | 96.654 | 92.694 | 95.408 | 93.385 | 91.523 | 92.418 | 90.242 | 91.380 | 89.531 | 91.081 | 91.833 | 92.930 |
| 709 | 97.685 | 99.348 | 98.383 | 97.536 | 98.364 | 98.534 | 99.208 | 98.164 | 98.749 | 97.898 | 98.026 | 97.731 | 97.810 | 97.448 | 96.494 | 97.754 | 96.639 | 97.692 | 93.738 | 94.857 | 88.733 | 92.422 | 96.896 | 96.453 | 97.461 | 96.348 | 97.276 | 96.031 | 97.075 | 95.831 | 96.693 | 92.716 | 95.431 | 93.435 | 91.591 | 92.463 | 90.254 | 91.356 | 89.527 | 91.055 | 91.821 | 92.943 |
| 710 | 97.678 | 99.414 | 98.359 | 97.553 | 98.359 | 98.560 | 99.157 | 98.231 | 98.733 | 97.966 | 97.987 | 97.750 | 97.739 | 97.457 | 96.483 | 97.785 | 96.655 | 97.729 | 93.740 | 94.908 | 88.712 | 92.438 | 96.898 | 96.484 | 97.452 | 96.378 | 97.221 | 96.077 | 97.108 | 95.903 | 96.705 | 92.732 | 95.434 | 93.483 | 91.598 | 92.520 | 90.237 | 91.467 | 89.505 | 91.074 | 91.749 | 93.008 |
| 711 | 97.668 | 99.422 | 98.349 | 97.575 | 98.320 | 98.569 | 99.175 | 98.271 | 98.742 | 97.997 | 97.986 | 97.790 | 97.725 | 97.487 | 96.493 | 97.794 | 96.655 | 97.768 | 93.802 | 94.949 | 88.723 | 92.511 | 96.922 | 96.514 | 97.433 | 96.428 | 97.213 | 96.110 | 97.109 | 95.905 | 96.732 | 92.768 | 95.511 | 93.541 | 91.623 | 92.590 | 90.251 | 91.462 | 89.507 | 91.076 | 91.765 | 93.038 |
| 712 | 97.675 | 99.472 | 98.444 | 97.618 | 98.329 | 98.632 | 99.195 | 98.285 | 98.761 | 98.056 | 97.984 | 97.867 | 97.735 | 97.571 | 96.488 | 97.840 | 96.634 | 97.802 | 93.898 | 94.959 | 88.744 | 92.554 | 96.933 | 96.523 | 97.434 | 96.469 | 97.222 | 96.190 | 97.105 | 95.959 | 96.712 | 92.865 | 95.574 | 93.625 | 91.655 | 92.662 | 90.234 | 91.491 | 89.502 | 91.086 | 91.746 | 93.029 |
| 713 | 97.687 | 99.465 | 98.417 | 97.630 | 98.324 | 98.664 | 99.172 | 98.262 | 98.728 | 98.084 | 97.982 | 97.891 | 97.759 | 97.593 | 96.492 | 97.831 | 96.634 | 97.763 | 93.927 | 94.940 | 88.754 | 92.559 | 96.927 | 96.525 | 97.457 | 96.468 | 97.255 | 96.225 | 97.134 | 95.983 | 96.712 | 92.921 | 95.591 | 93.664 | 91.671 | 92.709 | 90.198 | 91.445 | 89.519 | 91.069 | 91.743 | 92.998 |
| 714 | 97.692 | 99.480 | 98.406 | 97.647 | 98.344 | 98.617 | 99.164 | 98.273 | 98.778 | 98.085 | 98.004 | 97.836 | 97.764 | 97.556 | 96.528 | 97.822 | 96.610 | 97.763 | 93.998 | 94.940 | 88.791 | 92.571 | 96.935 | 96.524 | 97.468 | 96.487 | 97.255 | 96.224 | 97.203 | 95.989 | 96.716 | 92.914 | 95.617 | 93.677 | 91.668 | 92.710 | 90.207 | 91.412 | 89.516 | 91.042 | 91.748 | 92.971 |
| 715 | 97.651 | 99.453 | 98.352 | 97.646 | 98.333 | 98.610 | 99.169 | 98.293 | 98.807 | 98.096 | 98.004 | 97.838 | 97.738 | 97.503 | 96.535 | 97.812 | 96.727 | 97.824 | 94.006 | 94.967 | 88.781 | 92.577 | 96.958 | 96.507 | 97.487 | 96.451 | 97.284 | 96.257 | 97.233 | 95.984 | 96.738 | 92.933 | 95.606 | 93.676 | 91.687 | 92.778 | 90.212 | 91.460 | 89.553 | 91.068 | 91.762 | 92.985 |
| 716 | 97.640 | 99.465 | 98.387 | 97.656 | 98.351 | 98.564 | 99.200 | 98.259 | 98.889 | 98.102 | 98.022 | 97.818 | 97.707 | 97.533 | 96.550 | 97.822 | 96.689 | 97.892 | 94.058 | 94.976 | 88.778 | 92.623 | 96.957 | 96.532 | 97.463 | 96.476 | 97.305 | 96.251 | 97.194 | 95.971 | 96.772 | 92.915 | 95.635 | 93.698 | 91.738 | 92.832 | 90.233 | 91.425 | 89.597 | 91.041 | 91.773 | 92.968 |
| 717 | 97.702 | 99.503 | 98.405 | 97.665 | 98.342 | 98.608 | 99.203 | 98.257 | 98.879 | 98.105 | 98.030 | 97.837 | 97.697 | 97.576 | 96.540 | 97.846 | 96.730 | 97.894 | 94.117 | 95.003 | 88.786 | 92.655 | 96.947 | 96.552 | 97.502 | 96.515 | 97.339 | 96.320 | 97.209 | 96.036 | 96.798 | 92.984 | 95.674 | 93.720 | 91.794 | 92.895 | 90.246 | 91.484 | 89.624 | 91.123 | 91.763 | 93.020 |
| 718 | 97.732 | 99.452 | 98.392 | 97.663 | 98.382 | 98.586 | 99.202 | 98.262 | 98.848 | 98.066 | 98.044 | 97.822 | 97.718 | 97.567 | 96.541 | 97.827 | 96.747 | 97.835 | 94.161 | 94.985 | 88.842 | 92.661 | 96.942 | 96.563 | 97.521 | 96.547 | 97.361 | 96.302 | 97.250 | 96.076 | 96.827 | 92.959 | 95.712 | 93.720 | 91.853 | 92.920 | 90.260 | 91.448 | 89.633 | 91.121 | 91.751 | 92.976 |
| 719 | 97.717 | 99.480 | 98.385 | 97.678 | 98.372 | 98.585 | 99.186 | 98.259 | 98.873 | 98.086 | 98.052 | 97.860 | 97.760 | 97.541 | 96.572 | 97.826 | 96.852 | 97.833 | 94.242 | 94.963 | 88.890 | 92.649 | 96.940 | 96.562 | 97.586 | 96.547 | 97.381 | 96.335 | 97.279 | 96.046 | 96.866 | 92.979 | 95.744 | 93.744 | 91.883 | 92.976 | 90.304 | 91.452 | 89.657 | 91.101 | 91.767 | 92.957 |
| 720 | 97.650 | 99.504 | 98.349 | 97.673 | 98.349 | 98.583 | 99.167 | 98.283 | 98.875 | 98.139 | 98.040 | 97.889 | 97.793 | 97.554 | 96.604 | 97.864 | 96.831 | 97.909 | 94.270 | 94.972 | 88.897 | 92.655 | 96.923 | 96.590 | 97.591 | 96.565 | 97.386 | 96.405 | 97.291 | 96.069 | 96.882 | 93.018 | 95.723 | 93.816 | 91.898 | 93.017 | 90.312 | 91.526 | 89.655 | 91.117 | 91.798 | 92.959 |
| 721 | 97.705 | 99.568 | 98.406 | 97.652 | 98.394 | 98.573 | 99.174 | 98.300 | 98.908 | 98.141 | 98.032 | 97.905 | 97.817 | 97.584 | 96.631 | 97.881 | 96.783 | 97.972 | 94.336 | 95.011 | 88.890 | 92.685 | 96.905 | 96.613 | 97.589 | 96.634 | 97.384 | 96.394 | 97.280 | 96.040 | 96.881 | 93.075 | 95.723 | 93.876 | 91.913 | 93.052 | 90.300 | 91.540 | 89.653 | 91.149 | 91.808 | 92.978 |
| 722 | 97.761 | 99.486 | 98.444 | 97.605 | 98.423 | 98.584 | 99.218 | 98.319 | 98.934 | 98.052 | 98.082 | 97.904 | 97.848 | 97.617 | 96.646 | 97.903 | 96.785 | 97.967 | 94.392 | 95.046 | 88.916 | 92.697 | 96.955 | 96.591 | 97.606 | 96.654 | 97.427 | 96.418 | 97.327 | 96.083 | 96.921 | 93.099 | 95.783 | 93.897 | 91.999 | 93.090 | 90.363 | 91.580 | 89.687 | 91.187 | 91.836 | 93.005 |
| 723 | 97.755 | 99.492 | 98.459 | 97.610 | 98.458 | 98.628 | 99.261 | 98.338 | 98.995 | 98.045 | 98.147 | 97.948 | 97.865 | 97.686 | 96.689 | 97.924 | 96.862 | 97.945 | 94.444 | 95.076 | 88.984 | 92.747 | 97.050 | 96.621 | 97.657 | 96.693 | 97.511 | 96.431 | 97.372 | 96.130 | 96.965 | 93.102 | 95.861 | 93.915 | 92.080 | 93.134 | 90.450 | 91.577 | 89.764 | 91.220 | 91.876 | 93.059 |
| 724 | 97.734 | 99.492 | 98.412 | 97.661 | 98.460 | 98.636 | 99.251 | 98.316 | 98.965 | 98.101 | 98.172 | 97.972 | 97.851 | 97.663 | 96.725 | 97.921 | 96.870 | 97.940 | 94.468 | 95.044 | 89.025 | 92.754 | 97.081 | 96.645 | 97.665 | 96.677 | 97.491 | 96.443 | 97.380 | 96.093 | 96.993 | 93.145 | 95.883 | 93.920 | 92.103 | 93.153 | 90.495 | 91.596 | 89.796 | 91.240 | 91.882 | 93.064 |
| 725 | 97.738 | 99.486 | 98.439 | 97.685 | 98.493 | 98.597 | 99.265 | 98.303 | 98.970 | 98.160 | 98.206 | 97.903 | 97.859 | 97.628 | 96.732 | 97.880 | 96.882 | 97.945 | 94.464 | 95.022 | 89.046 | 92.751 | 97.123 | 96.635 | 97.635 | 96.665 | 97.478 | 96.473 | 97.412 | 96.081 | 97.019 | 93.128 | 95.896 | 93.943 | 92.134 | 93.153 | 90.512 | 91.628 | 89.837 | 91.264 | 91.899 | 92.996 |
| 726 | 97.785 | 99.442 | 98.485 | 97.675 | 98.500 | 98.593 | 99.233 | 98.280 | 98.972 | 98.184 | 98.152 | 97.860 | 97.855 | 97.606 | 96.718 | 97.847 | 96.846 | 97.895 | 94.463 | 95.002 | 89.008 | 92.701 | 97.096 | 96.612 | 97.648 | 96.669 | 97.445 | 96.468 | 97.390 | 96.071 | 97.008 | 93.121 | 95.936 | 93.939 | 92.109 | 93.168 | 90.534 | 91.626 | 89.867 | 91.288 | 91.898 | 92.953 |
| 727 | 97.795 | 99.474 | 98.499 | 97.693 | 98.501 | 98.675 | 99.220 | 98.321 | 99.010 | 98.207 | 98.183 | 97.913 | 97.884 | 97.675 | 96.712 | 97.889 | 96.838 | 97.911 | 94.465 | 95.040 | 89.023 | 92.730 | 97.100 | 96.669 | 97.701 | 96.730 | 97.470 | 96.521 | 97.397 | 96.165 | 96.986 | 93.139 | 96.028 | 93.991 | 92.147 | 93.231 | 90.642 | 91.719 | 89.911 | 91.368 | 91.937 | 93.021 |
| 728 | 97.792 | 99.477 | 98.479 | 97.718 | 98.499 | 98.701 | 99.245 | 98.358 | 99.022 | 98.207 | 98.248 | 98.027 | 97.911 | 97.757 | 96.741 | 97.928 | 96.949 | 97.968 | 94.484 | 95.074 | 89.074 | 92.765 | 97.133 | 96.709 | 97.810 | 96.769 | 97.576 | 96.535 | 97.456 | 96.199 | 97.037 | 93.169 | 96.074 | 94.023 | 92.197 | 93.295 | 90.716 | 91.756 | 89.974 | 91.441 | 91.967 | 93.093 |
| 729 | 97.753 | 99.446 | 98.438 | 97.737 | 98.452 | 98.677 | 99.247 | 98.376 | 99.015 | 98.225 | 98.307 | 98.068 | 97.932 | 97.773 | 96.797 | 97.941 | 96.963 | 98.020 | 94.524 | 95.070 | 89.121 | 92.814 | 97.168 | 96.739 | 97.835 | 96.785 | 97.638 | 96.543 | 97.485 | 96.211 | 97.074 | 93.204 | 96.104 | 94.032 | 92.245 | 93.321 | 90.815 | 91.808 | 90.029 | 91.466 | 92.004 | 93.074 |
| 730 | 97.784 | 99.458 | 98.451 | 97.720 | 98.473 | 98.669 | 99.271 | 98.322 | 99.027 | 98.221 | 98.354 | 98.065 | 97.945 | 97.762 | 96.832 | 97.938 | 97.018 | 98.023 | 94.568 | 95.115 | 89.173 | 92.855 | 97.228 | 96.725 | 97.846 | 96.724 | 97.675 | 96.574 | 97.519 | 96.214 | 97.136 | 93.208 | 96.109 | 94.047 | 92.314 | 93.332 | 90.872 | 91.827 | 90.091 | 91.494 | 92.003 | 93.054 |
| 731 | 97.801 | 99.421 | 98.439 | 97.697 | 98.506 | 98.634 | 99.308 | 98.291 | 99.050 | 98.197 | 98.340 | 98.032 | 97.925 | 97.742 | 96.821 | 97.918 | 96.967 | 97.987 | 94.640 | 95.102 | 89.219 | 92.831 | 97.296 | 96.705 | 97.857 | 96.696 | 97.660 | 96.571 | 97.503 | 96.192 | 97.142 | 93.202 | 96.110 | 94.036 | 92.372 | 93.326 | 90.910 | 91.850 | 90.138 | 91.499 | 92.034 | 93.035 |
| 732 | 97.780 | 99.420 | 98.434 | 97.663 | 98.526 | 98.597 | 99.332 | 98.254 | 99.090 | 98.165 | 98.366 | 98.026 | 97.942 | 97.762 | 96.792 | 97.876 | 96.979 | 97.986 | 94.645 | 95.096 | 89.250 | 92.863 | 97.287 | 96.695 | 97.856 | 96.695 | 97.662 | 96.538 | 97.547 | 96.174 | 97.155 | 93.214 | 96.114 | 94.066 | 92.432 | 93.382 | 90.971 | 91.879 | 90.198 | 91.546 | 92.089 | 93.062 |
| 733 | 97.783 | 99.367 | 98.478 | 97.582 | 98.484 | 98.539 | 99.312 | 98.220 | 99.059 | 98.132 | 98.374 | 98.023 | 97.947 | 97.768 | 96.770 | 97.859 | 96.964 | 97.972 | 94.610 | 95.082 | 89.250 | 92.862 | 97.305 | 96.688 | 97.861 | 96.678 | 97.669 | 96.504 | 97.584 | 96.170 | 97.239 | 93.228 | 96.116 | 94.062 | 92.449 | 93.433 | 91.008 | 91.895 | 90.221 | 91.582 | 92.124 | 93.100 |
| 734 | 97.788 | 99.454 | 98.514 | 97.594 | 98.483 | 98.573 | 99.282 | 98.307 | 99.046 | 98.204 | 98.404 | 98.089 | 97.978 | 97.776 | 96.825 | 97.888 | 97.007 | 98.000 | 94.604 | 95.131 | 89.248 | 92.970 | 97.313 | 96.769 | 97.807 | 96.754 | 97.661 | 96.543 | 97.563 | 96.227 | 97.260 | 93.281 | 96.186 | 94.092 | 92.481 | 93.494 | 91.057 | 91.972 | 90.262 | 91.649 | 92.152 | 93.159 |
| 735 | 97.846 | 99.591 | 98.531 | 97.693 | 98.532 | 98.676 | 99.268 | 98.420 | 99.026 | 98.321 | 98.416 | 98.155 | 97.975 | 97.845 | 96.841 | 97.970 | 97.008 | 98.111 | 94.578 | 95.246 | 89.246 | 93.088 | 97.283 | 96.832 | 97.783 | 96.812 | 97.632 | 96.635 | 97.550 | 96.323 | 97.270 | 93.343 | 96.206 | 94.194 | 92.510 | 93.598 | 91.064 | 92.053 | 90.295 | 91.796 | 92.127 | 93.252 |
| 736 | 97.848 | 99.586 | 98.505 | 97.758 | 98.570 | 98.713 | 99.274 | 98.441 | 99.029 | 98.342 | 98.401 | 98.146 | 97.979 | 97.864 | 96.828 | 97.979 | 97.039 | 98.078 | 94.602 | 95.275 | 89.298 | 93.107 | 97.299 | 96.839 | 97.762 | 96.853 | 97.633 | 96.657 | 97.528 | 96.360 | 97.175 | 93.357 | 96.224 | 94.203 | 92.489 | 93.619 | 91.124 | 92.060 | 90.330 | 91.872 | 92.158 | 93.252 |
| 737 | 97.823 | 99.595 | 98.503 | 97.758 | 98.602 | 98.746 | 99.264 | 98.397 | 99.049 | 98.336 | 98.421 | 98.122 | 97.989 | 97.893 | 96.835 | 97.981 | 97.089 | 98.113 | 94.625 | 95.303 | 89.357 | 93.126 | 97.299 | 96.873 | 97.804 | 96.840 | 97.658 | 96.669 | 97.537 | 96.387 | 97.150 | 93.394 | 96.232 | 94.222 | 92.457 | 93.671 | 91.177 | 92.089 | 90.398 | 91.954 | 92.174 | 93.240 |
| 738 | 97.821 | 99.527 | 98.510 | 97.710 | 98.587 | 98.678 | 99.251 | 98.349 | 99.051 | 98.285 | 98.414 | 98.125 | 98.005 | 97.905 | 96.825 | 97.951 | 97.068 | 98.108 | 94.667 | 95.288 | 89.408 | 93.142 | 97.335 | 96.885 | 97.809 | 96.803 | 97.631 | 96.666 | 97.534 | 96.352 | 97.138 | 93.393 | 96.240 | 94.228 | 92.439 | 93.641 | 91.176 | 92.122 | 90.441 | 91.987 | 92.176 | 93.244 |
| 739 | 97.857 | 99.499 | 98.559 | 97.743 | 98.606 | 98.671 | 99.243 | 98.361 | 99.040 | 98.325 | 98.403 | 98.155 | 98.004 | 97.928 | 96.865 | 97.963 | 97.128 | 98.102 | 94.726 | 95.283 | 89.457 | 93.230 | 97.374 | 96.876 | 97.785 | 96.849 | 97.633 | 96.659 | 97.540 | 96.326 | 97.144 | 93.405 | 96.245 | 94.234 | 92.477 | 93.635 | 91.202 | 92.188 | 90.528 | 92.048 | 92.189 | 93.241 |
| 740 | 97.908 | 99.480 | 98.570 | 97.751 | 98.607 | 98.672 | 99.262 | 98.355 | 99.035 | 98.353 | 98.413 | 98.159 | 98.047 | 97.940 | 96.895 | 97.989 | 97.117 | 98.097 | 94.750 | 95.329 | 89.526 | 93.303 | 97.421 | 96.843 | 97.789 | 96.843 | 97.630 | 96.661 | 97.569 | 96.342 | 97.235 | 93.413 | 96.269 | 94.228 | 92.498 | 93.677 | 91.278 | 92.240 | 90.628 | 92.123 | 92.222 | 93.292 |
| 741 | 97.874 | 99.430 | 98.541 | 97.674 | 98.581 | 98.620 | 99.272 | 98.251 | 99.033 | 98.272 | 98.380 | 98.149 | 98.038 | 97.927 | 96.869 | 97.967 | 97.115 | 98.037 | 94.763 | 95.322 | 89.568 | 93.288 | 97.442 | 96.786 | 97.766 | 96.796 | 97.597 | 96.627 | 97.557 | 96.352 | 97.240 | 93.374 | 96.295 | 94.156 | 92.516 | 93.657 | 91.355 | 92.251 | 90.689 | 92.144 | 92.239 | 93.291 |
| 742 | 97.834 | 99.494 | 98.529 | 97.694 | 98.573 | 98.686 | 99.303 | 98.304 | 99.056 | 98.281 | 98.434 | 98.144 | 98.047 | 97.981 | 96.851 | 97.981 | 97.055 | 98.119 | 94.743 | 95.414 | 89.611 | 93.357 | 97.426 | 96.857 | 97.810 | 96.826 | 97.597 | 96.655 | 97.587 | 96.379 | 97.271 | 93.420 | 96.305 | 94.221 | 92.533 | 93.749 | 91.389 | 92.355 | 90.772 | 92.254 | 92.229 | 93.385 |
| 743 | 97.774 | 99.507 | 98.497 | 97.715 | 98.572 | 98.711 | 99.303 | 98.343 | 99.095 | 98.325 | 98.540 | 98.165 | 98.082 | 97.984 | 96.848 | 97.970 | 97.144 | 98.110 | 94.778 | 95.477 | 89.709 | 93.434 | 97.506 | 96.923 | 97.855 | 96.822 | 97.640 | 96.667 | 97.640 | 96.309 | 97.275 | 93.441 | 96.295 | 94.244 | 92.628 | 93.729 | 91.433 | 92.394 | 90.852 | 92.346 | 92.288 | 93.408 |
| 744 | 97.788 | 99.526 | 98.513 | 97.765 | 98.567 | 98.755 | 99.258 | 98.380 | 99.090 | 98.401 | 98.571 | 98.175 | 98.066 | 97.944 | 96.861 | 97.992 | 97.147 | 98.111 | 94.842 | 95.511 | 89.789 | 93.547 | 97.559 | 96.975 | 97.876 | 96.852 | 97.673 | 96.700 | 97.667 | 96.262 | 97.276 | 93.471 | 96.302 | 94.247 | 92.722 | 93.732 | 91.446 | 92.430 | 90.942 | 92.454 | 92.312 | 93.393 |
| 745 | 97.799 | 99.557 | 98.499 | 97.757 | 98.544 | 98.757 | 99.224 | 98.326 | 99.067 | 98.397 | 98.542 | 98.148 | 98.056 | 97.917 | 96.890 | 97.997 | 97.124 | 98.084 | 94.876 | 95.554 | 89.838 | 93.616 | 97.542 | 96.974 | 97.870 | 96.848 | 97.677 | 96.677 | 97.645 | 96.245 | 97.254 | 93.454 | 96.307 | 94.297 | 92.759 | 93.758 | 91.449 | 92.433 | 90.997 | 92.523 | 92.347 | 93.385 |
| 746 | 97.817 | 99.527 | 98.484 | 97.751 | 98.516 | 98.765 | 99.217 | 98.274 | 99.015 | 98.391 | 98.471 | 98.163 | 98.048 | 97.948 | 96.894 | 97.993 | 97.098 | 98.103 | 94.911 | 95.577 | 89.873 | 93.680 | 97.514 | 96.979 | 97.825 | 96.868 | 97.636 | 96.675 | 97.585 | 96.301 | 97.208 | 93.475 | 96.303 | 94.316 | 92.758 | 93.805 | 91.503 | 92.463 | 91.077 | 92.618 | 92.334 | 93.413 |
| 747 | 97.876 | 99.503 | 98.490 | 97.759 | 98.525 | 98.798 | 99.222 | 98.298 | 99.052 | 98.438 | 98.511 | 98.200 | 98.115 | 97.990 | 96.909 | 97.983 | 97.118 | 98.137 | 94.927 | 95.658 | 89.940 | 93.788 | 97.562 | 97.024 | 97.825 | 96.900 | 97.637 | 96.683 | 97.607 | 96.337 | 97.255 | 93.506 | 96.284 | 94.367 | 92.766 | 93.887 | 91.561 | 92.540 | 91.132 | 92.718 | 92.322 | 93.473 |
| 748 | 97.903 | 99.500 | 98.501 | 97.789 | 98.559 | 98.803 | 99.198 | 98.291 | 99.038 | 98.455 | 98.561 | 98.261 | 98.106 | 98.047 | 96.886 | 97.977 | 97.142 | 98.191 | 94.976 | 95.719 | 90.009 | 93.867 | 97.585 | 97.083 | 97.784 | 96.926 | 97.646 | 96.740 | 97.620 | 96.358 | 97.285 | 93.527 | 96.301 | 94.348 | 92.797 | 93.908 | 91.628 | 92.593 | 91.231 | 92.810 | 92.341 | 93.493 |
| 749 | 97.848 | 99.496 | 98.479 | 97.816 | 98.559 | 98.803 | 99.185 | 98.282 | 99.041 | 98.468 | 98.552 | 98.264 | 98.059 | 98.054 | 96.886 | 98.006 | 97.103 | 98.195 | 95.036 | 95.737 | 90.059 | 93.954 | 97.529 | 97.126 | 97.764 | 96.935 | 97.654 | 96.797 | 97.596 | 96.404 | 97.295 | 93.529 | 96.345 | 94.357 | 92.809 | 93.925 | 91.707 | 92.624 | 91.327 | 92.875 | 92.353 | 93.515 |
| 750 | 97.781 | 99.514 | 98.486 | 97.783 | 98.571 | 98.767 | 99.196 | 98.260 | 99.073 | 98.436 | 98.552 | 98.221 | 98.066 | 98.054 | 96.898 | 98.026 | 97.086 | 98.163 | 95.072 | 95.771 | 90.122 | 94.014 | 97.504 | 97.132 | 97.770 | 96.922 | 97.668 | 96.793 | 97.569 | 96.415 | 97.277 | 93.530 | 96.352 | 94.376 | 92.814 | 93.928 | 91.780 | 92.620 | 91.401 | 92.929 | 92.380 | 93.503 |
| 751 | 97.752 | 99.503 | 98.483 | 97.716 | 98.558 | 98.714 | 99.223 | 98.240 | 99.123 | 98.416 | 98.616 | 98.208 | 98.109 | 98.073 | 96.920 | 98.028 | 97.082 | 98.151 | 95.163 | 95.802 | 90.220 | 94.046 | 97.533 | 97.132 | 97.750 | 96.903 | 97.671 | 96.774 | 97.611 | 96.407 | 97.306 | 93.551 | 96.413 | 94.363 | 92.833 | 93.897 | 91.871 | 92.653 | 91.507 | 92.996 | 92.389 | 93.507 |
| 752 | 97.755 | 99.532 | 98.462 | 97.744 | 98.549 | 98.708 | 99.234 | 98.265 | 99.155 | 98.457 | 98.642 | 98.234 | 98.173 | 98.111 | 96.917 | 98.023 | 97.121 | 98.163 | 95.208 | 95.892 | 90.355 | 94.132 | 97.572 | 97.126 | 97.763 | 96.900 | 97.675 | 96.767 | 97.659 | 96.408 | 97.357 | 93.580 | 96.432 | 94.422 | 92.880 | 93.877 | 91.923 | 92.747 | 91.606 | 93.137 | 92.409 | 93.566 |
| 753 | 97.794 | 99.572 | 98.492 | 97.772 | 98.583 | 98.734 | 99.189 | 98.295 | 99.114 | 98.507 | 98.619 | 98.247 | 98.199 | 98.128 | 96.889 | 98.020 | 97.056 | 98.192 | 95.218 | 95.972 | 90.474 | 94.215 | 97.580 | 97.148 | 97.759 | 96.936 | 97.667 | 96.789 | 97.673 | 96.441 | 97.352 | 93.626 | 96.442 | 94.433 | 92.899 | 93.863 | 91.982 | 92.831 | 91.722 | 93.250 | 92.413 | 93.592 |
| 754 | 97.810 | 99.591 | 98.484 | 97.821 | 98.577 | 98.785 | 99.196 | 98.315 | 99.117 | 98.524 | 98.602 | 98.253 | 98.203 | 98.119 | 96.913 | 98.032 | 97.107 | 98.194 | 95.296 | 96.075 | 90.574 | 94.354 | 97.634 | 97.166 | 97.804 | 96.959 | 97.715 | 96.820 | 97.701 | 96.474 | 97.377 | 93.625 | 96.432 | 94.458 | 92.939 | 93.918 | 92.042 | 92.891 | 91.787 | 93.346 | 92.419 | 93.624 |
| 755 | 97.844 | 99.623 | 98.473 | 97.808 | 98.574 | 98.807 | 99.223 | 98.336 | 99.121 | 98.512 | 98.662 | 98.300 | 98.207 | 98.126 | 96.917 | 98.024 | 97.060 | 98.225 | 95.373 | 96.189 | 90.616 | 94.463 | 97.698 | 97.221 | 97.868 | 96.992 | 97.762 | 96.858 | 97.696 | 96.476 | 97.419 | 93.630 | 96.451 | 94.479 | 92.931 | 93.998 | 92.102 | 92.924 | 91.867 | 93.427 | 92.440 | 93.663 |
| 756 | 97.838 | 99.581 | 98.474 | 97.748 | 98.574 | 98.768 | 99.298 | 98.326 | 99.177 | 98.506 | 98.724 | 98.268 | 98.224 | 98.130 | 96.955 | 97.976 | 97.186 | 98.175 | 95.462 | 96.202 | 90.718 | 94.505 | 97.744 | 97.229 | 97.923 | 96.952 | 97.836 | 96.853 | 97.732 | 96.449 | 97.461 | 93.604 | 96.526 | 94.482 | 92.956 | 94.010 | 92.194 | 92.944 | 91.977 | 93.466 | 92.500 | 93.649 |
| 757 | 97.822 | 99.503 | 98.477 | 97.727 | 98.568 | 98.720 | 99.322 | 98.291 | 99.198 | 98.476 | 98.750 | 98.232 | 98.253 | 98.094 | 96.973 | 97.934 | 97.186 | 98.119 | 95.468 | 96.181 | 90.808 | 94.540 | 97.740 | 97.156 | 97.979 | 96.920 | 97.862 | 96.798 | 97.755 | 96.398 | 97.456 | 93.572 | 96.549 | 94.477 | 92.967 | 93.997 | 92.254 | 92.952 | 92.088 | 93.485 | 92.534 | 93.651 |
| 758 | 97.813 | 99.530 | 98.505 | 97.777 | 98.581 | 98.750 | 99.273 | 98.332 | 99.167 | 98.524 | 98.743 | 98.268 | 98.266 | 98.128 | 96.980 | 97.984 | 97.209 | 98.136 | 95.502 | 96.208 | 90.896 | 94.638 | 97.755 | 97.182 | 97.931 | 96.957 | 97.825 | 96.805 | 97.760 | 96.439 | 97.441 | 93.601 | 96.554 | 94.515 | 92.970 | 94.041 | 92.295 | 93.051 | 92.171 | 93.556 | 92.564 | 93.688 |
| 759 | 97.783 | 99.564 | 98.492 | 97.839 | 98.551 | 98.761 | 99.231 | 98.363 | 99.116 | 98.584 | 98.693 | 98.300 | 98.256 | 98.182 | 96.907 | 98.024 | 97.179 | 98.188 | 95.529 | 96.240 | 90.938 | 94.749 | 97.746 | 97.230 | 97.850 | 96.939 | 97.766 | 96.848 | 97.725 | 96.461 | 97.410 | 93.628 | 96.503 | 94.557 | 92.947 | 94.091 | 92.271 | 93.132 | 92.186 | 93.676 | 92.531 | 93.737 |
| 760 | 97.803 | 99.596 | 98.493 | 97.845 | 98.539 | 98.807 | 99.206 | 98.410 | 99.093 | 98.677 | 98.719 | 98.330 | 98.225 | 98.219 | 96.852 | 98.088 | 97.203 | 98.251 | 95.550 | 96.337 | 90.997 | 94.841 | 97.751 | 97.316 | 97.835 | 97.002 | 97.728 | 96.912 | 97.696 | 96.517 | 97.399 | 93.677 | 96.480 | 94.616 | 92.948 | 94.148 | 92.282 | 93.230 | 92.231 | 93.785 | 92.528 | 93.763 |
| 761 | 97.797 | 99.573 | 98.472 | 97.833 | 98.513 | 98.787 | 99.213 | 98.372 | 99.098 | 98.669 | 98.696 | 98.314 | 98.185 | 98.196 | 96.872 | 98.088 | 97.198 | 98.230 | 95.568 | 96.386 | 91.079 | 94.913 | 97.768 | 97.307 | 97.844 | 97.005 | 97.730 | 96.909 | 97.681 | 96.532 | 97.393 | 93.664 | 96.466 | 94.601 | 92.952 | 94.148 | 92.293 | 93.262 | 92.304 | 93.811 | 92.524 | 93.719 |
| 762 | 97.785 | 99.531 | 98.441 | 97.811 | 98.504 | 98.754 | 99.236 | 98.317 | 99.133 | 98.612 | 98.728 | 98.328 | 98.231 | 98.117 | 96.866 | 98.053 | 97.211 | 98.184 | 95.643 | 96.392 | 91.174 | 94.965 | 97.814 | 97.278 | 97.912 | 96.988 | 97.768 | 96.856 | 97.712 | 96.479 | 97.436 | 93.644 | 96.518 | 94.588 | 93.000 | 94.136 | 92.356 | 93.254 | 92.405 | 93.819 | 92.568 | 93.697 |
| 763 | 97.782 | 99.541 | 98.432 | 97.862 | 98.512 | 98.802 | 99.241 | 98.313 | 99.142 | 98.653 | 98.692 | 98.334 | 98.271 | 98.133 | 96.871 | 98.025 | 97.339 | 98.201 | 95.712 | 96.435 | 91.242 | 95.065 | 97.820 | 97.325 | 97.910 | 96.982 | 97.786 | 96.887 | 97.757 | 96.457 | 97.479 | 93.684 | 96.556 | 94.612 | 93.048 | 94.167 | 92.417 | 93.306 | 92.444 | 93.905 | 92.603 | 93.747 |
| 764 | 97.775 | 99.526 | 98.420 | 97.874 | 98.498 | 98.778 | 99.240 | 98.307 | 99.143 | 98.681 | 98.655 | 98.315 | 98.276 | 98.155 | 96.805 | 97.980 | 97.353 | 98.224 | 95.755 | 96.468 | 91.256 | 95.145 | 97.825 | 97.328 | 97.873 | 97.002 | 97.769 | 96.884 | 97.791 | 96.458 | 97.464 | 93.696 | 96.550 | 94.648 | 93.032 | 94.216 | 92.456 | 93.359 | 92.463 | 93.997 | 92.622 | 93.797 |
| 765 | 97.778 | 99.502 | 98.418 | 97.841 | 98.504 | 98.778 | 99.250 | 98.290 | 99.159 | 98.675 | 98.669 | 98.308 | 98.286 | 98.207 | 96.776 | 97.980 | 97.394 | 98.194 | 95.803 | 96.497 | 91.344 | 95.242 | 97.857 | 97.357 | 97.911 | 97.056 | 97.770 | 96.836 | 97.770 | 96.500 | 97.441 | 93.669 | 96.539 | 94.681 | 93.011 | 94.225 | 92.514 | 93.403 | 92.552 | 94.068 | 92.697 | 93.824 |
| 766 | 97.775 | 99.473 | 98.419 | 97.762 | 98.503 | 98.732 | 99.254 | 98.234 | 99.148 | 98.605 | 98.695 | 98.298 | 98.293 | 98.223 | 96.727 | 97.985 | 97.207 | 98.151 | 95.828 | 96.470 | 91.398 | 95.228 | 97.889 | 97.330 | 97.895 | 97.005 | 97.759 | 96.798 | 97.716 | 96.486 | 97.403 | 93.623 | 96.514 | 94.625 | 92.976 | 94.167 | 92.511 | 93.339 | 92.572 | 94.008 | 92.719 | 93.763 |
| 767 | 97.808 | 99.447 | 98.461 | 97.722 | 98.515 | 98.706 | 99.258 | 98.194 | 99.135 | 98.578 | 98.734 | 98.278 | 98.269 | 98.204 | 96.689 | 97.997 | 97.205 | 98.117 | 95.809 | 96.469 | 91.458 | 95.171 | 97.895 | 97.328 | 97.851 | 96.952 | 97.740 | 96.803 | 97.679 | 96.445 | 97.381 | 93.609 | 96.537 | 94.566 | 92.989 | 94.153 | 92.485 | 93.284 | 92.546 | 93.957 | 92.696 | 93.735 |
| 768 | 97.792 | 99.478 | 98.435 | 97.742 | 98.518 | 98.710 | 99.232 | 98.182 | 99.118 | 98.596 | 98.701 | 98.266 | 98.221 | 98.160 | 96.675 | 97.993 | 97.100 | 98.144 | 95.788 | 96.531 | 91.452 | 95.193 | 97.839 | 97.299 | 97.788 | 96.928 | 97.695 | 96.843 | 97.706 | 96.414 | 97.398 | 93.662 | 96.557 | 94.557 | 93.007 | 94.147 | 92.472 | 93.316 | 92.497 | 93.980 | 92.652 | 93.739 |
| 769 | 97.755 | 99.536 | 98.410 | 97.762 | 98.475 | 98.730 | 99.205 | 98.268 | 99.142 | 98.637 | 98.702 | 98.318 | 98.218 | 98.170 | 96.664 | 98.005 | 97.198 | 98.192 | 95.854 | 96.646 | 91.505 | 95.306 | 97.853 | 97.317 | 97.798 | 96.965 | 97.689 | 96.883 | 97.762 | 96.447 | 97.409 | 93.700 | 96.572 | 94.605 | 93.075 | 94.178 | 92.512 | 93.359 | 92.553 | 94.104 | 92.682 | 93.770 |
| 770 | 97.730 | 99.555 | 98.395 | 97.770 | 98.485 | 98.803 | 99.187 | 98.342 | 99.135 | 98.679 | 98.694 | 98.392 | 98.238 | 98.221 | 96.674 | 98.051 | 97.130 | 98.244 | 95.905 | 96.725 | 91.591 | 95.444 | 97.831 | 97.335 | 97.834 | 97.006 | 97.706 | 96.914 | 97.759 | 96.512 | 97.421 | 93.735 | 96.573 | 94.687 | 93.120 | 94.226 | 92.539 | 93.414 | 92.619 | 94.189 | 92.715 | 93.810 |
| 771 | 97.732 | 99.578 | 98.357 | 97.793 | 98.498 | 98.843 | 99.189 | 98.408 | 99.134 | 98.683 | 98.716 | 98.432 | 98.308 | 98.285 | 96.696 | 98.070 | 97.163 | 98.279 | 95.980 | 96.752 | 91.644 | 95.519 | 97.867 | 97.375 | 97.874 | 97.084 | 97.692 | 96.939 | 97.772 | 96.541 | 97.469 | 93.741 | 96.585 | 94.730 | 93.169 | 94.270 | 92.588 | 93.424 | 92.635 | 94.237 | 92.743 | 93.863 |
| 772 | 97.789 | 99.557 | 98.417 | 97.816 | 98.537 | 98.824 | 99.228 | 98.397 | 99.129 | 98.673 | 98.754 | 98.396 | 98.318 | 98.253 | 96.711 | 98.067 | 97.104 | 98.264 | 96.018 | 96.735 | 91.704 | 95.553 | 97.873 | 97.394 | 97.877 | 97.096 | 97.713 | 96.925 | 97.780 | 96.561 | 97.514 | 93.759 | 96.627 | 94.765 | 93.178 | 94.248 | 92.616 | 93.440 | 92.662 | 94.244 | 92.753 | 93.842 |
| 773 | 97.799 | 99.565 | 98.420 | 97.807 | 98.561 | 98.827 | 99.226 | 98.388 | 99.155 | 98.643 | 98.814 | 98.362 | 98.300 | 98.273 | 96.704 | 98.075 | 97.037 | 98.246 | 96.037 | 96.753 | 91.722 | 95.611 | 97.888 | 97.376 | 97.879 | 97.055 | 97.744 | 96.942 | 97.783 | 96.583 | 97.504 | 93.810 | 96.651 | 94.777 | 93.185 | 94.230 | 92.667 | 93.492 | 92.656 | 94.316 | 92.759 | 93.850 |
| 774 | 97.765 | 99.563 | 98.418 | 97.810 | 98.537 | 98.840 | 99.192 | 98.407 | 99.165 | 98.688 | 98.791 | 98.353 | 98.317 | 98.310 | 96.690 | 98.010 | 97.037 | 98.264 | 96.054 | 96.810 | 91.792 | 95.691 | 97.933 | 97.411 | 97.891 | 97.035 | 97.778 | 96.945 | 97.772 | 96.574 | 97.474 | 93.835 | 96.656 | 94.820 | 93.211 | 94.285 | 92.699 | 93.541 | 92.694 | 94.379 | 92.767 | 93.853 |
| 775 | 97.769 | 99.577 | 98.410 | 97.833 | 98.555 | 98.844 | 99.098 | 98.403 | 99.100 | 98.735 | 98.728 | 98.364 | 98.275 | 98.310 | 96.604 | 98.006 | 96.949 | 98.304 | 96.000 | 96.836 | 91.809 | 95.780 | 97.907 | 97.439 | 97.791 | 97.052 | 97.719 | 96.933 | 97.720 | 96.555 | 97.407 | 93.846 | 96.609 | 94.828 | 93.158 | 94.335 | 92.645 | 93.612 | 92.679 | 94.392 | 92.711 | 93.891 |
| 776 | 97.780 | 99.578 | 98.429 | 97.806 | 98.552 | 98.810 | 99.094 | 98.392 | 99.107 | 98.723 | 98.726 | 98.387 | 98.310 | 98.296 | 96.608 | 97.999 | 97.217 | 98.281 | 96.043 | 96.852 | 91.900 | 95.836 | 97.918 | 97.452 | 97.782 | 97.094 | 97.675 | 96.869 | 97.750 | 96.541 | 97.417 | 93.788 | 96.587 | 94.815 | 93.175 | 94.341 | 92.668 | 93.598 | 92.710 | 94.400 | 92.722 | 93.928 |
| 777 | 97.783 | 99.564 | 98.460 | 97.826 | 98.561 | 98.826 | 99.140 | 98.344 | 99.177 | 98.693 | 98.776 | 98.386 | 98.319 | 98.289 | 96.679 | 98.037 | 97.170 | 98.263 | 96.116 | 96.918 | 91.995 | 95.833 | 97.921 | 97.413 | 97.803 | 97.070 | 97.693 | 96.861 | 97.799 | 96.529 | 97.454 | 93.797 | 96.641 | 94.796 | 93.195 | 94.354 | 92.683 | 93.622 | 92.772 | 94.408 | 92.792 | 93.907 |
| 778 | 97.797 | 99.573 | 98.434 | 97.796 | 98.551 | 98.816 | 99.158 | 98.342 | 99.220 | 98.678 | 98.785 | 98.388 | 98.357 | 98.348 | 96.774 | 98.052 | 97.396 | 98.279 | 96.193 | 96.945 | 92.043 | 95.869 | 97.971 | 97.411 | 97.871 | 97.044 | 97.722 | 96.875 | 97.837 | 96.539 | 97.497 | 93.777 | 96.697 | 94.822 | 93.236 | 94.362 | 92.757 | 93.668 | 92.823 | 94.491 | 92.851 | 93.953 |
| 779 | 97.824 | 99.535 | 98.453 | 97.808 | 98.577 | 98.832 | 99.175 | 98.312 | 99.225 | 98.685 | 98.809 | 98.363 | 98.438 | 98.293 | 96.842 | 98.011 | 97.256 | 98.313 | 96.287 | 96.955 | 92.106 | 95.923 | 98.031 | 97.402 | 97.956 | 97.018 | 97.811 | 96.892 | 97.854 | 96.505 | 97.530 | 93.801 | 96.734 | 94.828 | 93.259 | 94.361 | 92.812 | 93.656 | 92.877 | 94.486 | 92.900 | 93.920 |
| 780 | 97.847 | 99.525 | 98.442 | 97.758 | 98.587 | 98.812 | 99.152 | 98.271 | 99.156 | 98.672 | 98.805 | 98.369 | 98.420 | 98.274 | 96.842 | 98.062 | 97.460 | 98.290 | 96.263 | 96.918 | 92.134 | 95.930 | 98.016 | 97.396 | 97.918 | 97.013 | 97.795 | 96.924 | 97.798 | 96.509 | 97.485 | 93.780 | 96.688 | 94.800 | 93.203 | 94.344 | 92.817 | 93.641 | 92.874 | 94.522 | 92.870 | 93.940 |
| 781 | 97.843 | 99.568 | 98.450 | 97.735 | 98.583 | 98.814 | 99.182 | 98.327 | 99.170 | 98.679 | 98.792 | 98.411 | 98.408 | 98.302 | 96.895 | 98.063 | 97.380 | 98.254 | 96.272 | 96.914 | 92.168 | 95.965 | 98.011 | 97.444 | 97.916 | 97.041 | 97.770 | 96.912 | 97.816 | 96.567 | 97.519 | 93.798 | 96.688 | 94.863 | 93.186 | 94.369 | 92.848 | 93.634 | 92.909 | 94.573 | 92.853 | 93.944 |
| 782 | 97.863 | 99.586 | 98.485 | 97.781 | 98.594 | 98.837 | 99.159 | 98.358 | 99.156 | 98.680 | 98.791 | 98.421 | 98.355 | 98.356 | 96.840 | 98.088 | 97.439 | 98.274 | 96.232 | 96.958 | 92.154 | 95.937 | 97.980 | 97.383 | 97.829 | 97.049 | 97.705 | 96.900 | 97.795 | 96.591 | 97.445 | 93.830 | 96.700 | 94.857 | 93.153 | 94.380 | 92.808 | 93.647 | 92.917 | 94.605 | 92.853 | 93.951 |
| 783 | 97.880 | 99.626 | 98.527 | 97.819 | 98.600 | 98.847 | 99.142 | 98.412 | 99.133 | 98.743 | 98.781 | 98.450 | 98.354 | 98.393 | 96.780 | 98.115 | 97.480 | 98.336 | 96.276 | 97.038 | 92.191 | 96.001 | 97.967 | 97.413 | 97.816 | 97.075 | 97.765 | 96.902 | 97.770 | 96.661 | 97.396 | 93.859 | 96.718 | 94.917 | 93.164 | 94.405 | 92.781 | 93.692 | 92.956 | 94.635 | 92.848 | 94.016 |
| 784 | 97.876 | 99.637 | 98.517 | 97.910 | 98.598 | 98.909 | 99.145 | 98.389 | 99.156 | 98.778 | 98.868 | 98.452 | 98.414 | 98.414 | 96.788 | 98.140 | 97.397 | 98.385 | 96.308 | 97.136 | 92.291 | 96.103 | 98.005 | 97.403 | 97.900 | 97.063 | 97.835 | 96.963 | 97.767 | 96.650 | 97.399 | 93.931 | 96.723 | 94.919 | 93.166 | 94.450 | 92.769 | 93.771 | 92.980 | 94.668 | 92.886 | 94.042 |
| 785 | 97.875 | 99.621 | 98.502 | 97.858 | 98.611 | 98.871 | 99.150 | 98.355 | 99.169 | 98.768 | 98.882 | 98.464 | 98.398 | 98.405 | 96.819 | 98.115 | 97.468 | 98.335 | 96.342 | 97.116 | 92.320 | 96.154 | 98.016 | 97.471 | 97.940 | 97.067 | 97.823 | 96.976 | 97.759 | 96.648 | 97.451 | 93.908 | 96.679 | 94.938 | 93.184 | 94.478 | 92.795 | 93.817 | 92.938 | 94.683 | 92.880 | 94.035 |
| 786 | 97.886 | 99.601 | 98.516 | 97.848 | 98.628 | 98.877 | 99.148 | 98.348 | 99.177 | 98.741 | 98.895 | 98.458 | 98.381 | 98.390 | 96.825 | 98.089 | 97.244 | 98.316 | 96.374 | 97.084 | 92.297 | 96.197 | 98.010 | 97.505 | 97.909 | 97.065 | 97.790 | 96.984 | 97.755 | 96.643 | 97.475 | 93.922 | 96.630 | 94.969 | 93.131 | 94.472 | 92.814 | 93.778 | 92.914 | 94.664 | 92.869 | 93.999 |
| 787 | 97.906 | 99.564 | 98.507 | 97.800 | 98.651 | 98.842 | 99.128 | 98.284 | 99.136 | 98.712 | 98.829 | 98.406 | 98.379 | 98.371 | 96.756 | 98.070 | 97.361 | 98.337 | 96.374 | 97.089 | 92.305 | 96.185 | 98.029 | 97.448 | 97.821 | 97.011 | 97.726 | 96.960 | 97.740 | 96.635 | 97.400 | 93.853 | 96.621 | 94.952 | 93.156 | 94.428 | 92.829 | 93.750 | 92.925 | 94.693 | 92.867 | 94.007 |
| 788 | 97.896 | 99.616 | 98.483 | 97.847 | 98.641 | 98.871 | 99.198 | 98.316 | 99.181 | 98.765 | 98.827 | 98.427 | 98.407 | 98.388 | 96.813 | 98.115 | 97.247 | 98.360 | 96.425 | 97.175 | 92.386 | 96.266 | 98.052 | 97.540 | 97.838 | 97.065 | 97.821 | 96.951 | 97.789 | 96.658 | 97.417 | 93.856 | 96.693 | 95.036 | 93.235 | 94.467 | 92.834 | 93.787 | 93.025 | 94.738 | 92.878 | 94.032 |
| 789 | 97.845 | 99.627 | 98.406 | 97.913 | 98.589 | 98.932 | 99.253 | 98.332 | 99.266 | 98.799 | 98.948 | 98.480 | 98.478 | 98.401 | 96.890 | 98.127 | 97.499 | 98.361 | 96.448 | 97.241 | 92.450 | 96.328 | 98.056 | 97.537 | 97.910 | 97.111 | 97.854 | 97.010 | 97.881 | 96.618 | 97.455 | 93.906 | 96.747 | 95.040 | 93.303 | 94.499 | 92.858 | 93.807 | 93.085 | 94.762 | 92.941 | 94.035 |
| 790 | 97.817 | 99.569 | 98.423 | 97.876 | 98.619 | 98.920 | 99.269 | 98.344 | 99.278 | 98.799 | 98.952 | 98.537 | 98.426 | 98.391 | 96.902 | 98.109 | 97.482 | 98.331 | 96.503 | 97.240 | 92.453 | 96.361 | 98.052 | 97.567 | 97.967 | 97.131 | 97.870 | 97.003 | 97.912 | 96.615 | 97.516 | 93.888 | 96.779 | 95.059 | 93.364 | 94.518 | 92.896 | 93.797 | 93.085 | 94.752 | 92.962 | 94.028 |
| 791 | 97.828 | 99.485 | 98.492 | 97.817 | 98.647 | 98.856 | 99.203 | 98.331 | 99.209 | 98.754 | 98.903 | 98.518 | 98.416 | 98.402 | 96.908 | 98.050 | 97.479 | 98.309 | 96.504 | 97.231 | 92.463 | 96.331 | 98.049 | 97.573 | 97.929 | 97.089 | 97.828 | 96.977 | 97.887 | 96.617 | 97.519 | 93.863 | 96.783 | 95.032 | 93.334 | 94.494 | 92.892 | 93.799 | 93.070 | 94.753 | 92.933 | 94.038 |
| 792 | 97.844 | 99.455 | 98.527 | 97.759 | 98.663 | 98.810 | 99.169 | 98.298 | 99.147 | 98.726 | 98.846 | 98.441 | 98.382 | 98.417 | 96.846 | 98.022 | 97.443 | 98.276 | 96.471 | 97.192 | 92.508 | 96.313 | 98.083 | 97.526 | 97.894 | 97.038 | 97.770 | 96.938 | 97.877 | 96.629 | 97.496 | 93.812 | 96.798 | 94.970 | 93.362 | 94.468 | 92.890 | 93.805 | 93.071 | 94.780 | 92.889 | 94.039 |
| 793 | 97.834 | 99.573 | 98.491 | 97.806 | 98.636 | 98.838 | 99.231 | 98.340 | 99.161 | 98.758 | 98.898 | 98.444 | 98.408 | 98.410 | 96.868 | 98.059 | 97.405 | 98.286 | 96.475 | 97.189 | 92.593 | 96.411 | 98.125 | 97.549 | 97.892 | 97.078 | 97.840 | 96.978 | 97.919 | 96.646 | 97.534 | 93.868 | 96.835 | 95.024 | 93.382 | 94.520 | 92.918 | 93.837 | 93.121 | 94.839 | 92.888 | 94.013 |
| 794 | 97.802 | 99.620 | 98.446 | 97.845 | 98.618 | 98.907 | 99.263 | 98.324 | 99.162 | 98.771 | 98.924 | 98.460 | 98.425 | 98.407 | 96.843 | 98.099 | 97.417 | 98.344 | 96.478 | 97.224 | 92.592 | 96.483 | 98.134 | 97.517 | 97.860 | 97.060 | 97.816 | 97.057 | 97.897 | 96.678 | 97.532 | 93.916 | 96.825 | 95.040 | 93.391 | 94.547 | 92.941 | 93.866 | 93.103 | 94.913 | 92.924 | 94.017 |
| 795 | 97.755 | 99.563 | 98.480 | 97.808 | 98.630 | 98.901 | 99.269 | 98.312 | 99.167 | 98.790 | 98.893 | 98.455 | 98.414 | 98.408 | 96.879 | 98.102 | 97.249 | 98.302 | 96.511 | 97.243 | 92.623 | 96.465 | 98.117 | 97.512 | 97.865 | 97.058 | 97.841 | 97.018 | 97.855 | 96.657 | 97.552 | 93.904 | 96.827 | 95.007 | 93.431 | 94.494 | 92.938 | 93.898 | 93.105 | 94.905 | 92.910 | 93.996 |
| 796 | 97.752 | 99.502 | 98.471 | 97.843 | 98.609 | 98.861 | 99.232 | 98.319 | 99.200 | 98.769 | 98.920 | 98.472 | 98.459 | 98.399 | 96.923 | 98.049 | 97.376 | 98.309 | 96.566 | 97.328 | 92.650 | 96.431 | 98.112 | 97.527 | 97.907 | 97.068 | 97.846 | 97.017 | 97.836 | 96.651 | 97.541 | 93.925 | 96.772 | 95.017 | 93.424 | 94.488 | 92.871 | 93.933 | 93.103 | 94.900 | 92.927 | 93.980 |
| 797 | 97.733 | 99.526 | 98.433 | 97.895 | 98.584 | 98.886 | 99.264 | 98.363 | 99.268 | 98.797 | 98.961 | 98.539 | 98.458 | 98.434 | 96.923 | 98.076 | 97.350 | 98.391 | 96.570 | 97.395 | 92.658 | 96.503 | 98.163 | 97.563 | 97.957 | 97.096 | 97.854 | 97.040 | 97.839 | 96.684 | 97.505 | 93.977 | 96.755 | 95.120 | 93.436 | 94.549 | 92.866 | 93.965 | 93.115 | 94.951 | 92.946 | 94.039 |
| 798 | 97.751 | 99.586 | 98.453 | 97.912 | 98.627 | 98.898 | 99.305 | 98.399 | 99.294 | 98.821 | 98.998 | 98.589 | 98.503 | 98.431 | 96.970 | 98.110 | 97.354 | 98.401 | 96.611 | 97.386 | 92.691 | 96.571 | 98.195 | 97.619 | 97.936 | 97.106 | 97.918 | 97.063 | 97.887 | 96.719 | 97.505 | 93.999 | 96.793 | 95.183 | 93.457 | 94.589 | 92.892 | 93.978 | 93.167 | 94.994 | 92.957 | 94.076 |
| 799 | 97.755 | 99.594 | 98.459 | 97.888 | 98.636 | 98.891 | 99.305 | 98.341 | 99.291 | 98.800 | 98.998 | 98.540 | 98.501 | 98.429 | 96.980 | 98.099 | 97.327 | 98.339 | 96.642 | 97.341 | 92.715 | 96.565 | 98.189 | 97.570 | 97.918 | 97.077 | 97.889 | 97.026 | 97.897 | 96.718 | 97.506 | 93.951 | 96.812 | 95.133 | 93.442 | 94.552 | 92.911 | 93.967 | 93.176 | 94.971 | 92.950 | 94.057 |
| 800 | 97.751 | 99.560 | 98.450 | 97.841 | 98.645 | 98.855 | 99.296 | 98.274 | 99.272 | 98.777 | 98.997 | 98.465 | 98.489 | 98.392 | 97.004 | 98.033 | 97.296 | 98.270 | 96.693 | 97.304 | 92.779 | 96.531 | 98.174 | 97.529 | 97.941 | 97.086 | 97.881 | 96.983 | 97.885 | 96.655 | 97.541 | 93.902 | 96.823 | 95.069 | 93.440 | 94.498 | 92.912 | 93.989 | 93.193 | 94.918 | 92.929 | 94.010 |
